# Supplementary material for: Janus Kinase Inhibitors Improve Disease Activity and Patient-Reported Outcomes in Rheumatoid Arthritis: A Systematic Review and Meta-Analysis of 24,135 Patients
Source: Int J Mol Sci. 2022 Jan 23;23(3):1246. doi: 10.3390/ijms23031246 (PMC8836107; doi:10.3390/ijms23031246)
Supplement: Supplementary file 1 [file ijms-23-01246-s001.zip › Supplementary_material/Supplementary material 1.pdf]

Supplementary material

Study	Year	Population	Total n⁰ of patients	Female (%)*	Disease duration (years)*	Age (years) *	Intervention		Comparator		Concomitant medication
							Type	Daily dose	PBO	Active comparator	
Greenwald 2010 [34] NCT00902486	2010	inadequate response to DMARDs	124	n.a	7 - 9	54 - 58	bari	4,7,10 mg - 1x	+	-	csDMARD
I4V-MC-JADA [32,65,66] NCT01185353	2015	inadequate response to MTX	301	71-87%	5.3 - 6.6	49 - 53	bari	1 mg, 2 mg, 4 mg, 8 mg - 1x	+	-	MTX
RA-BALANCE [31,67] NCT02265705	2020	inadequate response to MTX	290	73.1-87.6%	9.1 - 10.7	48.9 - 49.5	bari	4 mg - 1x	+	-	MTX
RA-BEACON [29,68] NCT01721044	2016	inadequate response to TNFi	527	79-84%	14	55 - 56	bari	2 mg, 4 mg - 1x	+	-	csDMARD
RA-BEAM [28,69] NCT01710358	2017	inadequate response to MTX	1305	76-78%	10	53 - 54	bari	4 mg - 1x	+	adalimumab	csDMARD
RA-BEGIN [59,70,71] NCT01711359	2017	csDMARD naive	584	70-76%	1.3 - 1.9	49 - 51	bari	4 mg - 1x	+	MTX	
RA-BUILD [30,64,72] NCT01721057	2017	inadequate response to csDMARDs	684	80-83%	7 - 8	51 - 52	bari	2 mg, 4 mg - 1x	+	-	csDMARDs
Tanaka 2016 [33] NCT01469013	2016	inadequate response to MTX	145	71-92%	5.06 - 6.32	51.1 - 57.5	bari	1 mg, 2 mg, 4 mg, 8 mg - 1x	+	-	MTX
Fleischmann 2015 [53,73] NCT01052194	2015	inadequate response to MTX	204	78-85%	6.3 - 10	54.9 - 56.8	decerno	25 mg, 50 mg, 100 mg, or 150 mg - 2x	+	-	
Genovese 2016 [51,74] NCT2011-004419-22	2016	inadequate response to MTX	358	71.8-87.3%	6.5-8.1	50.1 - 53.5	decerno	100 mg, 150 mg, 200 mg - 1x, or 100 mg - 2x	+	-	MTX
Genovese 2016 II [52] NCT01754935	2016	inadequate response to DMARDs	43	50-83.3%	6.8 - 11	50.5 - 56.7	decerno	100 mg, 200 mg, 300 mg - 1x	+	-	DMARDs
DARWIN 1 [46] NCT01888874	2016	inadequate response to MTX	594	76.5-86%	7 - 10	52 - 55	filgo	50, 100 or 200 mg 1x, 50, 100 or 200 mg - 2x	+	-	MTX
DARWIN 2 [45] NCT01894516	2017	inadequate response to MTX	283	75.7-87%	9 - 10	52 - 53	filgo	50, 100 or 200 mg - 1x,	+	-	
FINCH 1 [43] NCT02889796	2019	inadequate response to MTX	1755	n.a	n.a	n.a	filgo	100 mg, 200 mg - 1x	+	adalimumab	MTX
FINCH 2 [44] NCT02873936	2019	inadequate response or intolerance to bDMARDs	448	77.8-81.8%	9.8 - 10.3 (median)	55 - 56	filgo	100 mg, 200 mg - 1x	+	-	csDMARD
FINCH 3 [57] NCT02886728	2019	MTX naive	1249	n.a	n.a	n.a	filgo	100 mg, 200 mg, 200 mg (mono) - 1x	+	MTX	
Vanhoutte 2017 I [62] NCT01384422	2017	n.a	36	91.7%	5.6 - 9.7	47 - 53	filgo	200 mg - 1x or 100 mg - 2x	+	-	MTX
Vanhoutte 2017 II [62] NCT01668641	2017	n.a	91	72.7-82.3%	4.4 - 10	44 - 55	filgo	30 mg, 75 mg, 150 mg, or 300 mg - 1x	+	-	MTX
Luchi [63] NCT01626573	2013	n.a	60	73%	n.a	53.2	ita	100 mg, 200 mg - 2x, 300 mg, 60 mg - 1x	+	-	csDMARDs
Genovese 2017 [47,75] NCT01565655	2017	inadequate response to csDMARDs	289	78-87.9%	9.8-10	52.6 - 54.9	pefi	25 mg, 50 mg, 100 mg, 150 mg - 1x	+	-	
Kivitz 2016 [48,76] NCT01554696	2017	inadequate response to MTX	379	81-87.5%	7.2 - 8.1	52.3 - 54.5	pefi	25 mg, 50 mg, 100 mg, or 150 mg - 1x	+	-	MTX

***RAJ3* [50]** NCT02308163	2019	inadequate response to csDMARDs	507	69-76.5%	6.98 - 10.39	54.1 - 56.3	pefi	100 mg, 150 mg - 1x	+	etanercept	MTX
***RAJ4* [49]** NCT02305849	2019	inadequate response to MTX	519	67.8-71.8%	4.3 - 4.41	55.3 - 58.5	pefi	100 mg, 150 mg - 1x	+	-	MTX
***Takeuchi 2016* [60]** NCT01649999	2016	no restrictions	281	76.4-83.6%	6.92 - 8.03	51.6 - 54.2	pefi	25, 50, 100, 150 mg - 1x	+	-	
***Robinson 2020* [54]** NCT02969044	2020	inadequate response to MTX	70	78.6-85.7%	7.1-8.4	54.2 - 55.4	ritle	200 mg - 1x	+	-	MTX
***Boyle 2015* [23]** NCT00976599	2015	Inadequate response to MTX	29	85.7-93.3%	5.5 - 12.2	53.1 - 53.5	tofa	10 mg - 2x	+	-	MTX
***Conaghan 2016* [55]** NCT01164579	2016	MTX naive	109	78.4-86.1%	0.6 - 0.8	47.8 - 50.8	tofa	10 mg - 2x	+	MTX	
***Fleischmann 2012* [21]** NCT00550446	2012	inadequate response to DMARDs	384	85.2-88.1%	8.1 - 10.8	52 - 55	tofa	1mg, 3 mg, 5 mg, 10 mg, 15 mg - 2x	+	adalimumab	
***Kremer 2009* [24,77]** NCT00147498	2009	inadequate response to DMARDs	264	84.1-87%	8.7 - 10.2	47.9 - 51.8	tofa	5 mg, 15 mg, 30 mg - 2x	+	-	
***Kremer 2012* [25]** NCT00413660	2012	inadequate response to DMARDs	507	74.3-88%	7.5 - 11.8	51 - 56	tofa	1mg, 3 mg, 5 mg, 10 mg, 15 mg - 2x, 20 mg - 1x	+	-	MTX
***Menshikova 2018* [61]** n.a	2018	n. a	30	70%	3.5	48.2	tofa	5 mg - 2x	-	etanercept	n. a
***Nakamura 2018* [22]** NCT02157012	2018	inadequate response to DMARDs	50	59-85%	3.4 - 3.5	67.2 - 68.3	tofa	5 mg - 2x	-	tocilizumab, abatacept	MTX
***ORAL Scan* [15,78]** NCT00847613	2013	Inadequate response to MTX	797	80.2 - 91.1%	8.8 - 9.5	52.0 - 53.7	tofa	5 mg, 10 mg - 2x	+	-	MTX
***ORAL Solo* [21,79]** NCT00814307	2012	inadequate response to DMARDs	610	85.2-88.2%	7.7 - 8.6	49.7 - 52.4	tofa	5 mg, 10 mg - 2x	+	-	
***ORAL Standard* [16,80,81]** NCT00853385	2012	Inadequate response to MTX	717	75.0 - 85.3%	6.9 - 9.0	51.9 - 55.5	tofa	5 mg, 10 mg - 2x	+	adalimumab	MTX
***ORAL Start* [58,82,83]** NCT01039688	2014	MTX naive	956	76.7-82.4%	2.9-3.4	48.8 - 50.3	tofa	5 mg, 10 mg - 2x	-	MTX	
***ORAL Step* [18,84]** NCT00960440	2013	inadequate response to TNFi	399	80.3-86,6%	11.3–13.0	54.4 - 55.4	tofa	5 mg, 10 mg - 2x	+	-	MTX
***ORAL Strategy* [20,85,86]** NCT02187055	2017	Inadequate response to MTX	1146	83%	5.4 - 6.1	49.7 - 50.7	tofa	5 mg - 2x	-	adalimumab	MTX
***ORAL Sync* [17,87]** NCT00856544	2013	inadequate response to DMARDs	792	75.0 - 83.8%	8.1 - 10.2	50.8 - 53.3	tofa	5 mg, 10 mg - 2x	+	-	nonbiologic DMARDs
***Tanaka 2011* [26]** NCT00603512	2011	inadequate response to MTX	136	75-96.2%	5.7 - 8.7	50 - 53.3	tofa	1mg, 3 mg, 5 mg, 10 mg - 2x	+	-	MTX
***Tanaka 2015* [27]** NCT00687193	2015	inadequate response to at least one synthetic or bDMARD	317	79.2-88.7 %	6.8 - 11	52.6 - 54.7	tofa	1mg, 3 mg, 5 mg, 10 mg, 15 mg - 2x	+	-	
***BALANCE I* [39]** NCT01960855	2016	inadequate response to TNFi	276	78-86%	10.9 - 12.3	56 - 59	upa	3 mg, 6 mg, 12 mg, or 18 mg - 2x	+	-	MTX
***BALANCE II* [38]** NCT02066389	2016	inadequate response to MTX	299	68-86%	3.9 - 9.3	53 - 56	upa	3 mg, 6 mg, 12 mg, or 18 mg - 2x, or 24 mg - 1x	+	-	MTX
***SELECT-BEYOND* [40,88,89]** NCT02706847	2018	inadequate response to bDMARDs	499	84-85%	12.4 - 14.5	56.3 - 57.6	upa	15 mg, 30 mg - 1x	+	-	csDMARD
***SELECT-CHOICE* [41]** NCT03086343	2020	inadequate response to bDMARDs	612	81.9-82.2%	11.8 - 12.4	55.3 - 55.8	upa	15 mg - 1x	-	abatacept	csDMARD
***SELECT-COMPARE* [36,86]** NCT02629159	2019	inadequate response to MTX	1629	79-80%	8	54	upa	15 mg - 1x	+	adalimumab	MTX

***SELECT-EARLY* [56,90,91]** NCT02706873	2020	MTX naive	945	76-76.4%	2.6-2.9	51.9 - 54.9	upa	15 mg, 30 mg - 1x	-	MTX	
***SELECT-MONOTHERAPY* [35,92]** NCT02706951	2019	inadequate response to MTX	648	79-83%	5.8 - 7.5	53.1 - 55.3	upa	15 mg, 30 mg - 1x	-	MTX	
***SELECT-NEXT* [37,93]** NCT02675426	2018	inadequate response to csDMARDs	661	75-82%	7.2 - 7.3	55.3 - 56	upa	15 mg, 30 mg - 1x	+	-	csDMARD
***SELECT-SUNRISE* [42]** NCT02720523	2020	inadequate response to csDMARDs	197	69.4-86	2.1 - 4 (median)	54.3 - 56	upa	7,5 mg, 15 mg, 30 mg - 1x	+	-	csDMARD

Supplementary Table S1. Characteristics of the included studies

*All included studies are RCTs. *Values are means, ranging among study groups, unless stated otherwise. DMARD denotes disease modifying antirheumatic drugs; csDMARD, conventional synthetic DMARD; bDMARD, biological DMARD; TNFi, tumor necrosis factor inhibitor; MTX, methotrexate; tofa, tofacitinib; bari, baricitinib; upa, upadacitinib; filgo, filgotinib; pefi, peficitinib; decerno, decernotinib; ritle, ritlecitinib, ita, itacitinib and n.a, not available*

Supplementary material

Summary of outcomes

**ACR20 response**	Number of patients who reached at least 20% improvement in measures involved in ACR Core Data Set (SJC, TJC, PtGA, PGA, pain, ESR or CRP)
**ACR50 response**	Number of patients who reached at least 50% improvement in measures involved in ACR Core Data Set (SJC, TJC, PtGA, PGA, pain, ESR or CRP)
**ACR70 response**	Number of patients who reached at least 70% improvement in measures involved in ACR Core Data Set (SJC, TJC, PtGA, PGA, pain, ESR or CRP)
**CDAI difference**	LSM change from baseline in the score on CDAI
**CDAI remission**	Number of patients reaching remission defined by CDAI (≤ 2.8)
**CRP**	LSM change from baseline in CRP value (mg/l)
**DAS28-CRP difference**	LSM change from baseline in the score on DAS28-CRP
**DAS28-CRP remission**	Number of patients reaching remission defined by DAS28-CRP (< 2.6)
**DAS28-ESR difference**	LSM change from baseline in the score on DAS28-ESR
**DAS28-ESR remission**	Number of patients reaching remission defined by DAS28-ESR (< 2.6)
**Deaths**	Number of patients who died during study period
**Discontinuation**	Number of patients experiencing symptoms leading to discontinuation of the study drugs
**EQ-5D (US)**	LSM change from baseline in the score on EQ-5D assessed by the US scoring algorithm

**EQ-5D (US)**	LSM change from baseline in the score on EQ-5D assessed by the UK scoring algorithm
**EQ-5D (VAS)**	LSM change from baseline in the score on EQ-5D assessed by the VAS
**ESR**	LSM change from baseline in ESR value (mm/hour)
**FACIT-F**	LSM change from baseline in the score on FACIT-F
**HAQ-DI difference**	LSM change from baseline in the score on HAQ-DI
**HAQ-DI improvement**	Number of patients reaching at least the minimum clinically important difference (≥0.22 MCID) in HAQ-DI
**MJS duration**	LSM change from baseline in the duration of morning joint stiffness
**MOS-Sleep**	LSM change from baseline in the score on MOS-Sleep
**Pain**	LSM change from baseline in pain measured on VAS ranging from 0-100 mm
**PGA**	LSM change from baseline in PGA measured on VAS ranging from 0-100 mm
**PtGA**	LSM change from baseline in PtGA measured on VAS ranging from 0-100 mm
**SDAI difference**	LSM change from baseline in the score on SDAI
**SDAI remission**	Number of patients reaching remission defined by SDAI (≤ 3.3)
**Serious side effects**	Number of patients experiencing serious side effects during the study
**SF-36 MCS**	LSM change from baseline in the score on SF-36 assessing the mental component score

**SF-36 PCS**	LSM change from baseline in the score on SF-36 assessing the physical component score
**Side effects**	Number of patients experiencing side effects during the study
**Swollen joint counts**	LSM change from baseline in the number of swollen joint counts
**Tender joint counts**	LSM change from baseline in the number of tender joint counts
**WPAI A**	LSM change from baseline in the score on WPAI assessing absenteeism (missed worked time)
**WPAI AI**	LSM change from baseline in the score on WPAI assessing activity impairment
**WPAI OWI**	LSM change from baseline in the score on WPAI assessing overall work impairment (productivity loss)
**WPAI P**	LSM change from baseline in the score on WPAI assessing presenteeism (impairment while working)

Supplementary Table S2. Summary of the investigated outcomes

ACR denotes American College of Rheumatology; SJC, swollen joint counts; TJC, tender joint counts; PtGA, Patient's Global Assessment of Disease Activity; PGA; Physician's Global Assessment of Disease Activity; CDAI, Clinical Disease Activity Index; LSM, least square mean, CRP, C-reactive protein; DAS28-CRP, Disease Activity Score 28 using C-reactive protein; DAS28-ESR, Disease Activity Score 28 using erythrocyte sedimentation rate; EQ-5D UK, EuroQol 5 Dimensions Questionnaire - UK scoring algorithm; EQ-5D US, EuroQol 5 Dimensions Questionnaire - US scoring algorithm and EQ-5d (VAS), EQ-5D measured on Visual Analogue Scale; ESR, erythrocyte sedimentation rate; FACIT-F, Functional Assessment of Chronic Illness Therapy – Fatigue; HAQ-DI, Health Assessment Questionnaire - Disability Index; MJS, Morning Joint Stiffness; MOS-Sleep, Medical Outcomes Study Sleep Scale; SDAI, Simple Disease Activity Index; SF-36 MCS, 36-Item Short Form Survey – Mental Component

Score, SF-36 PCS, SF-36 Physical Component Score ; WPAI AI, Work Productivity and Activity Impairment Questionnaire – Activity Impairment; WPAI A, WPAI - Absenteeism; WPAI - P, WPAI Presenteeism; WPAI - OWI, WPAI Overall Work Impairment;

	**JAK inhibitors vs Placebo**	**JAK inhibitors vs bDMARDS**	**JAK inhibitors vs MTX**
**ACR20 response**	yes	yes	yes
**ACR50 response**	yes	yes	yes
**ACR70 response**	yes	yes	yes
**CDAI difference**	yes	no*	no*
**CDAI remission**	yes	yes	yes
**CRP**	yes	yes	no*
**DAS28-CRP difference**	yes	no*	no*
**DAS28-CRP remission**	yes	yes	yes
**DAS28-ESR difference**	yes	no*	no*
**DAS28-ESR remission**	yes	yes	yes
**Deaths**	yes	yes	no*
**Discontinuation**	yes	no*	no*
**EQ-5D (US)**	yes	no*	no*
**EQ-5D (US)**	yes	no*	no*
**EQ-5D (VAS)**	yes	no*	no*
**ESR**	yes	no*	no*
**Erosion score**	no*	no*	no*
**FACIT-F**	yes	yes	yes
**HAQ-DI difference**	yes	yes	yes

**HAQ-DI Improvement**	yes	no*	yes
**Joint-space narrowing score**	no*	no*	no*
**MJS duration**	yes	no*	no*
**MJS severity**	no*	no*	no*
**MOS-Sleep**	yes	no*	no*
**Modified total Sharp score**	no*	no*	no*
**Pain**	yes	no*	no*
**Pain catastrophizing scale**	no*	yes	no*
**PGA**	yes	yes	no*
**PtGA**	yes	yes	no*
**SDAI difference**	yes	no*	no*
**SDAI remission**	yes	yes	yes
**Serious side effects**	yes	yes	yes
**SF-36 MCS**	yes	yes	no*
**SF-36 PCS**	yes	yes	yes
**Side effects**	yes	no*	no*
**Swollen joint counts**	yes	yes	no*
**Tender joint counts**	yes	yes	no*
**Worst Joint Pain**	no*	no*	no*
**Worst Tiredness**	no*	no*	no*
**WPAI A**	yes	no*	no*
**WPAI AI**	yes	no*	no*
**WPAI OWI**	yes	yes	no*
**WPAI P**	yes	no*	no*

Supplementary Table S3. List of the investigated outcomes in each comparisons

*Outcomes could not be included in the quantitative analysis if (1) did not appear at least in 3 of the randomized controlled trials measured in the same way or (2) about which data only at a time-point with a not acceptable study design could be obtained.

Summary of risk of bias

Unique ID	Experimental	Comparator	Weight	Randomization process	Deviations from intended interventions	Missing outcome data	Measurement of the outcome	Selection of the reported result	Overall
Greenwald 2010	baricitinib	placebo	1	?	+	+	?	?	!
I4V-MC-JADA	baricitinib	placebo	1	+	+	+	+	+	+
RA-BALANCE	baricitinib	placebo	1	+	+	+	+	+	+
RA-BEACON	baricitinib	placebo	1	+	+	+	+	+	+
RA-BEAM	baricitinib	placebo and active agent	1	+	+	+	+	+	+
RA-BEGIN	baricitinib	placebo and active agent	1	+	+	+	+	+	+
RA-BUILD	baricitinib	placebo	1	+	+	+	+	+	+
Tanaka 2016	baricitinib	placebo	1	+	+	+	+	+	+
Fleischmann 2015	decernotinib	placebo	1	?	+	+	+	+	!
Genovese 2016	decernotinib	placebo	1	?	+	?	+	+	!
Genovese 2016 II	decernotinib	placebo	1	?	+	+	+	+	!
DARWIN 1	filgotinib	placebo	1	+	+	+	+	+	+
DARWIN 2	filgotinib	placebo	1	+	+	+	+	+	+
FINCH 1	filgotinib	placebo and active agent	1	+	+	+	+	+	+
FINCH 2	filgotinib	placebo	1	+	+	+	+	+	+
FINCH 3	filgotinib	placebo and active agent	1	+	+	+	+	+	+
Vanhouette 2017 I	filgotinib	placebo	1	+	+	+	+	+	+
Vanhouette 2017 II	filgotinib	placebo	1	+	+	+	+	+	+
Luchi 2013	itacitinib	placebo	1	?	+	+	+	?	!
Genovese 2017	peficitinib	placebo	1	?	+	+	+	+	!
Kivitz 2016	peficitinib	placebo	1	?	+	+	+	+	!
RAJ 3	peficitinib	placebo	1	+	+	+	+	+	+
RAJ 4	peficitinib	placebo	1	+	+	+	+	+	+
Takeuchi 2016	peficitinib	placebo	1	+	+	+	+	+	+
Robinson 2020	rilecitinib	placebo	1	+	+	+	+	+	+
Boyle 2015	tofacitinib	Placebo	1	?	+	+	+	+	!
Conaghan 2016	tofacitinib	placebo and active agent	1	+	?	?	+	+	!
Fleischmann 2012	tofacitinib	placebo and active agent	1	?	+	+	?	+	!
Kremer 2009	tofacitinib	Placebo	1	?	+	?	+	+	!
Kremer 2012	tofacitinib	placebo	1	?	+	?	+	+	!
Menshikova 2018	tofacitinib	active agent	1	?	?	−	?	?	−
Nakamura 2018	tofacitinib	active agents	1	?	?	+	?	?	!
ORAL Scan	tofacitinib	placebo	1	+	+	+	+	+	+
ORAL Solo	tofacitinib	placebo	1	+	+	+	+	+	+
ORAL Standard	tofacitinib	placebo and active agent	1	+	+	+	+	+	+
ORAL Start	tofacitinib	active agent	1	+	+	+	+	+	+
ORAL Step	tofacitinib	placebo	1	+	+	+	+	+	+
ORAL Strategy	tofacitinib	active agent	1	+	+	+	+	+	+
ORAL Sync	tofacitinib	placebo	1	+	+	+	+	+	+
Tanaka 2011	tofacitinib	placebo	1	+	+	+	+	+	+
Tanaka 2015	tofacitinib	placebo	1	+	+	+	+	+	+
BALANCE I	upadacitinib	placebo	1	+	+	+	+	+	+
BALANCE II	upadacitinib	placebo	1	+	+	+	+	+	+
SELECT-BEYOND	upadacitinib	placebo	1	+	+	+	+	+	+
SELECT-CHOICE	upadacitinib	active agent	1	+	+	+	+	+	+
SELECT-COMPARE	upadacitinib	placebo and active agent	1	+	+	+	+	+	+
SELECT-EARLY	upadacitinib	active agent	1	+	+	+	+	+	+
SELECT-MONOTHERAPY	upadacitinib	active agent	1	+	+	+	+	+	+
SELECT-NEXT	upadacitinib	placebo	1	+	+	+	+	+	+
SELECT-SUNRISE	upadacitinib	placebo	1	+	+	+	+	+	+

Supplementary Table S4. Summary of the overall risk of bias in the included studies

Summary of results

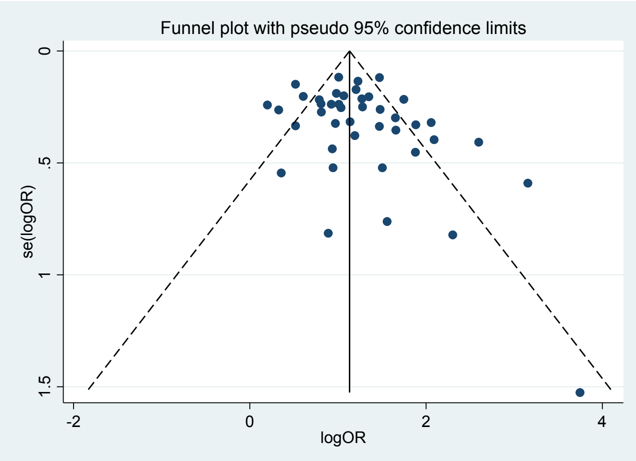

S1. Funnel plot of ACR 20 response (JAK inhibitor vs placebo)

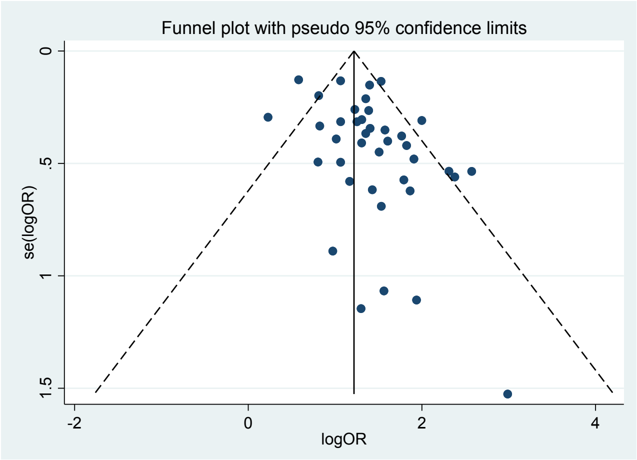

S2. Funnel plot of ACR 50 response (JAK inhibitor vs placebo)

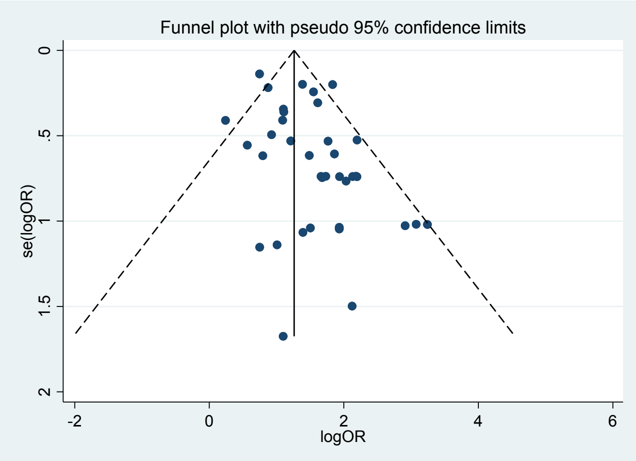

S3. Funnel plot of ACR 70 response (JAK inhibitor vs placebo)

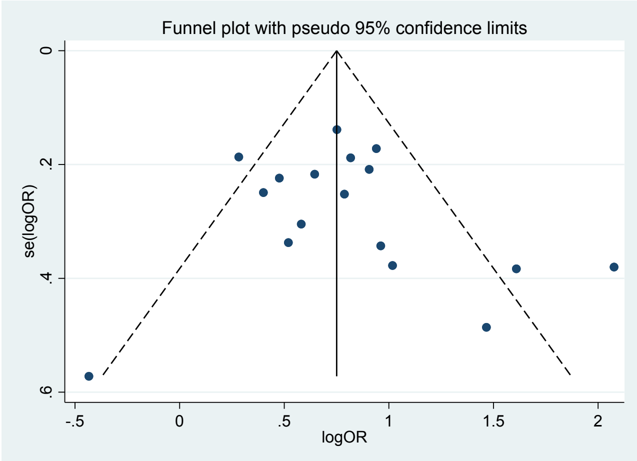

S4. Funnel plot of HAQ-DI improvement (JAK inhibitor vs placebo)

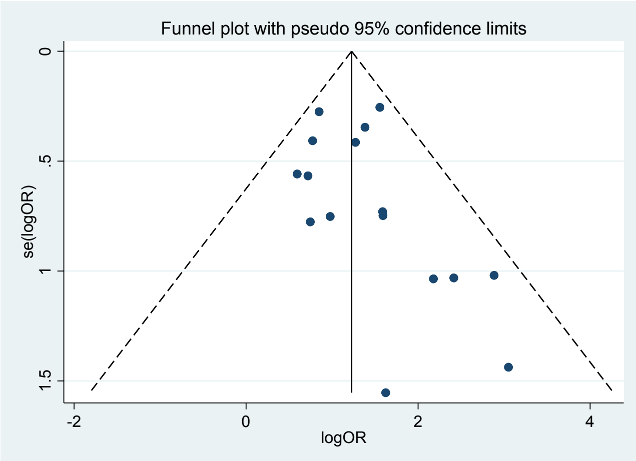

S5. Funnel plot of CDAI remission (JAK inhibitor vs placebo)

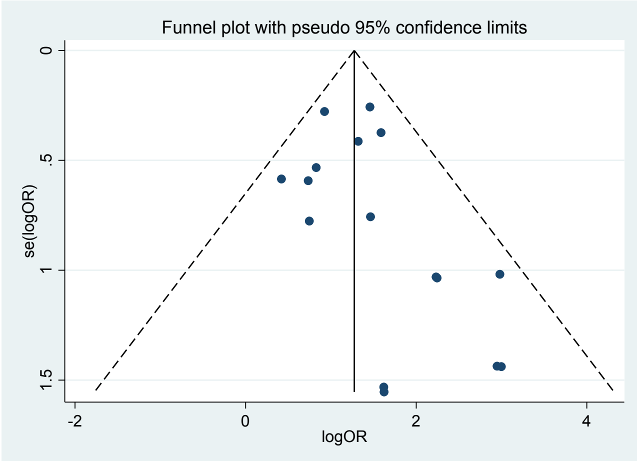

S6. Funnel plot of SDAI remission (JAK inhibitor vs placebo)

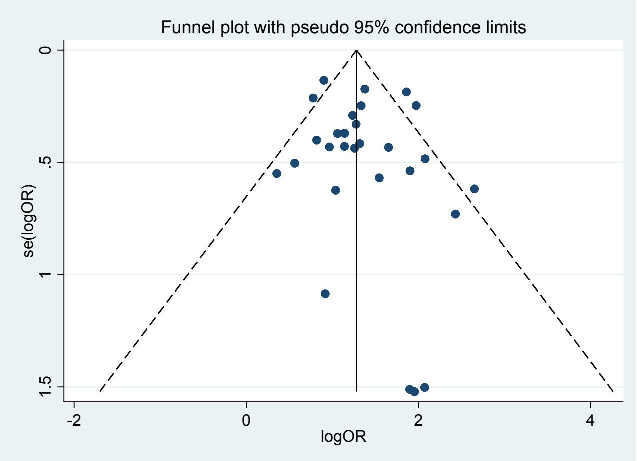

S7. Funnel plot of DAS28-CRP remission (JAK inhibitor vs placebo)

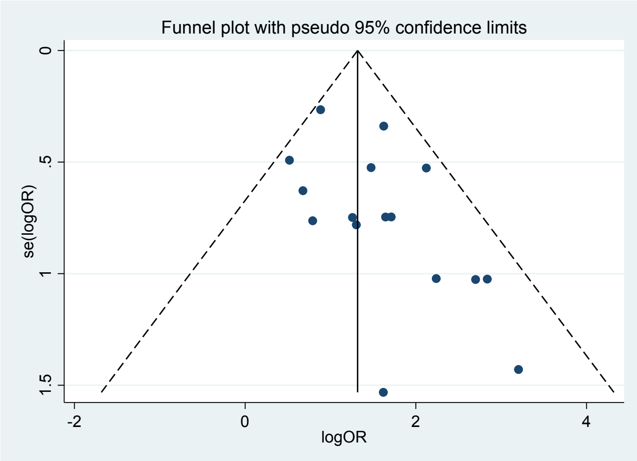

S8. Funnel plot of DAS28-ESR remission (JAK inhibitor vs placebo)

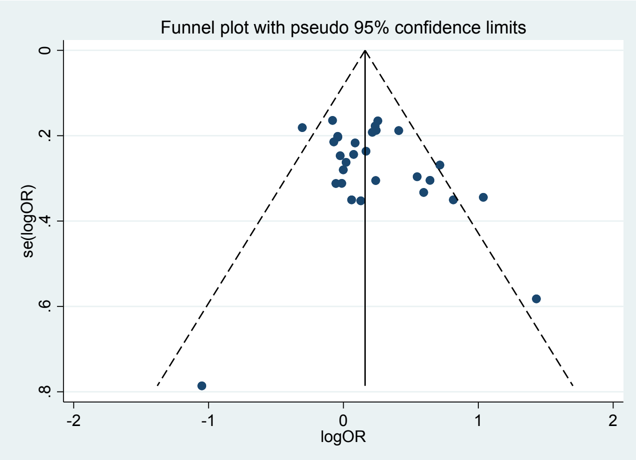

S9. Funnel plot of side effects remission (JAK inhibitor vs placebo)

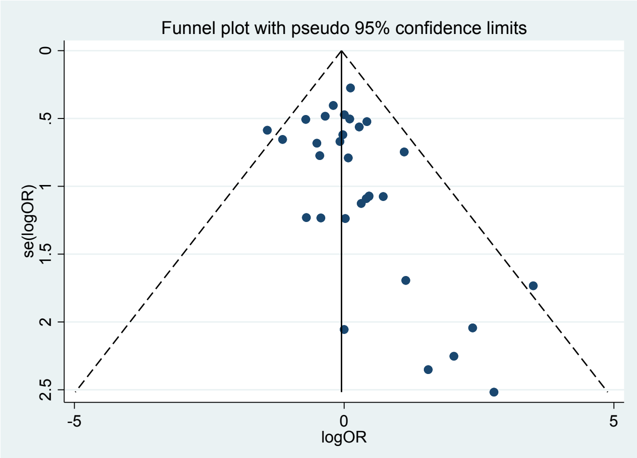

S10. Funnel plot of serious side effects remission (JAK inhibitor vs placebo)

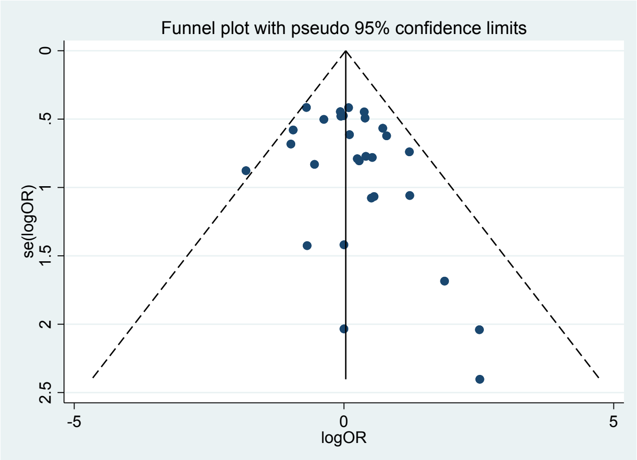

S11. Funnel plot of discontinuation (JAK inhibitor vs placebo)

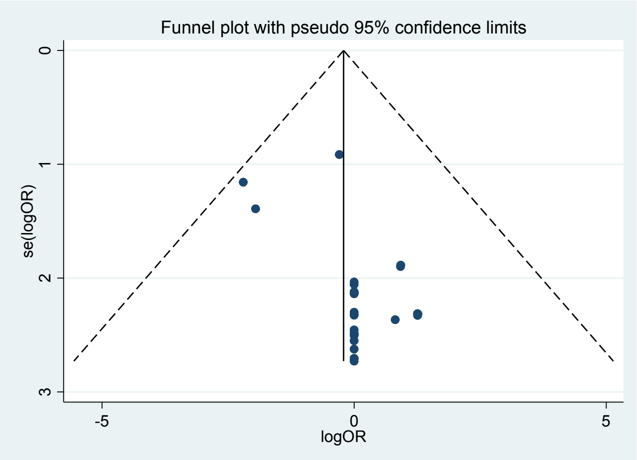

S12. Funnel plot of deaths (JAK inhibitor vs placebo)

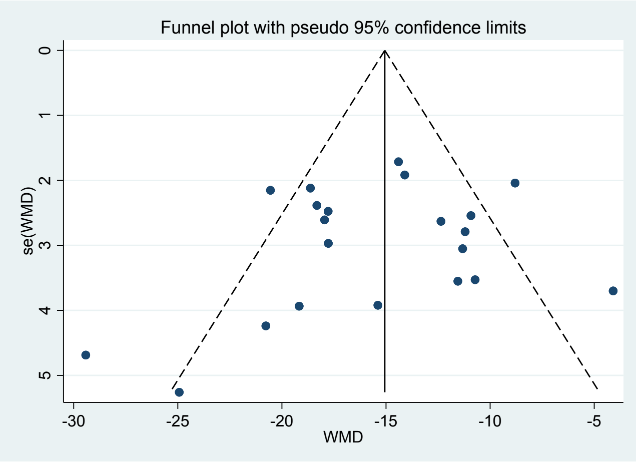

S13. Funnel plot of Pain VAS (JAK inhibitor vs placebo)

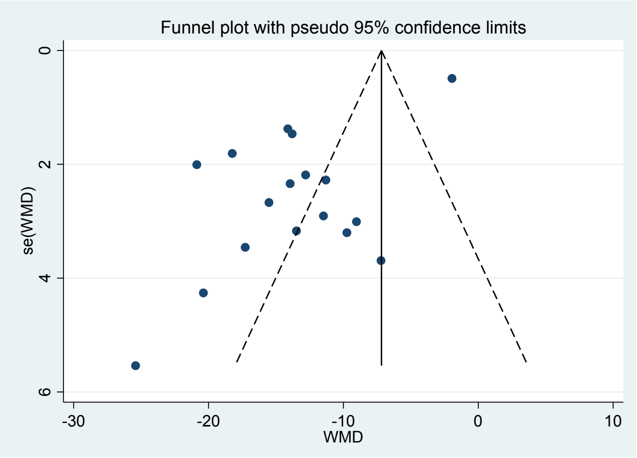

S14. Funnel plot of PGA VAS (JAK inhibitor vs placebo)

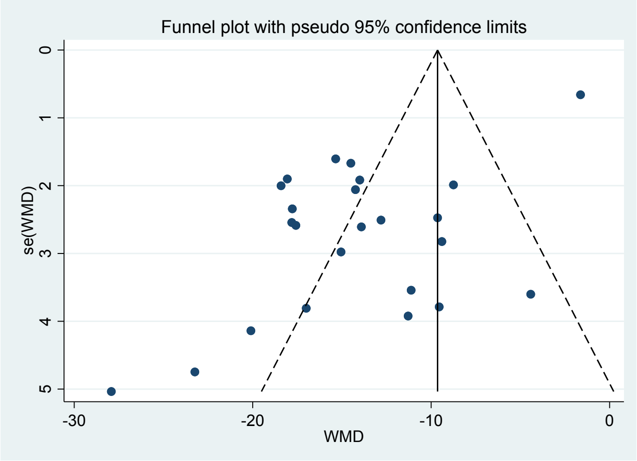

S15. Funnel plot of PtGA VAS (JAK inhibitor vs placebo)

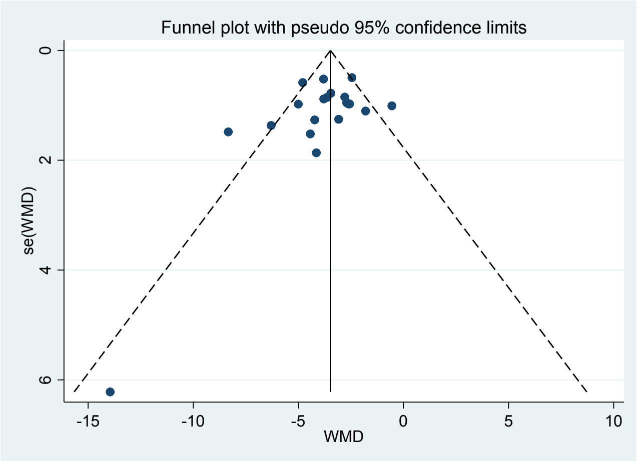

S16. Funnel plot of Swollen Joint Count (JAK inhibitor vs placebo)

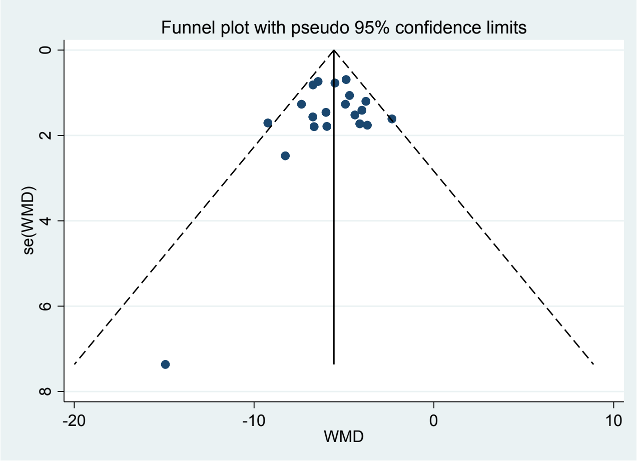

S17. Funnel plot of Tender Joint Count (JAK inhibitor vs placebo)

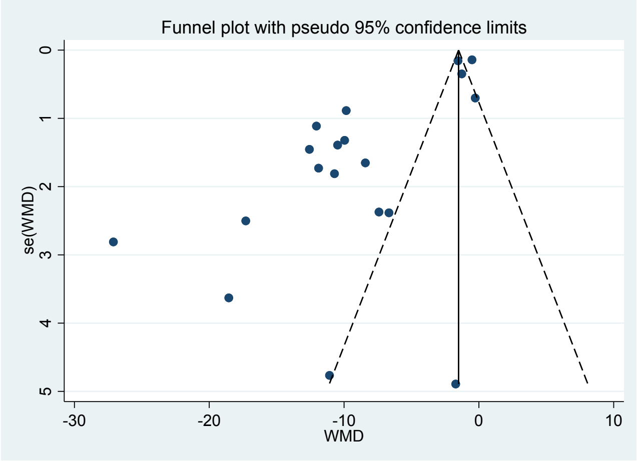

S18. Funnel plot of CRP (JAK inhibitor vs placebo)

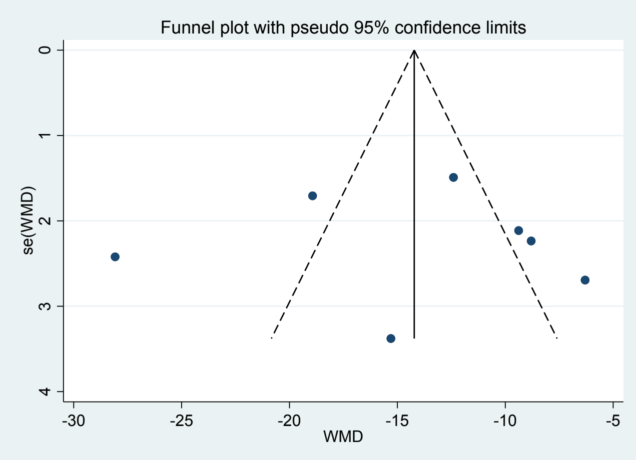

S19. Funnel plot of ESR (JAK inhibitor vs placebo)

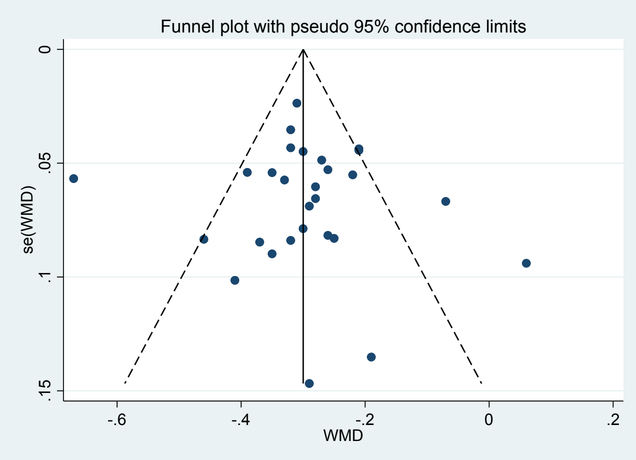

S20. Funnel plot of HAQ-DI improvement (JAK inhibitor vs placebo)

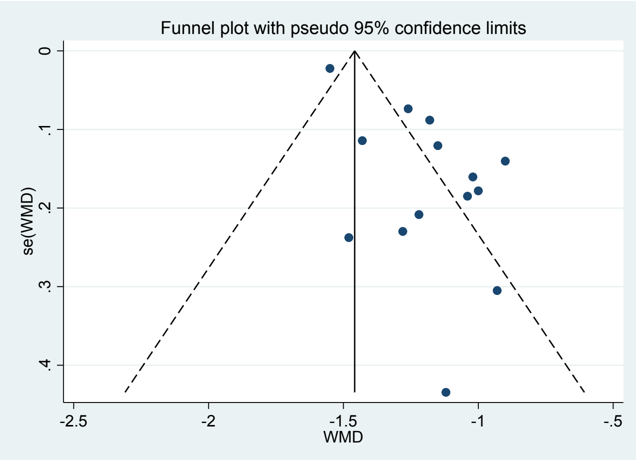

S21. Funnel plot of DAS28-CRP difference (JAK inhibitor vs placebo)

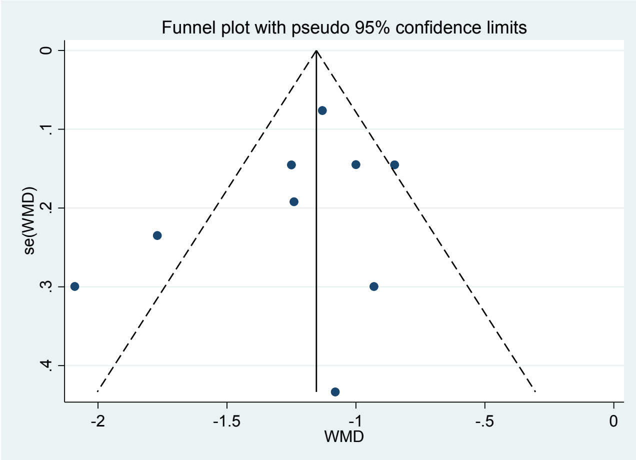

S22. Funnel plot of DAS28-ESR difference (JAK inhibitor vs placebo)

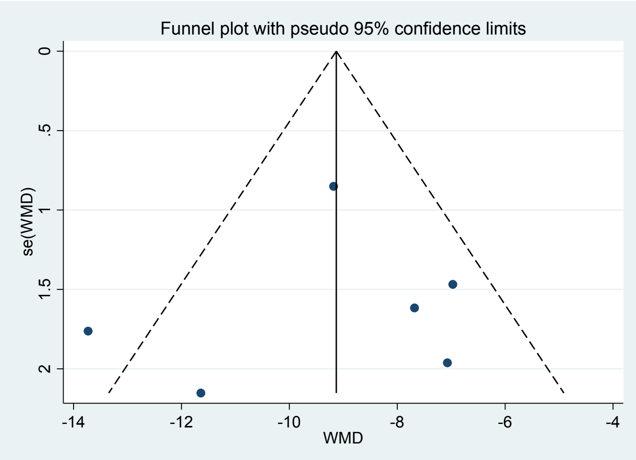

S23. Funnel plot of CDAI difference (JAK inhibitor vs placebo)

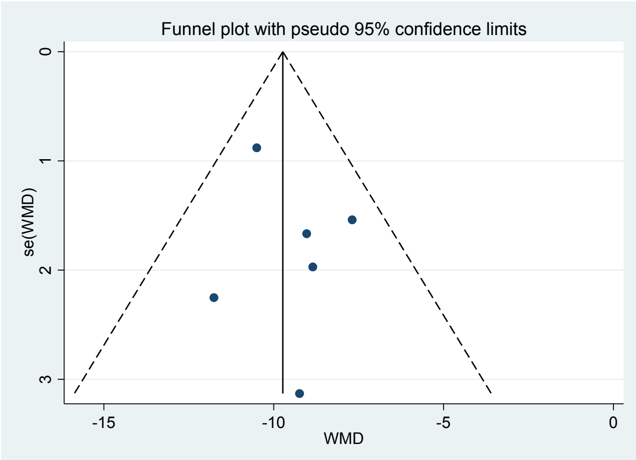

S24. Funnel plot of SDAI difference (JAK inhibitor vs placebo)

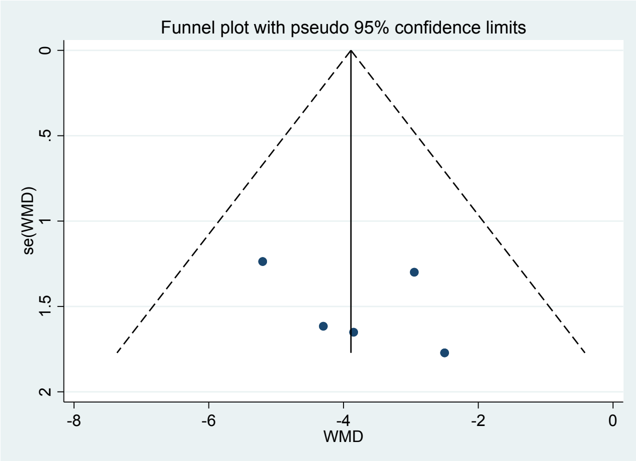

S25. Funnel plot of MOS-Sleep (JAK inhibitor vs placebo)

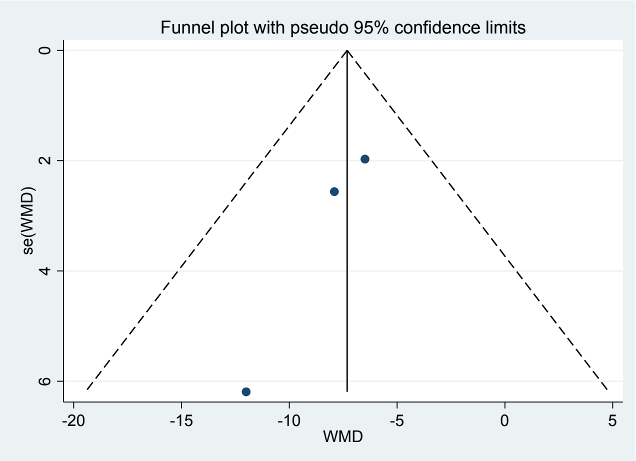

S26. Funnel plot of WPAI AI (JAK inhibitor vs placebo)

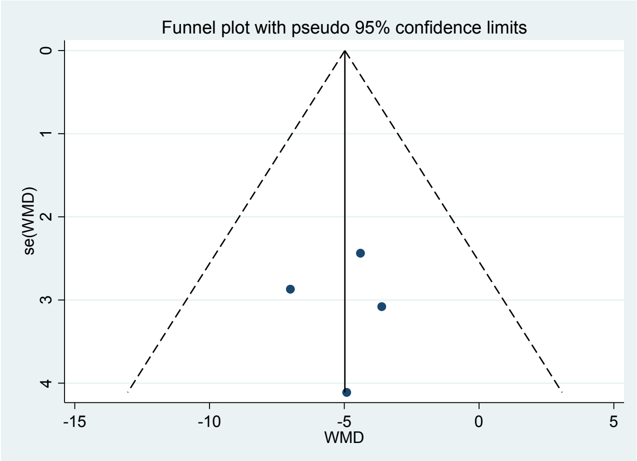

S27. Funnel plot of WPAI A (JAK inhibitor vs placebo)

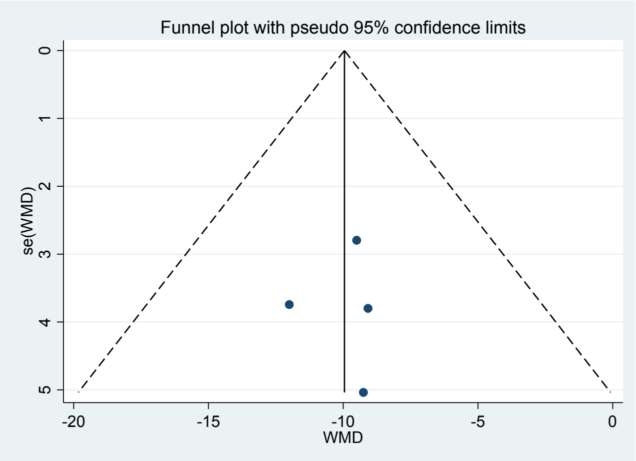

S28. Funnel plot of WPAI OWI (JAK inhibitor vs placebo)

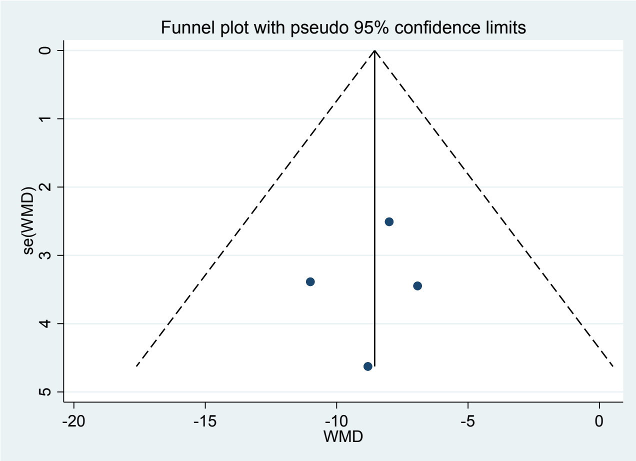

S29. Funnel plot of WPAI P (JAK inhibitor vs placebo)

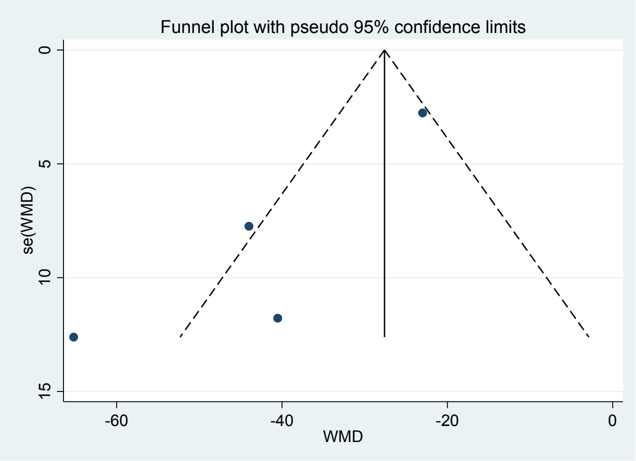

S30. Funnel plot of MJS duration (JAK inhibitor vs placebo)

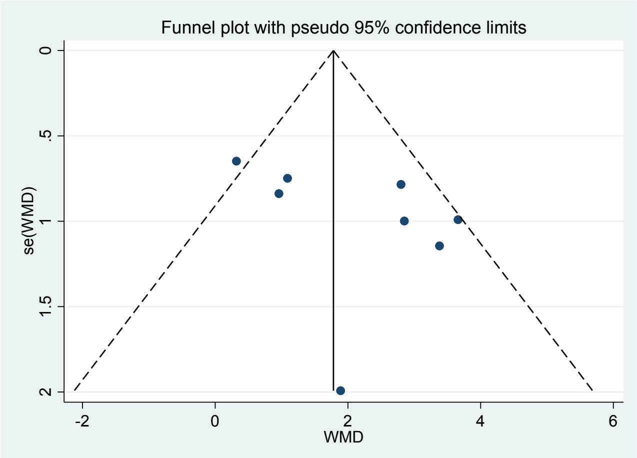

S31. Funnel plot of SF-36 MCS (JAK inhibitor vs placebo)

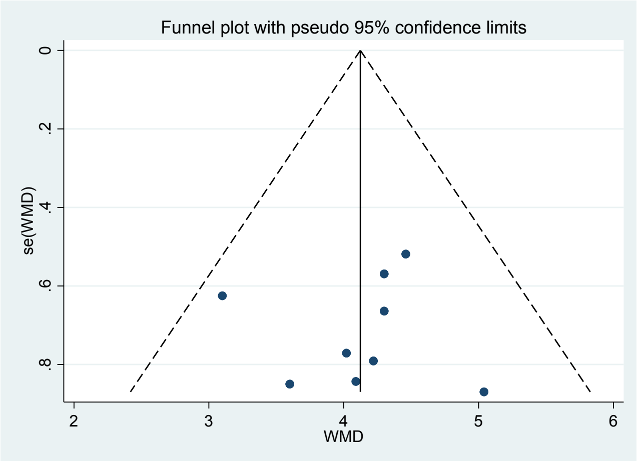

S32. Funnel plot of SF-36 PCS (JAK inhibitor vs placebo)

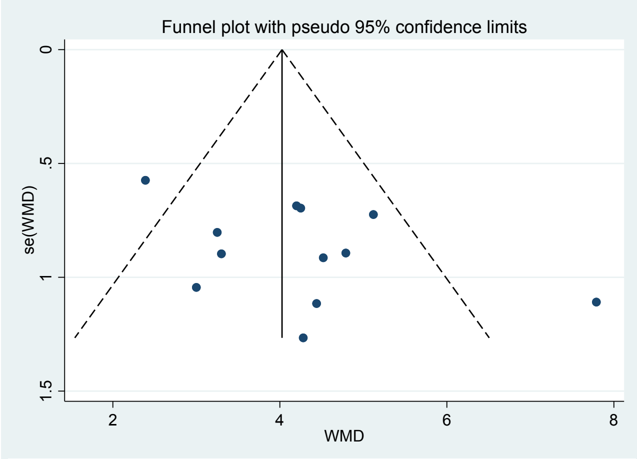

S33. Funnel plot of FACIT-F (JAK inhibitor vs placebo)

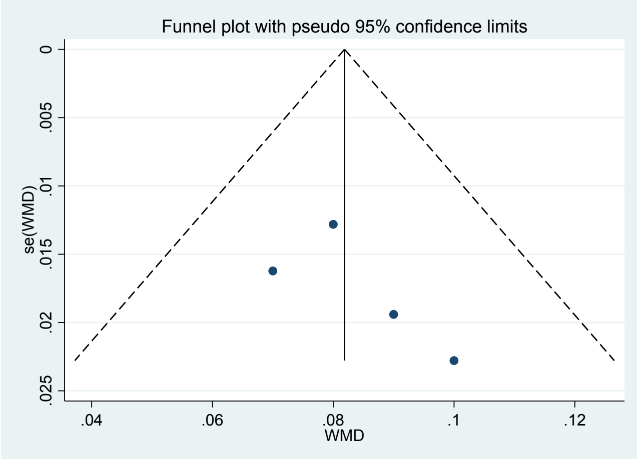

S34. Funnel plot of EQ-5D UK (JAK inhibitor vs placebo)

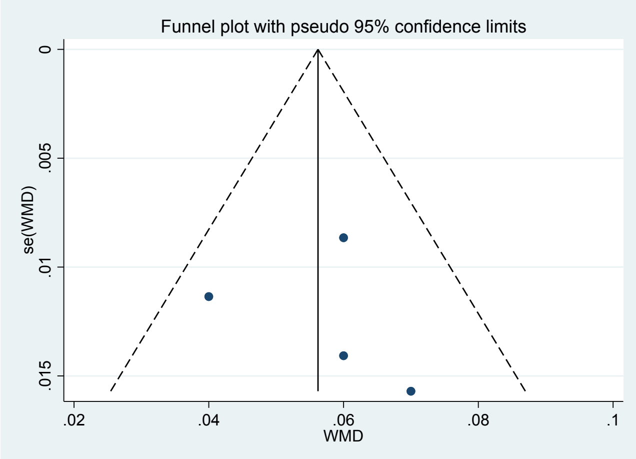

S35. Funnel plot of EQ-5D US (JAK inhibitor vs placebo)

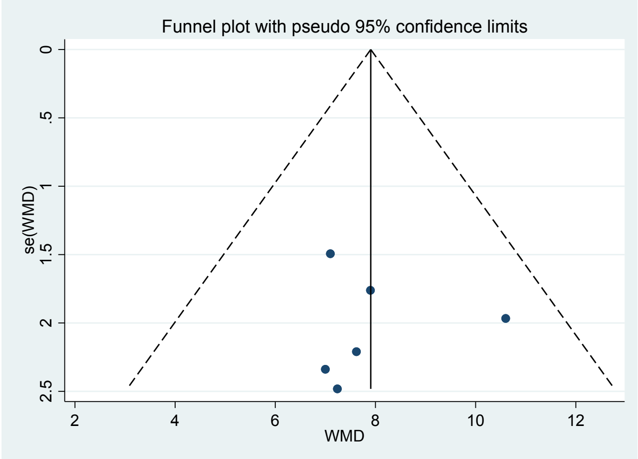

S36. Funnel plot of EQ-5D VAS (JAK inhibitor vs placebo)

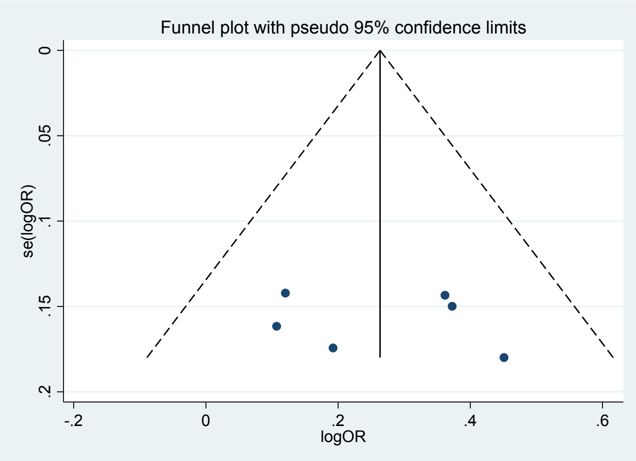

S37. Funnel plot of ACR20 response (JAK inhibitor vs bDMARDs)

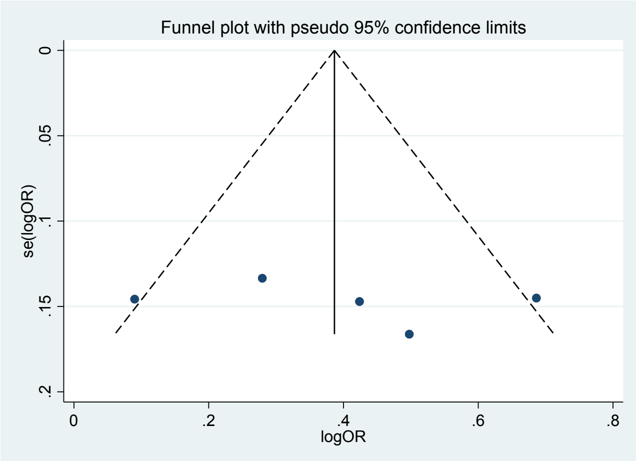

S38. Funnel plot of ACR50 response (JAK inhibitor vs bDMARDs)

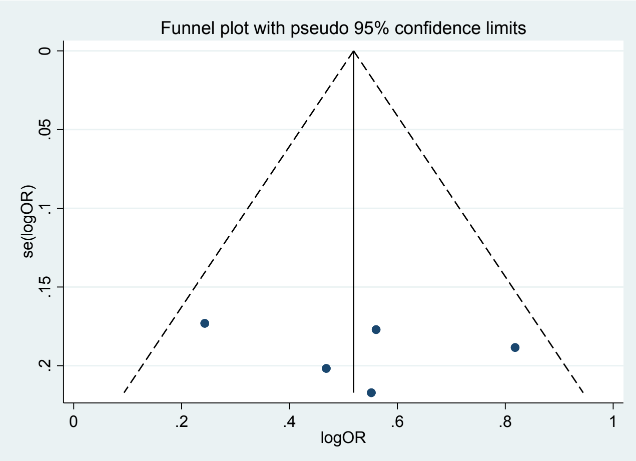

S39. Funnel plot of ACR70 response (JAK inhibitor vs bDMARDs)

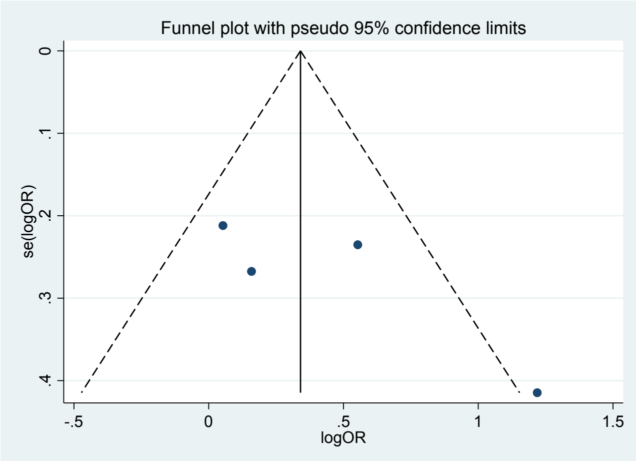

S40. Funnel plot of CDAI remission (JAK inhibitor vs bDMARDs)

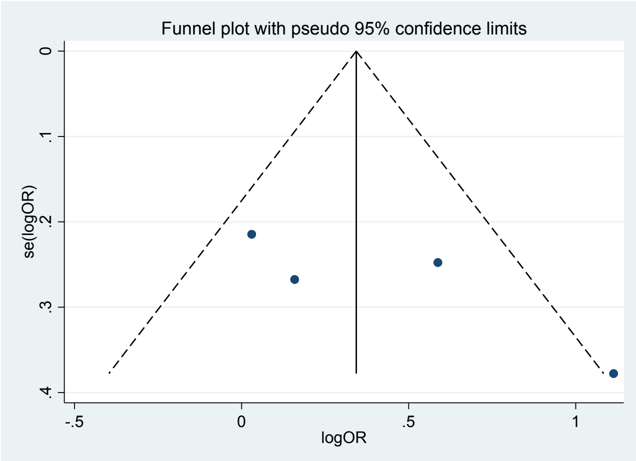

S41. Funnel plot of SDAI remission (JAK inhibitor vs bDMARDs)

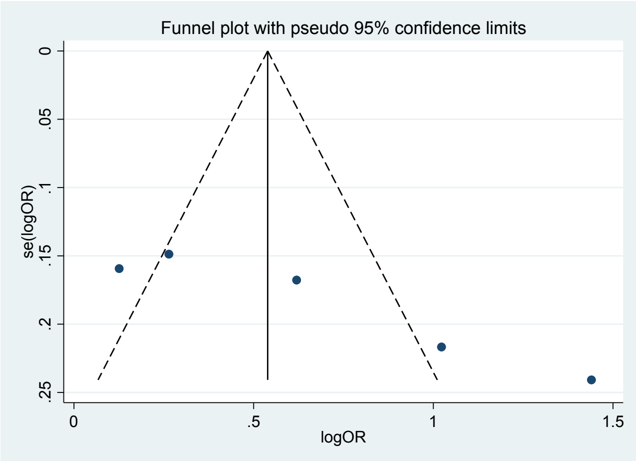

S42. Funnel plot of DAS28 - CRP remission (JAK inhibitor vs bDMARDs)

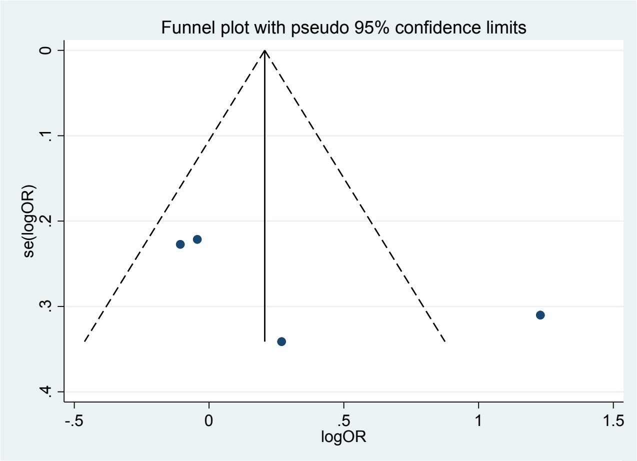

S43. Funnel plot of DAS28 - ESR remission (JAK inhibitor vs bDMARDs)

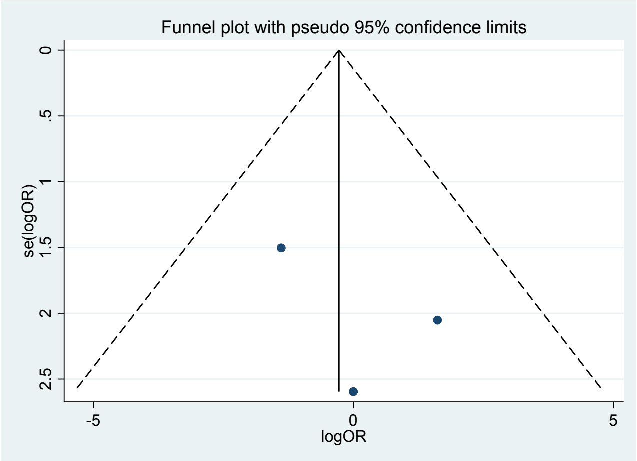

S44. Funnel plot of deaths (JAK inhibitor vs bDMARDs)

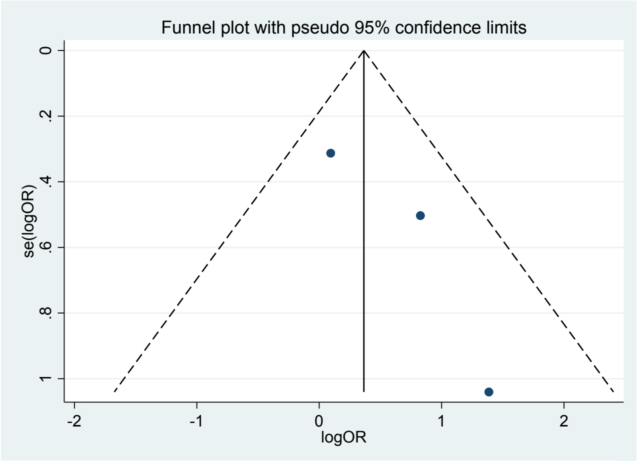

S45. Funnel plot of serious side effects (JAK inhibitor vs bDMARDs)

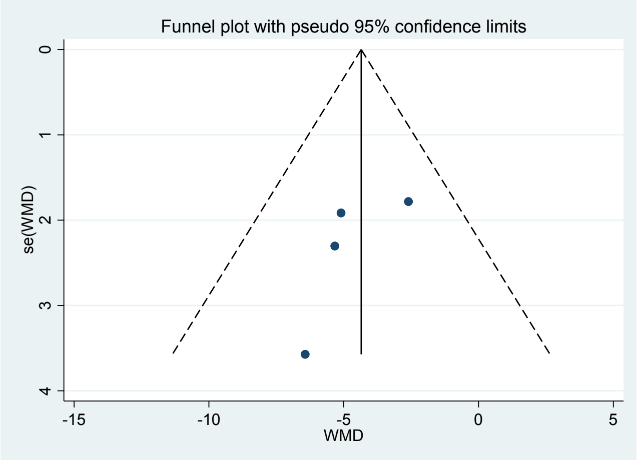

S46. Funnel plot of Pain VAS (JAK inhibitor vs bDMARDs)

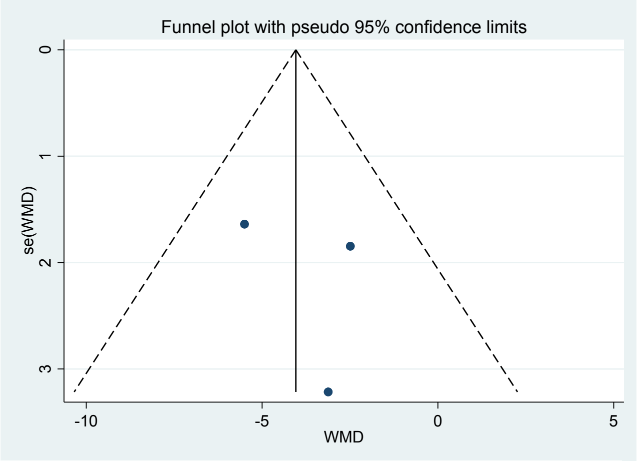

S47. Funnel plot of PGA VAS (JAK inhibitor vs bDMARDs)

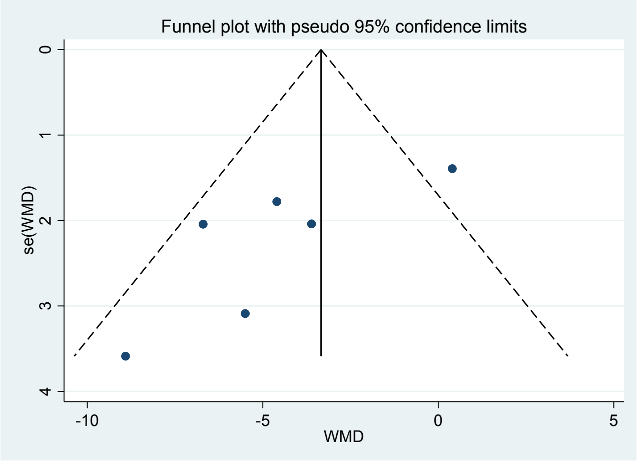

S48. Funnel plot of PtGA VAS (JAK inhibitor vs bDMARDs)

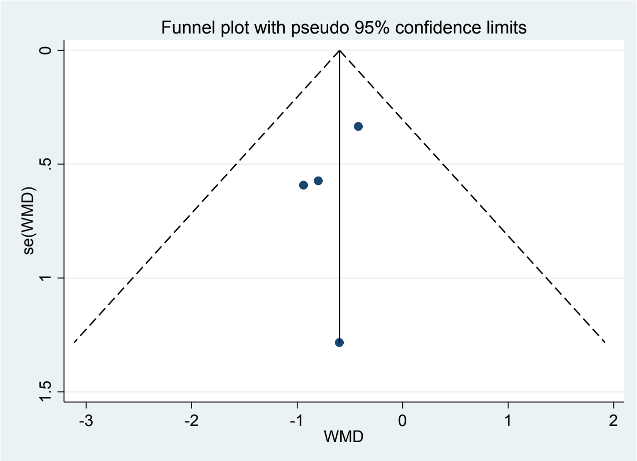

S49. Funnel plot of Swollen Joint Count (JAK inhibitor vs bDMARDs)

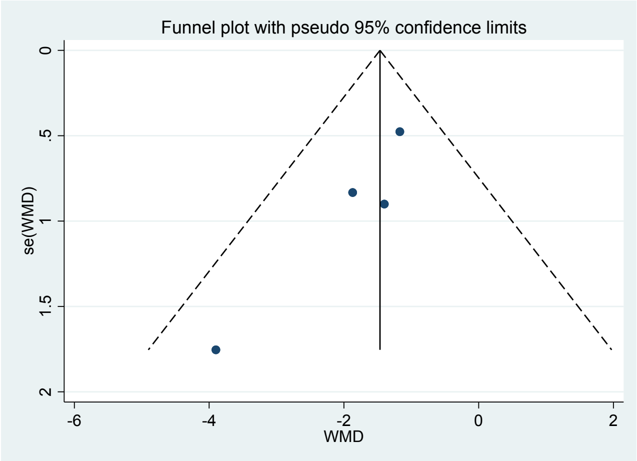

S50. Funnel plot of Tender Joint Count (JAK inhibitor vs bDMARDs)

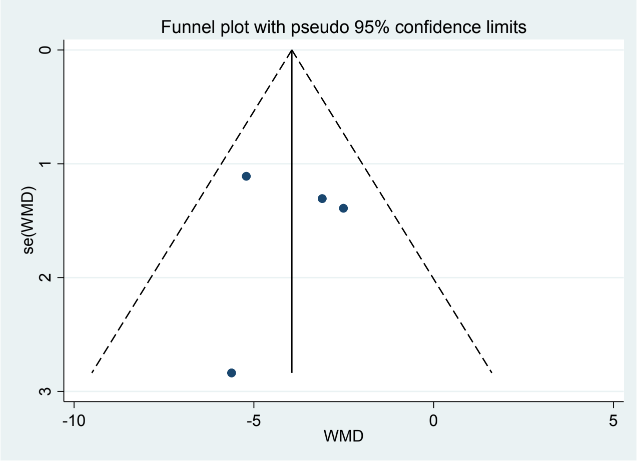

S51. Funnel plot of CRP (JAK inhibitor vs bDMARDs)

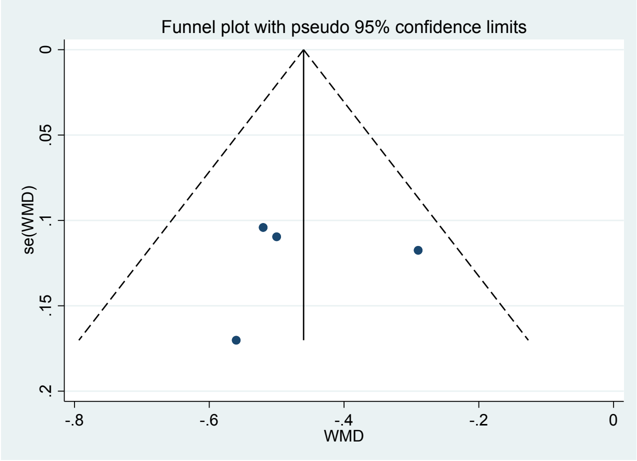

S52. Funnel plot of DAS28 - CRP difference (JAK inhibitor vs bDMARDs)

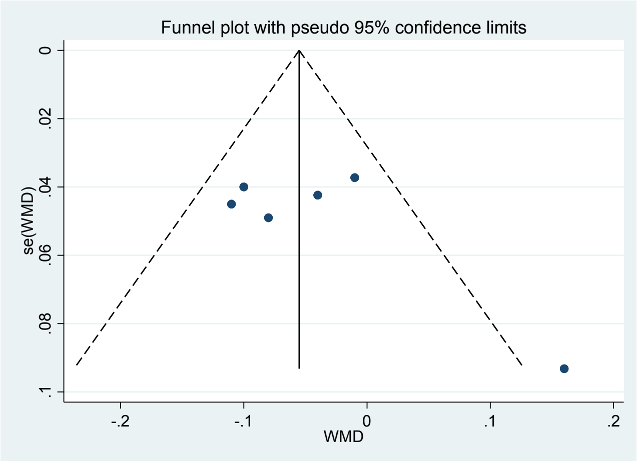

S53. Funnel plot of HAQ-DI difference (JAK inhibitor vs bDMARDs)

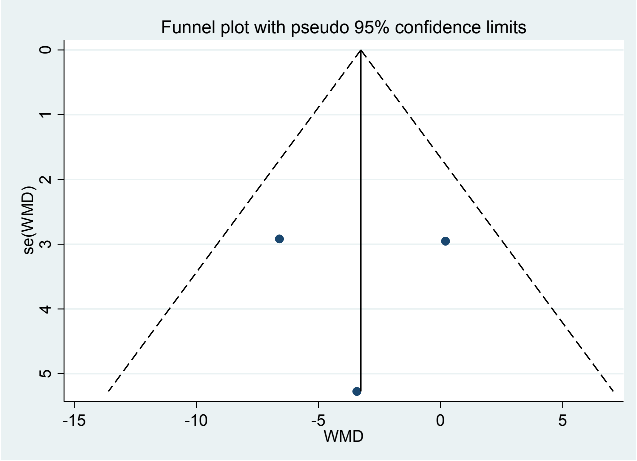

S54. Funnel plot of WPAI OWI (JAK inhibitor vs bDMARDs)

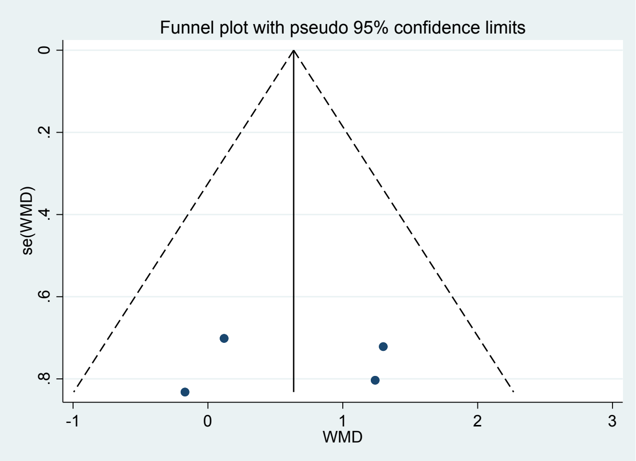

S55. Funnel plot of SF-36 MCS (JAK inhibitor vs bDMARDs)

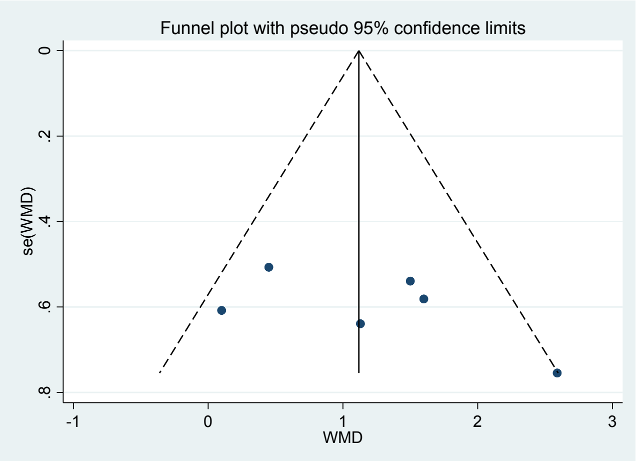

S56. Funnel plot of SF-36 PCS (JAK inhibitor vs bDMARDs)

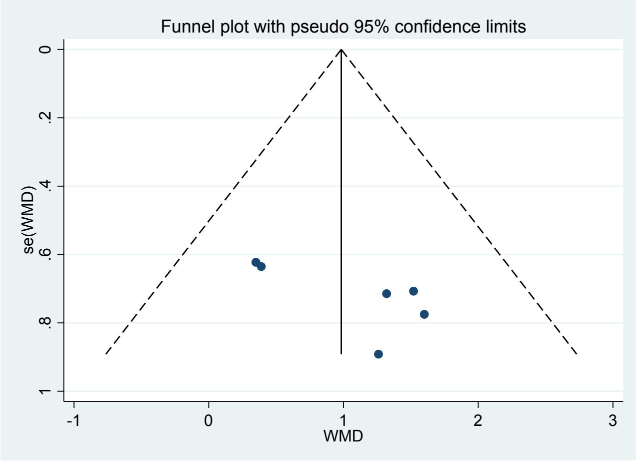

S57. Funnel plot of FACIT-F (JAK inhibitor vs bDMARDs)

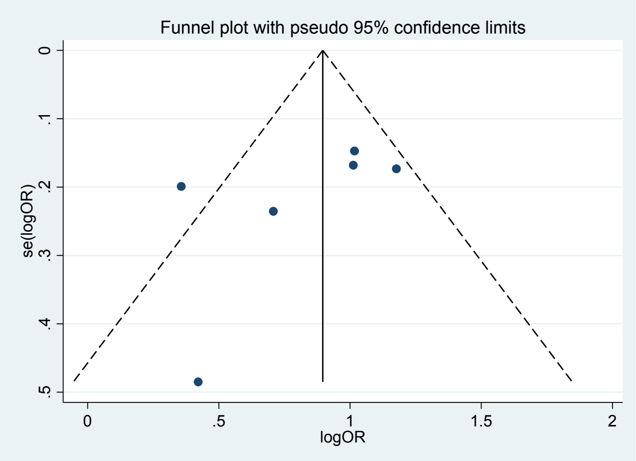

S58. Funnel plot of ACR20 response (JAK inhibitor vs MTX)

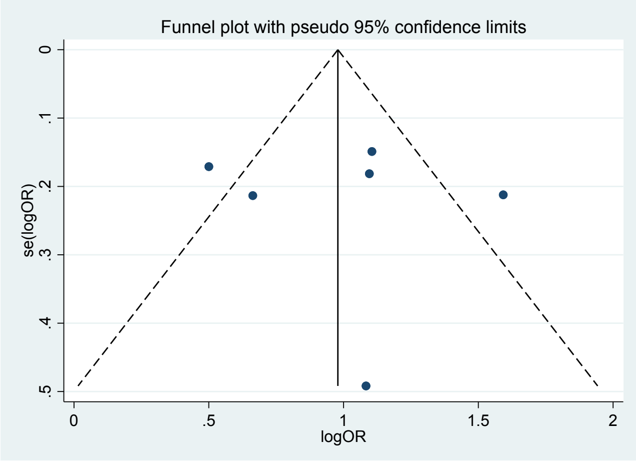

S59. Funnel plot of ACR50 response (JAK inhibitor vs MTX)

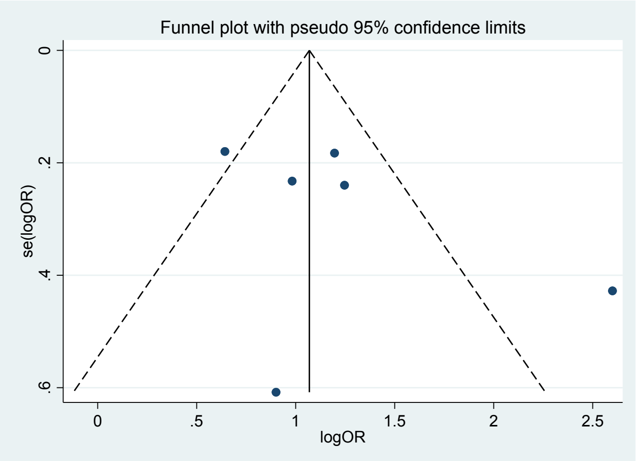

S60. Funnel plot of ACR70 response (JAK inhibitor vs MTX)

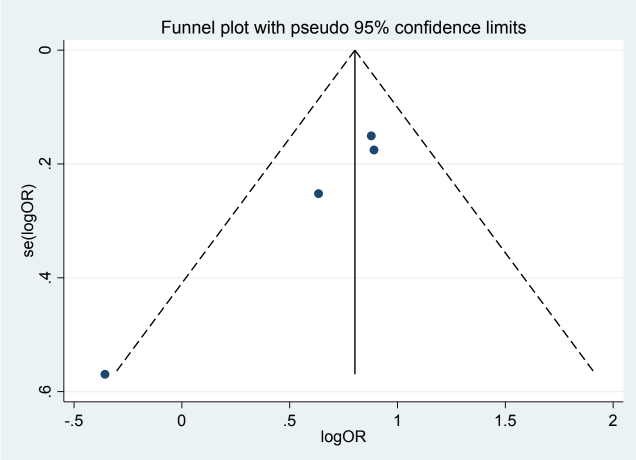

S61. Funnel plot of HAQ - DI improvement (JAK inhibitor vs MTX)

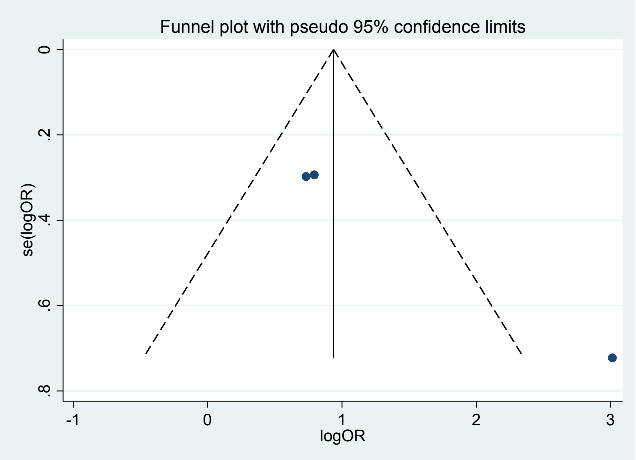

S62. Funnel plot of CDAI remission (JAK inhibitor vs MTX)

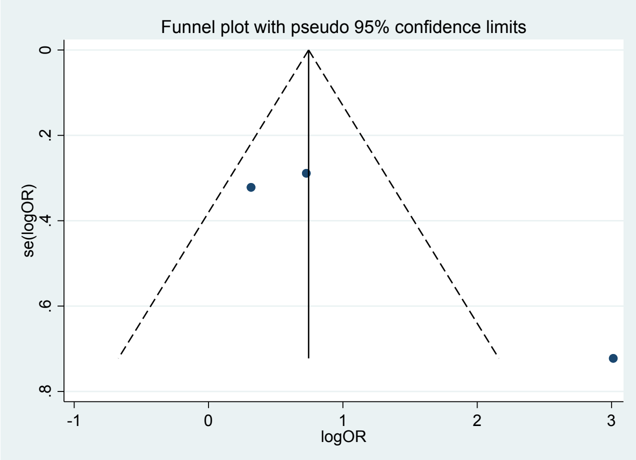

S63. Funnel plot of SDAI remission (JAK inhibitor vs MTX)

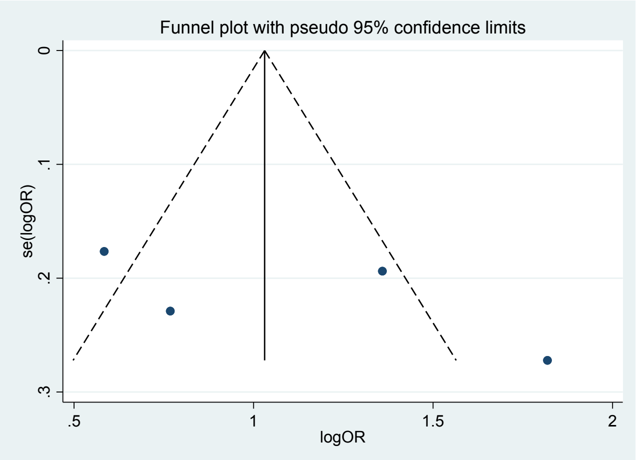

S64. Funnel plot of DAS28 – CRP remission (JAK inhibitor vs MTX)

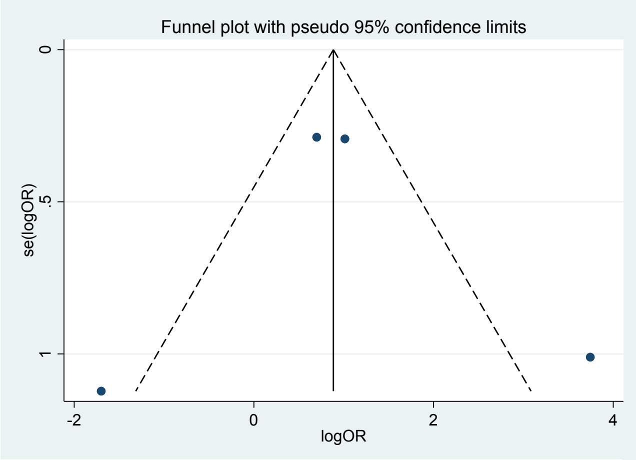

S65. Funnel plot of DAS28 – ESR remission (JAK inhibitor vs MTX)

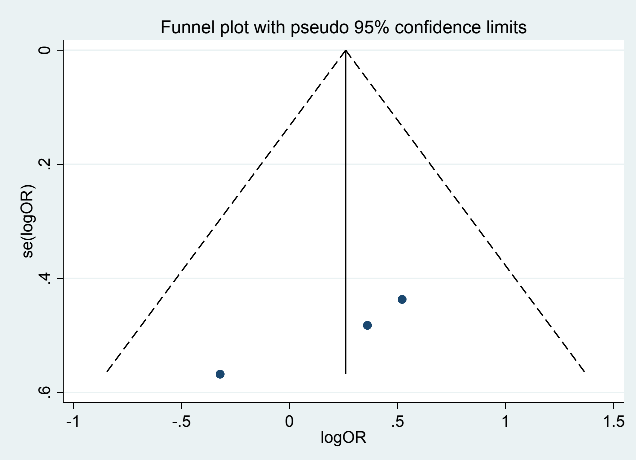

S66. Funnel plot of serious side effects (JAK inhibitor vs MTX)

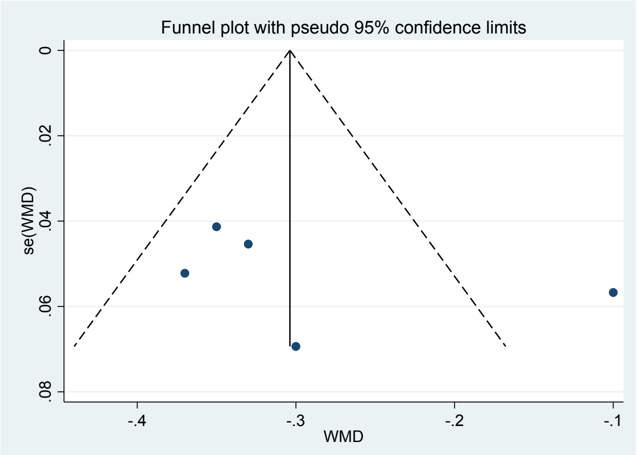

S67. Funnel plot of HAQ-DI difference (JAK inhibitor vs MTX)

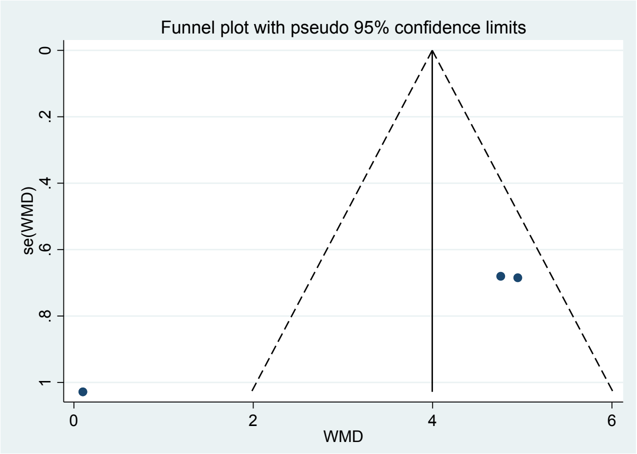

S68. Funnel plot of SF-36 PCS (JAK inhibitor vs MTX)

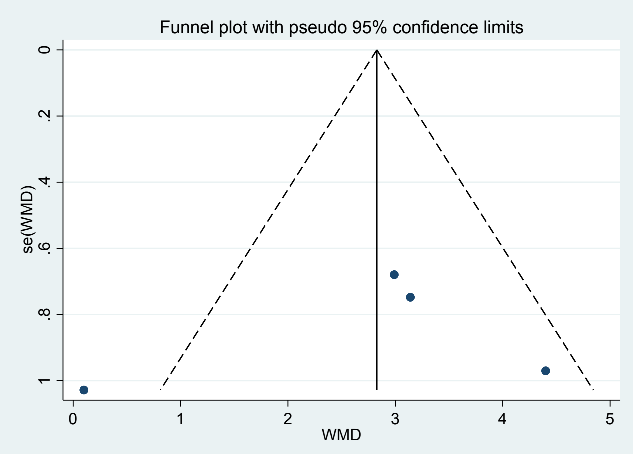

S69. Funnel plot of FACIT - F (JAK inhibitor vs MTX)

(1) <u>JAK inhibitors compared to placebo</u>

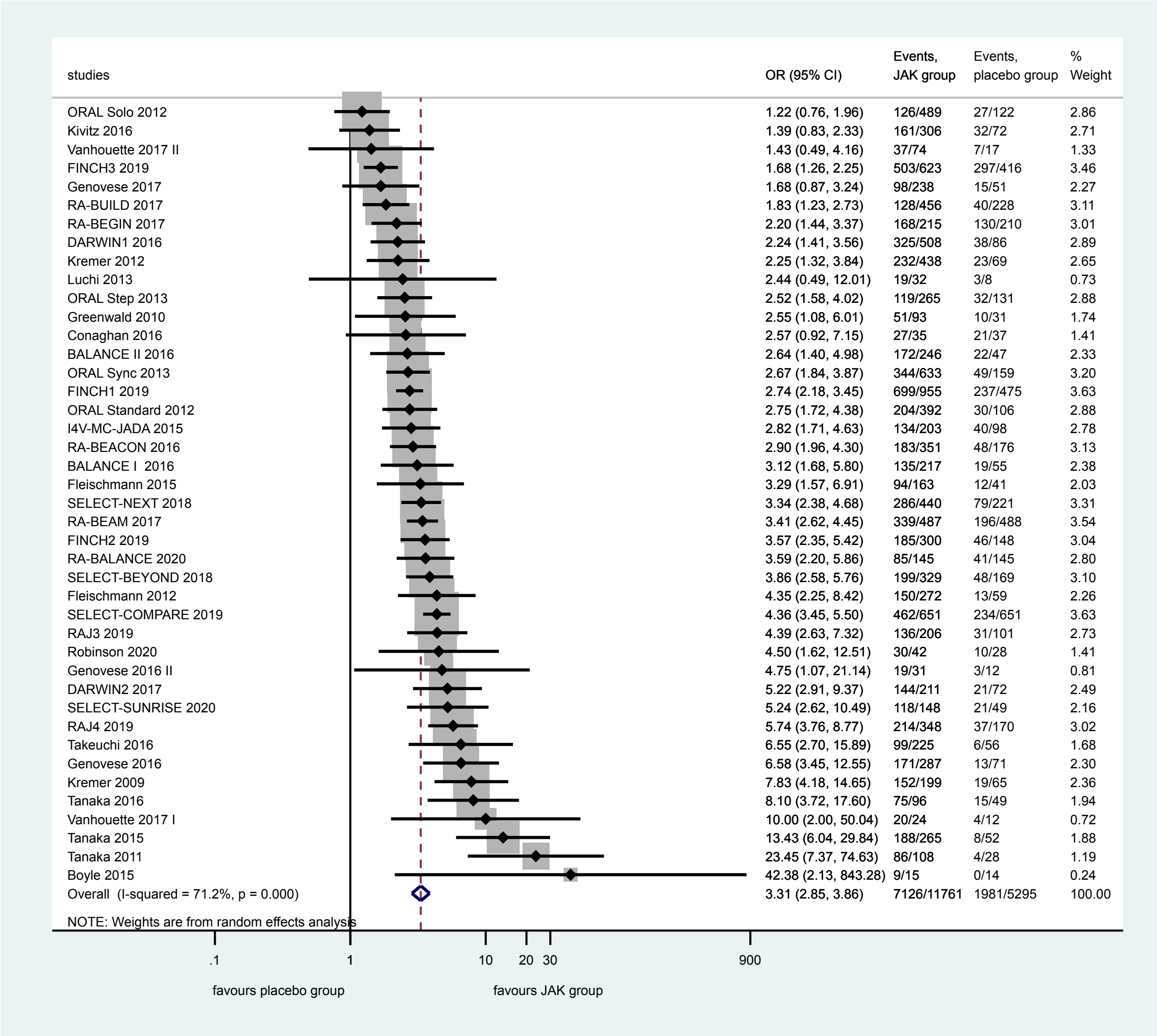

S70. Forest plot of studies comparing the number of patients reaching at least 20% in ACR criteria between patients treated with JAK inhibitors and placebo within 6 months (ACR20 response)

Black diamonds exhibit the individual effect of each study, whereas outer lines show the confidence intervals (CI) and the size of grey squares are proportional to the weight of each study. The blue diamond demonstrates the overall effect, the edges of which represent the CIs.

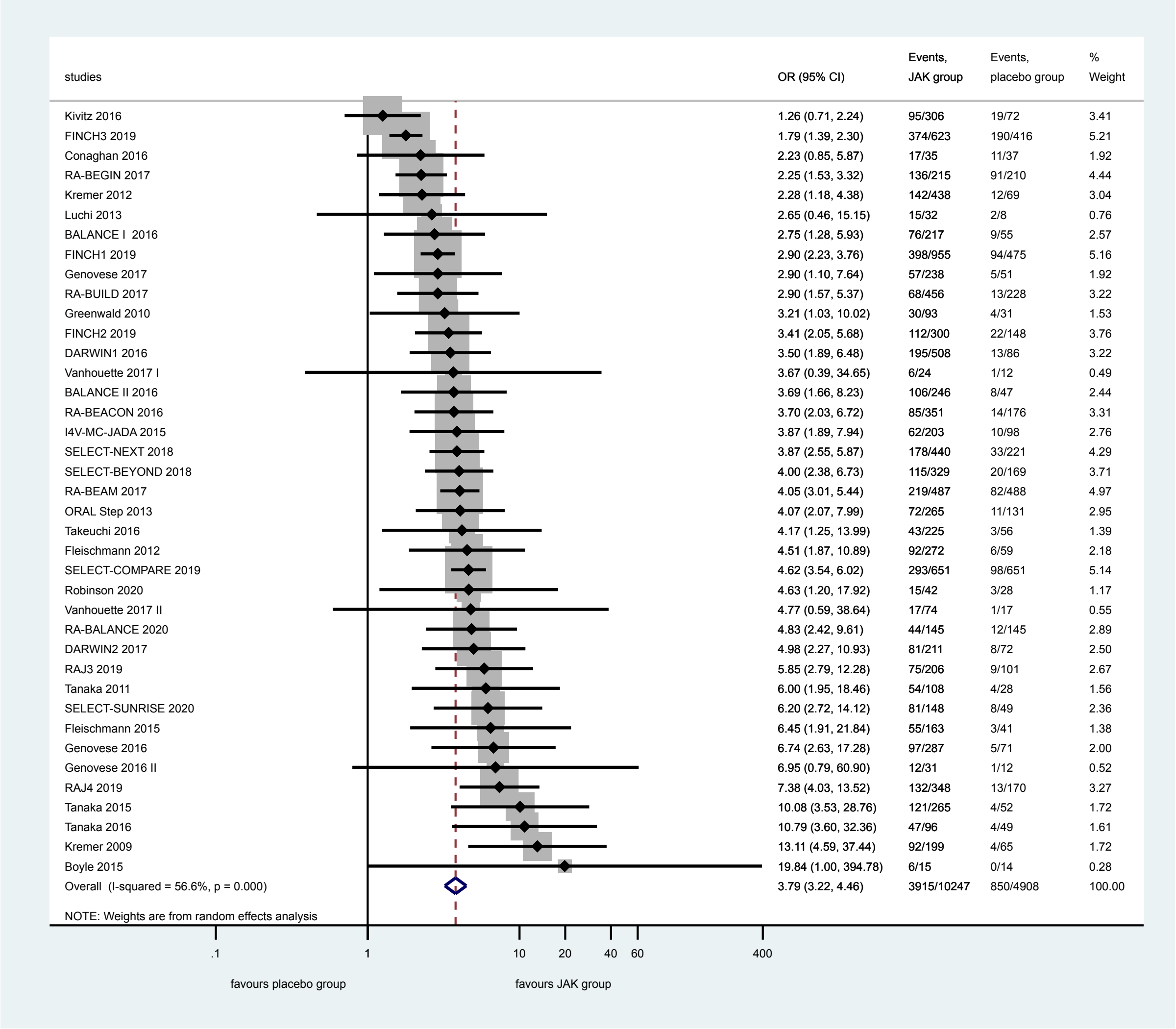

S71. Forest plot of studies comparing the number of patients reaching at least 50% in ACR criteria between patients treated with JAK inhibitors and placebo within 6 months (ACR50 response)

Black diamonds exhibit the individual effect of each study, whereas outer lines show the confidence intervals (CI) and the size of grey squares are proportional to the weight of each study. The blue diamond demonstrates the overall effect, the edges of which represent the CIs.

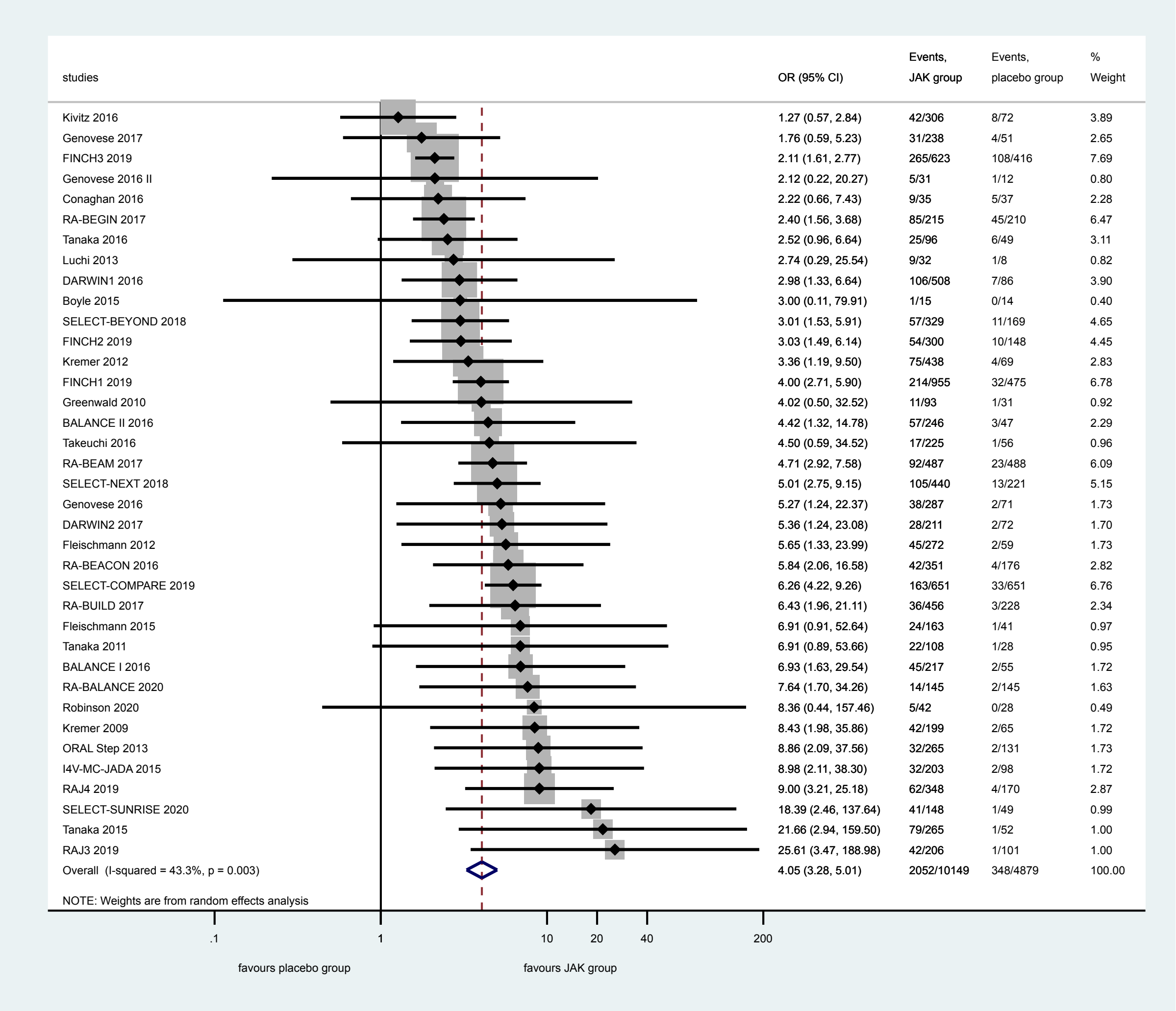

S72. Forest plot of studies comparing the number of patients reaching at least 70% in ACR criteria between patients treated with JAK inhibitors and placebo within 6 months (ACR70 response)

Black diamonds exhibit the individual effect of each study, whereas outer lines show the confidence intervals (CI) and the size of grey squares are proportional to the weight of each study. The blue diamond demonstrates the overall effect, the edges of which represent the CIs.

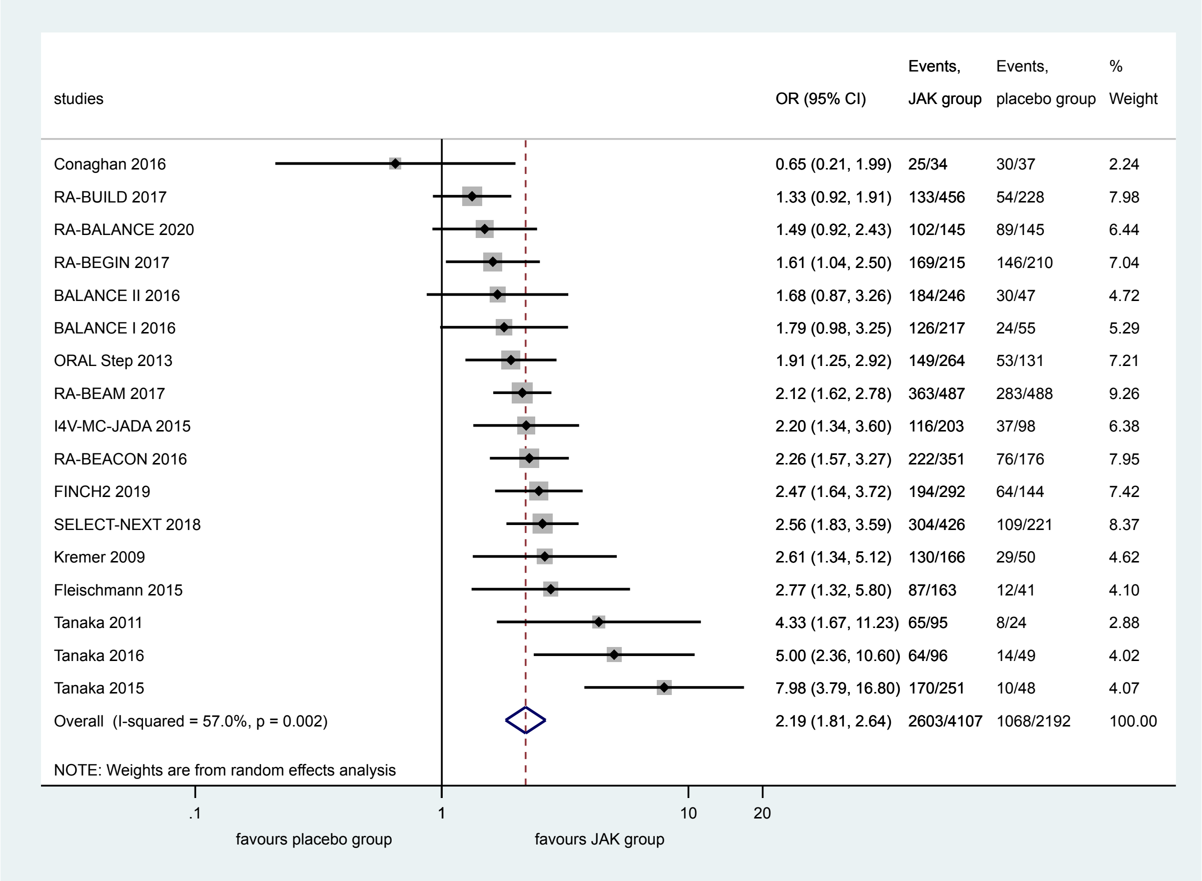

S73. Forest plot of studies comparing the number of patients reaching at least the minimum clinically important difference (≥0.22 MCID) in HAQ-DI between patients treated with JAK inhibitors and placebo within 6 months (HAQ-DI improvement)

Black diamonds exhibit the individual effect of each study, whereas outer lines show the confidence intervals (CI) and the size of grey squares are proportional to the weight of each study. The blue diamond demonstrates the overall effect, the edges of which represent the CIs.

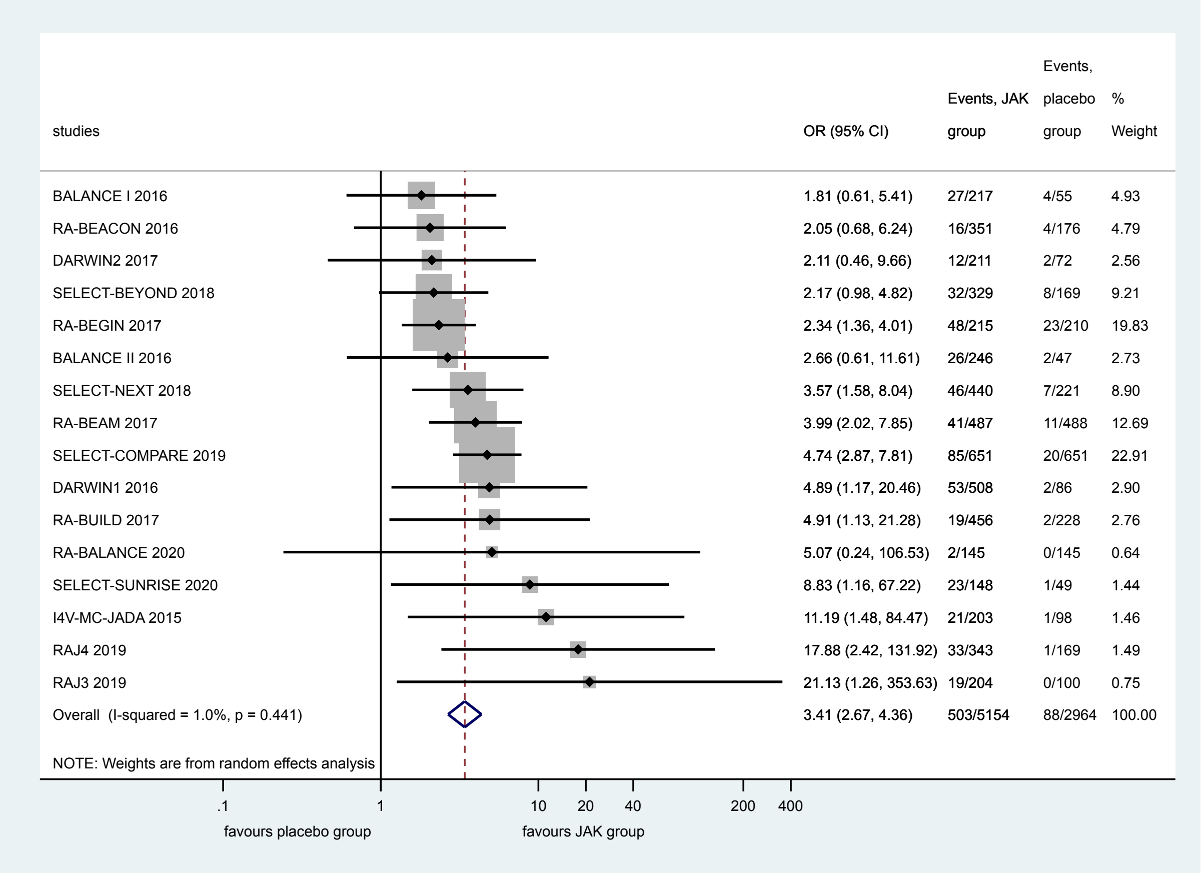

S74. Forest plot of studies comparing the number of patients reaching remission defined by CDAI (≤ 2.8) between patients treated with JAK inhibitors and placebo within 6 months (CDAI remission)

Black diamonds exhibit the individual effect of each study, whereas outer lines show the confidence intervals (CI) and the size of grey squares are proportional to the weight of each study. The blue diamond demonstrates the overall effect, the edges of which represent the CIs.

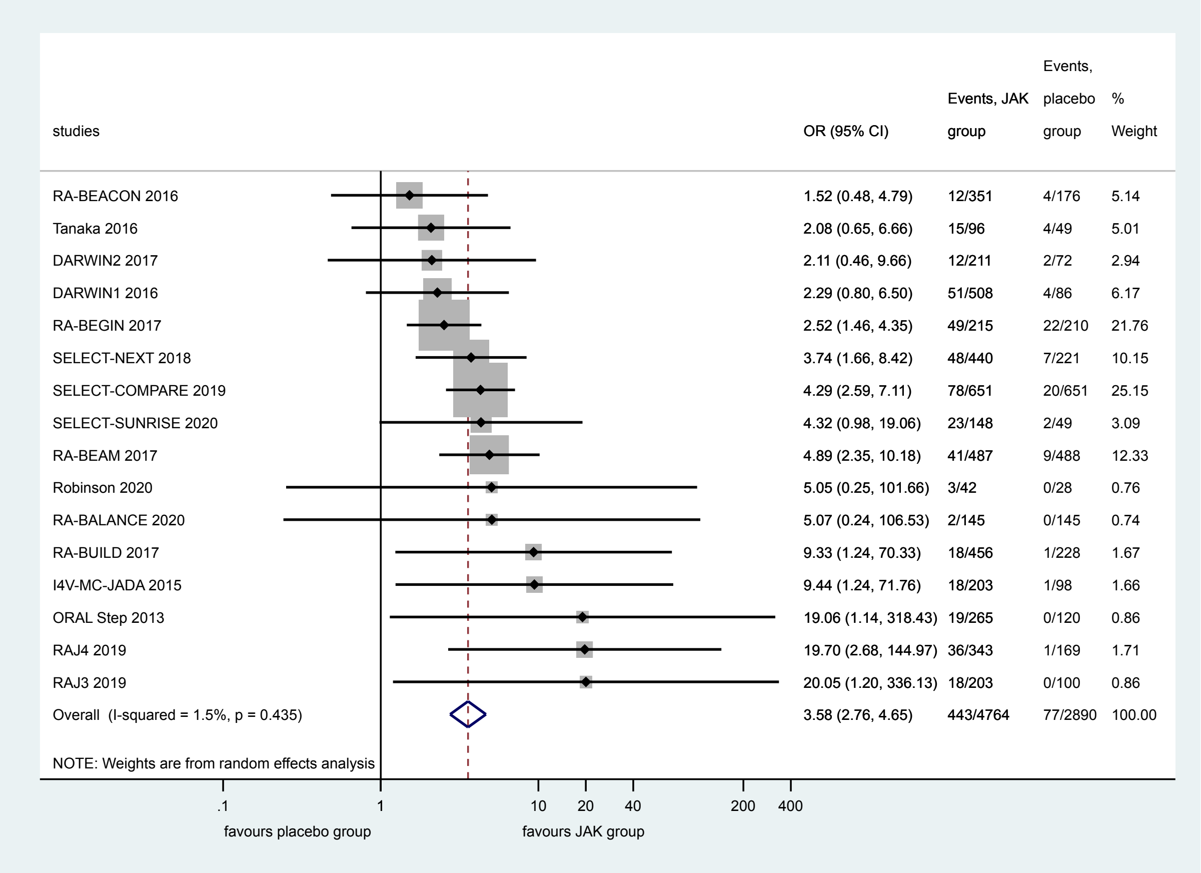

S75. Forest plot of studies comparing the number of patients reaching remission defined by SDAI (≤ 3.3) between patients treated with JAK inhibitors and placebo within 6 months (SDAI remission)

Black diamonds exhibit the individual effect of each study, whereas outer lines show the confidence intervals (CI), and the size of grey squares are proportional to the weight of each study. The blue diamond demonstrates the overall effect, the edges of which represent the CIs.

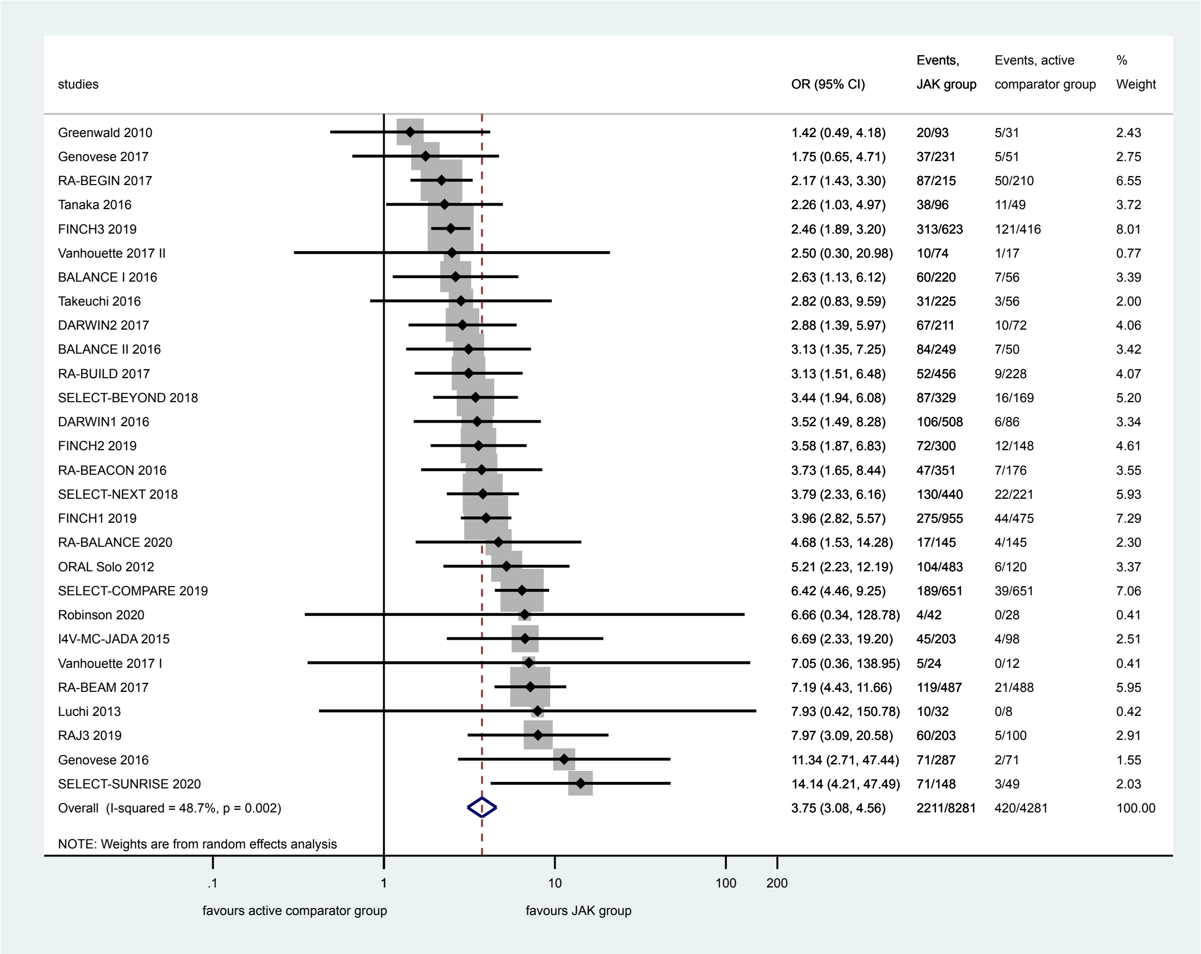

S76. Forest plot of studies comparing the number of patients reaching remission defined by DAS28-CRP (<2.6) between patients treated with JAK inhibitors and placebo within 6 months (DAS28-CRP remission)

Black diamonds exhibit the individual effect of each study, whereas outer lines show the confidence intervals (CI), and the size of grey squares are proportional to the weight of each study. The blue diamond demonstrates the overall effect, the edges of which represent the CIs.

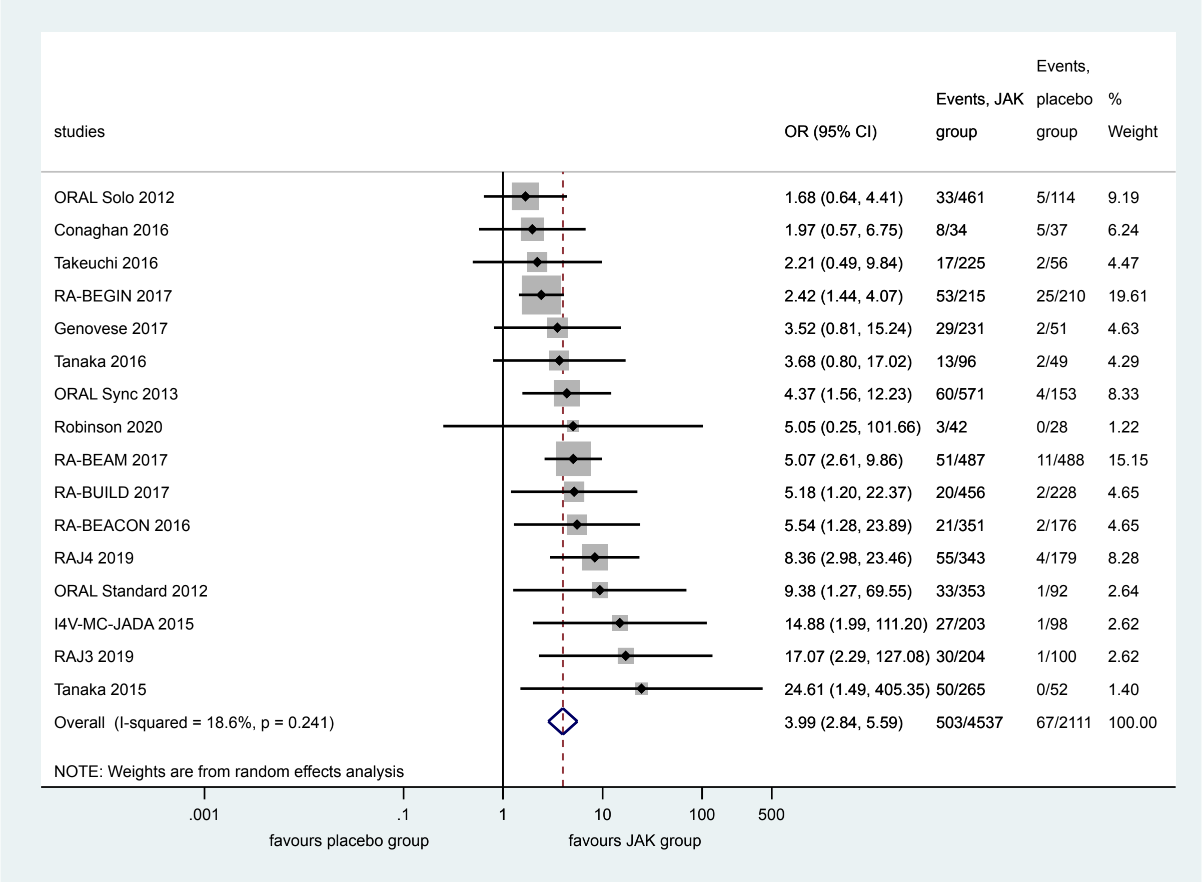

S77. Forest plot of studies comparing the number of patients reaching remission defined by DAS28-ESR (<2.6) between patients treated with JAK inhibitors and placebo within 6 months (DAS28-ESR remission)

Black diamonds exhibit the individual effect of each study, whereas outer lines show the confidence intervals (CI), and the size of grey squares are proportional to the weight of each study. The blue diamond demonstrates the overall effect, the edges of which represent the CIs.

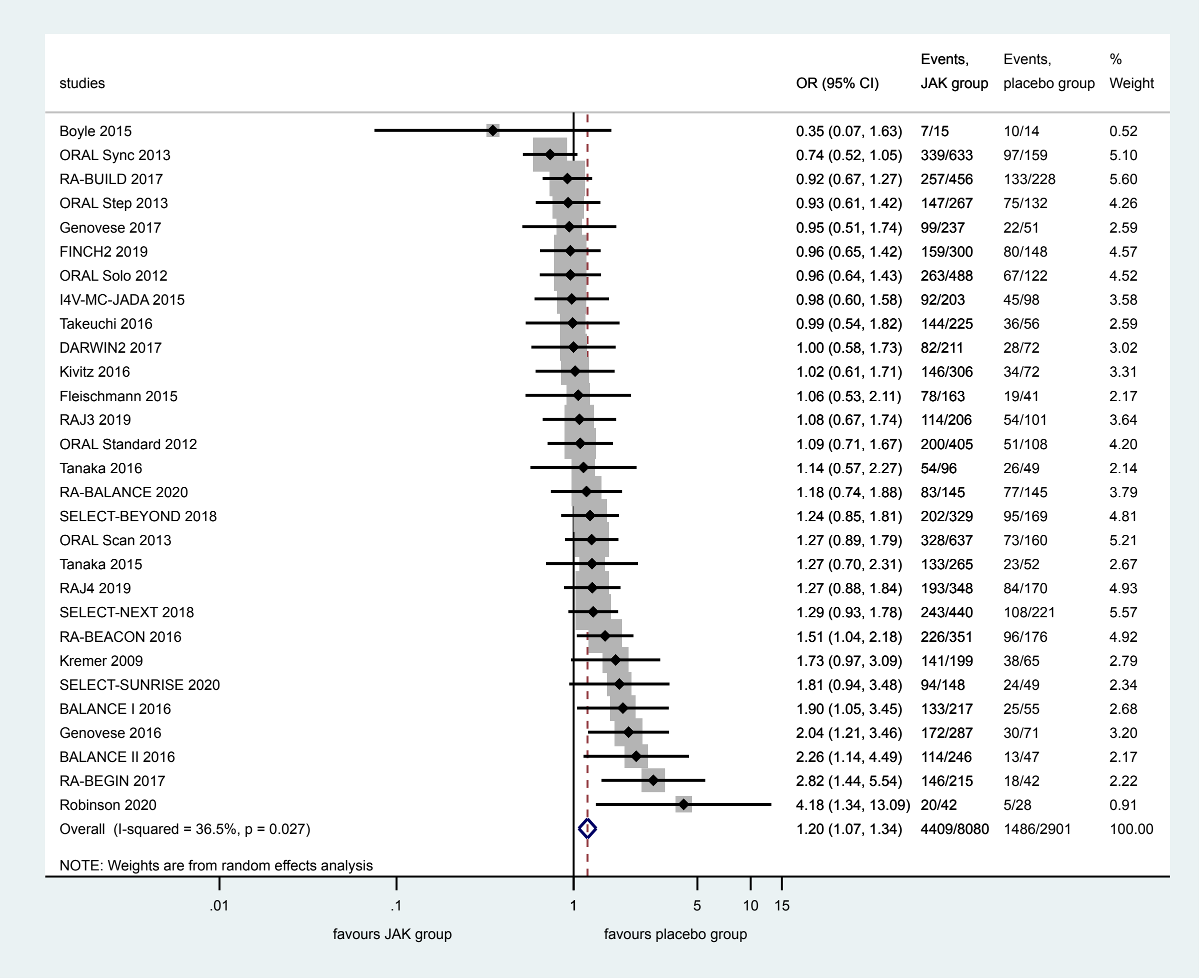

S78. Forest plot of studies comparing the number of patients experiencing side effects during the study between patients treated with JAK inhibitors and placebo within 6 months (Side effects)

Black diamonds exhibit the individual effect of each study, whereas outer lines show the confidence intervals (CI), and the size of grey squares are proportional to the weight of each study. The blue diamond demonstrates the overall effect, the edges of which represent the CIs.

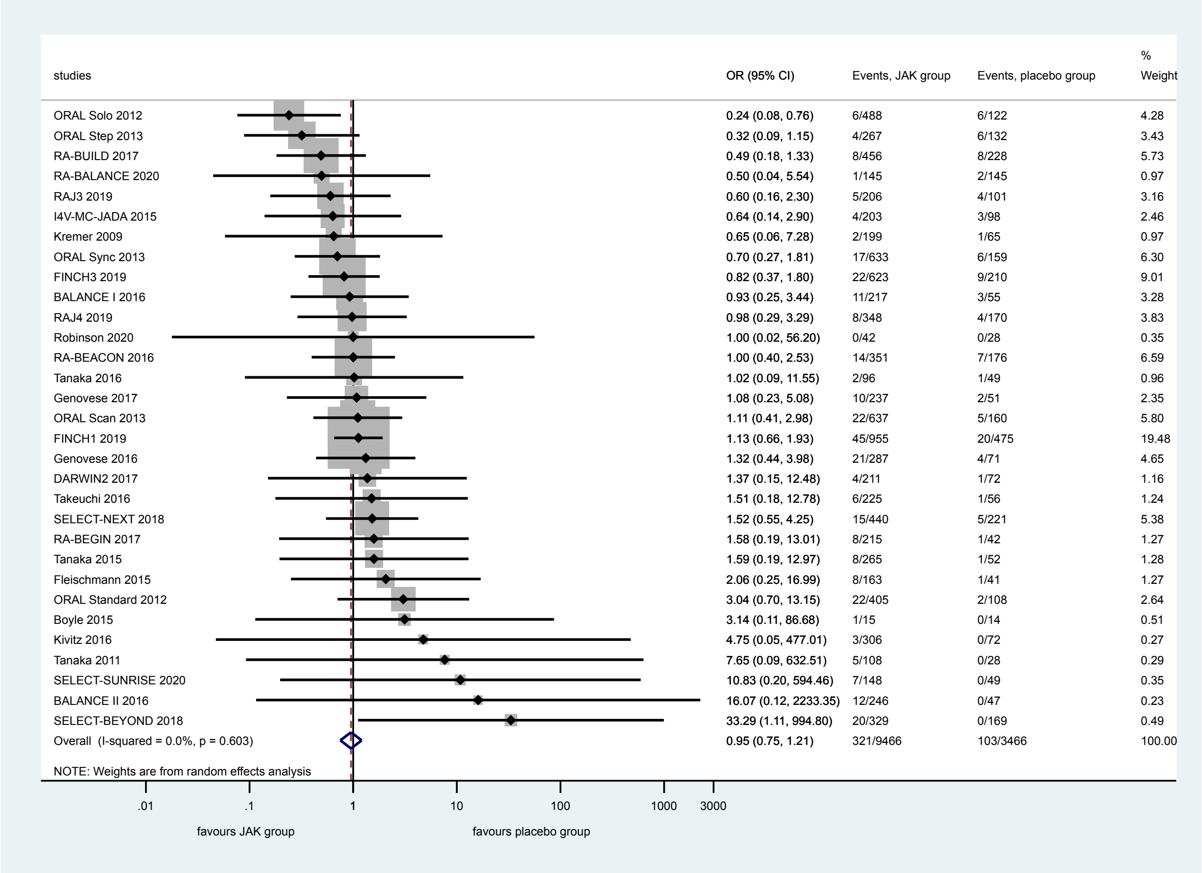

S79. Forest plot of studies comparing the number of patients experiencing serious side effects during the study between patients treated with JAK inhibitors and placebo within 6 months (Serious side effects)

Black diamonds exhibit the individual effect of each study, whereas outer lines show the confidence intervals (CI), and the size of grey squares are proportional to the weight of each study. The blue diamond demonstrates the overall effect, the edges of which represent the CIs.

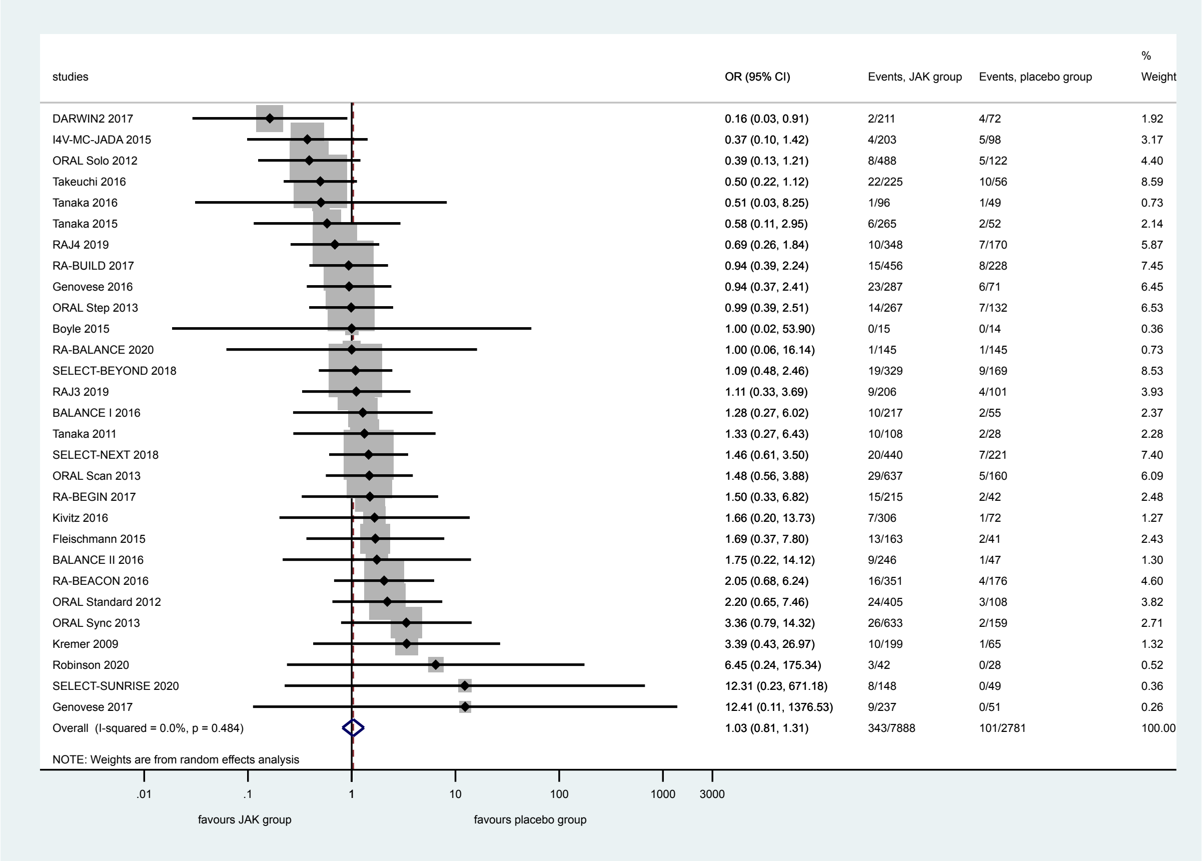

S80. Forest plot of studies comparing the number of patients experiencing symptoms leading to discontinuation of the study drugs between patients treated with JAK inhibitors and placebo within 6 months (Discontinuation)

Black diamonds exhibit the individual effect of each study, whereas outer lines show the confidence intervals (CI), and the size of grey squares are proportional to the weight of each study. The blue diamond demonstrates the overall effect, the edges of which represent the CIs.

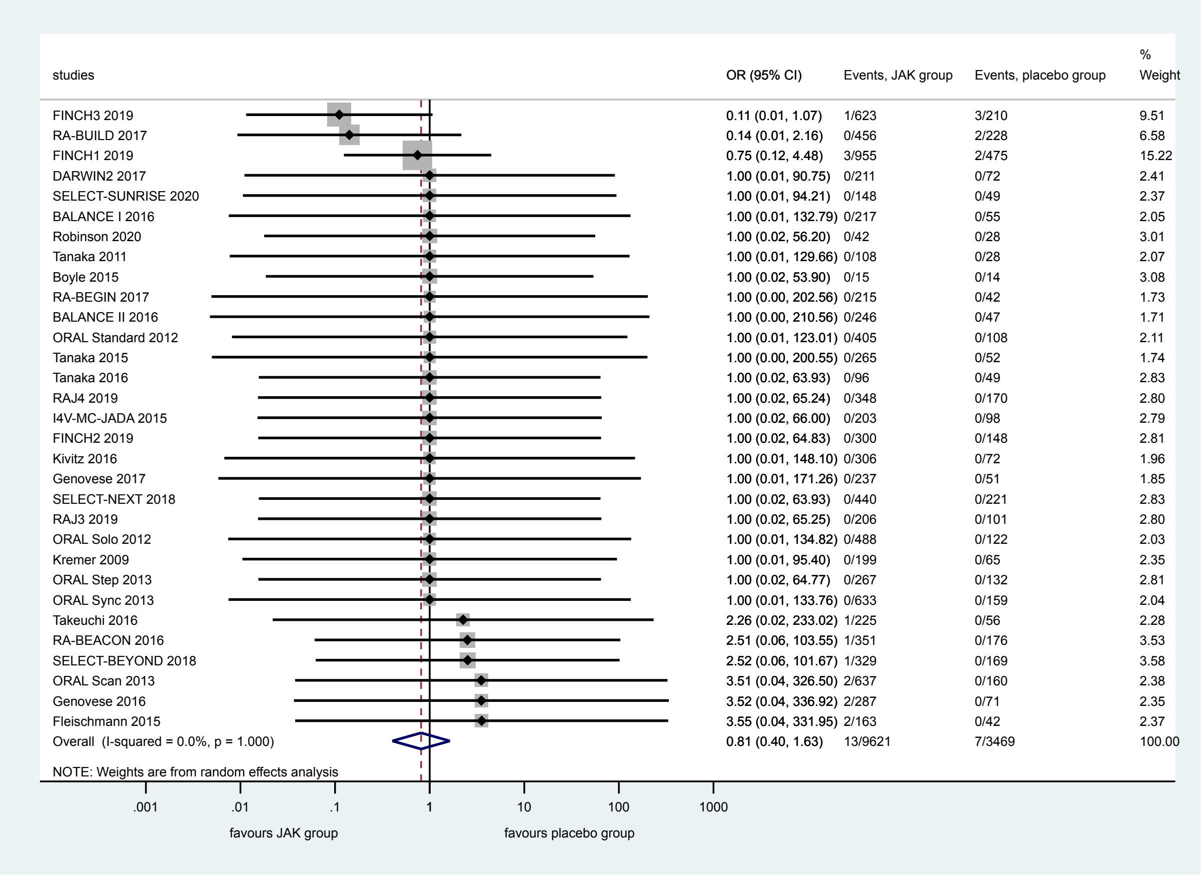

S81. Forest plot of studies comparing the number of patients who dies during study period between patients treated with JAK inhibitors and placebo (Deaths)

Black diamonds exhibit the individual effect of each study, whereas outer lines show the confidence intervals (CI), and the size of grey squares are proportional to the weight of each study. The blue diamond demonstrates the overall effect, the edges of which represent the CIs.

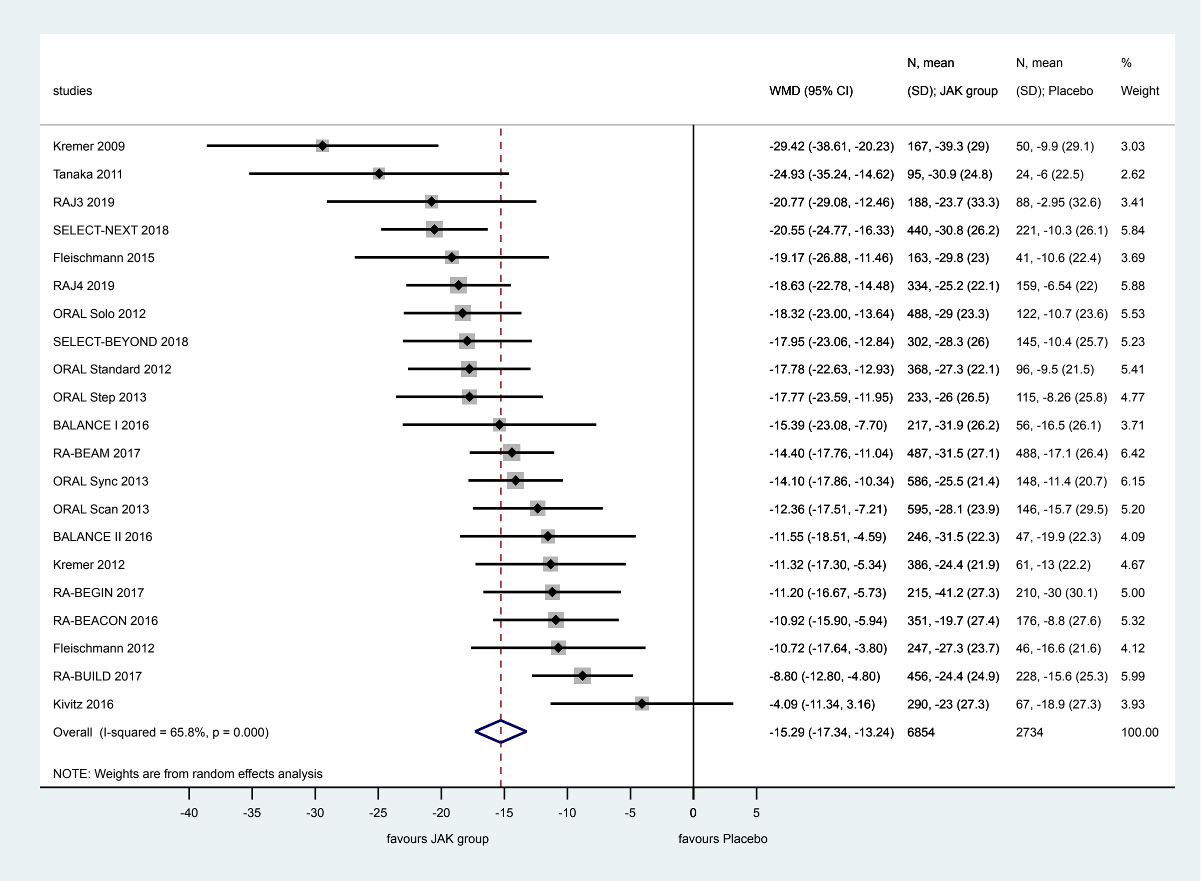

S82. Forest plot of studies comparing the LSM change from baseline in pain measured on VAS ranging from 0-100 mm between patients treated with JAK inhibitors and placebo within 6 months (Pain VAS)

Black diamonds exhibit the individual effect of each study, whereas outer lines show the confidence intervals (CI), and the size of grey squares are proportional to the weight of each study. The blue diamond demonstrates the overall effect, the edges of which represent the CIs.

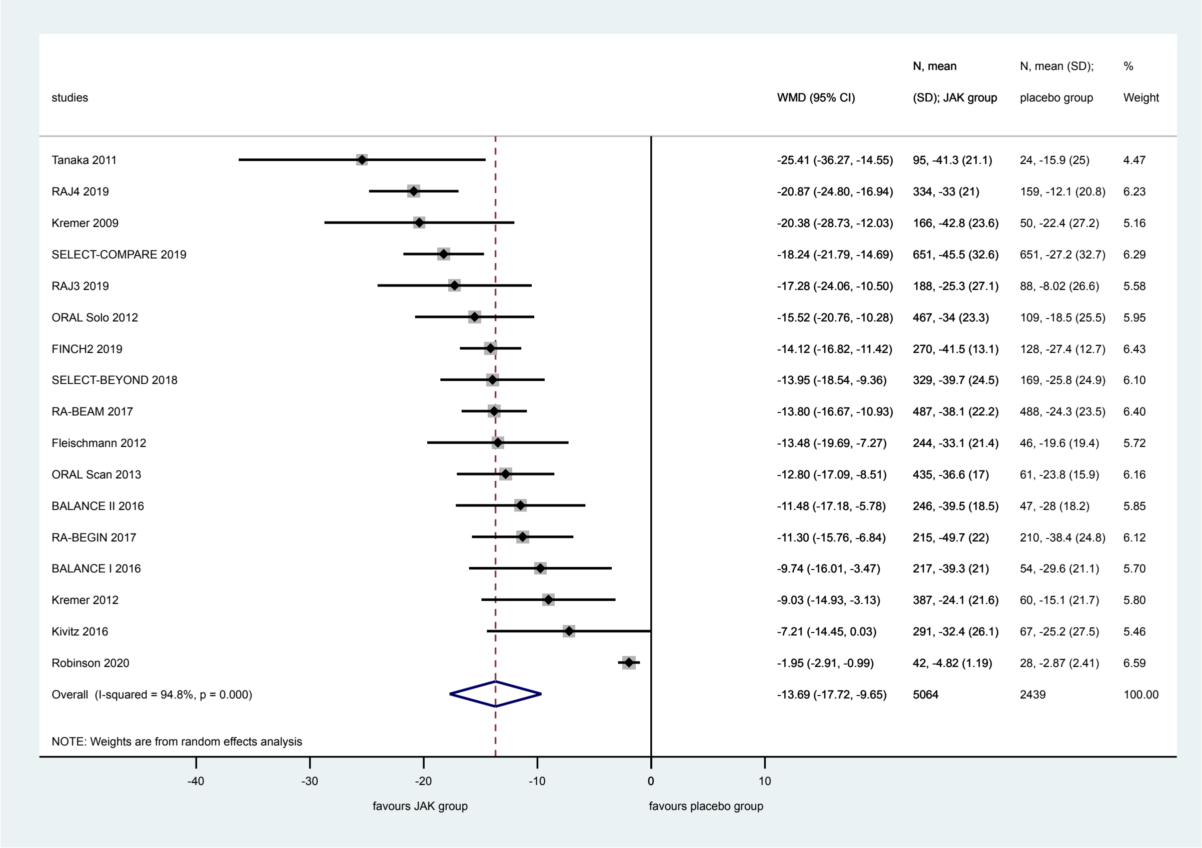

S83. Forest plot of studies comparing the LSM change from baseline in PGA measured on VAS ranging from 0-100 mm between patients treated with JAK inhibitors and placebo within 6 months (PGA VAS)

Black diamonds exhibit the individual effect of each study, whereas outer lines show the confidence intervals (CI), and the size of grey squares are proportional to the weight of each study. The blue diamond demonstrates the overall effect, the edges of which represent the CIs.

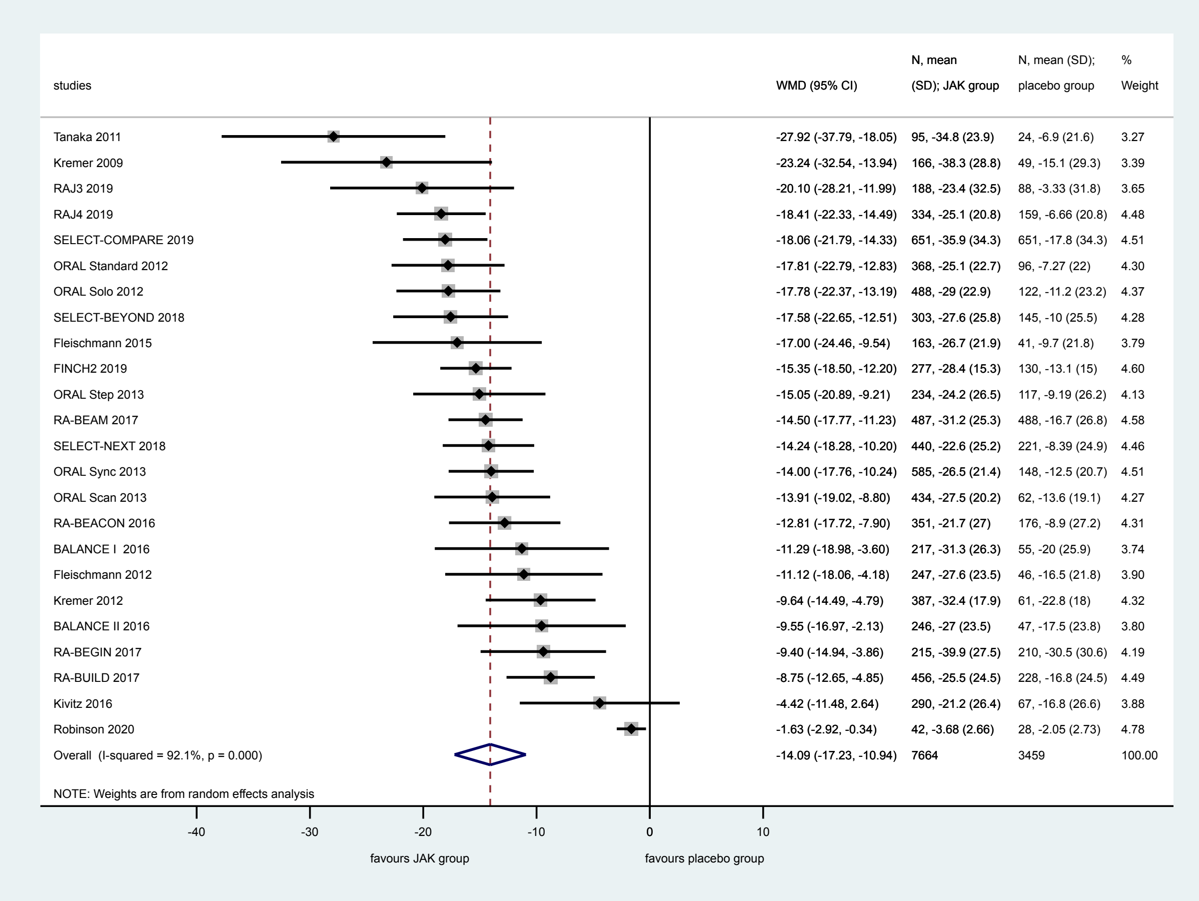

S84. Forest plot of studies comparing the LSM change from baseline in PtGA measured on VAS ranging from 0-100 mm between patients treated with JAK inhibitors and placebo within 6 months (PtGA VAS)

Black diamonds exhibit the individual effect of each study, whereas outer lines show the confidence intervals (CI), and the size of grey squares are proportional to the weight of each study. The blue diamond demonstrates the overall effect, the edges of which represent the CIs.

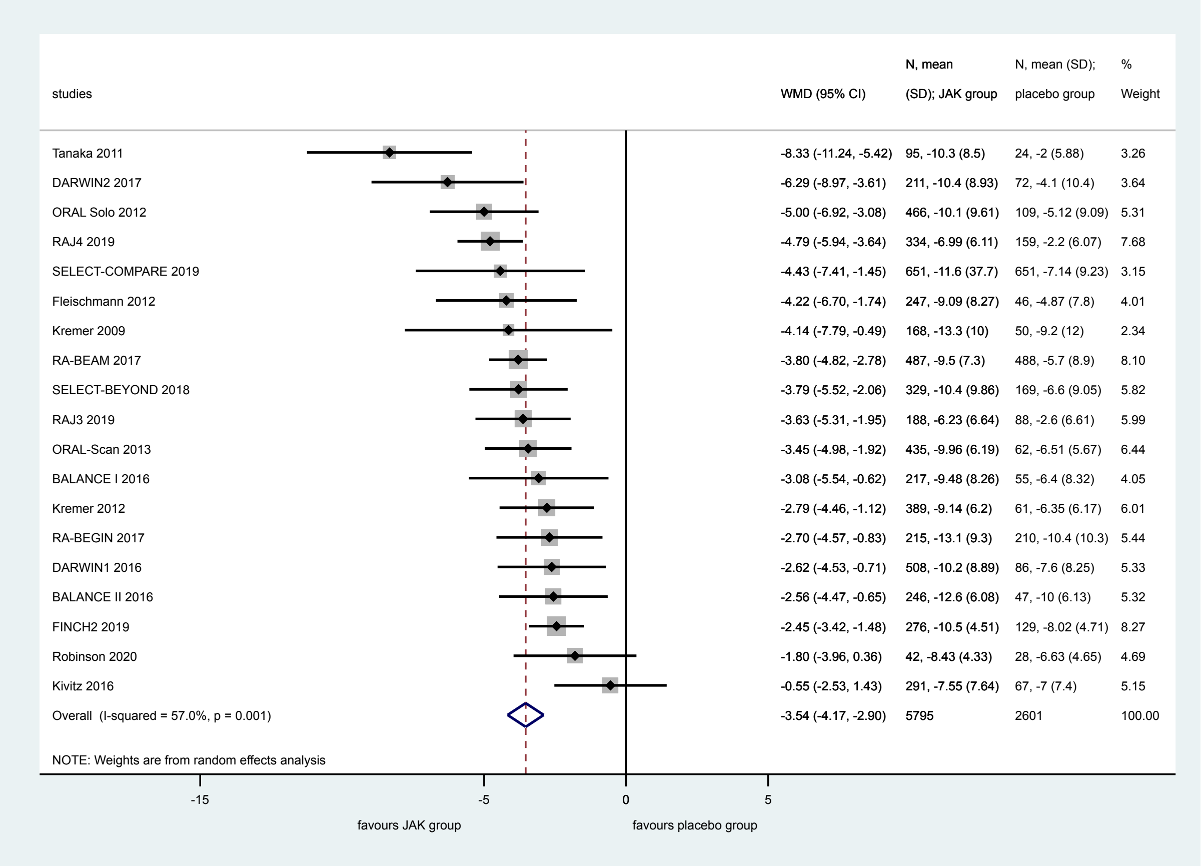

S85. Forest plot of studies comparing the LSM change from baseline in the number of swollen joint counts between patients treated with JAK inhibitors and placebo within 6 months (Swollen Joint Count)

Black diamonds exhibit the individual effect of each study, whereas outer lines show the confidence intervals (CI), and the size of grey squares are proportional to the weight of each study. The blue diamond demonstrates the overall effect, the edges of which represent the CIs.

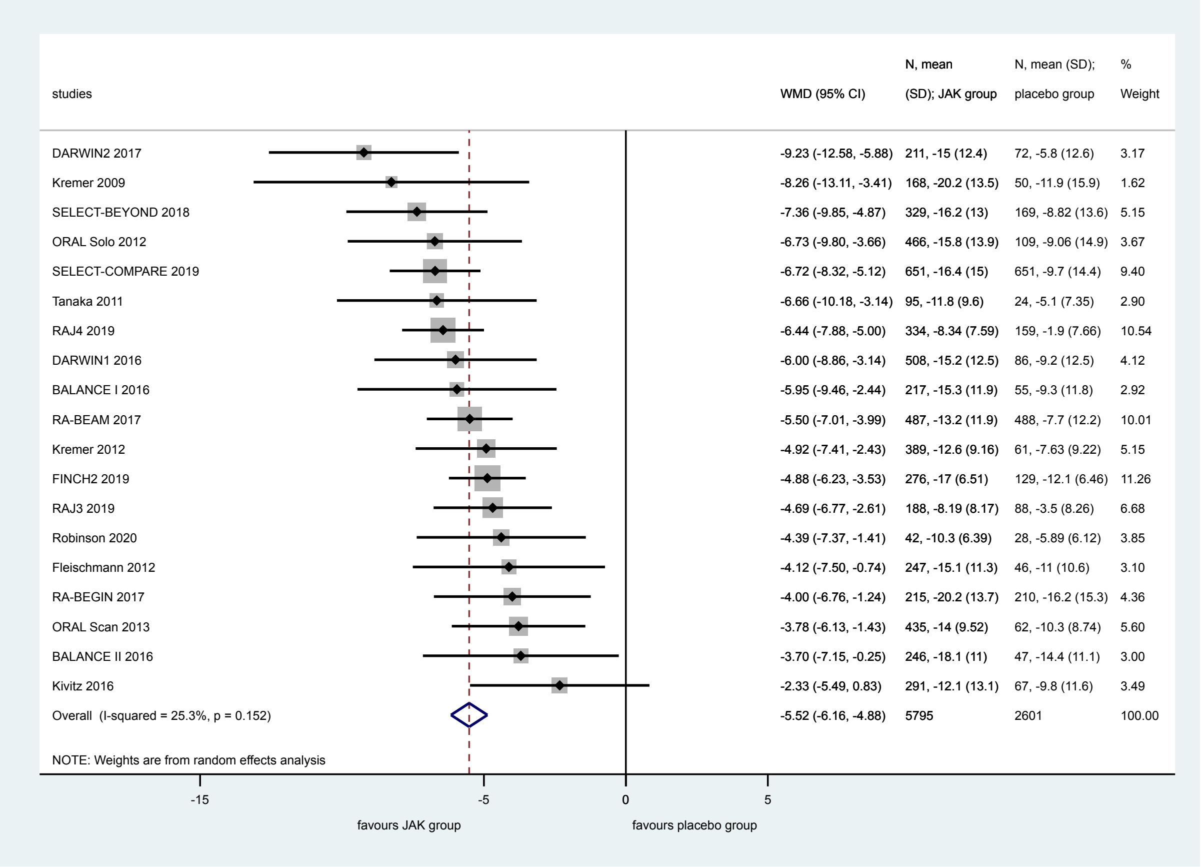

S86. Forest plot of studies comparing the LSM change from baseline in the number of tender joint counts between patients treated with JAK inhibitors and placebo within 6 months (Tender Joint Count)

Black diamonds exhibit the individual effect of each study, whereas outer lines show the confidence intervals (CI), and the size of grey squares are proportional to the weight of each study. The blue diamond demonstrates the overall effect, the edges of which represent the CIs.

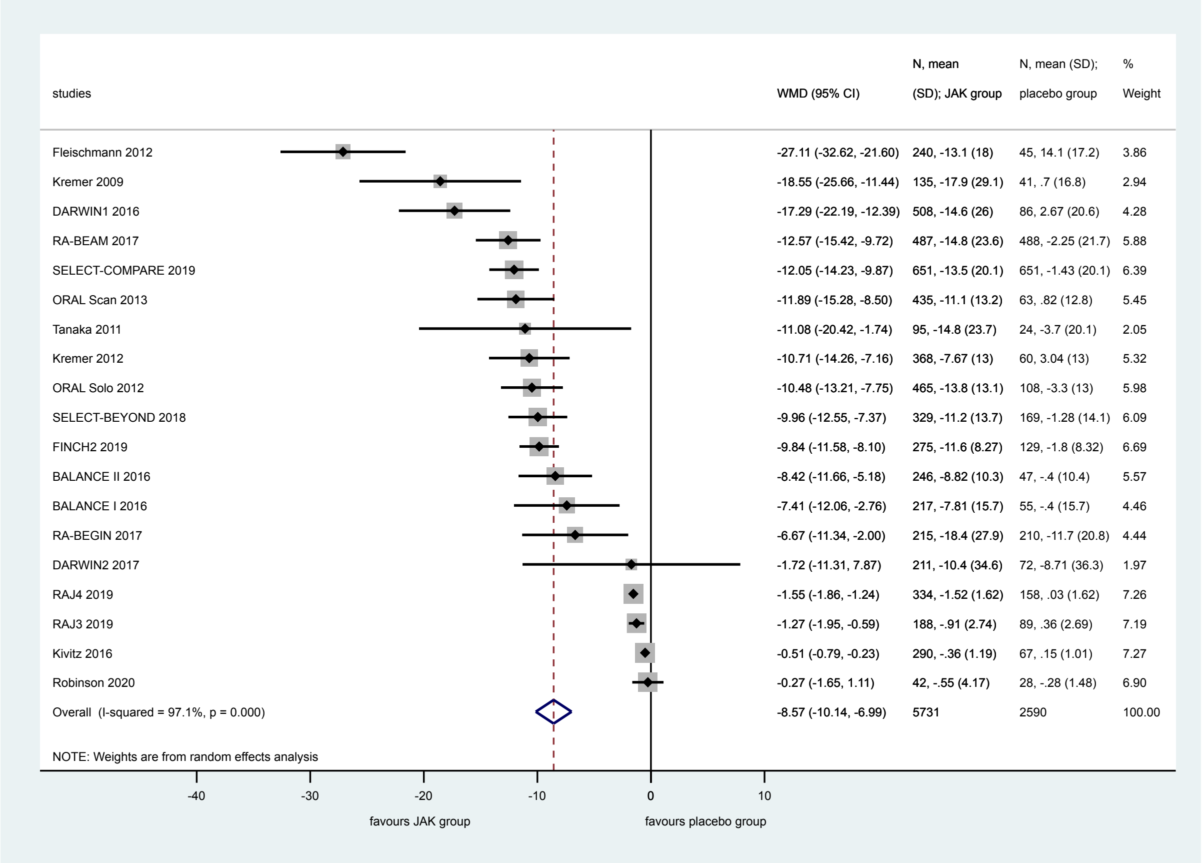

S87. Forest plot of studies comparing the LSM change from baseline in CRP value (mg/l) between patients treated with JAK inhibitors and placebo within 6 months (CRP)

Black diamonds exhibit the individual effect of each study, whereas outer lines show the confidence intervals (CI), and the size of grey squares are proportional to the weight of each study. The blue diamond demonstrates the overall effect, the edges of which represent the CIs.

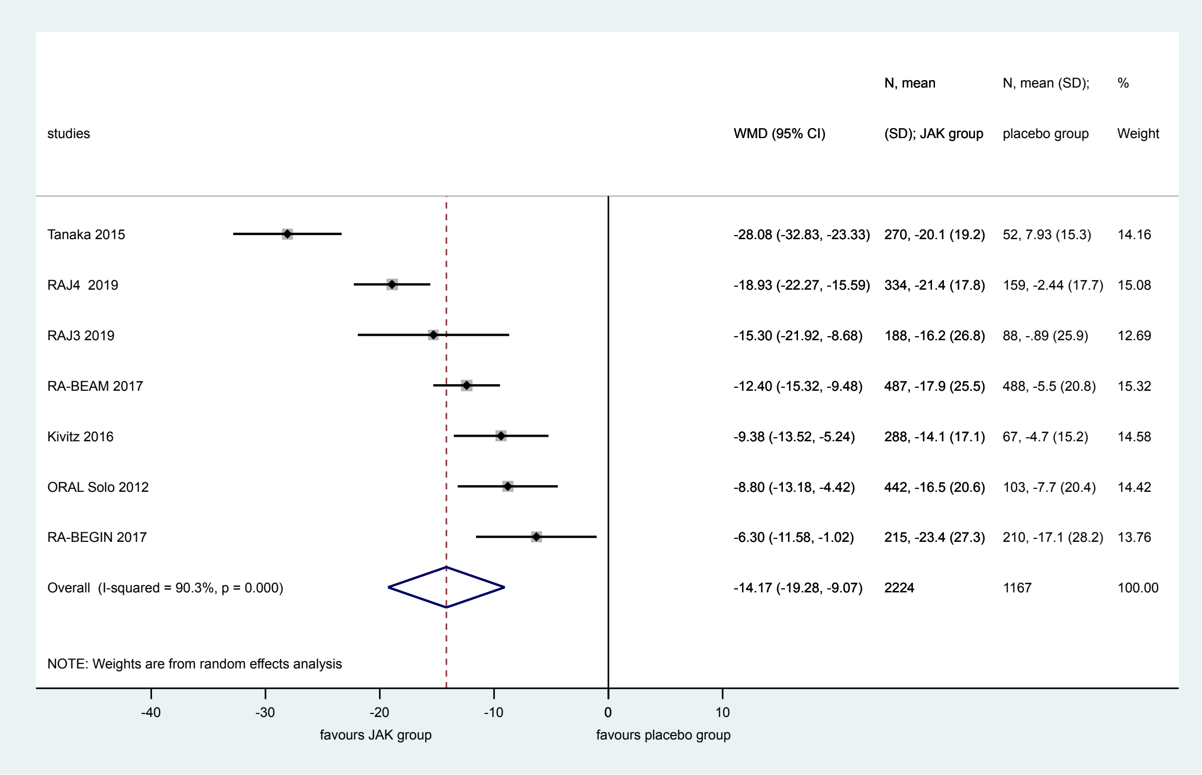

S88. Forest plot of studies comparing the LSM change from baseline in ESR value (mm/hour) between patients treated with JAK inhibitors and placebo within 6 months (ESR)

Black diamonds exhibit the individual effect of each study, whereas outer lines show the confidence intervals (CI), and the size of grey squares are proportional to the weight of each study. The blue diamond demonstrates the overall effect, the edges of which represent the CIs.

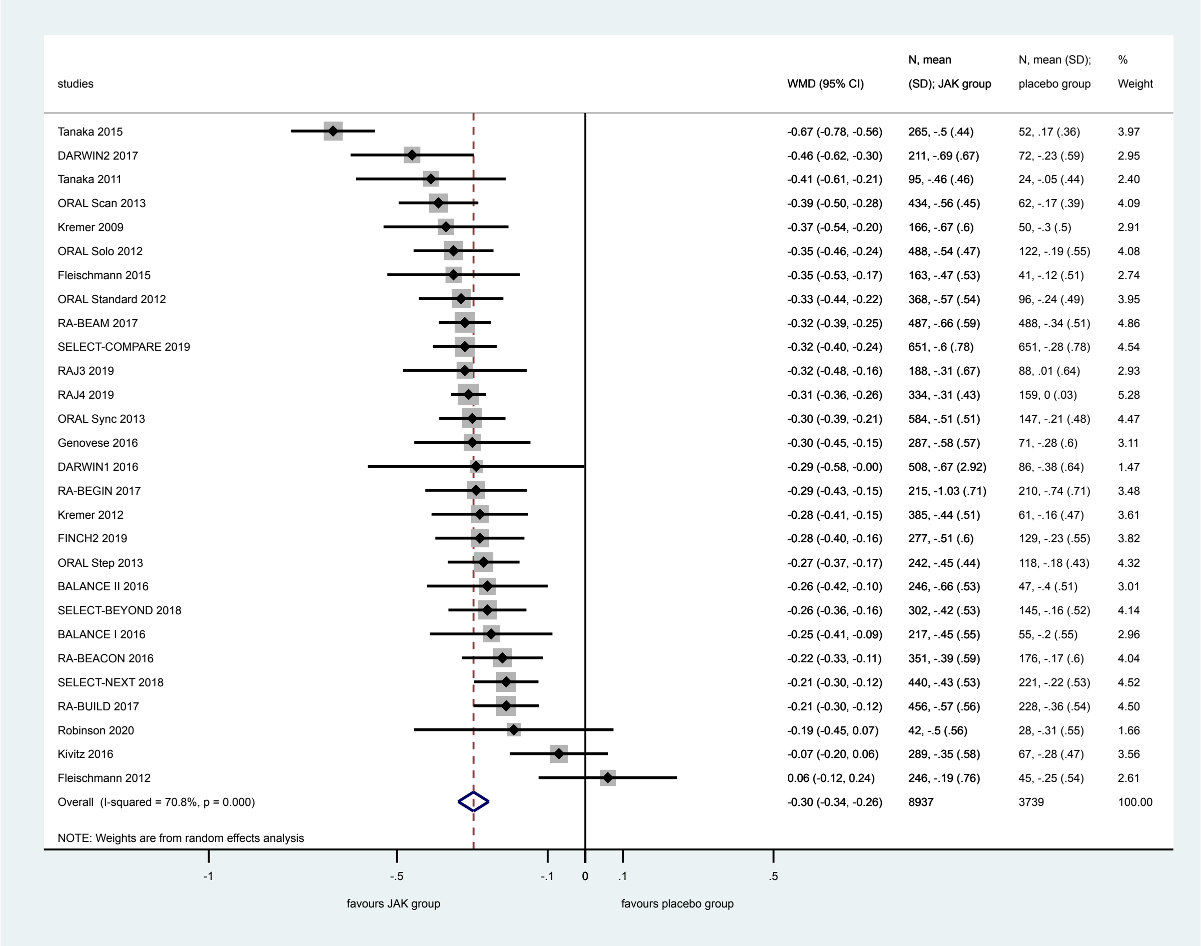

S89. Forest plot of studies comparing the LSM change from baseline in the score on HAQ-DI between patients treated with JAK inhibitors and placebo within 6 months (HAQ-DI difference)

Black diamonds exhibit the individual effect of each study, whereas outer lines show the confidence intervals (CI), and the size of grey squares are proportional to the weight of each study. The blue diamond demonstrates the overall effect, the edges of which represent the CIs.

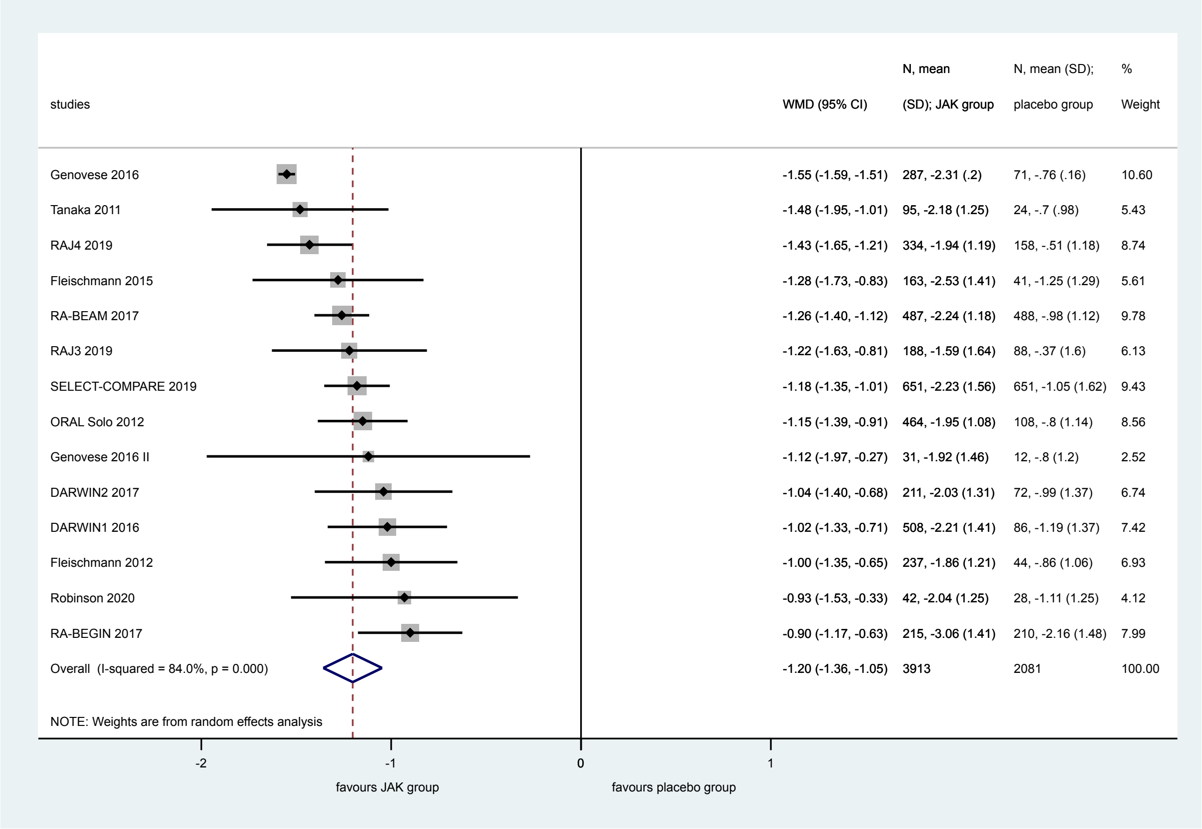

S90. Forest plot of studies comparing the LSM change from baseline in the score on DAS28-CRP between patients treated with JAK inhibitors and placebo within 6 months (DAS28-CRP difference)

Black diamonds exhibit the individual effect of each study, whereas outer lines show the confidence intervals (CI), and the size of grey squares are proportional to the weight of each study. The blue diamond demonstrates the overall effect, the edges of which represent the CIs.

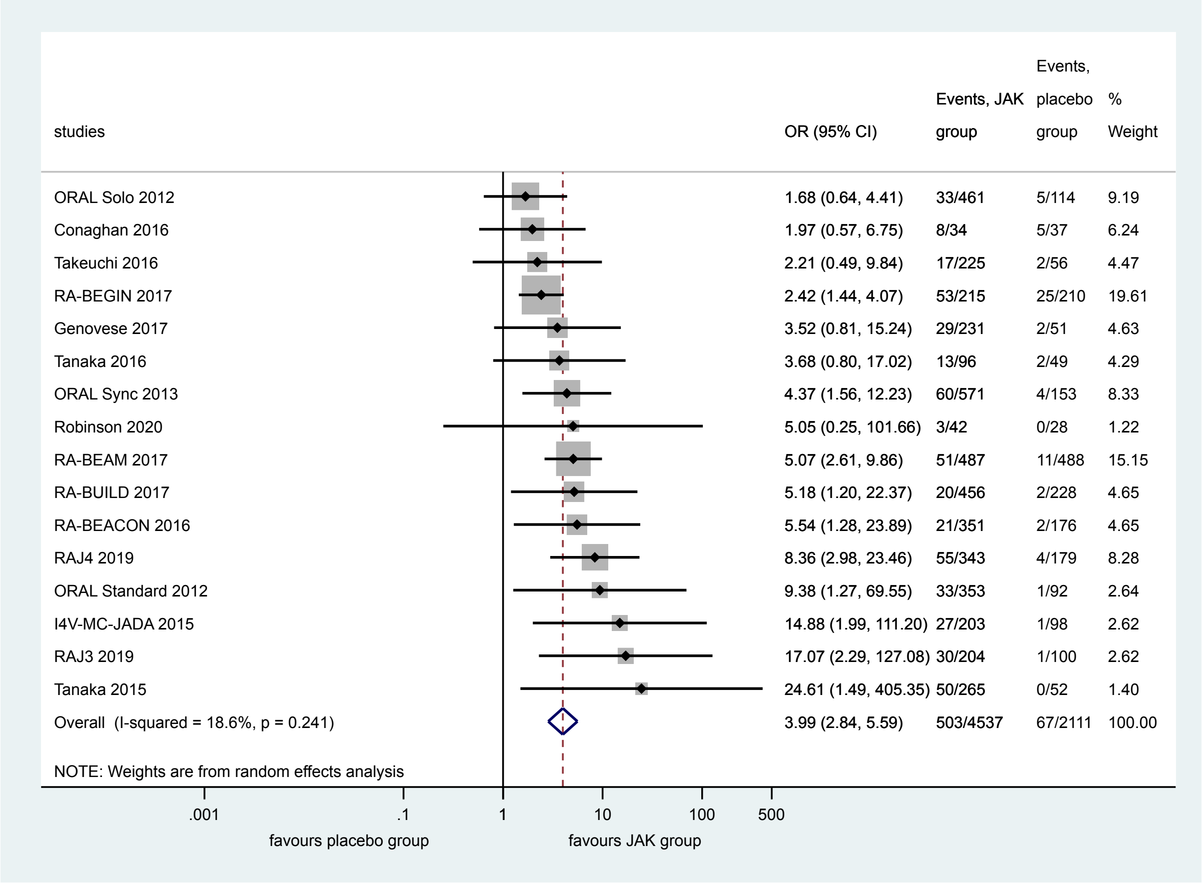

S91. Forest plot of studies comparing the LSM change from baseline in the score on DAS28-ESR between patients treated with JAK inhibitors and placebo within 6 months (DAS28-ESR difference)

Black diamonds exhibit the individual effect of each study, whereas outer lines show the confidence intervals (CI), and the size of grey squares are proportional to the weight of each study. The blue diamond demonstrates the overall effect, the edges of which represent the CIs.

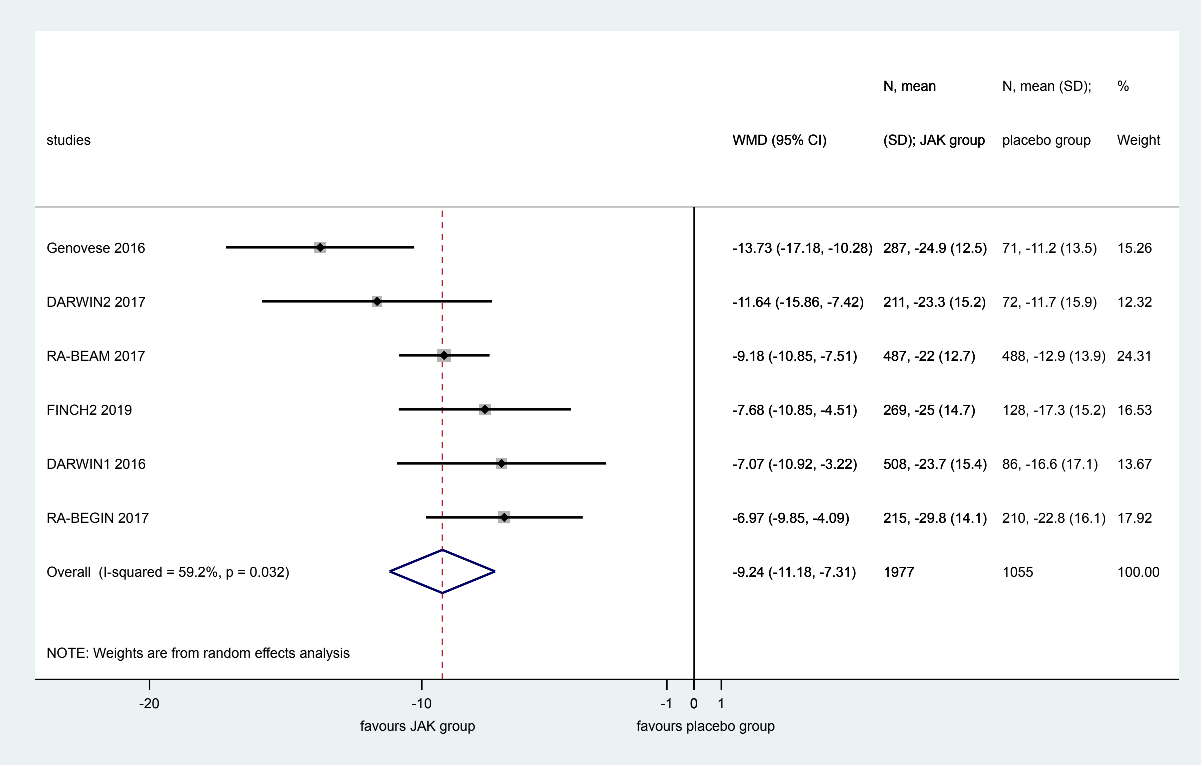

S92. Forest plot of studies comparing the LSM change from baseline in the score on CDAI between patients treated with JAK inhibitors and placebo within 6 months (CDAI difference)

Black diamonds exhibit the individual effect of each study, whereas outer lines show the confidence intervals (CI), and the size of grey squares are proportional to the weight of each study. The blue diamond demonstrates the overall effect, the edges of which represent the CIs.

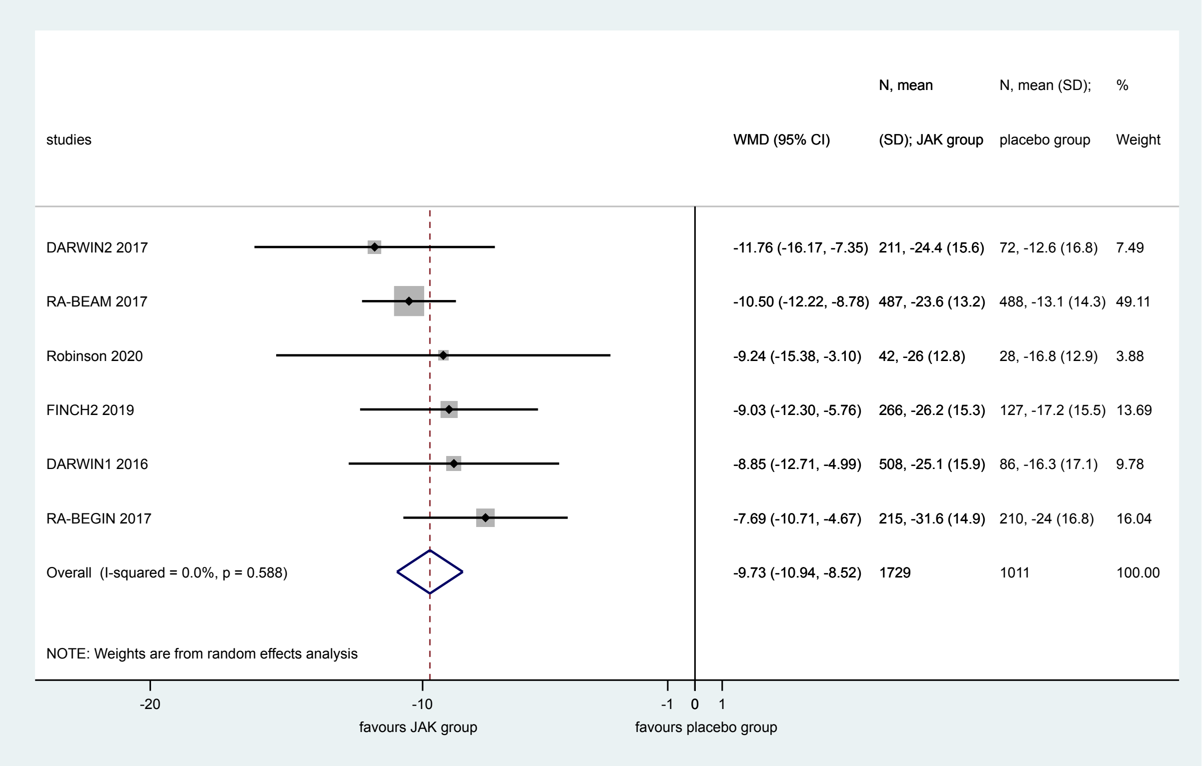

S93. Forest plot of studies comparing the LSM change from baseline in the score on SDAI between patients treated with JAK inhibitors and placebo within 6 months (SDAI difference)

Black diamonds exhibit the individual effect of each study, whereas outer lines show the confidence intervals (CI), and the size of grey squares are proportional to the weight of each study. The blue diamond demonstrates the overall effect, the edges of which represent the CIs.

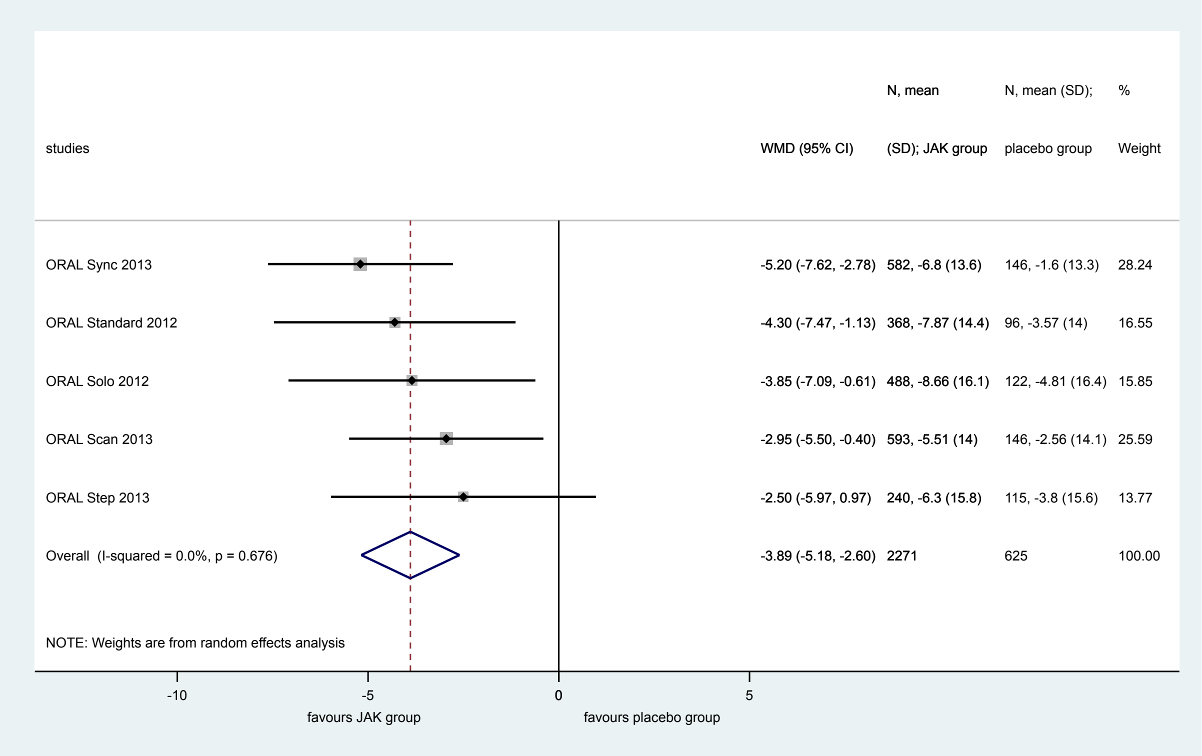

S94. Forest plot of studies comparing the LSM change from baseline in the score on MOS-Sleep between patients treated with JAK inhibitors and placebo within 6 months (MOS-Sleep)

Black diamonds exhibit the individual effect of each study, whereas outer lines show the confidence intervals (CI), and the size of grey squares are proportional to the weight of each study. The blue diamond demonstrates the overall effect, the edges of which represent the CIs.

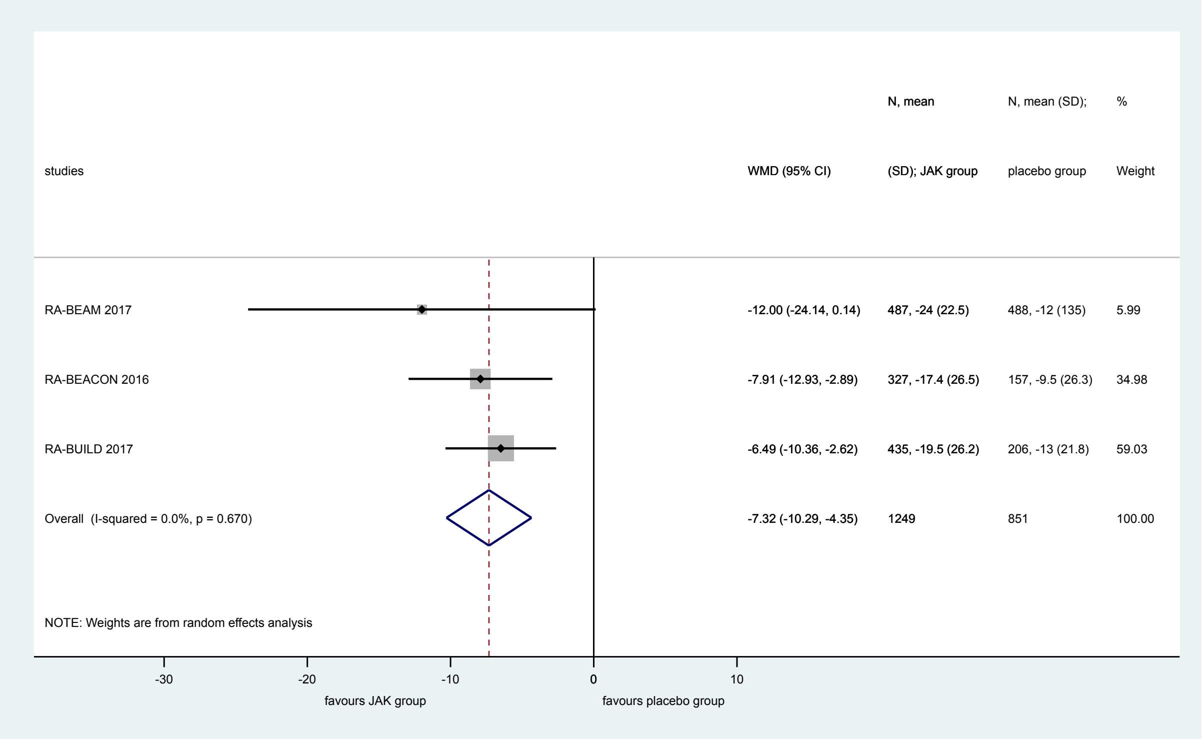

S95. Forest plot of studies comparing the LSM change from baseline in the score on WPAI assessing activity impairment between patients treated with JAK inhibitors and placebo within 6 months (WPAI AI)

Black diamonds exhibit the individual effect of each study, whereas outer lines show the confidence intervals (CI), and the size of grey squares are proportional to the weight of each study. The blue diamond demonstrates the overall effect, the edges of which represent the CIs.

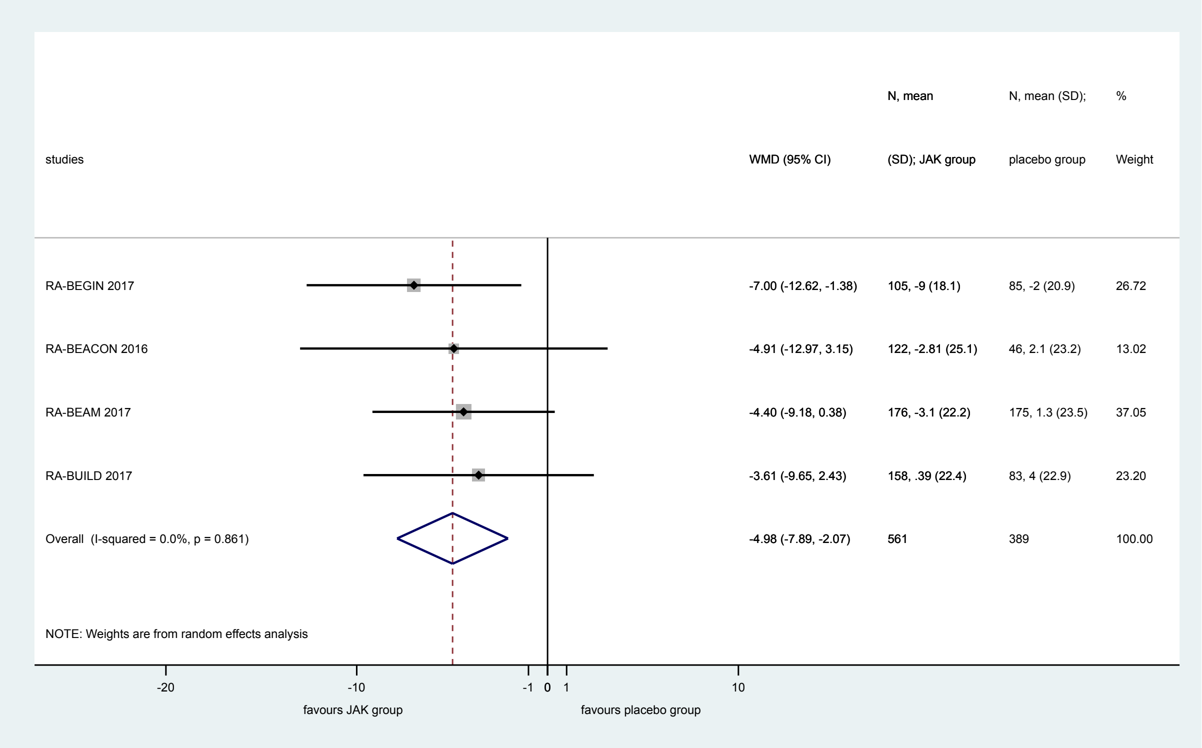

S96. Forest plot of studies comparing the LSM change from baseline in the score on WPAI assessing absenteeism (missed worked time) between patients treated with JAK inhibitors and placebo within 6 months (WPAI A)

Black diamonds exhibit the individual effect of each study, whereas outer lines show the confidence intervals (CI), and the size of grey squares are proportional to the weight of each study. The blue diamond demonstrates the overall effect, the edges of which represent the CIs.

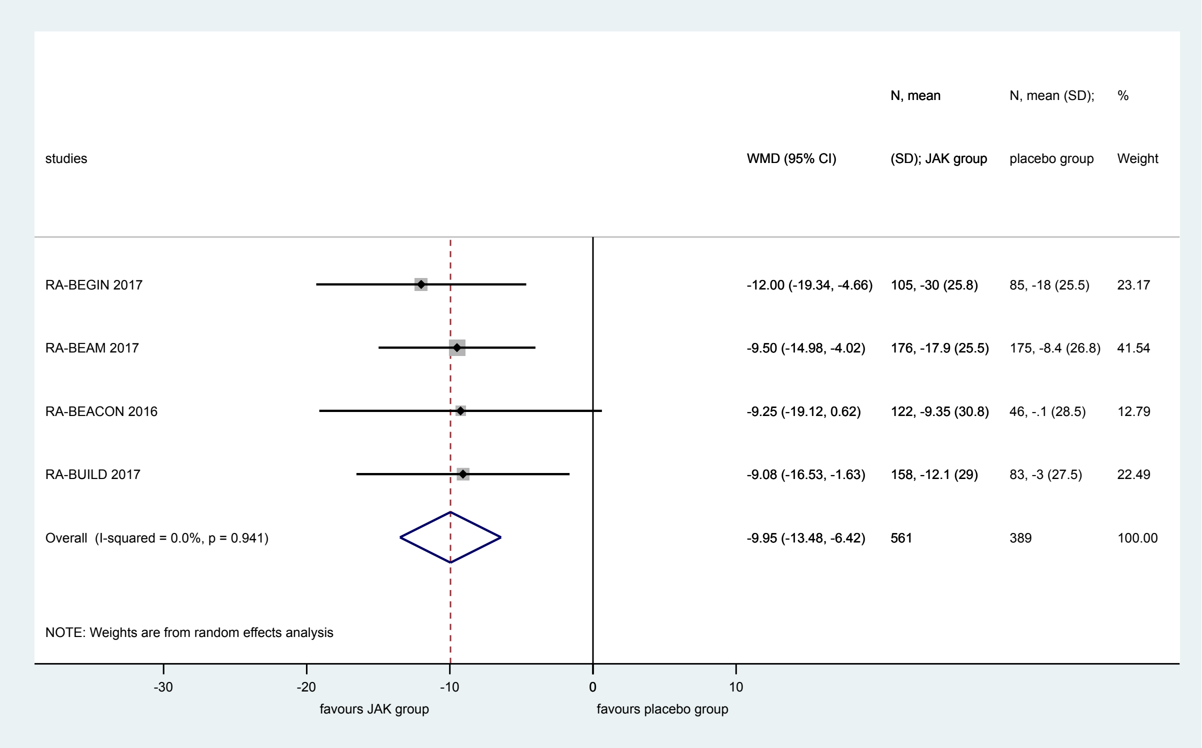

S97. Forest plot of studies comparing the LSM change from baseline in the score on WPAI assessing overall work impairment (productivity loss) between patients treated with JAK inhibitors and placebo within 6 months (WPAI OWI)

Black diamonds exhibit the individual effect of each study, whereas outer lines show the confidence intervals (CI), and the size of grey squares are proportional to the weight of each study. The blue diamond demonstrates the overall effect, the edges of which represent the CIs.

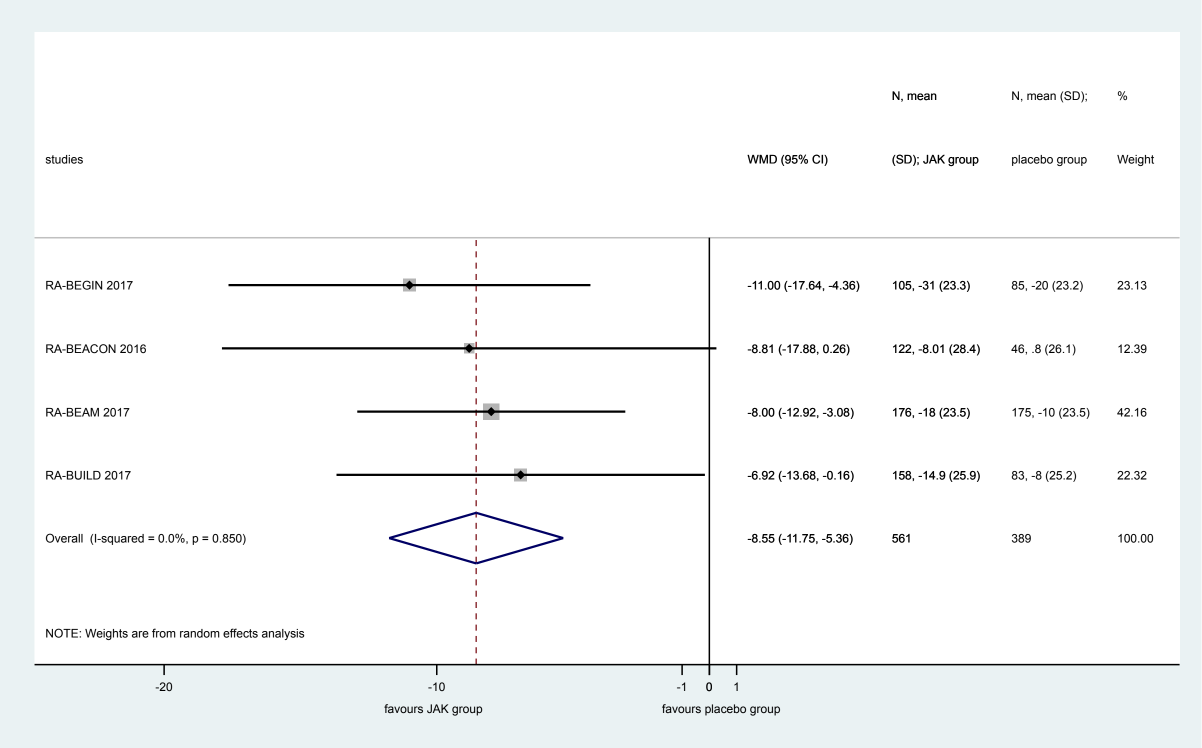

S98. Forest plot of studies comparing the LSM change from baseline in the score on WPAI assessing presenteeism (impairment while working) between patients treated with JAK inhibitors and placebo within 6 months (WPAI P)

Black diamonds exhibit the individual effect of each study, whereas outer lines show the confidence intervals (CI), and the size of grey squares are proportional to the weight of each study. The blue diamond demonstrates the overall effect, the edges of which represent the CIs.

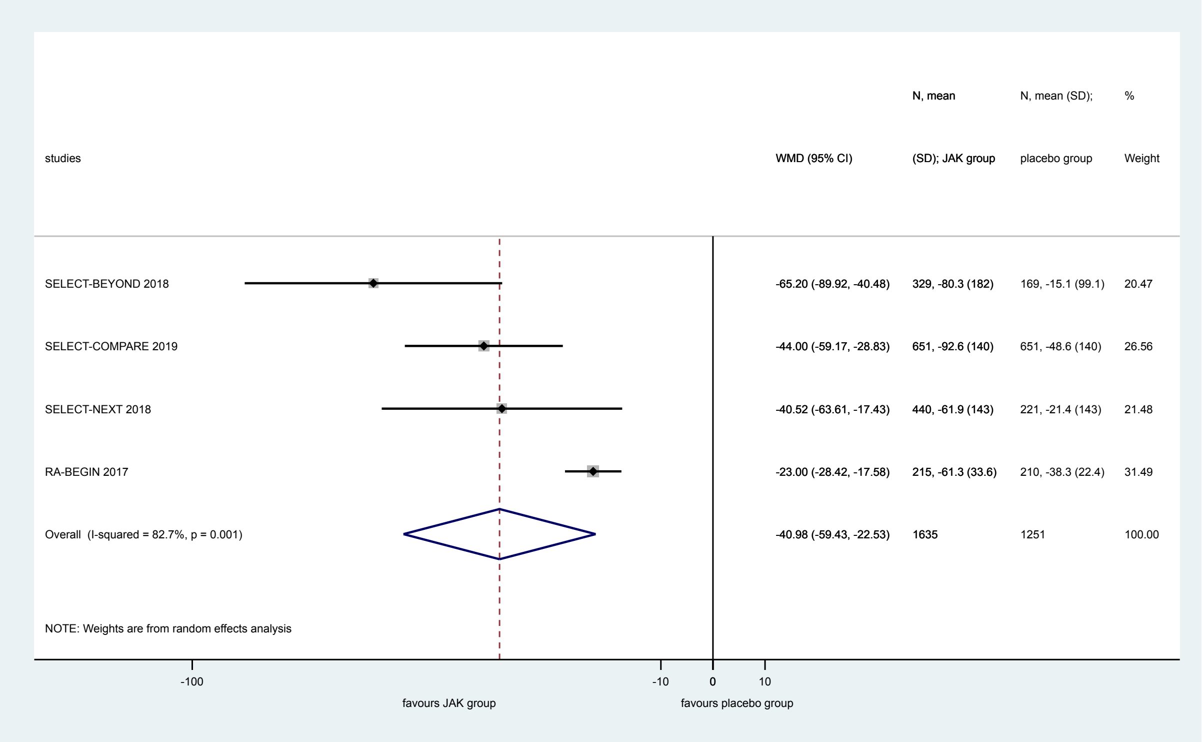

S99. Forest plot of studies comparing the LSM change from baseline in the duration of morning joint stiffness between patients treated with JAK inhibitors and placebo within 6 months (MJS duration)

Black diamonds exhibit the individual effect of each study, whereas outer lines show the confidence intervals (CI), and the size of grey squares are proportional to the weight of each study. The blue diamond demonstrates the overall effect, the edges of which represent the CIs.

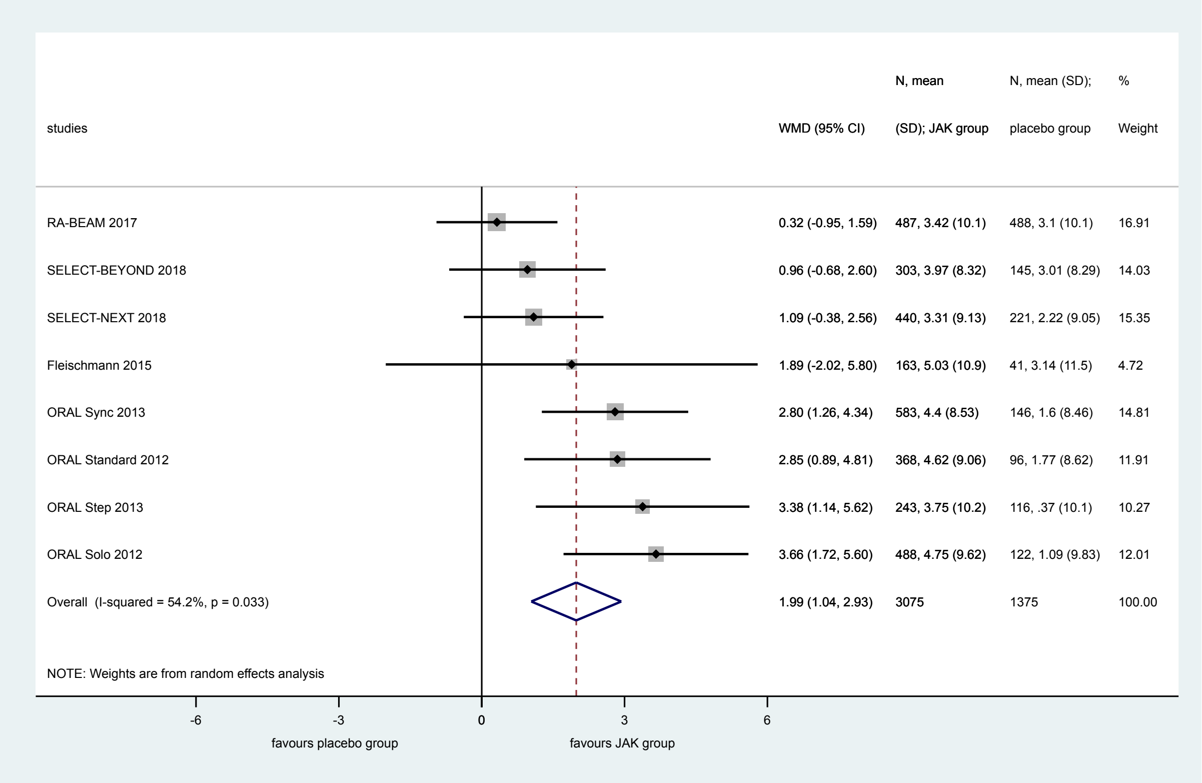

S100. Forest plot of studies comparing the LSM change from baseline in the score on SF-36 assessing the mental component score between patients treated with JAK inhibitors and placebo within 6 months (SF-36 MCS)

Black diamonds exhibit the individual effect of each study, whereas outer lines show the confidence intervals (CI), and the size of grey squares are proportional to the weight of each study. The blue diamond demonstrates the overall effect, the edges of which represent the CIs.

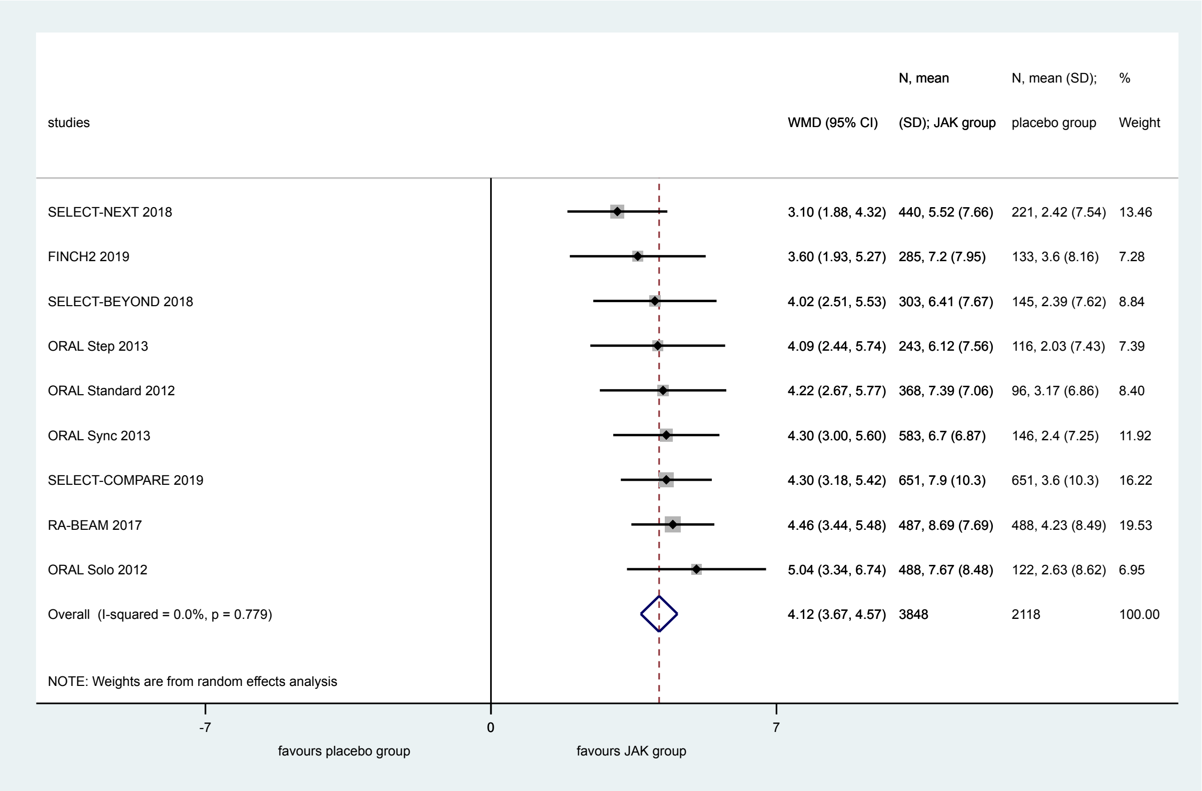

S101. Forest plot of studies comparing the LSM change from baseline in the score on SF-36 assessing the physical component score between patients treated with JAK inhibitors and placebo within 6 months (SF-36 PCS)

Black diamonds exhibit the individual effect of each study, whereas outer lines show the confidence intervals (CI), and the size of grey squares are proportional to the weight of each study. The blue diamond demonstrates the overall effect, the edges of which represent the CIs.

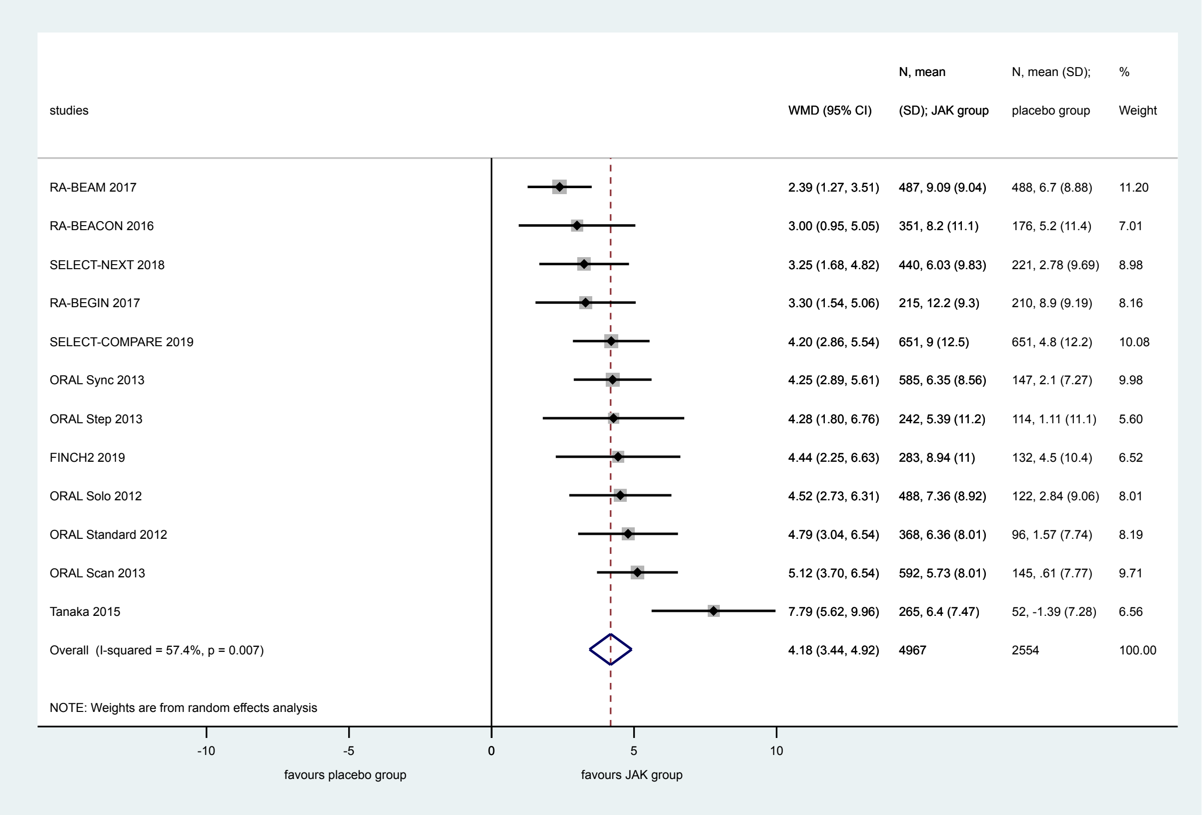

S102. Forest plot of studies comparing the LSM change from baseline in the score on FACIT-F between patients treated with JAK inhibitors and placebo within 6 months (FACIT-F)

Black diamonds exhibit the individual effect of each study, whereas outer lines show the confidence intervals (CI), and the size of grey squares are proportional to the weight of each study. The blue diamond demonstrates the overall effect, the edges of which represent the CIs.

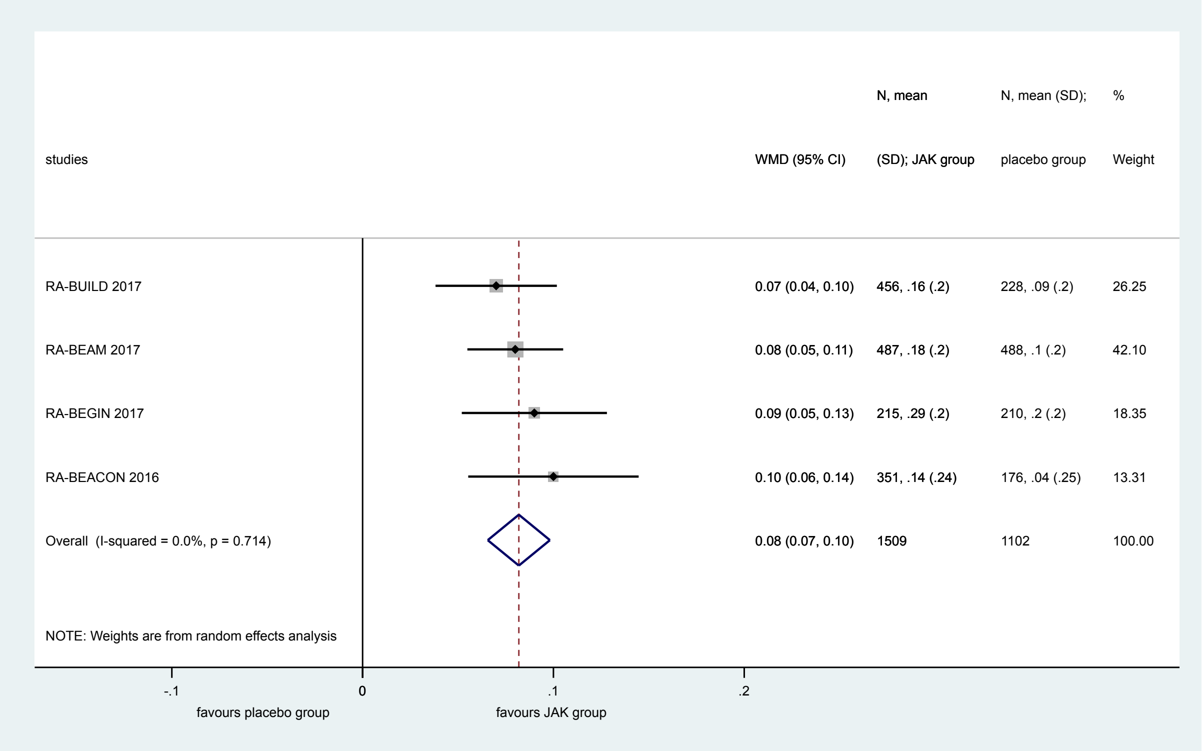

S103. Forest plot of studies comparing the LSM change from baseline in the score on EQ-5D assessed by the UK scoring algorithm between patients treated with JAK inhibitors and placebo within 6 months (EQ-5D UK)

Black diamonds exhibit the individual effect of each study, whereas outer lines show the confidence intervals (CI), and the size of grey squares are proportional to the weight of each study. The blue diamond demonstrates the overall effect, the edges of which represent the CIs.

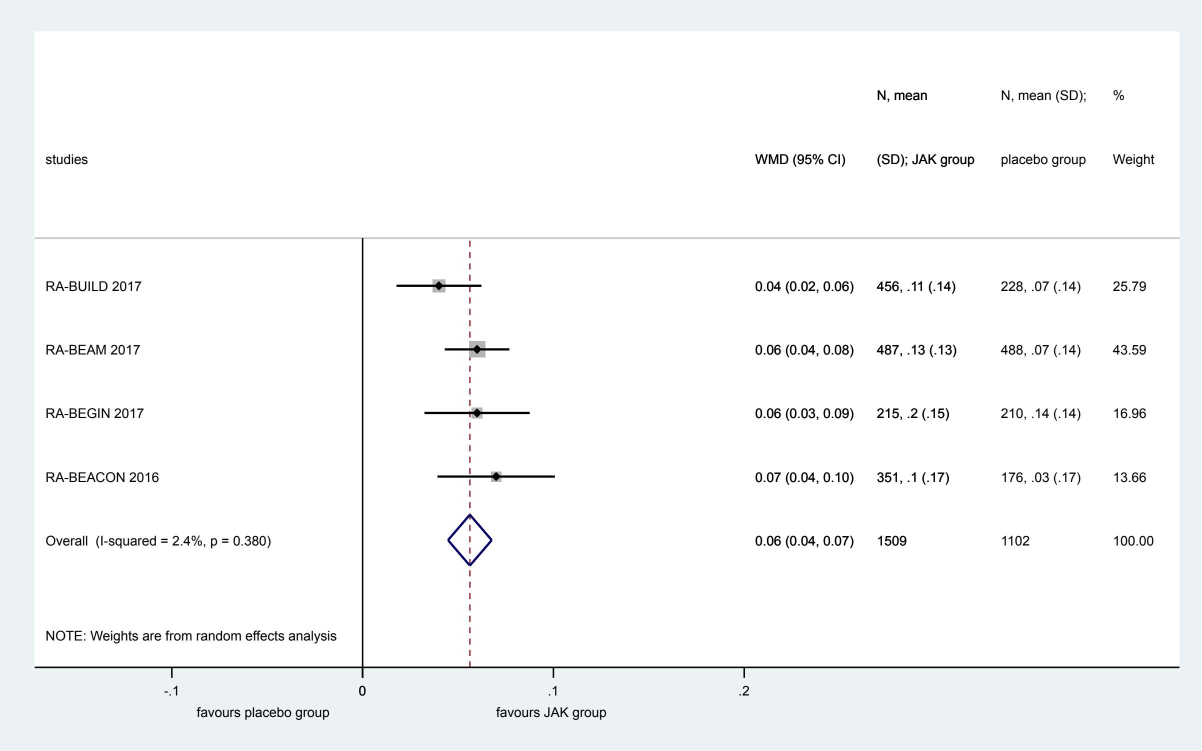

S104. Forest plot of studies comparing the LSM change from baseline in the score on EQ-5D assessed by the US scoring algorithm between patients treated with JAK inhibitors and placebo within 6 months (EQ-5D US)

Black diamonds exhibit the individual effect of each study, whereas outer lines show the confidence intervals (CI), and the size of grey squares are proportional to the weight of each study. The blue diamond demonstrates the overall effect, the edges of which represent the CIs.

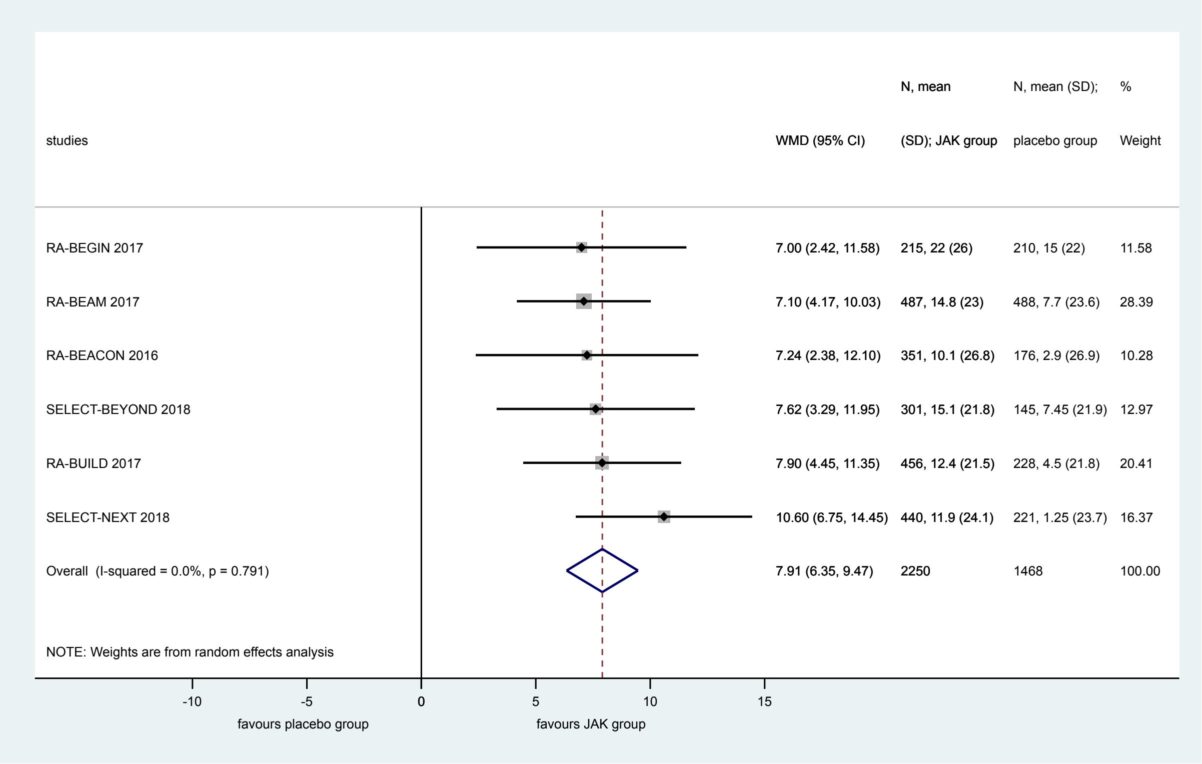

S105. Forest plot of studies comparing the LSM change from baseline in the score on EQ-5D assessed by VAS between patients treated with JAK inhibitors and placebo within 6 months (EQ-5D VAS)

Black diamonds exhibit the individual effect of each study, whereas outer lines show the confidence intervals (CI), and the size of grey squares are proportional to the weight of each study. The blue diamond demonstrates the overall effect, the edges of which represent the CIs.

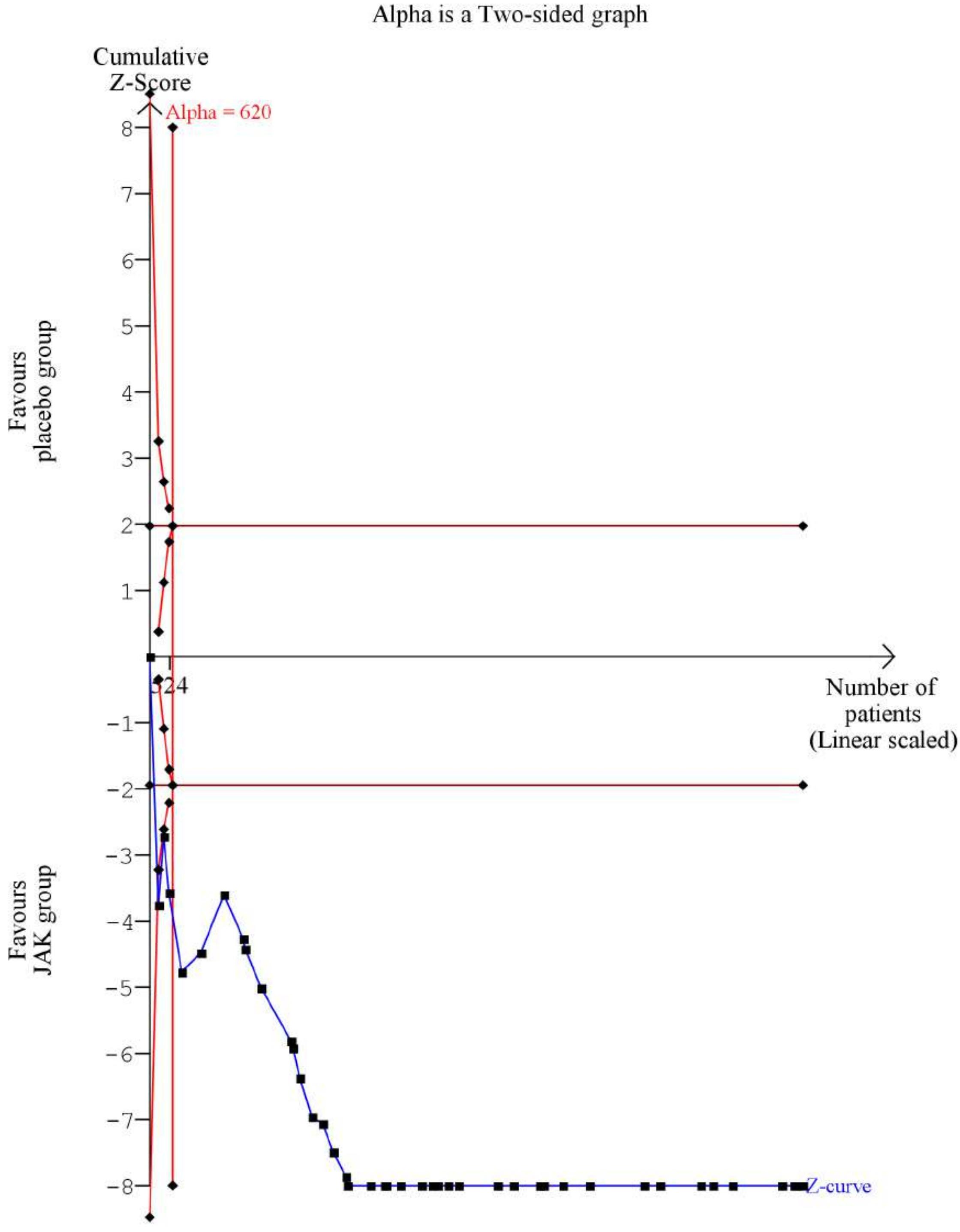

S106. TSA of ACR20 response (JAK inhibitors vs placebo)

The blue line (Z-curve) crosses the conventional boundary (brown straight line) and trial sequential boundary (red lines sloping inwards), and reaches the line of required sample size, therefore showing the statistically significant superiority of JAK inhibitors in this outcome and the addition of further studies would not influence this results.

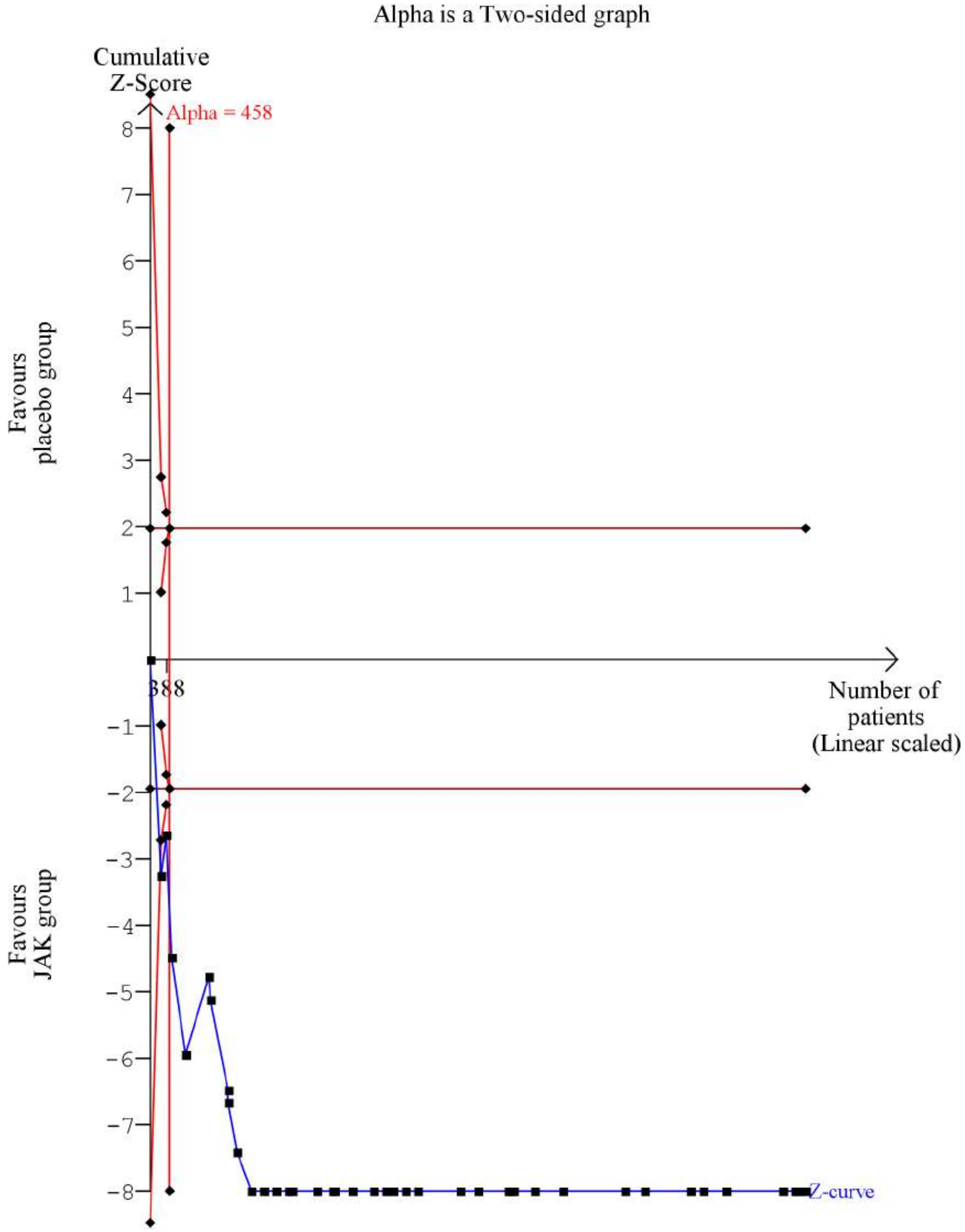

S107. TSA of ACR50 response (JAK inhibitors vs placebo)

The blue line (Z-curve) crosses the conventional boundary (brown straight line) and trial sequential boundary (red lines sloping inwards), and reaches the line of required sample size, therefore showing the statistically significant superiority of JAK inhibitors in this outcome and the addition of further studies would not influence these results.

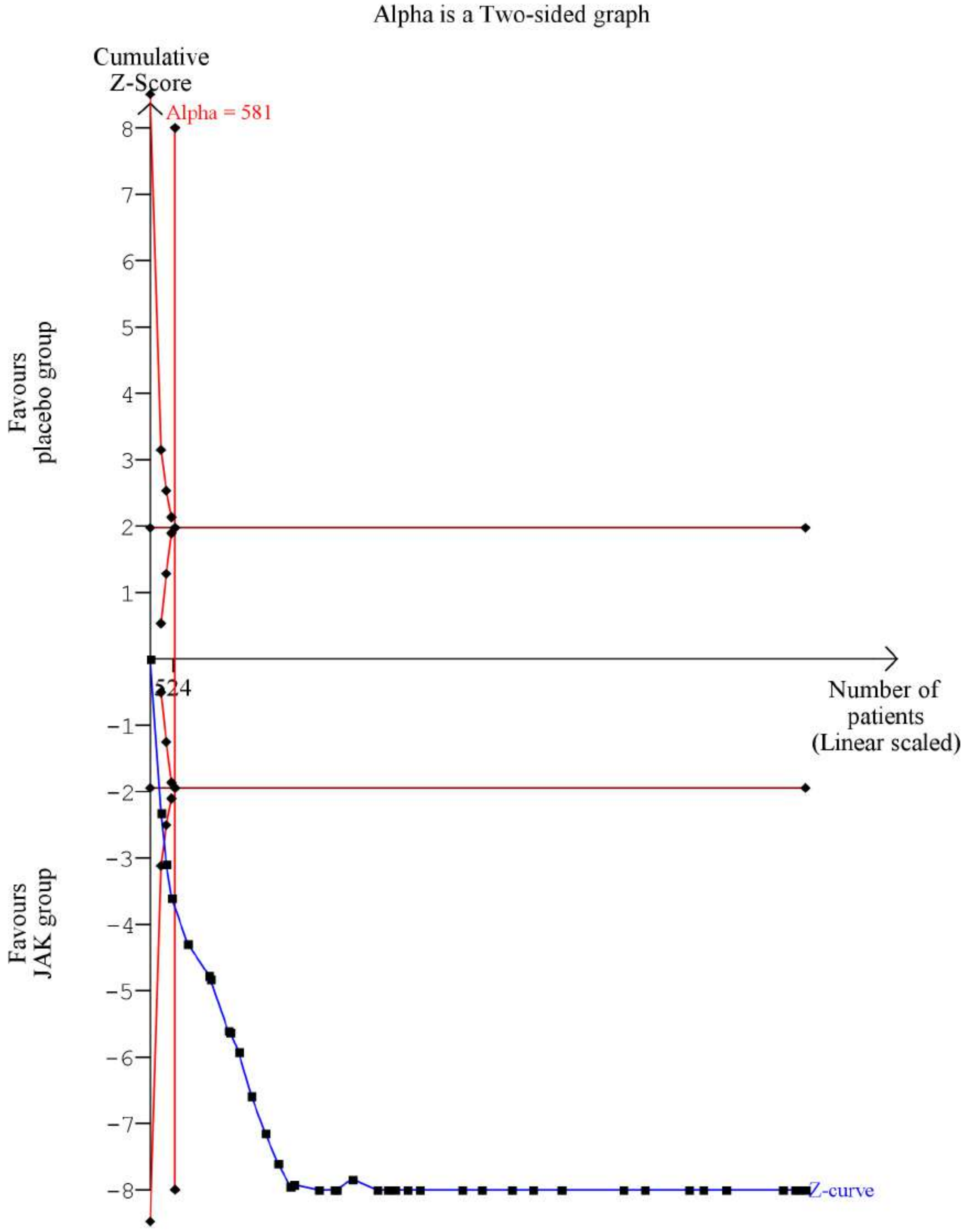

S108. TSA of ACR70 response (JAK inhibitors vs placebo)

The blue line (Z-curve) crosses the conventional boundary (brown straight line) and trial sequential boundary (red lines sloping inwards), and reaches the line of required sample size, therefore showing the statistically significant superiority of JAK inhibitors in this outcome and the addition of further studies would not influence these results.

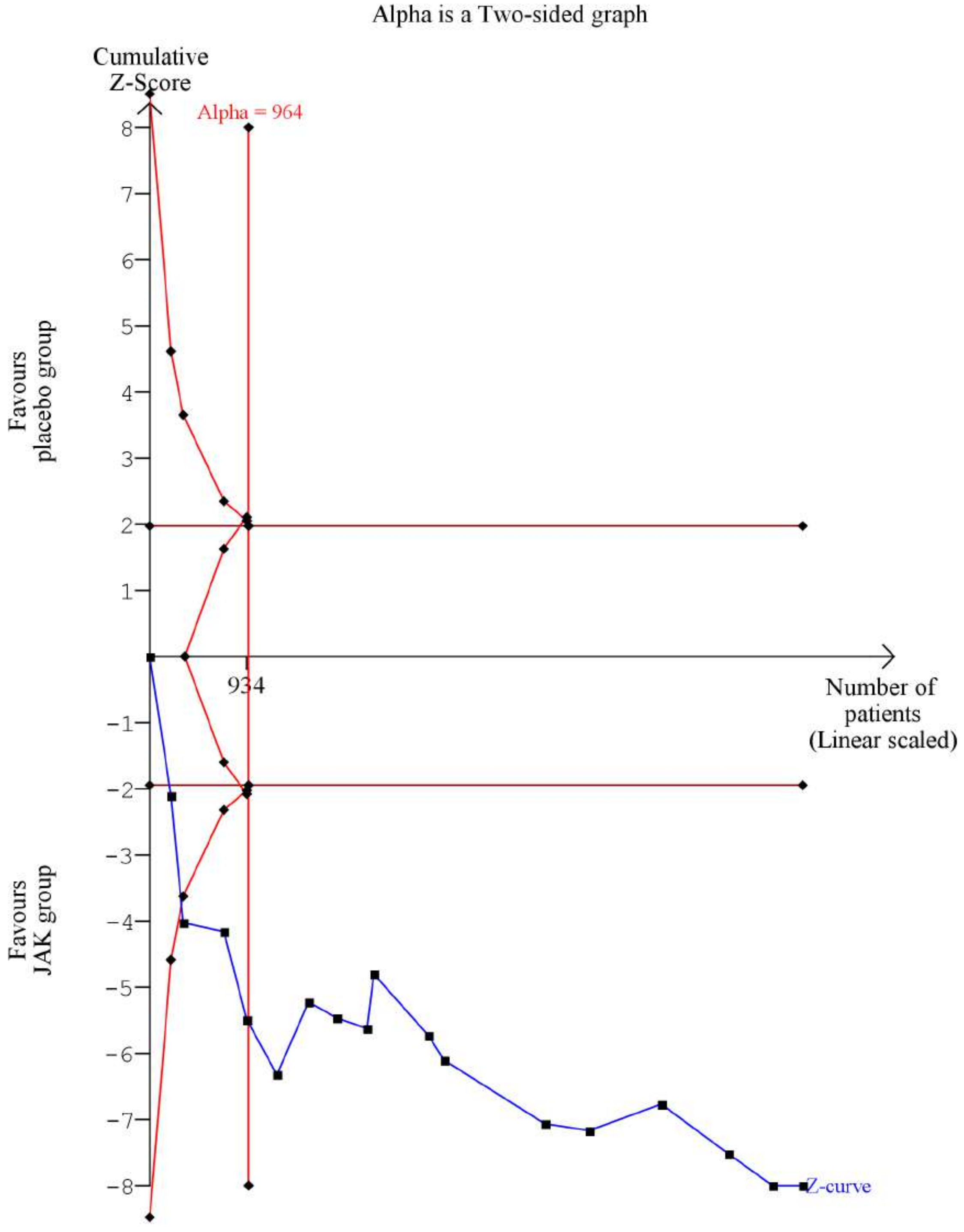

S109. TSA of HAQ-DI improvement (JAK inhibitors vs placebo)

The blue line (Z-curve) crosses the conventional boundary (brown straight line) and trial sequential boundary (red lines sloping inwards), and reaches the line of required sample size, therefore showing the statistically significant superiority of JAK inhibitors in this outcome and the addition of further studies would not influence these results.

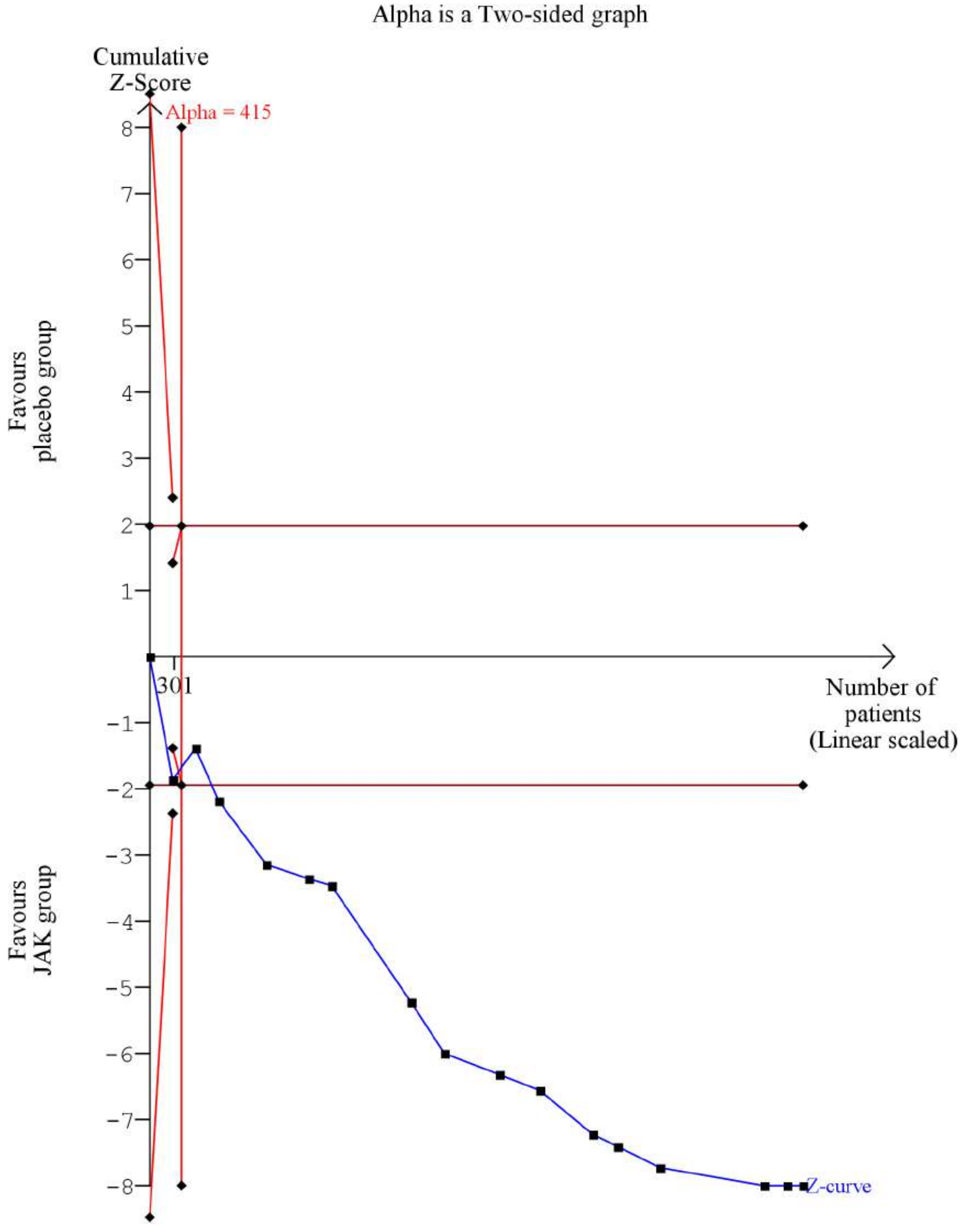

S110. TSA of CDAI remission (JAK inhibitors vs placebo)

The blue line (Z-curve) crosses the conventional boundary (brown straight line) and reaches the line of required sample size, therefore showing the statistically significant superiority of JAK inhibitors in this outcome and the addition of further studies would not influence these results.

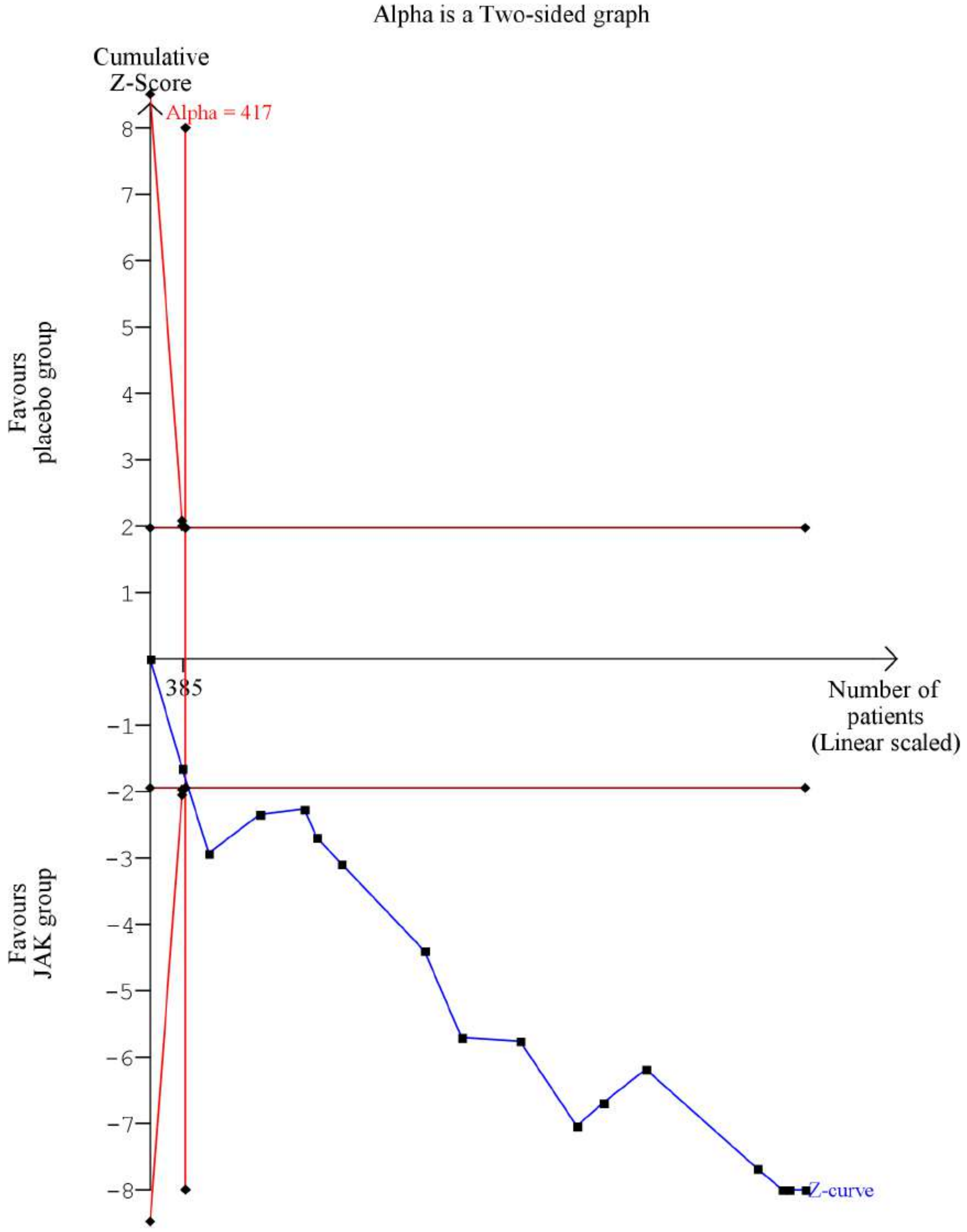

S111. TSA of SDAI remission (JAK inhibitors vs placebo)

The blue line (Z-curve) crosses the conventional boundary (brown straight line) and reaches the line of required sample size, therefore showing the statistically significant superiority of JAK inhibitors in this outcome and the addition of further studies would not influence these results.

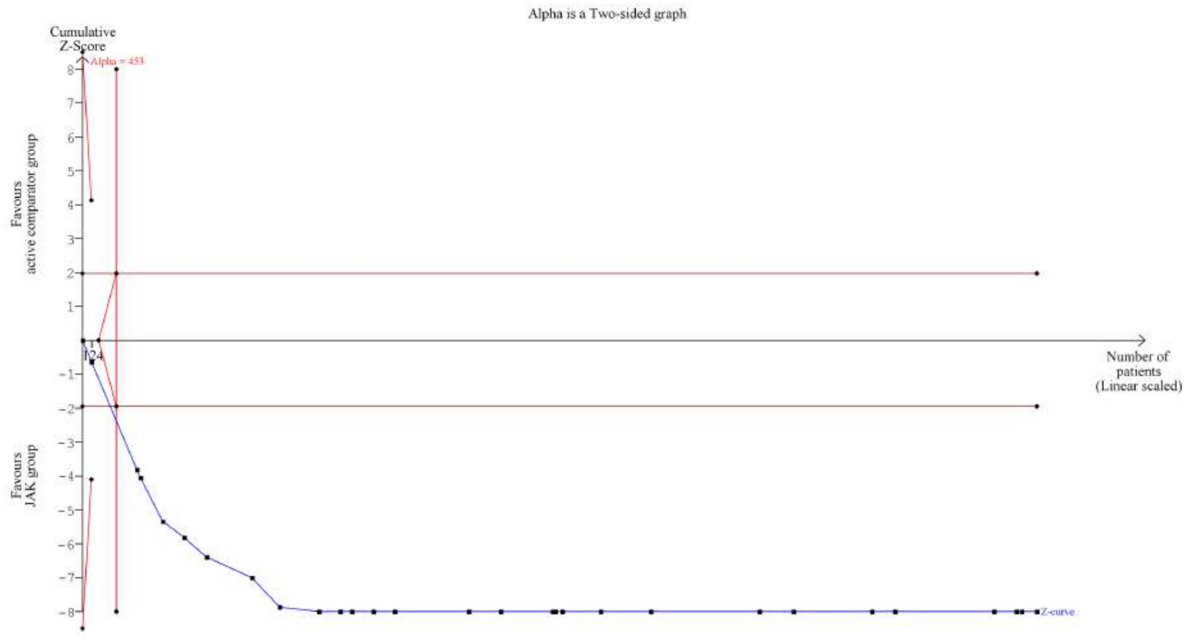

S112. TSA of DAS28-CRP remission (JAK inhibitors vs placebo)

The blue line (Z-curve) crosses the conventional boundary (brown straight line) and reaches the line of required sample size, therefore showing the statistically significant superiority of JAK inhibitors in this outcome and the addition of further studies would not influence these results.

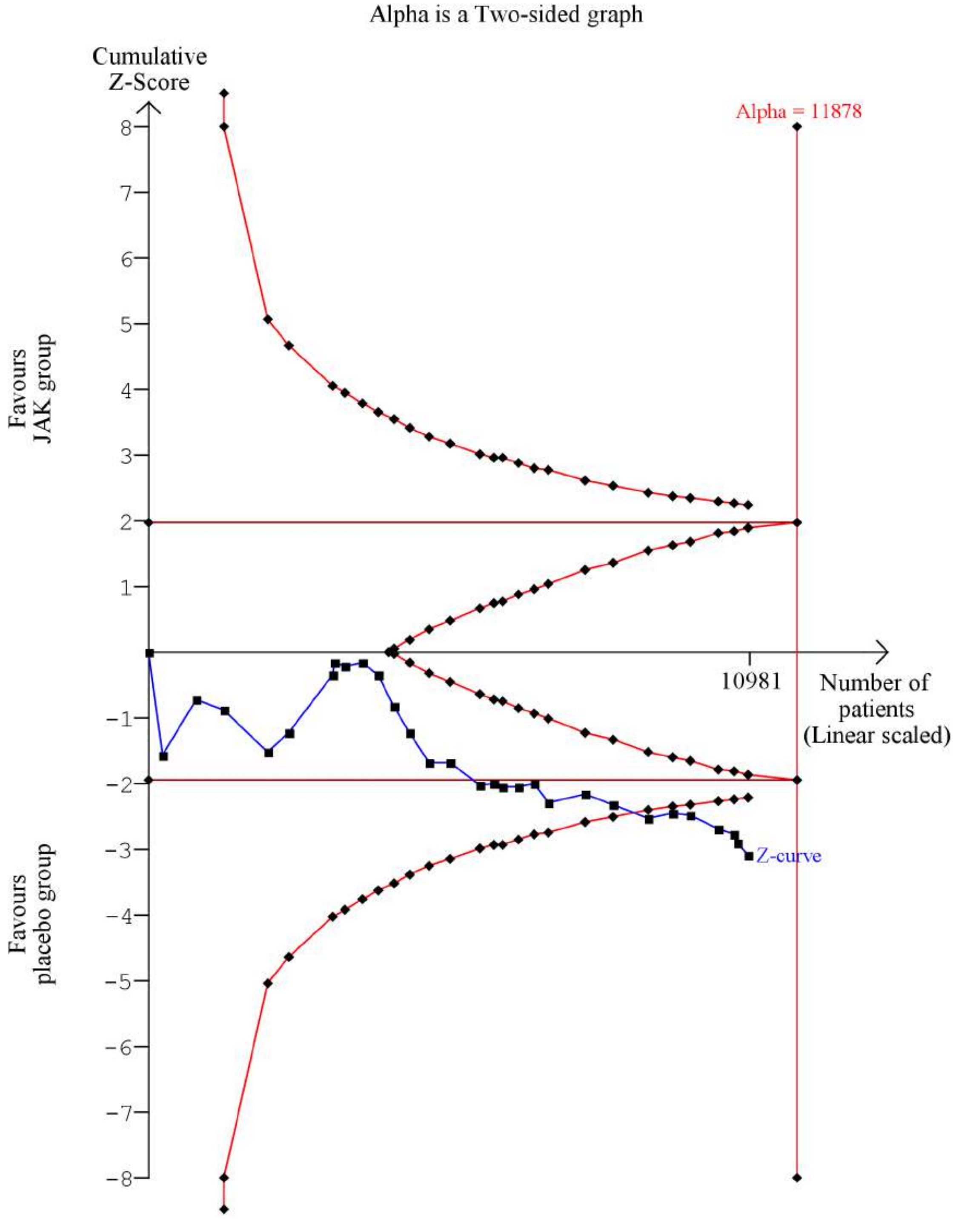

S113. TSA of side effects (JAK inhibitors vs placebo)

The blue line (Z-curve) crosses the conventional boundary (brown straight line) but does not reach the line of required sample size, therefore showing the statistically significant superiority of placebo in this outcome but the addition of further studies might influence these results.

Several studies ((2015)Boyle (2020)Robinson) could not be included due to low information content.

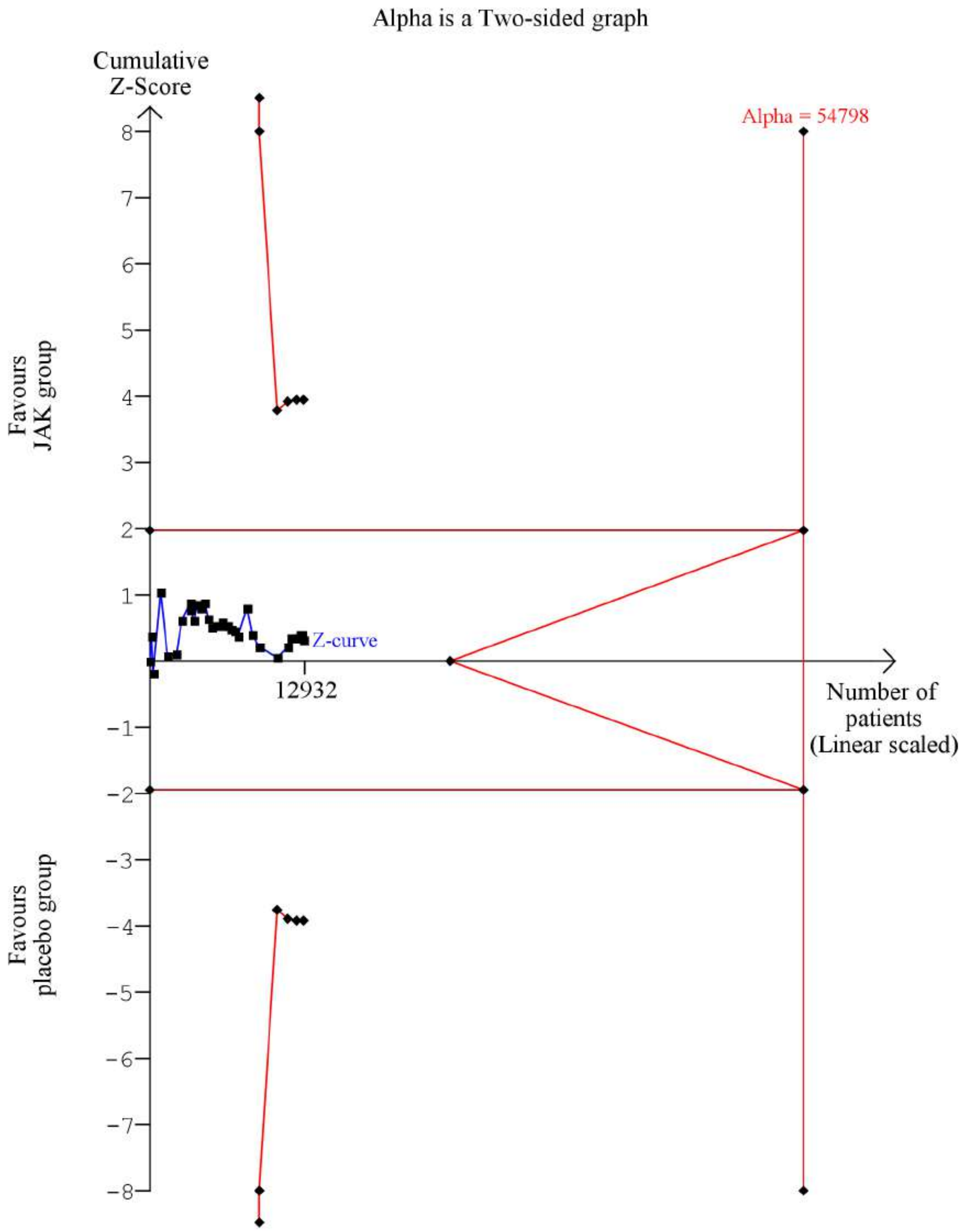

S114. TSA of serious side effects (JAK inhibitors vs placebo)

The blue line (Z-curve) does not cross the conventional boundary (brown straight line) and does not reach the line of required sample size, therefore showing no statistically significant

difference between groups in this outcome and the addition of further studies might influence these results. Several studies (2015)I4V-MC-JADA (2016)BALANCE I (2016)Genovese (2016)RA-BEACON (2016)Tanaka (2017)DARWIN2 (2017)RA-BEGIN (2018)SELECT-BEYOND (2019)RAJ3 (2020)RA-BALANCE (2020)Robinson) could not be included due to low information content.

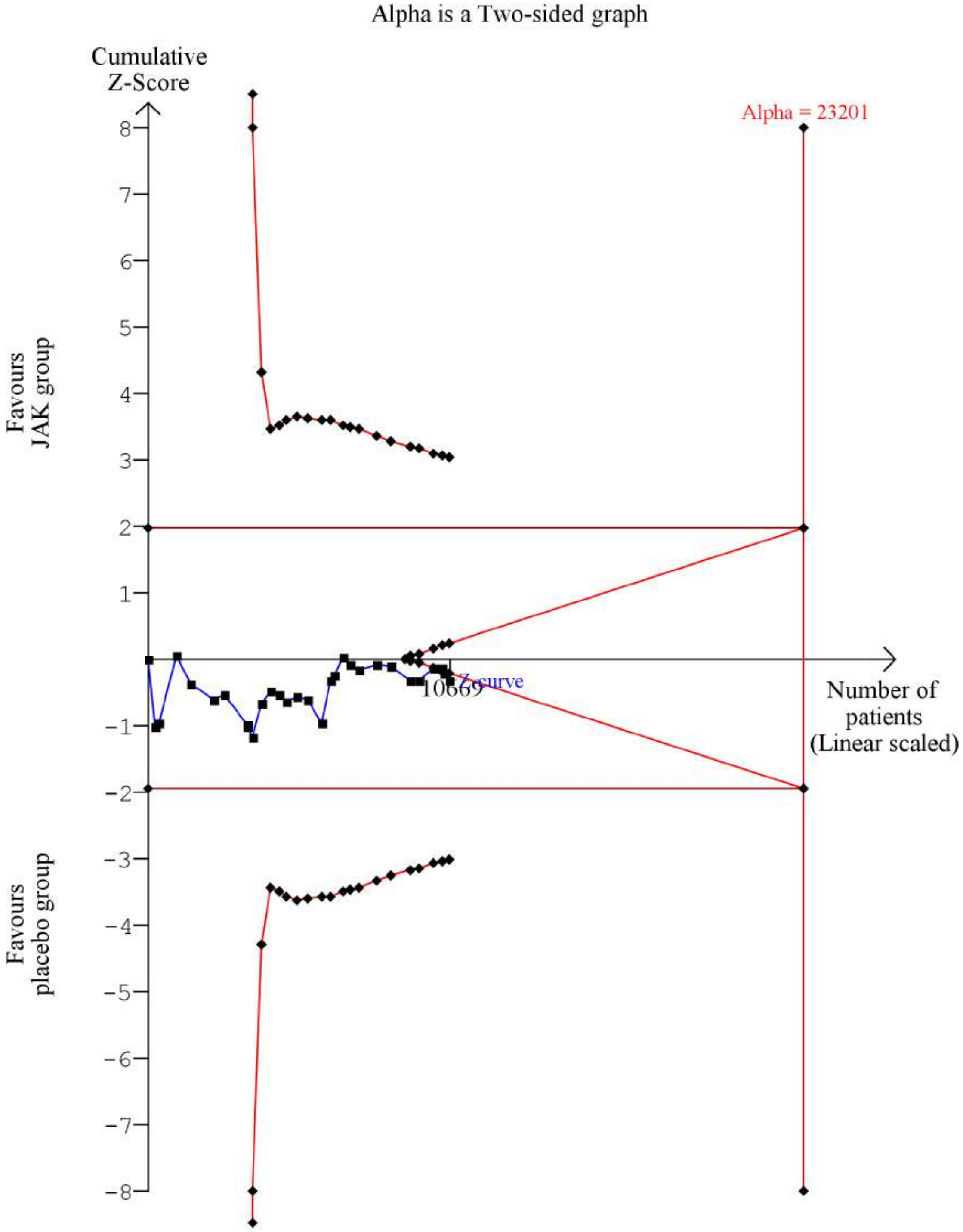

S115. TSA of discontinuation (JAK inhibitors vs placebo)

The blue line (Z-curve) does not cross the conventional boundary (brown straight line) and does not reach the line of required sample size, therefore showing no statistically significant difference between groups in this outcome and the addition of further studies might influence these results. Several studies ((2011)Tanaka, (2015)Boyle, (2016)Tanaka, (2020)Robinson) could not be included due to low information content.

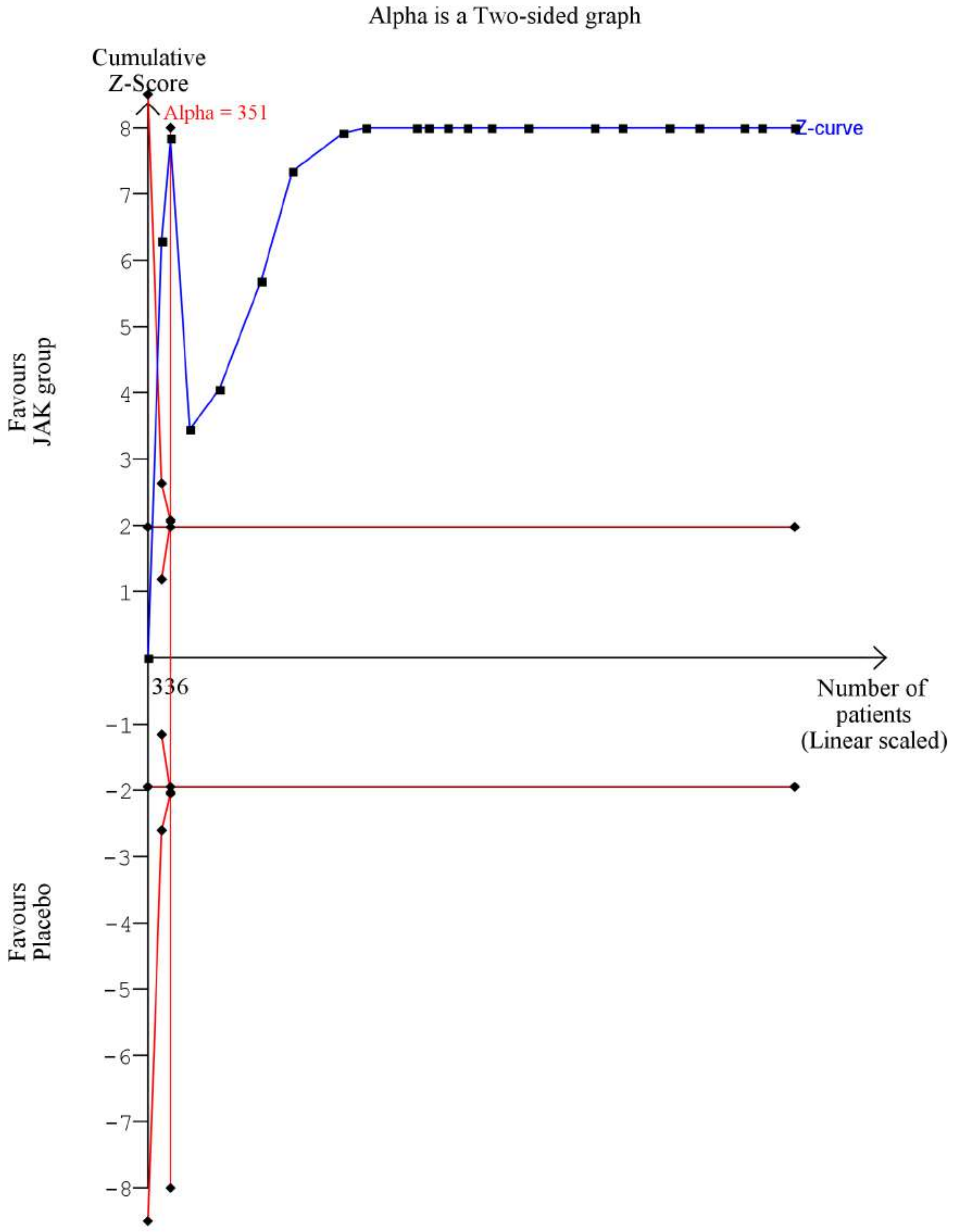

S116. TSA of Pain(VAS) (JAK inhibitors vs placebo)

The blue line (Z-curve) crosses the conventional boundary (brown straight line) and trial sequential boundary (red lines sloping inwards), and reaches the line of required sample size, therefore showing the statistically significant superiority of JAK inhibitors in this outcome and the addition of further studies would not influence these results.

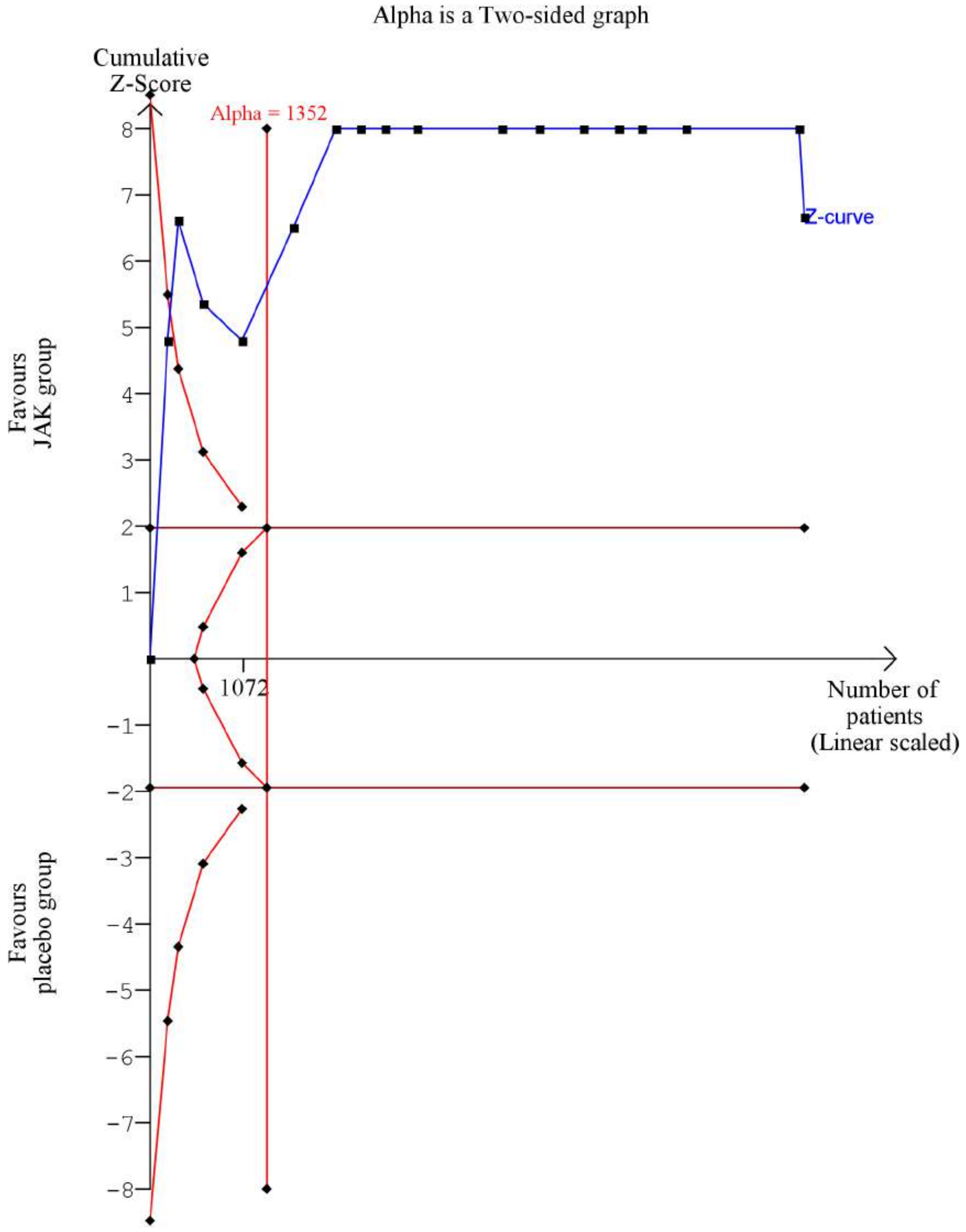

S117. TSA of PGA(VAS) (JAK inhibitors vs placebo)

The blue line (Z-curve) crosses the conventional boundary (brown straight line) and trial sequential boundary (red lines sloping inwards), and reaches the line of required sample size, therefore showing the statistically significant superiority of JAK inhibitors in this outcome and the addition of further studies would not influence these results.

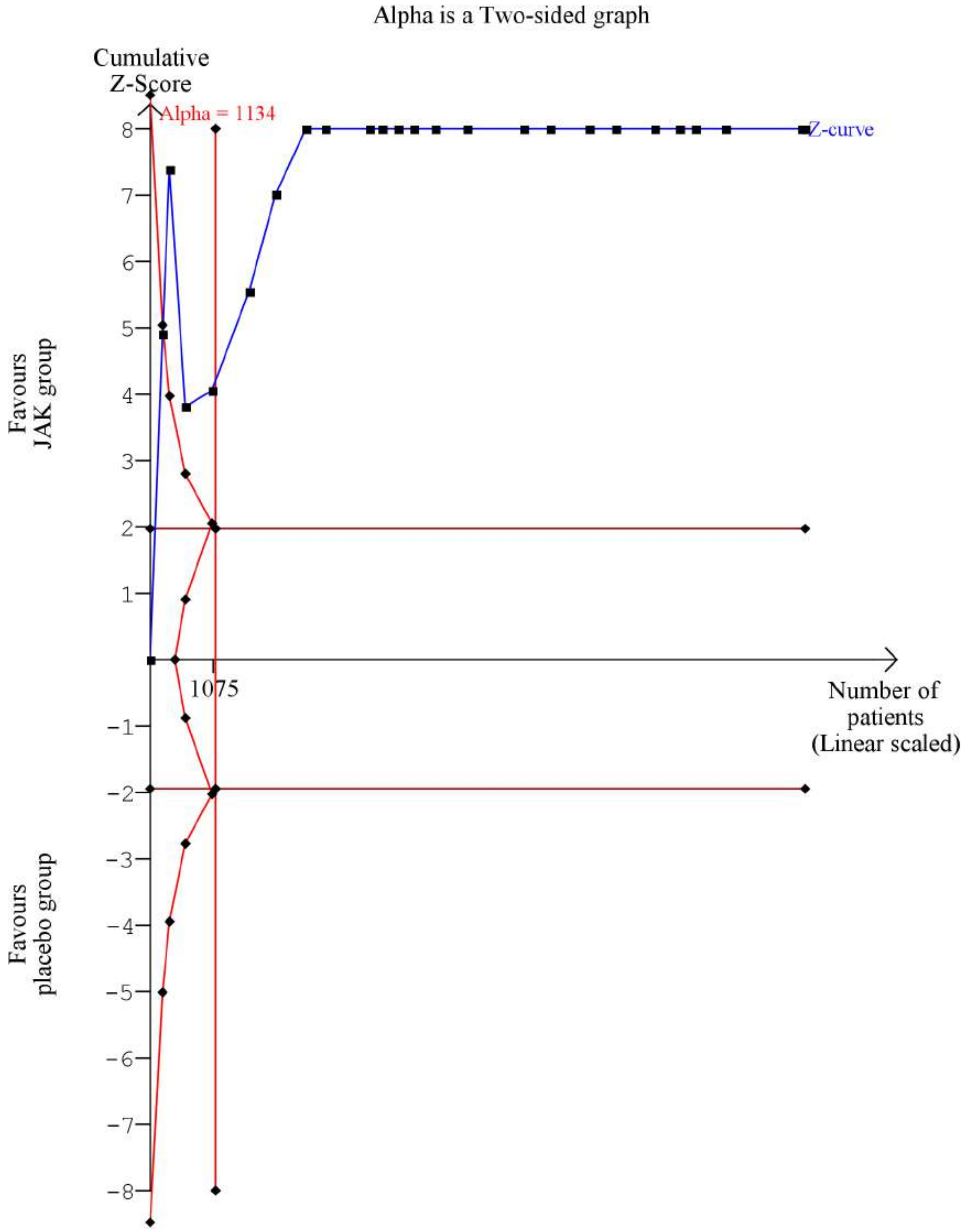

S118. TSA of PtGA(VAS) (JAK inhibitors vs placebo)

The blue line (Z-curve) crosses the conventional boundary (brown straight line) and trial sequential boundary (red lines sloping inwards), and reaches the line of required sample size, therefore showing the statistically significant superiority of JAK inhibitors in this outcome and the addition of further studies would not influence these results.

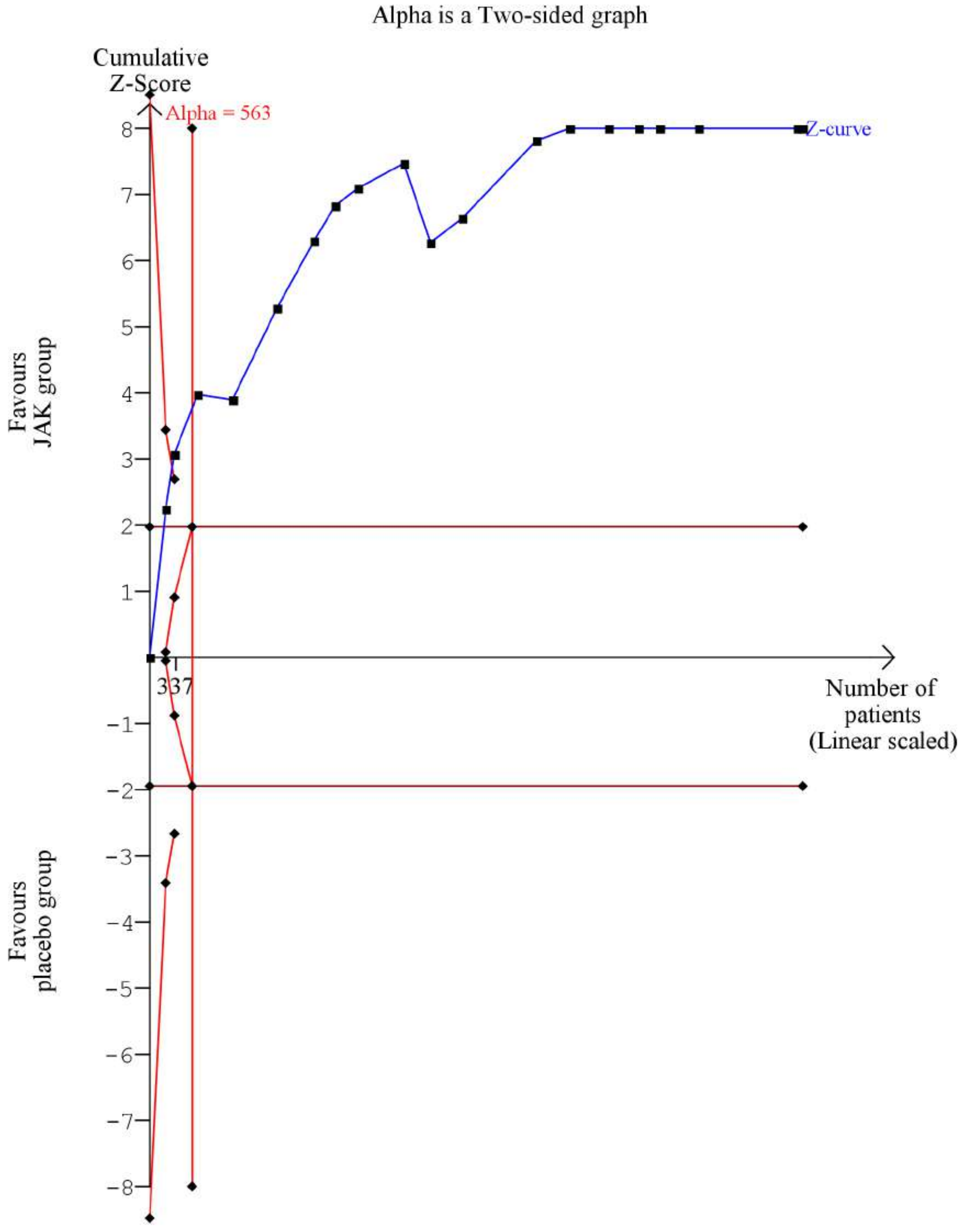

S119. TSA of Swollen Joint Count (JAK inhibitors vs placebo)

The blue line (Z-curve) crosses the conventional boundary (brown straight line) and trial sequential boundary (red lines sloping inwards), and reaches the line of required sample size, therefore showing the statistically significant superiority of JAK inhibitors in this outcome and the addition of further studies would not influence these results.

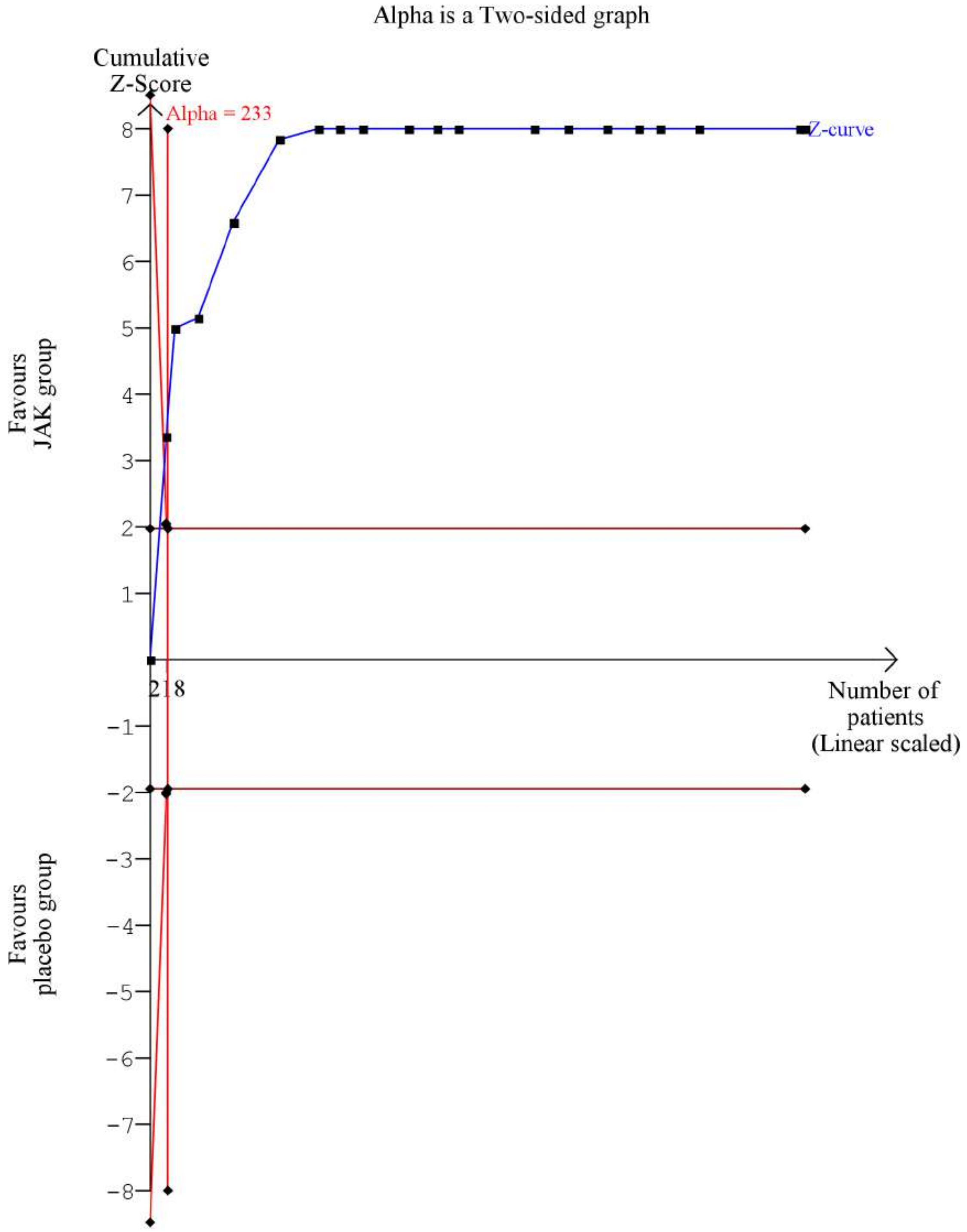

S120. TSA of Tender Joint Count (JAK inhibitors vs placebo)

The blue line (Z-curve) crosses the conventional boundary (brown straight line) and trial sequential boundary (red lines sloping inwards), and reaches the line of required sample size, therefore showing the statistically significant superiority of JAK inhibitors in this outcome and the addition of further studies would not influence these results.

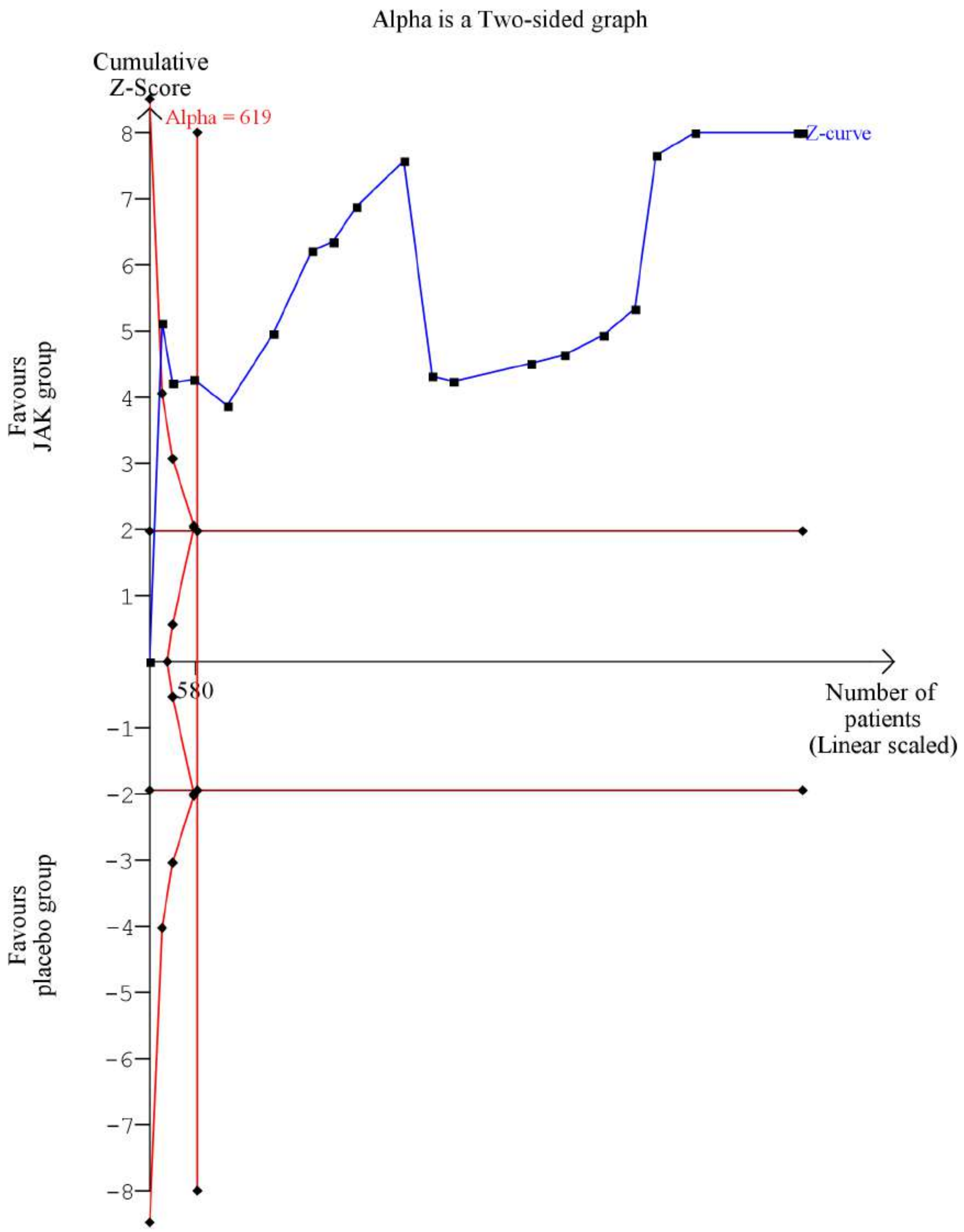

S121. TSA of CRP (mg/l) (JAK inhibitors vs placebo)

The blue line (Z-curve) crosses the conventional boundary (brown straight line) and trial sequential boundary (red lines sloping inwards), and reaches the line of required sample size, therefore showing the statistically significant superiority of JAK inhibitors in this outcome and the addition of further studies would not influence these results.

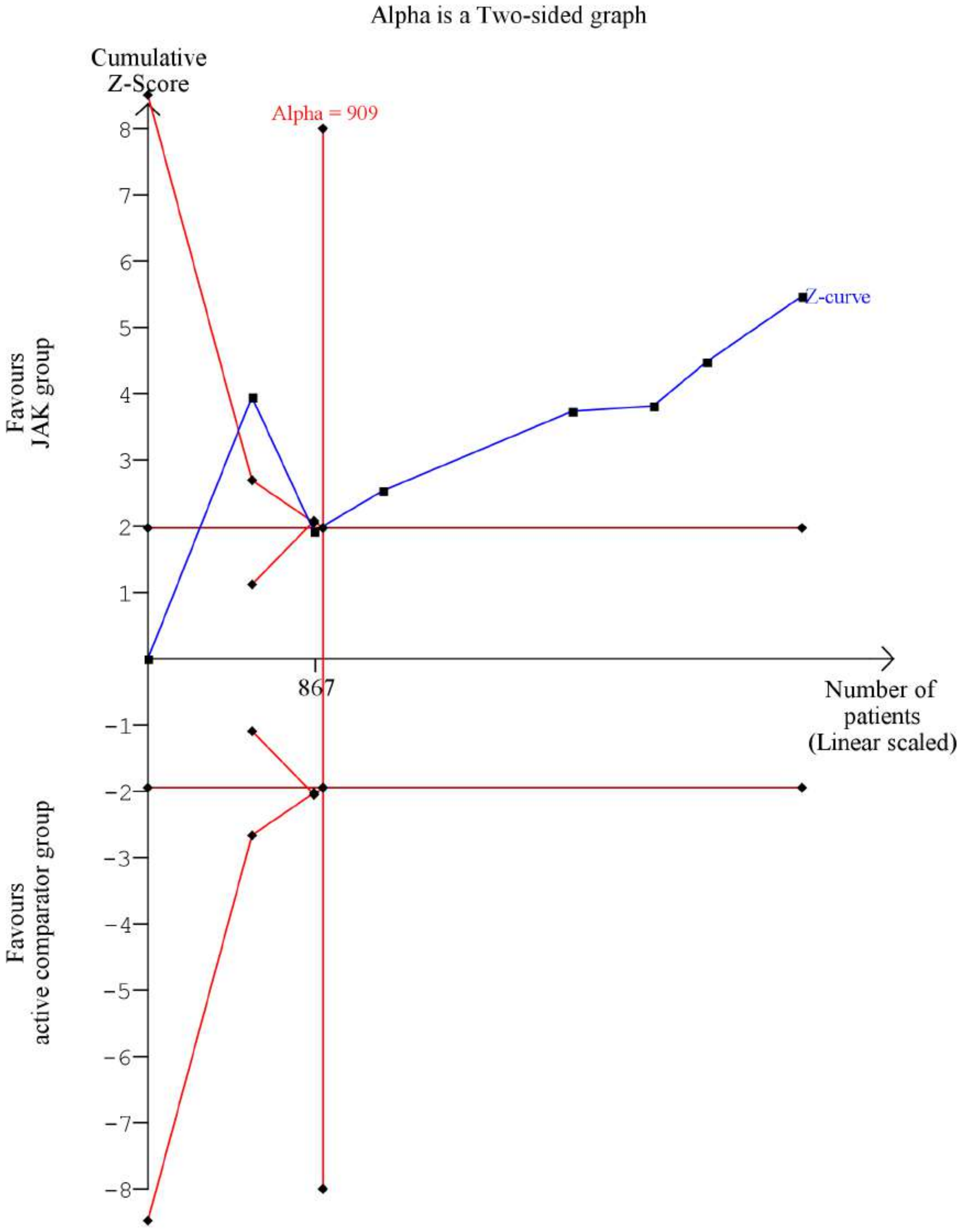

S122. TSA of ESR (mm/hour) (JAK inhibitors vs placebo)

The blue line (Z-curve) crosses the conventional boundary (brown straight line) and trial sequential boundary (red lines sloping inwards), and reaches the line of required sample size, therefore showing the statistically significant superiority of JAK inhibitors in this outcome and the addition of further studies would not influence these results.

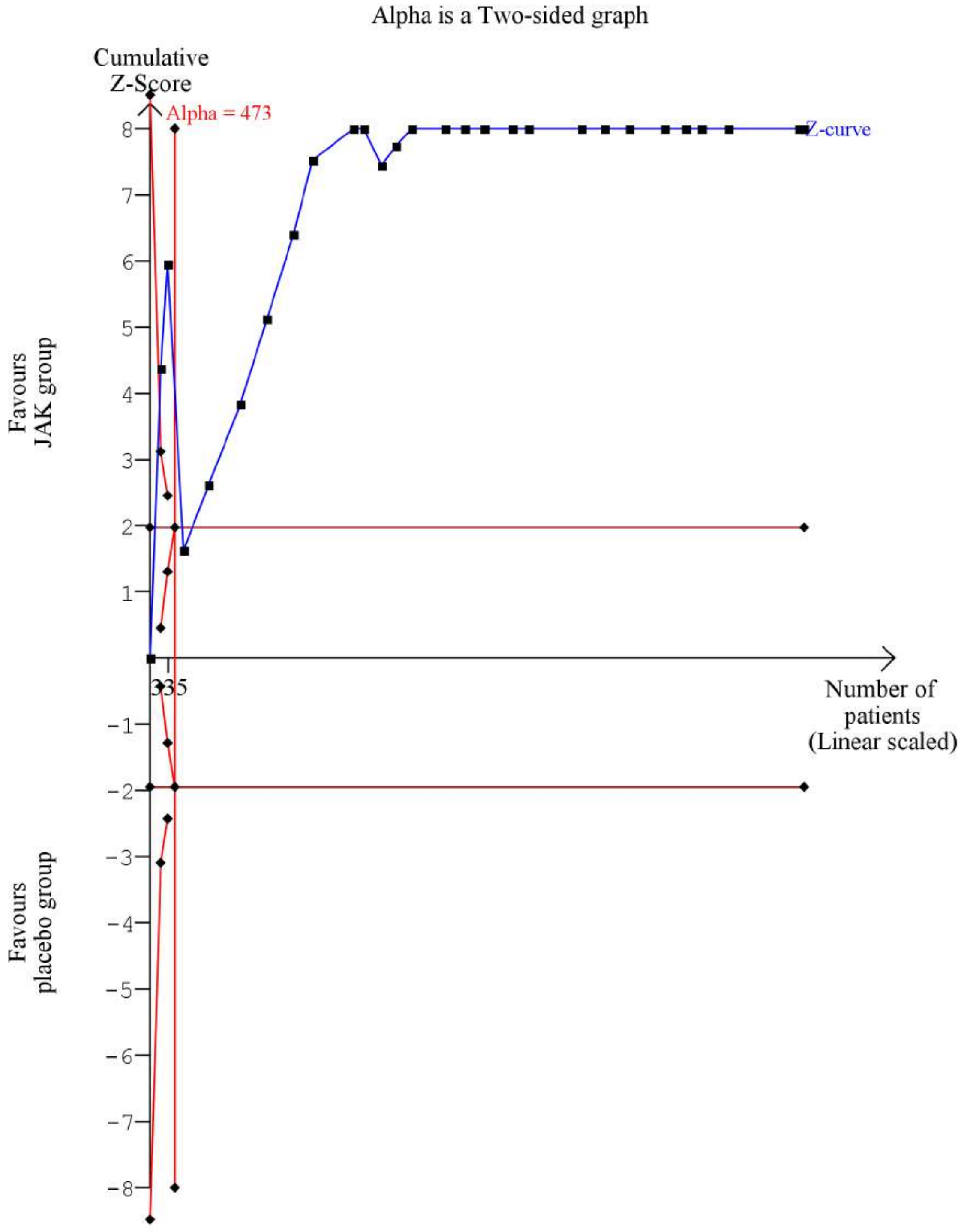

S123. TSA of HAQ-DI difference (JAK inhibitors vs placebo)

The blue line (Z-curve) crosses the conventional boundary (brown straight line) and trial sequential boundary (red lines sloping inwards), and reaches the line of required sample size, therefore showing the statistically significant superiority of JAK inhibitors in this outcome and the addition of further studies would not influence these results.

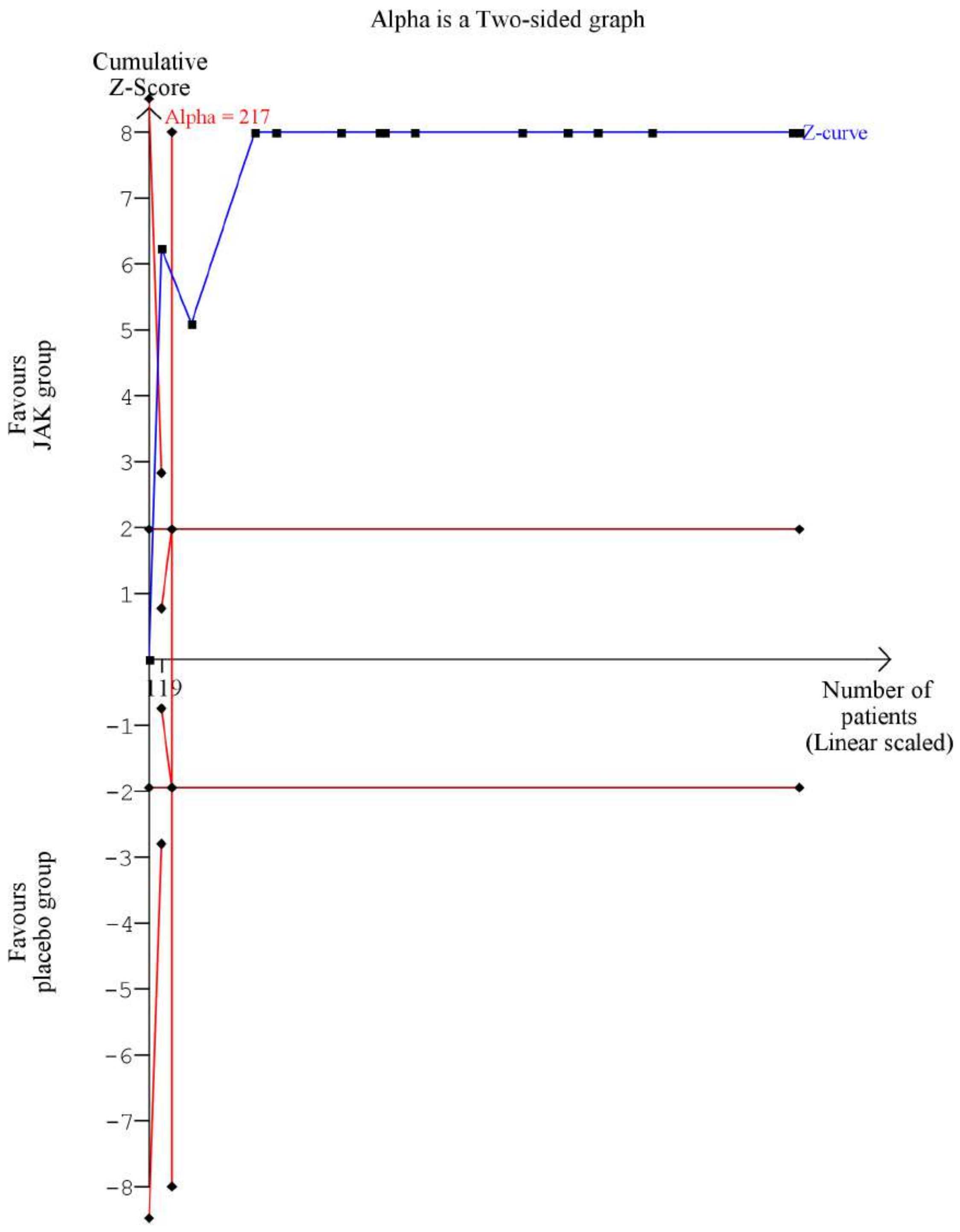

S124. TSA of DAS28-CRP difference (JAK inhibitors vs placebo)

The blue line (Z-curve) crosses the conventional boundary (brown straight line) and trial sequential boundary (red lines sloping inwards), and reaches the line of required sample size, therefore showing the statistically significant superiority of JAK inhibitors in outcome and the addition of further studies would not influence these results.

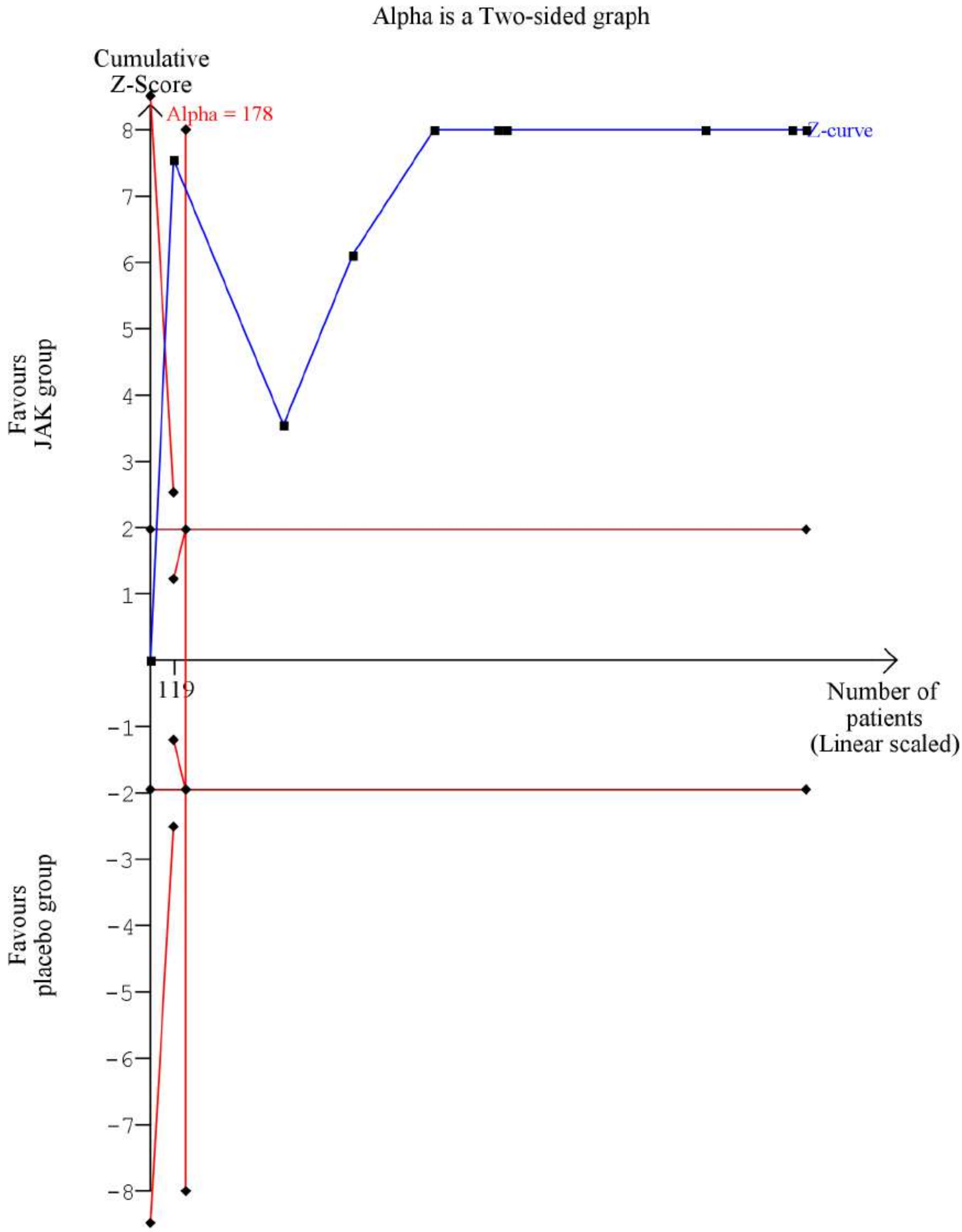

S125. TSA of DAS28-ESR difference (JAK inhibitors vs placebo)

The blue line (Z-curve) crosses the conventional boundary (brown straight line) and trial sequential boundary (red lines sloping inwards), and reaches the line of required sample size, therefore showing the statistically significant superiority of JAK inhibitors in outcome and the addition of further studies would not influence these results.

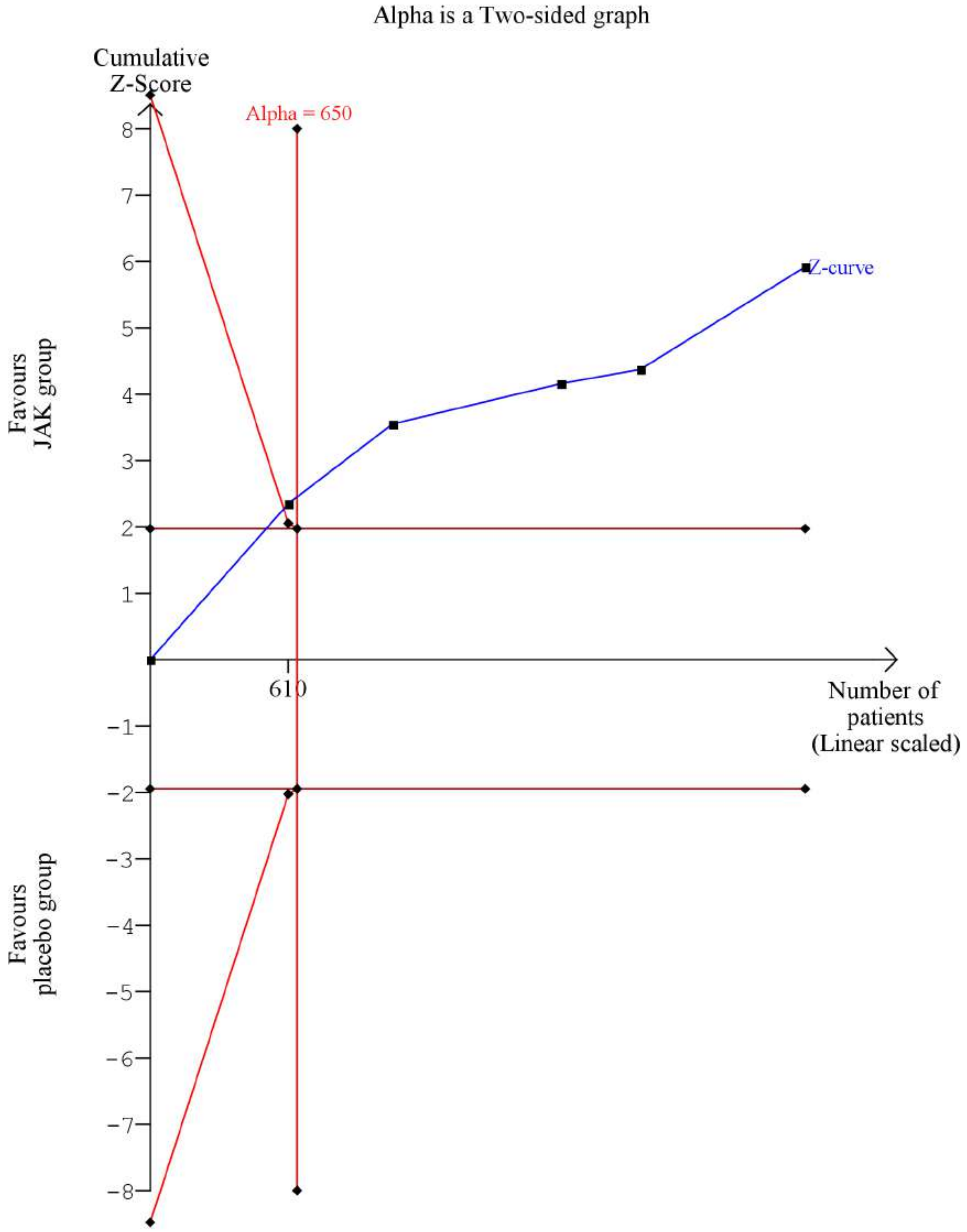

S126. TSA of MOS-Sleep (JAK inhibitors vs placebo)

The blue line (Z-curve) crosses the conventional boundary (brown straight line) and trial sequential boundary (red lines sloping inwards), and reaches the line of required sample size, therefore showing the statistically significant superiority of JAK inhibitors in outcome and the addition of further studies would not influence these results.

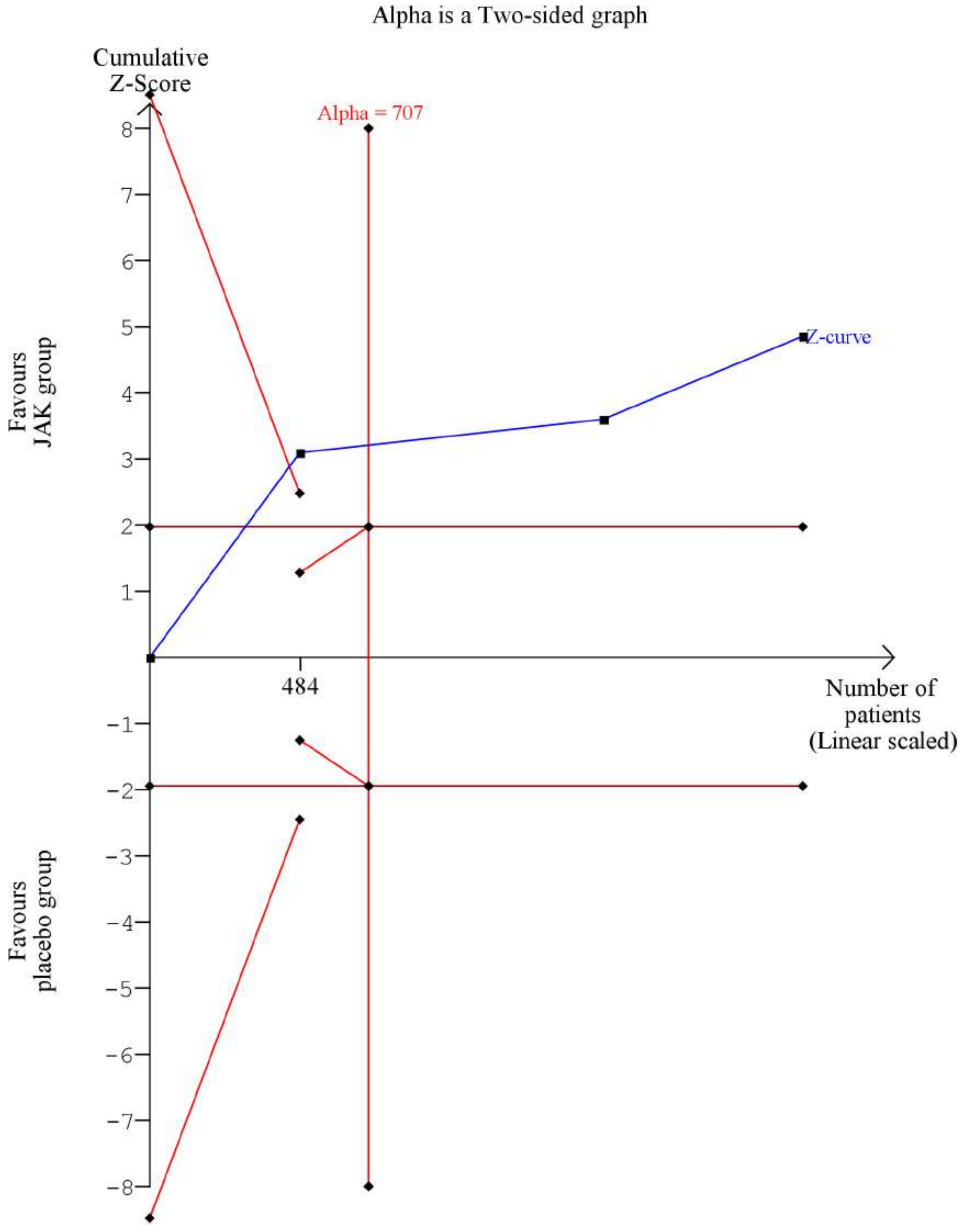

S127. TSA of WPAI AI (JAK inhibitors vs placebo)

The blue line (Z-curve) crosses the conventional boundary (brown straight line) and trial sequential boundary (red lines sloping inwards), and reaches the line of required sample size, therefore showing the statistically significant superiority of JAK inhibitors in outcome and the addition of further studies would not influence these results.

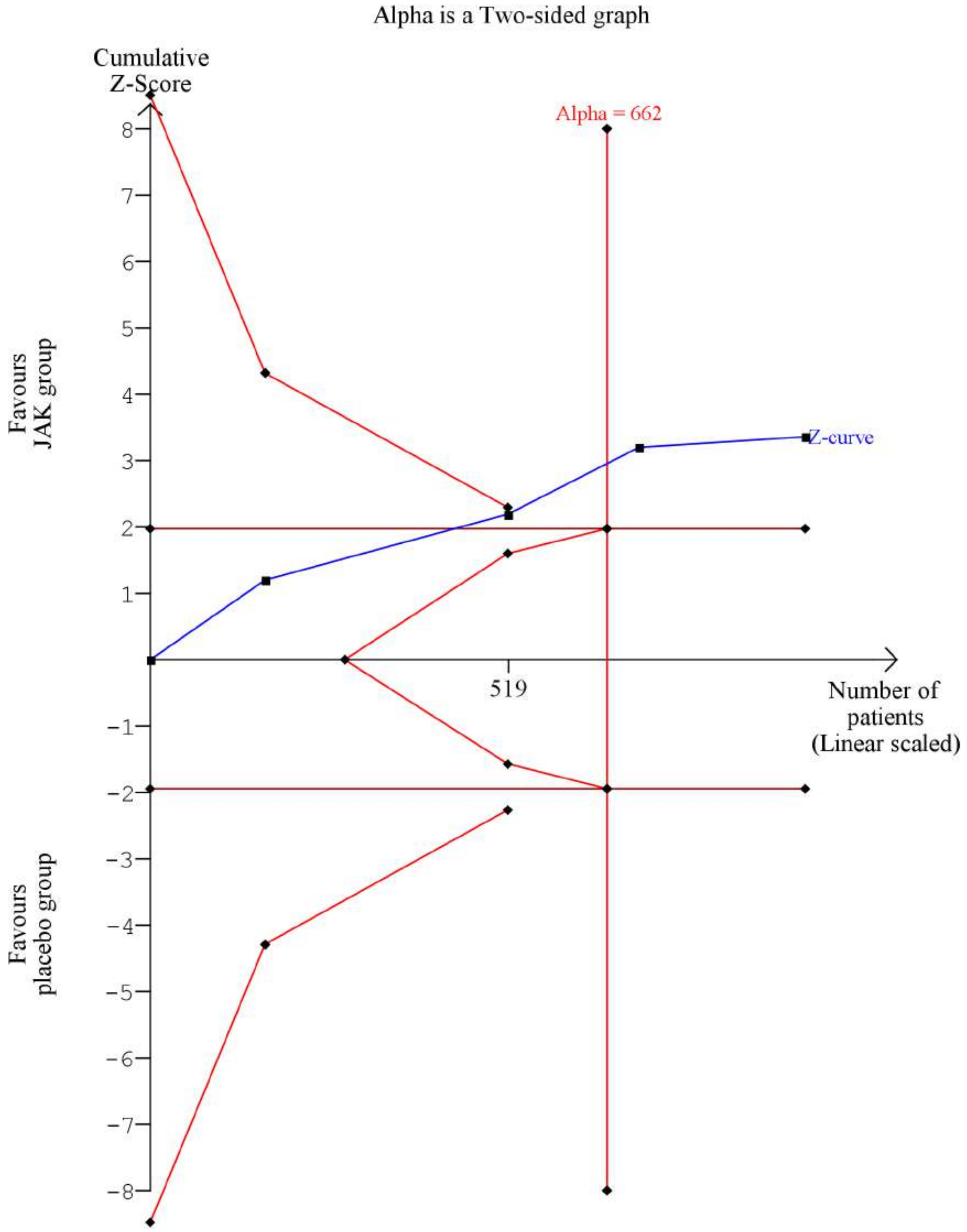

S128. TSA of WPAI A (JAK inhibitors vs placebo)

The blue line (Z-curve) crosses the conventional boundary (brown straight line) and trial sequential boundary (red lines sloping inwards), and reaches the line of required sample size, therefore showing the statistically significant superiority of JAK inhibitors in outcome and the addition of further studies would not influence these results.

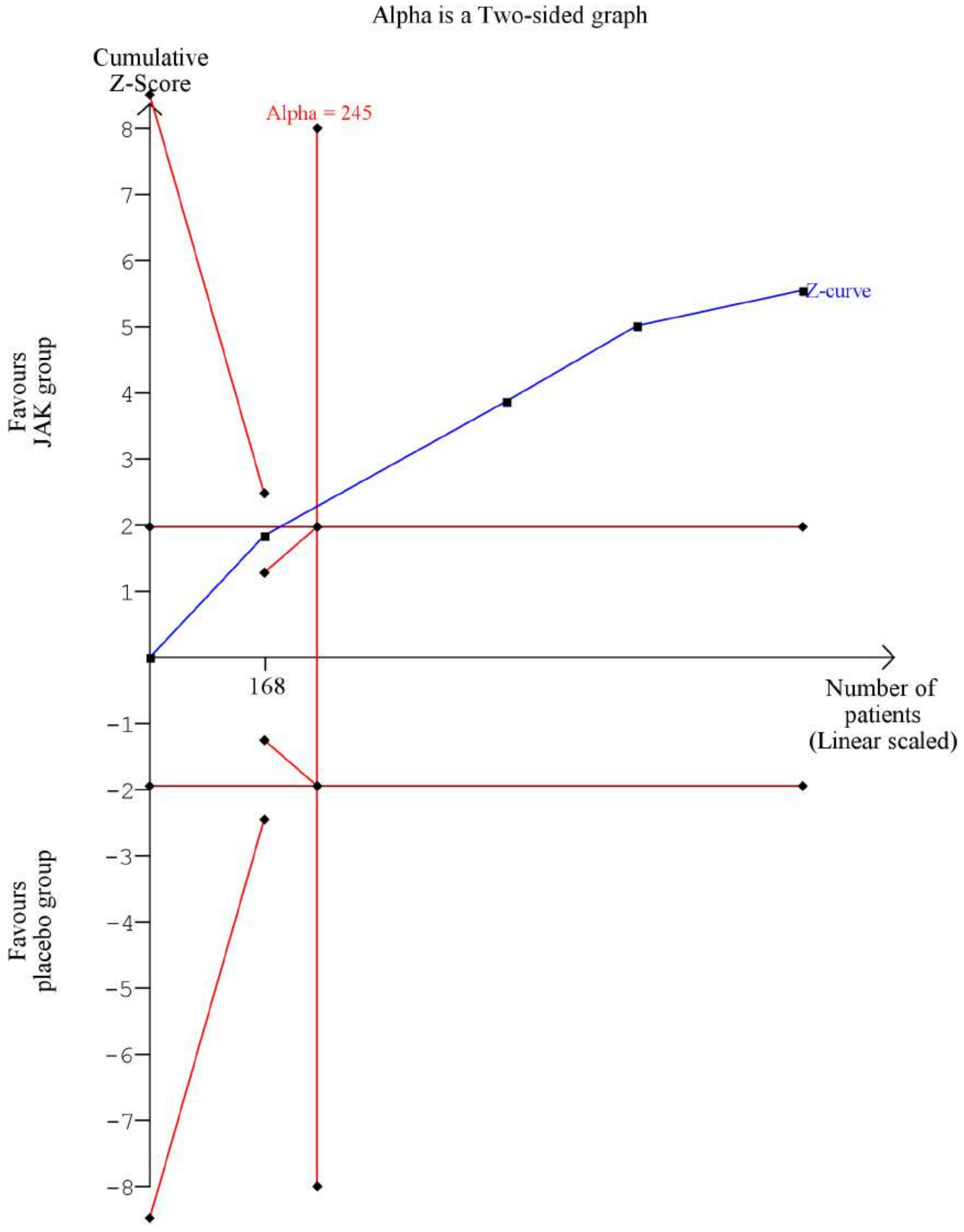

S129. TSA of WPAI OWI (JAK inhibitors vs placebo)

The blue line (Z-curve) crosses the conventional boundary (brown straight line) and reaches the line of required sample size, therefore showing the statistically significant superiority of JAK inhibitors in this outcome and the addition of further studies would not influence these results.

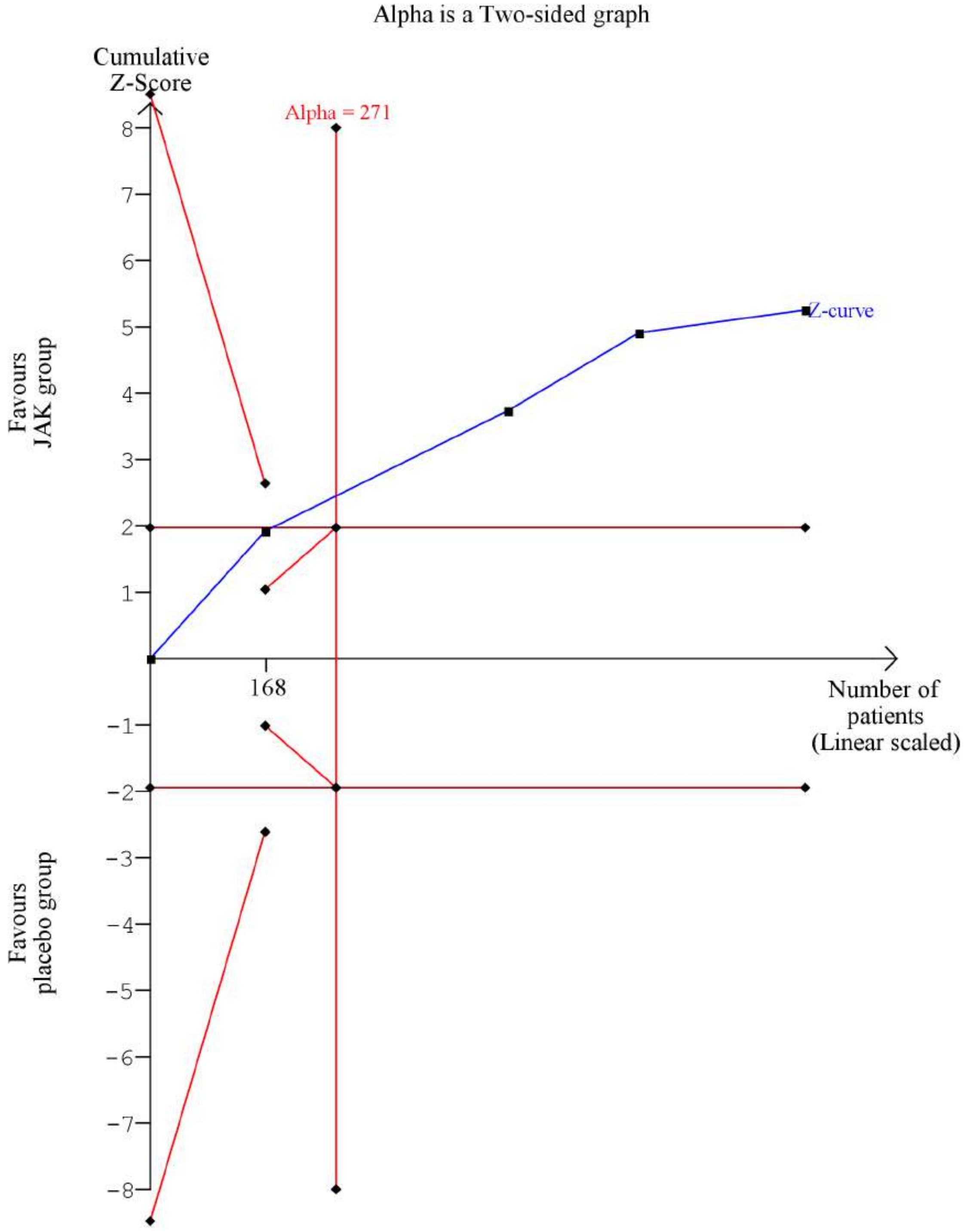

S130. TSA of WPAI P (JAK inhibitors vs placebo)

The blue line (Z-curve) crosses the conventional boundary (brown straight line) and reaches the line of required sample size, therefore showing the statistically significant superiority of JAK inhibitors in this outcome and the addition of further studies would not influence these results.

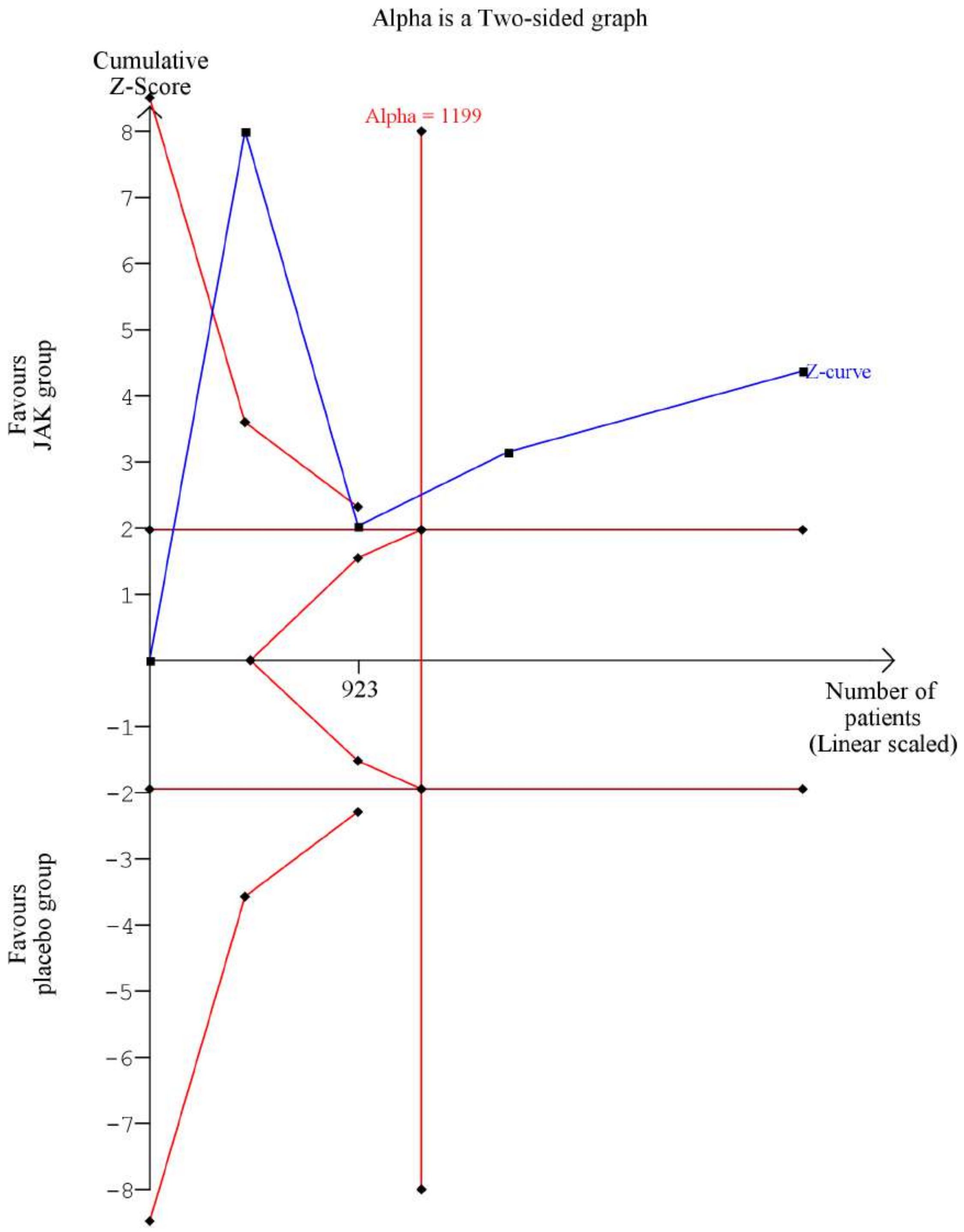

S131. TSA of MJS duration (JAK inhibitors vs placebo)

The blue line (Z-curve) crosses the conventional boundary (brown straight line) and trial sequential boundary (red lines sloping inwards), and reaches the line of required sample size,

therefore showing the statistically significant superiority of JAK inhibitors in outcome and the addition of further studies would not influence these results.

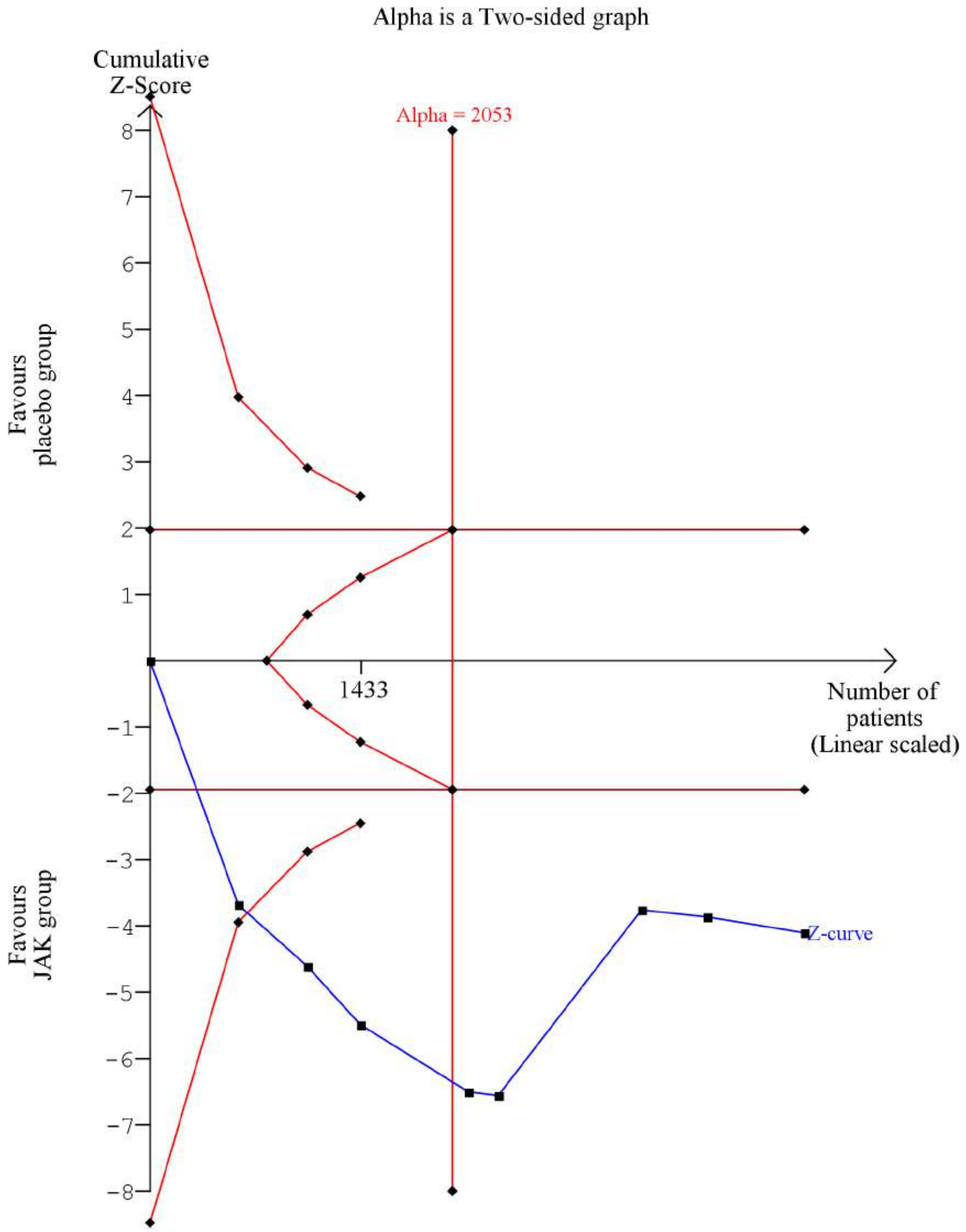

S132. TSA of SF-36 MCS (JAK inhibitors vs placebo)

The blue line (Z-curve) crosses the conventional boundary (brown straight line) and trial sequential boundary (red lines sloping inwards), and reaches the line of required sample size,

therefore showing the statistically significant superiority of JAK inhibitors in outcome and the addition of further studies would not influence these results.

(2) JAK inhibitors in monotherapy compared to MTX

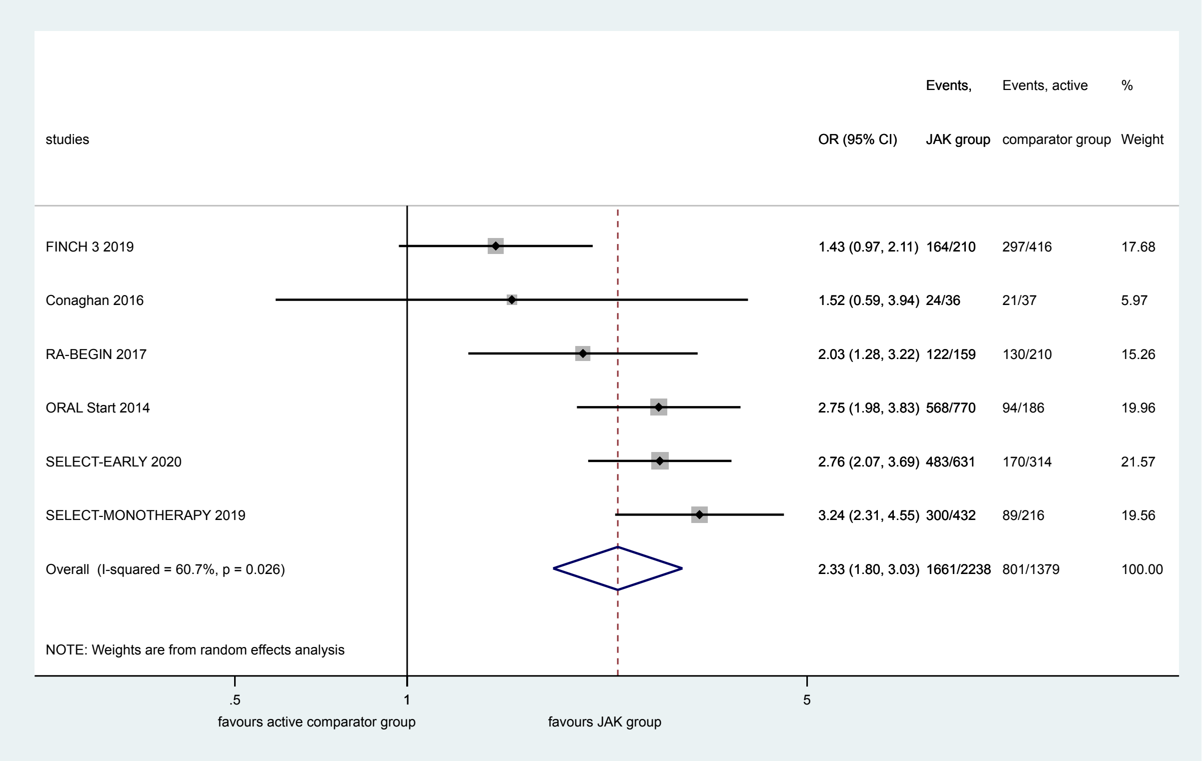

S133. Forest plot of studies comparing the number of patients reaching at least 20% in ACR criteria between patients treated with JAK inhibitors and MTX within 6 months (ACR20 response)

Black diamonds exhibit the individual effect of each study, whereas outer lines show the confidence intervals (CI) and the size of grey squares are proportional to the weight of each study. The blue diamond demonstrates the overall effect, the edges of which represent the CIs.

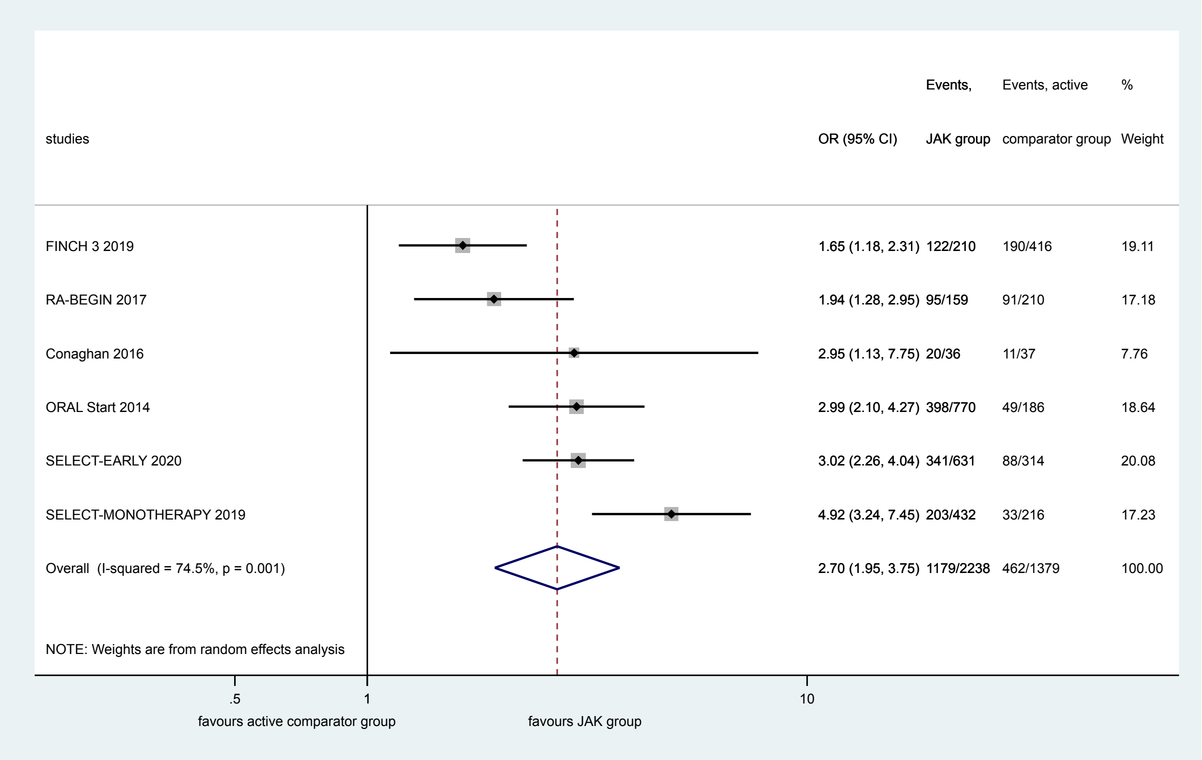

S134 a. Forest plot of studies comparing the number of patients reaching at least 50% in ACR criteria between patients treated with JAK inhibitors and MTX within 6 months (ACR50 response)

Black diamonds exhibit the individual effect of each study, whereas outer lines show the confidence intervals (CI) and the size of grey squares are proportional to the weight of each study. The blue diamond demonstrates the overall effect, the edges of which represent the CIs.

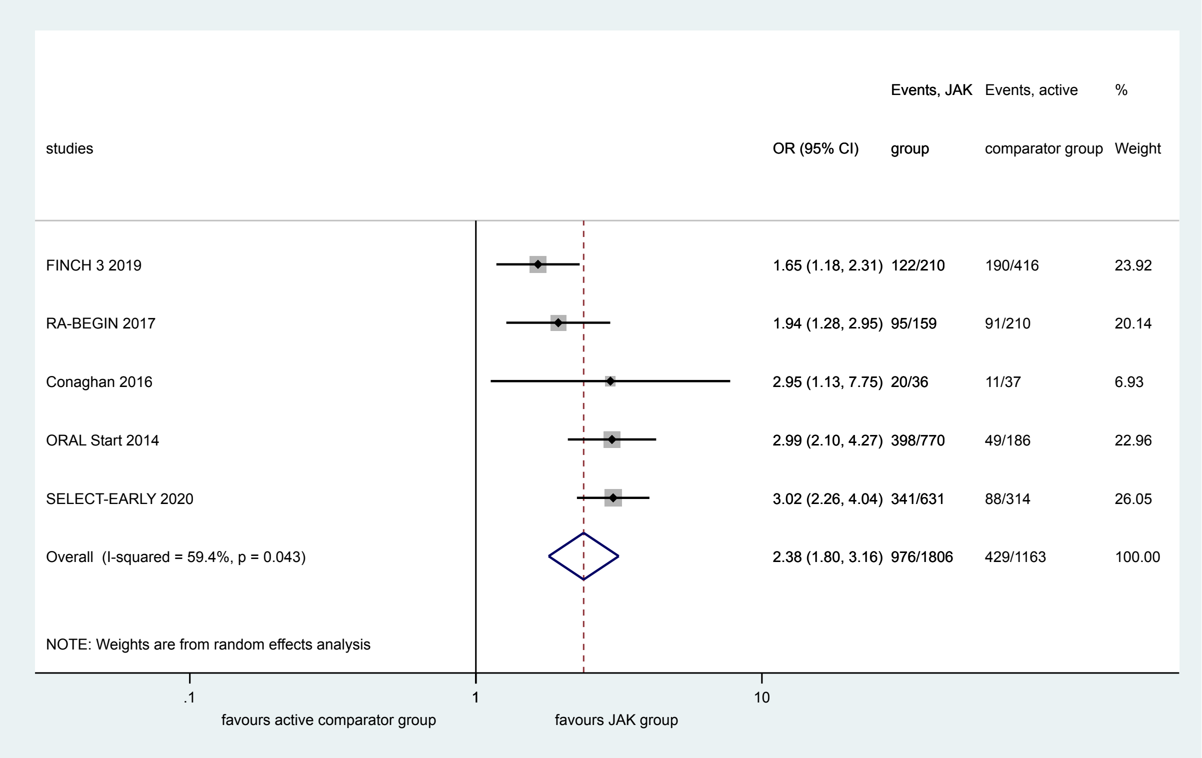

S134 b. Forest plot of studies comparing the number of patients reaching at least 50% in ACR criteria between patients treated with JAK inhibitors and MTX within 6 months (ACR50 response) – Secondary analysis only involving studies conducted in a MTX naïve population

Black diamonds exhibit the individual effect of each study, whereas outer lines show the confidence intervals (CI) and the size of grey squares are proportional to the weight of each study. The blue diamond demonstrates the overall effect, the edges of which represent the CIs.

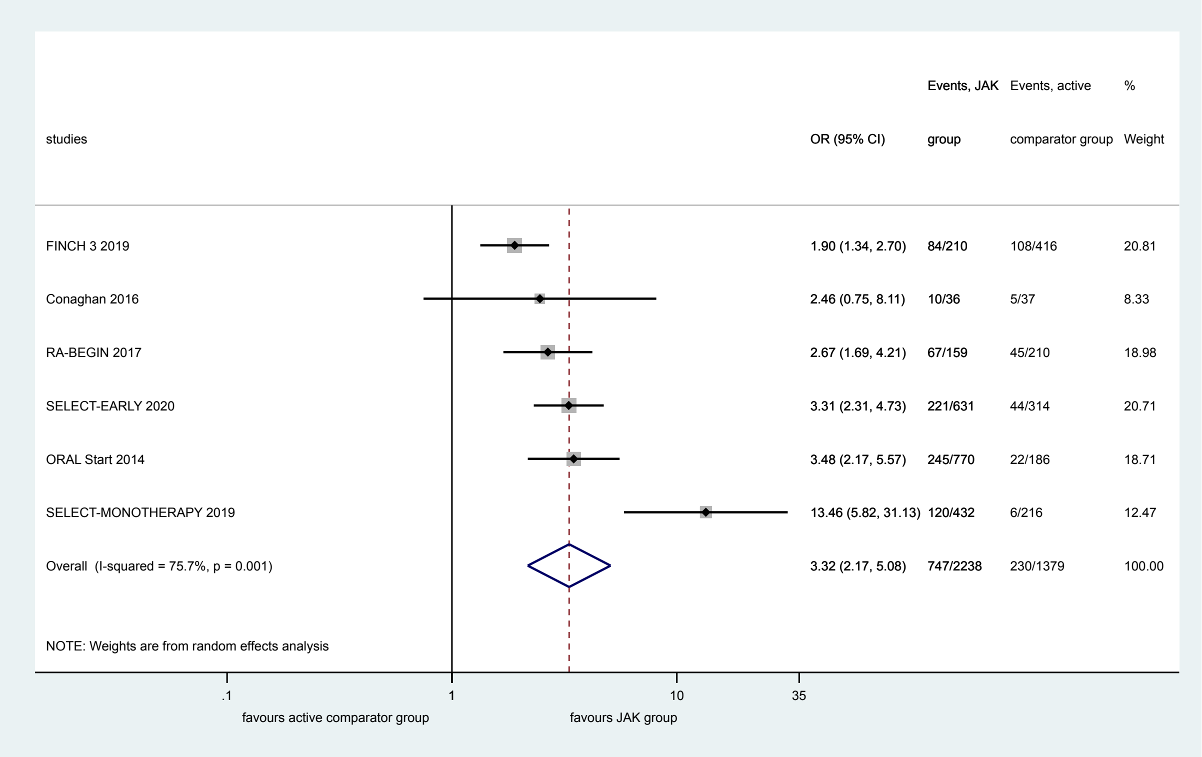

S135 a. Forest plot of studies comparing the number of patients reaching at least 70% in ACR criteria between patients treated with JAK inhibitors and MTX within 6 months (ACR70 response)

Black diamonds exhibit the individual effect of each study, whereas outer lines show the confidence intervals (CI) and the size of grey squares are proportional to the weight of each study. The blue diamond demonstrates the overall effect, the edges of which represent the CIs.

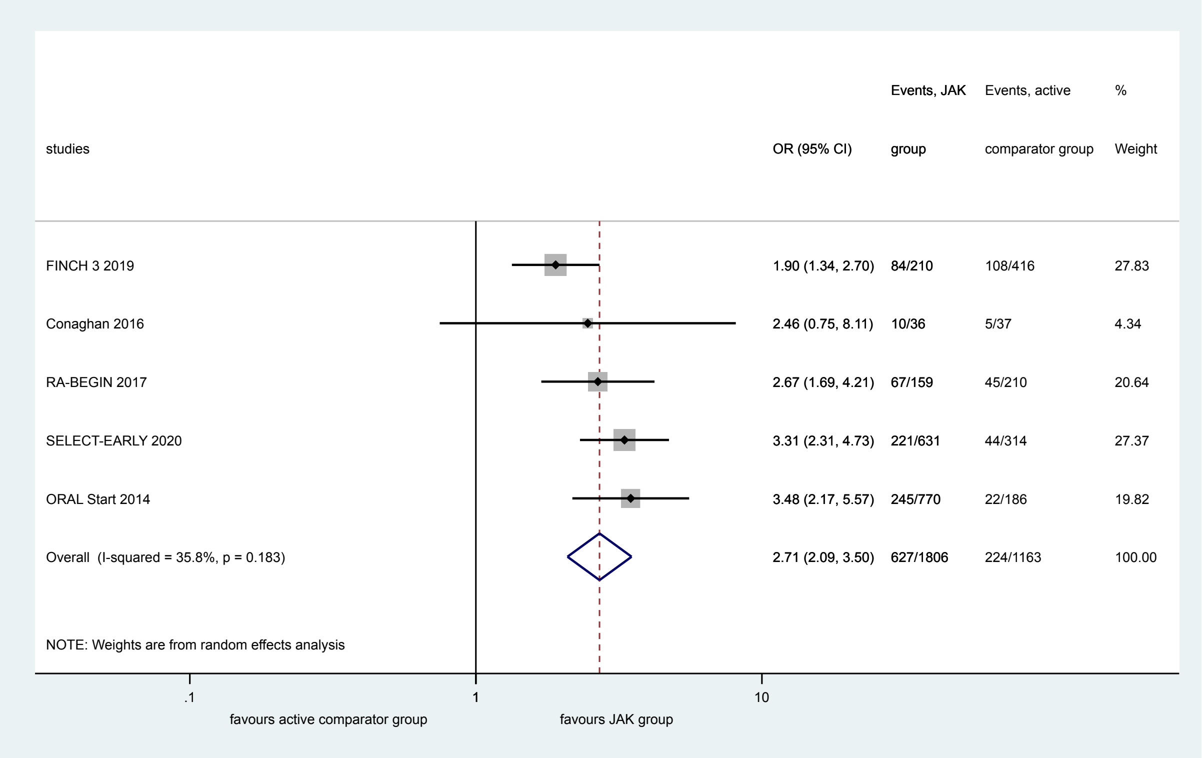

S135 b. Forest plot of studies comparing the number of patients reaching at least 70% in ACR criteria between patients treated with JAK inhibitors and MTX within 6 months (ACR70 response) - Secondary analysis only involving studies conducted in a MTX naïve population

Black diamonds exhibit the individual effect of each study, whereas outer lines show the confidence intervals (CI) and the size of grey squares are proportional to the weight of each study. The blue diamond demonstrates the overall effect, the edges of which represent the CIs.

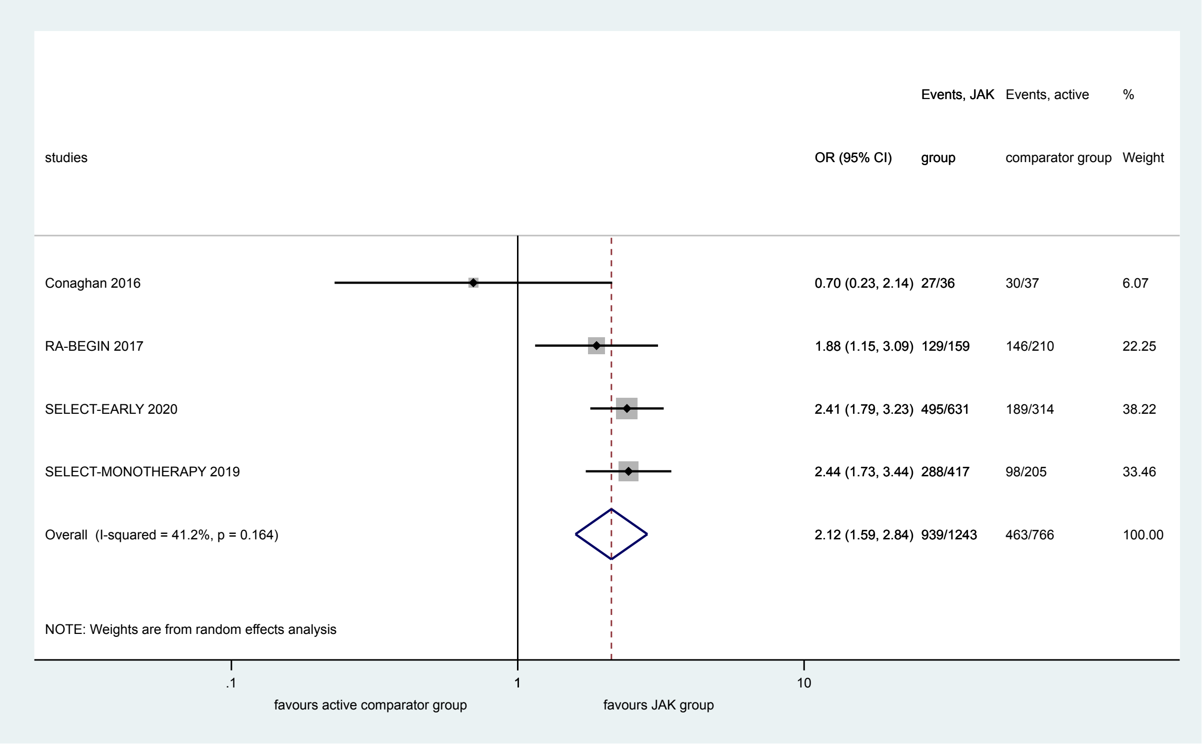

S136. Forest plot of studies comparing the number of patients reaching at least the minimum clinically important difference (≥0.22 MCID) in HAQ-DI between patients treated with JAK inhibitors and placebo within 6 months (HAQ-DI improvement)

Black diamonds exhibit the individual effect of each study, whereas outer lines show the confidence intervals (CI) and the size of grey squares are proportional to the weight of each study. The blue diamond demonstrates the overall effect, the edges of which represent the CIs.

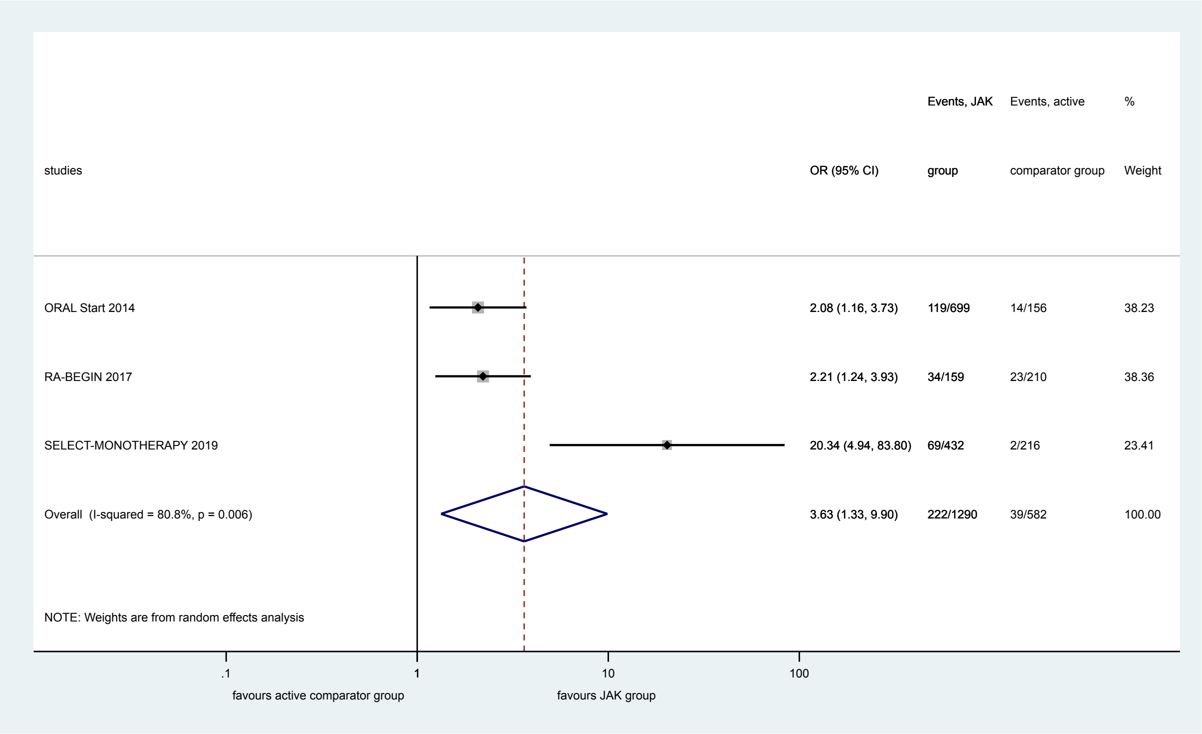

S137. Forest plot of studies comparing the number of patients reaching remission defined by CDAI (≤ 2.8) between patients treated with JAK inhibitors and MTX within 6 months (CDAI remission)

Black diamonds exhibit the individual effect of each study, whereas outer lines show the confidence intervals (CI) and the size of grey squares are proportional to the weight of each study. The blue diamond demonstrates the overall effect, the edges of which represent the CIs.

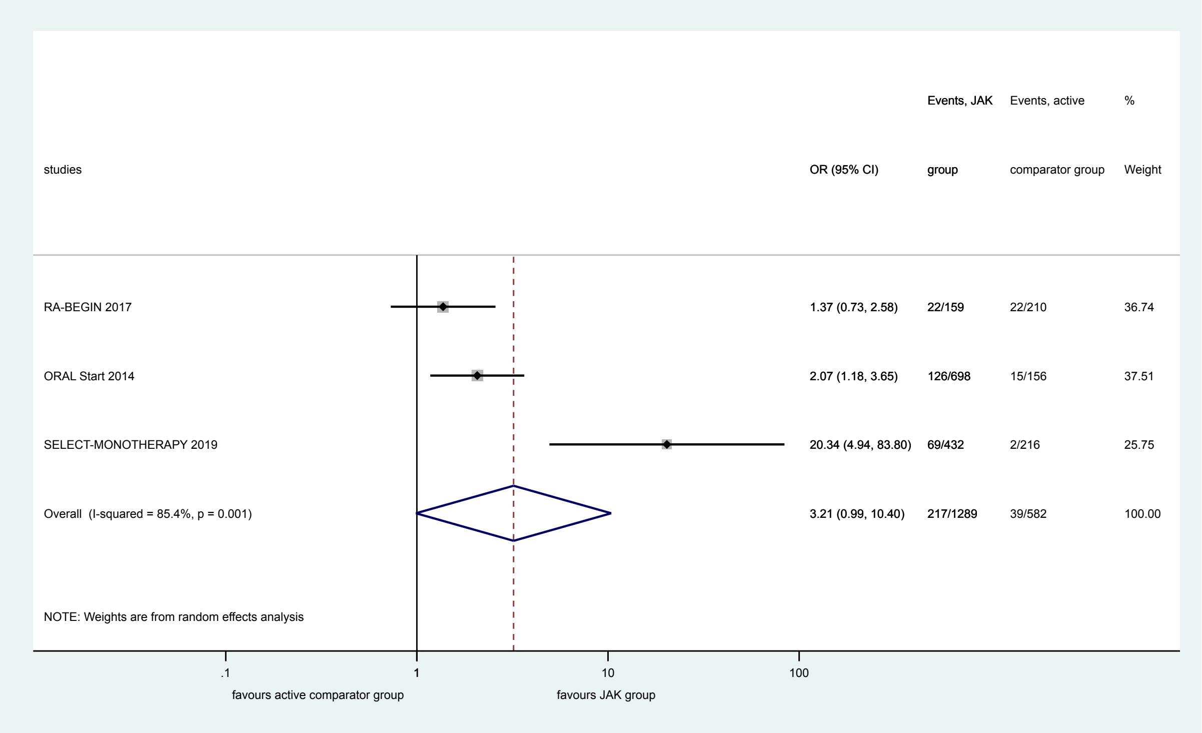

S138.Forest plot of studies comparing the number of patients reaching remission defined by SDAI (≤ 3.3) between patients treated with JAK inhibitors and MTX within 6 months (SDAI remission)

Black diamonds exhibit the individual effect of each study, whereas outer lines show the confidence intervals (CI) and the size of grey squares are proportional to the weight of each study. The blue diamond demonstrates the overall effect, the edges of which represent the CIs.

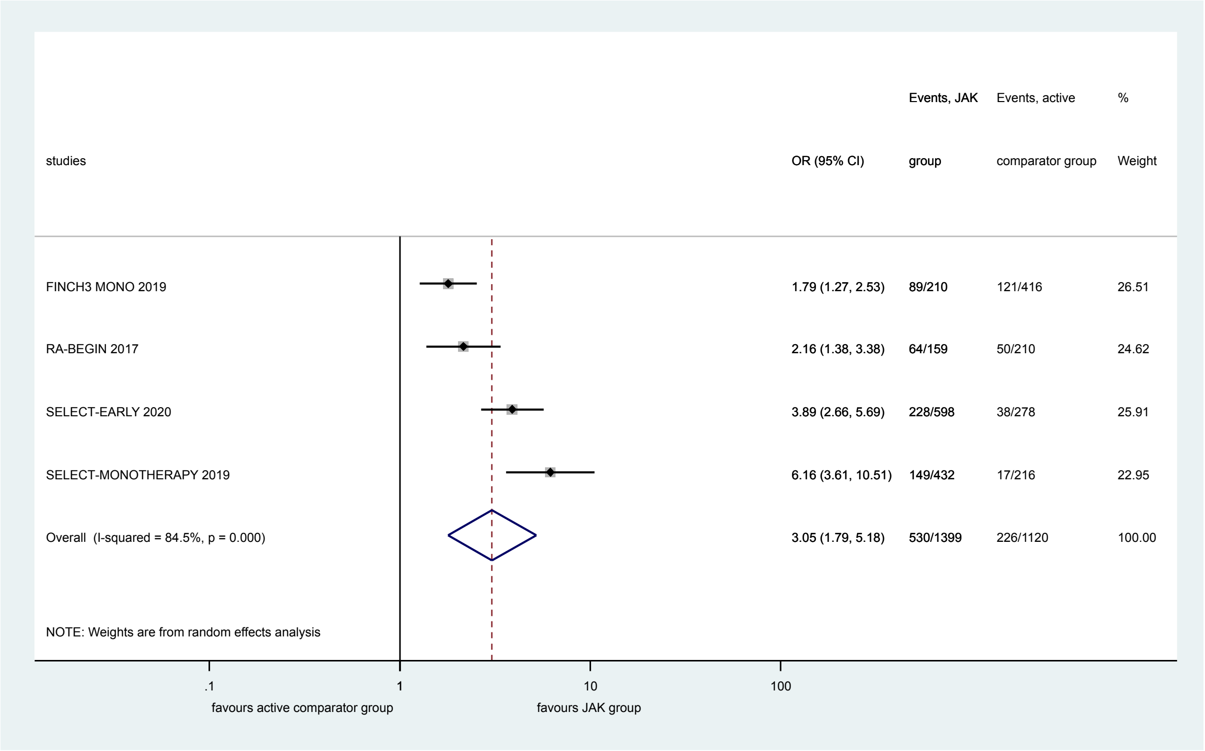

S7139 Forest plot of studies comparing the number of patients reaching remission defined by DAS28-CRP (<2.6) between patients treated with JAK inhibitors and MTX within 6 months (DAS28-CRP remission)

Black diamonds exhibit the individual effect of each study, whereas outer lines show the confidence intervals (CI), and the size of grey squares are proportional to the weight of each study. The blue diamond demonstrates the overall effect, the edges of which represent the CIs.

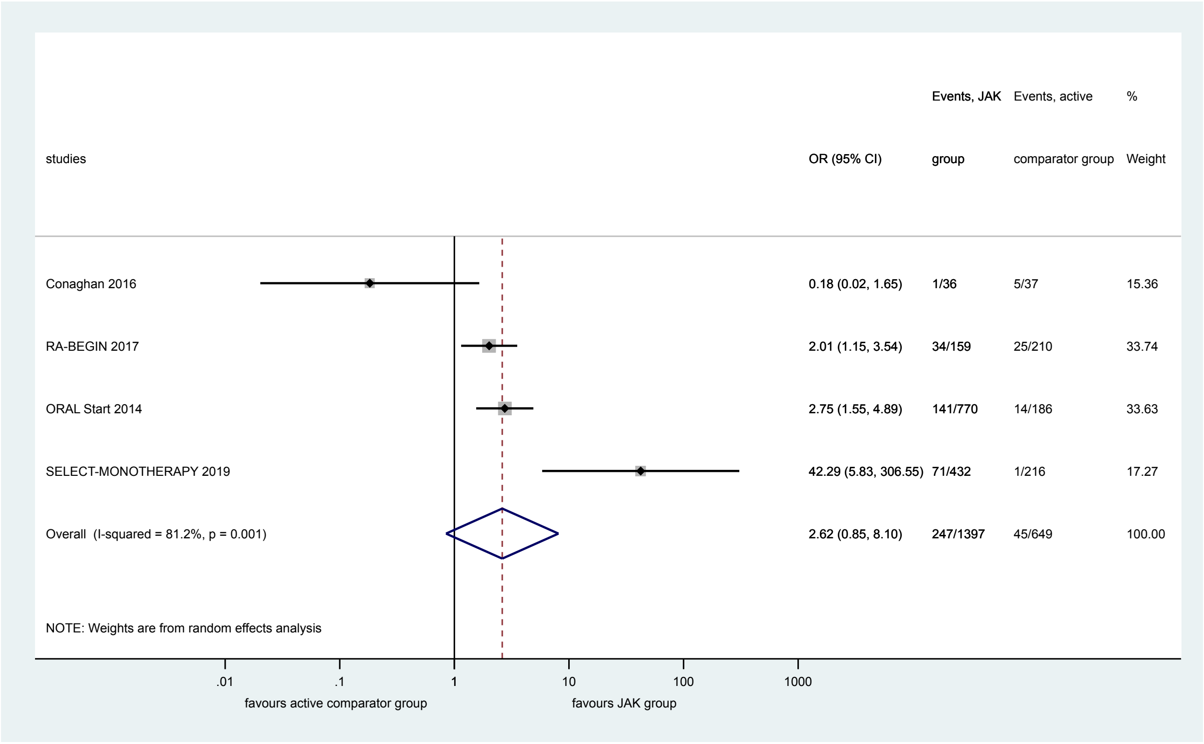

S140 a. Forest plot of studies comparing the number of patients reaching remission defined by DAS28-ESR (<2.6) between patients treated with JAK inhibitors and MTX within 6 months (DAS28-ESR remission)

Black diamonds exhibit the individual effect of each study, whereas outer lines show the confidence intervals (CI), and the size of grey squares are proportional to the weight of each study. The blue diamond demonstrates the overall effect, the edges of which represent the CIs.

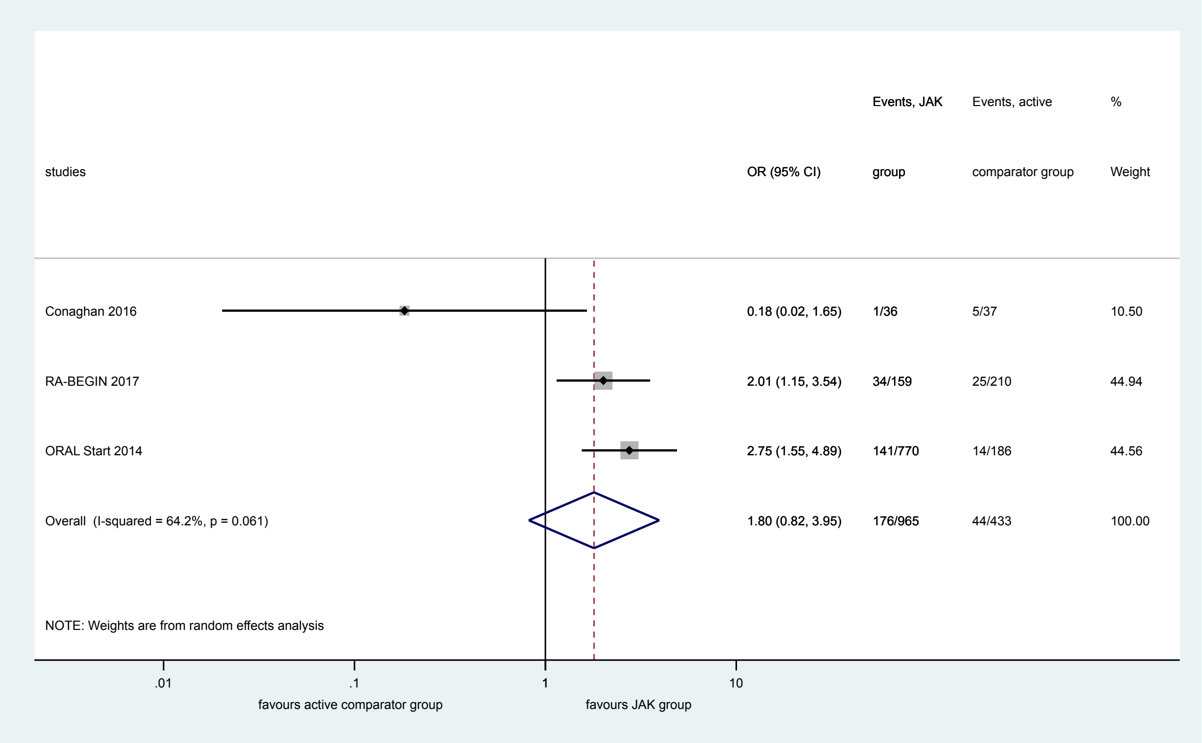

S140 b. Forest plot of studies comparing the number of patients reaching remission defined by DAS28-ESR (<2.6) between patients treated with JAK inhibitors and MTX within 6 months (DAS28-ESR remission) - Secondary analysis only involving studies conducted in a MTX naïve population

Black diamonds exhibit the individual effect of each study, whereas outer lines show the confidence intervals (CI), and the size of grey squares are proportional to the weight of each study. The blue diamond demonstrates the overall effect, the edges of which represent the CIs.

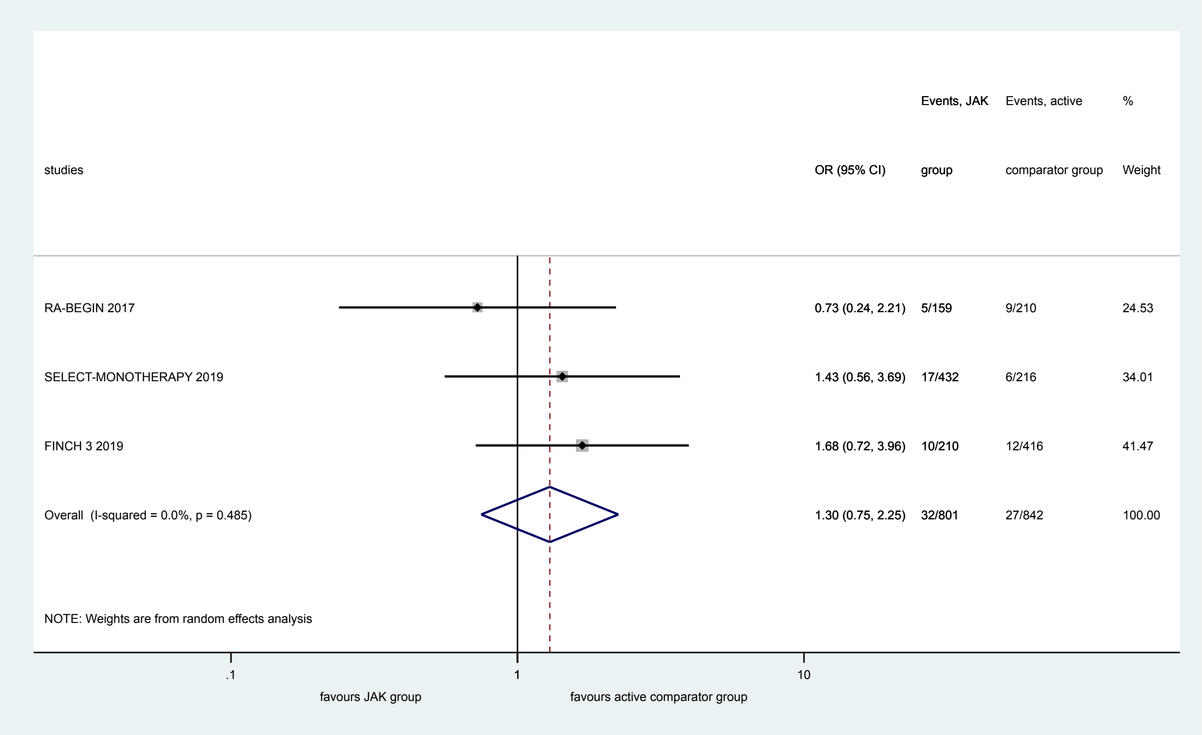

S141. Forest plot of studies comparing the number of patients experiencing serious side effects during the study between patients treated with JAK inhibitors and MTX within 6 months (Serious side effects)

Black diamonds exhibit the individual effect of each study, whereas outer lines show the confidence intervals (CI), and the size of grey squares are proportional to the weight of each study. The blue diamond demonstrates the overall effect, the edges of which represent the CIs.

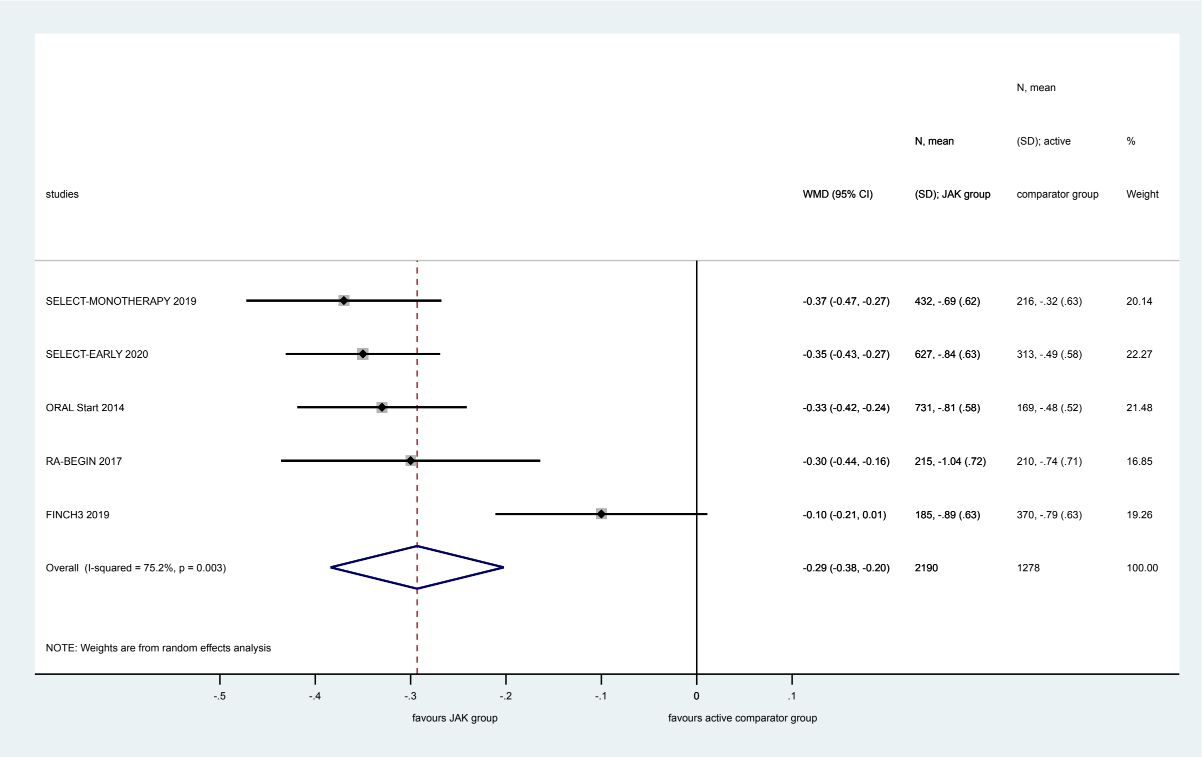

S142. Forest plot of studies comparing the LSM change from baseline in the score on HAQ-DI between patients treated with JAK inhibitors and MTX within 6 months (HAQ-DI difference)

Black diamonds exhibit the individual effect of each study, whereas outer lines show the confidence intervals (CI), and the size of grey squares are proportional to the weight of each study. The blue diamond demonstrates the overall effect, the edges of which represent the CIs.

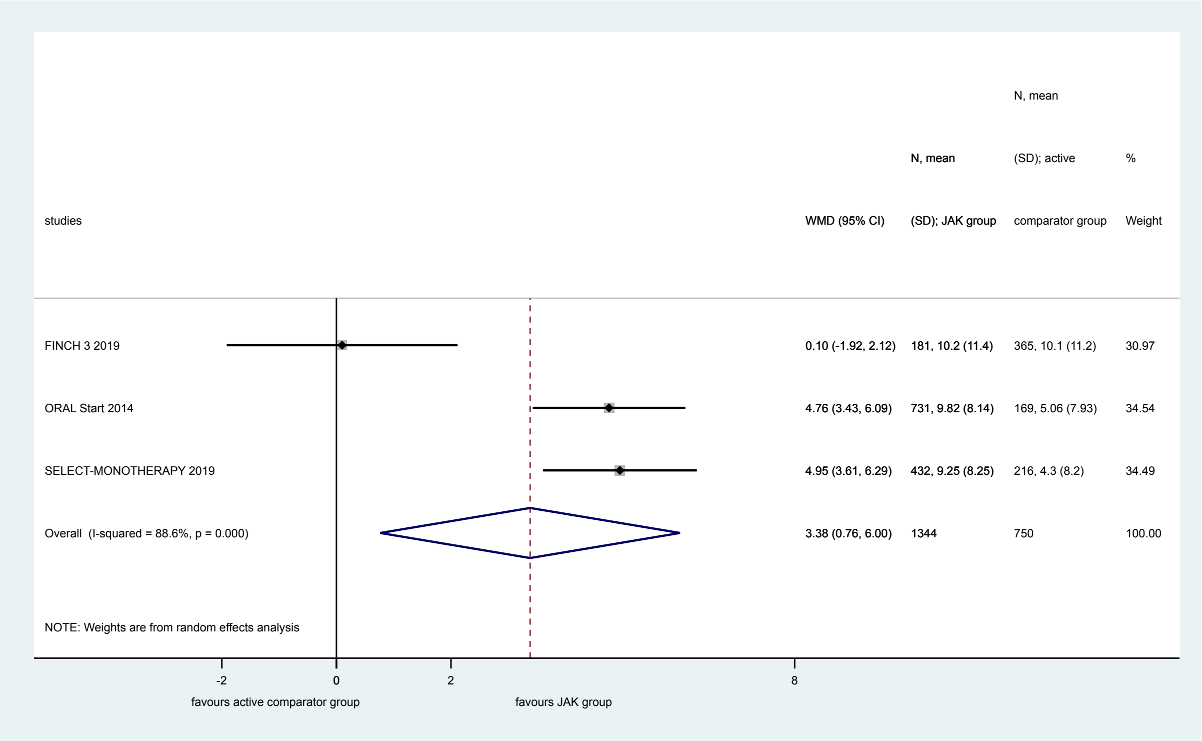

S143. Forest plot of studies comparing the LSM change from baseline in the score on SF-36 assessing the physical component score between patients treated with JAK inhibitors and MTX within 6 months (SF-36 PCS)

Black diamonds exhibit the individual effect of each study, whereas outer lines show the confidence intervals (CI), and the size of grey squares are proportional to the weight of each study. The blue diamond demonstrates the overall effect, the edges of which represent the CIs.

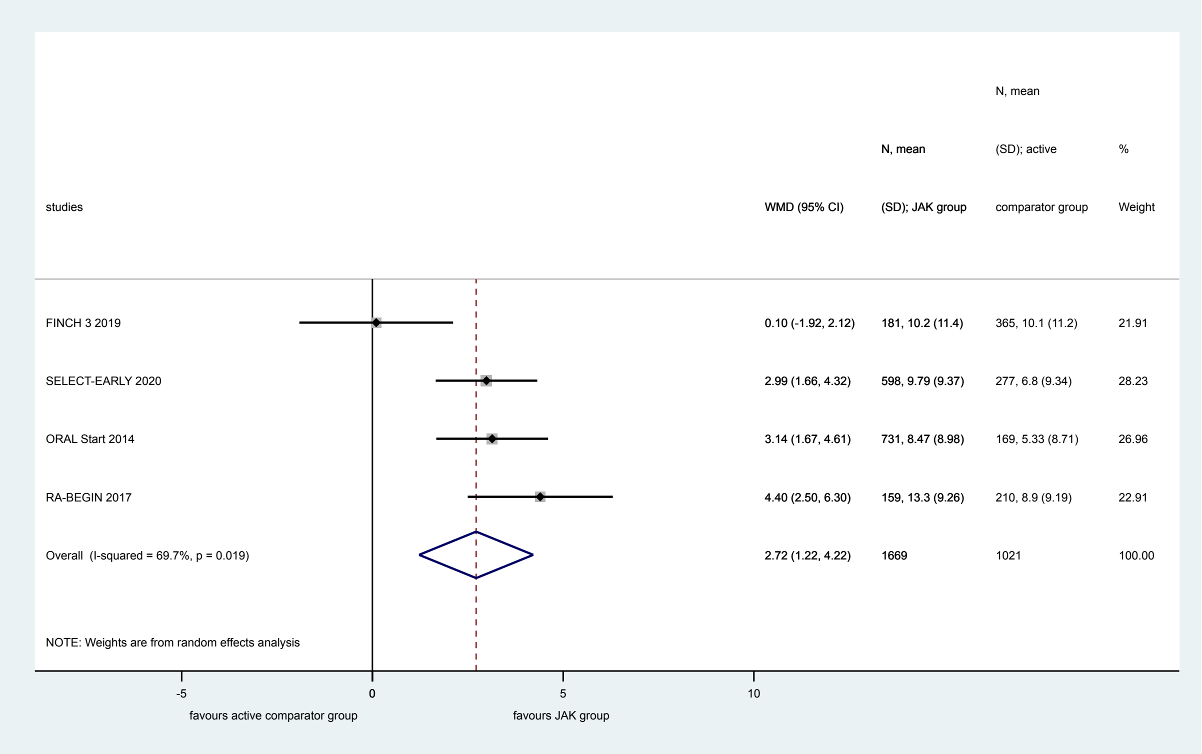

S144. Forest plot of studies comparing the LSM change from baseline in the score on FACIT-F between patients treated with JAK inhibitors and MTX within 6 months (FACIT-F)

Black diamonds exhibit the individual effect of each study, whereas outer lines show the confidence intervals (CI), and the size of grey squares are proportional to the weight of each study. The blue diamond demonstrates the overall effect, the edges of which represent the CIs.

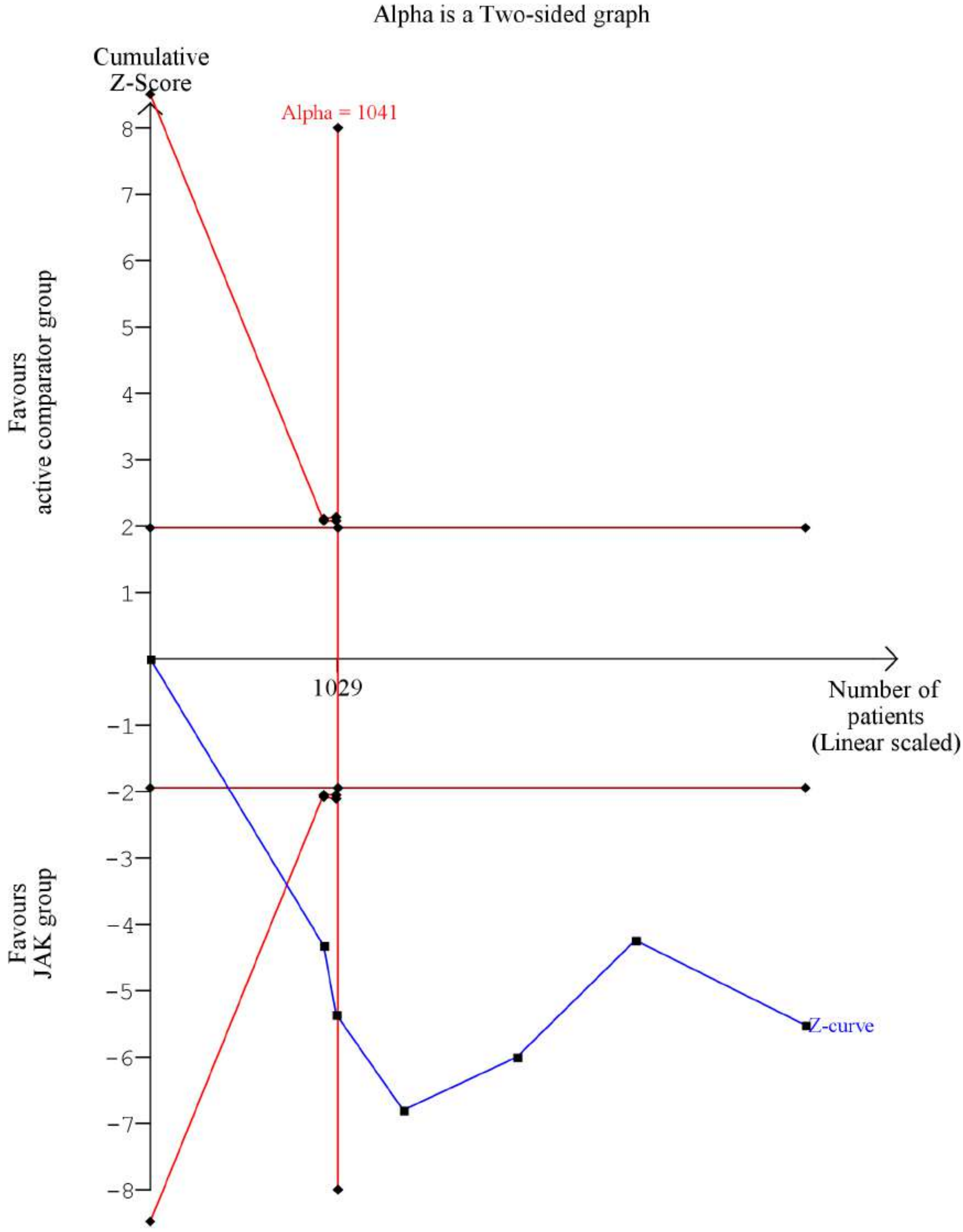

S145. TSA of ACR70 response (JAK-inhibitors vs MTX)

The blue line (Z-curve) crosses the conventional boundary (brown straight line) and trial sequential boundary (red lines sloping inwards), and reaches the line of required sample size, therefore showing the statistically significant superiority of JAK inhibitors in this outcome and the addition of further studies would not influence this results.

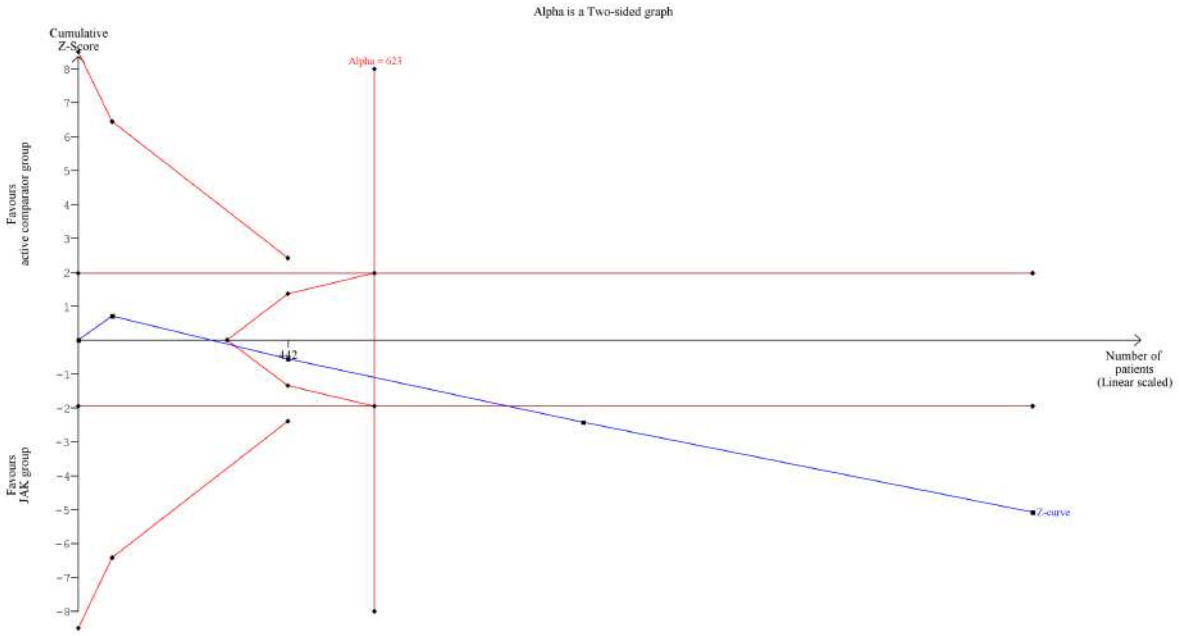

S146. TSA of HAQ-DI improvement (JAK-inhibitors vs MTX)

The blue line (Z-curve) crosses the conventional boundary (brown straight line) and reaches the line of required sample size, therefore showing the statistically significant superiority of JAK inhibitors in this outcome and the addition of further studies would not influence this results.

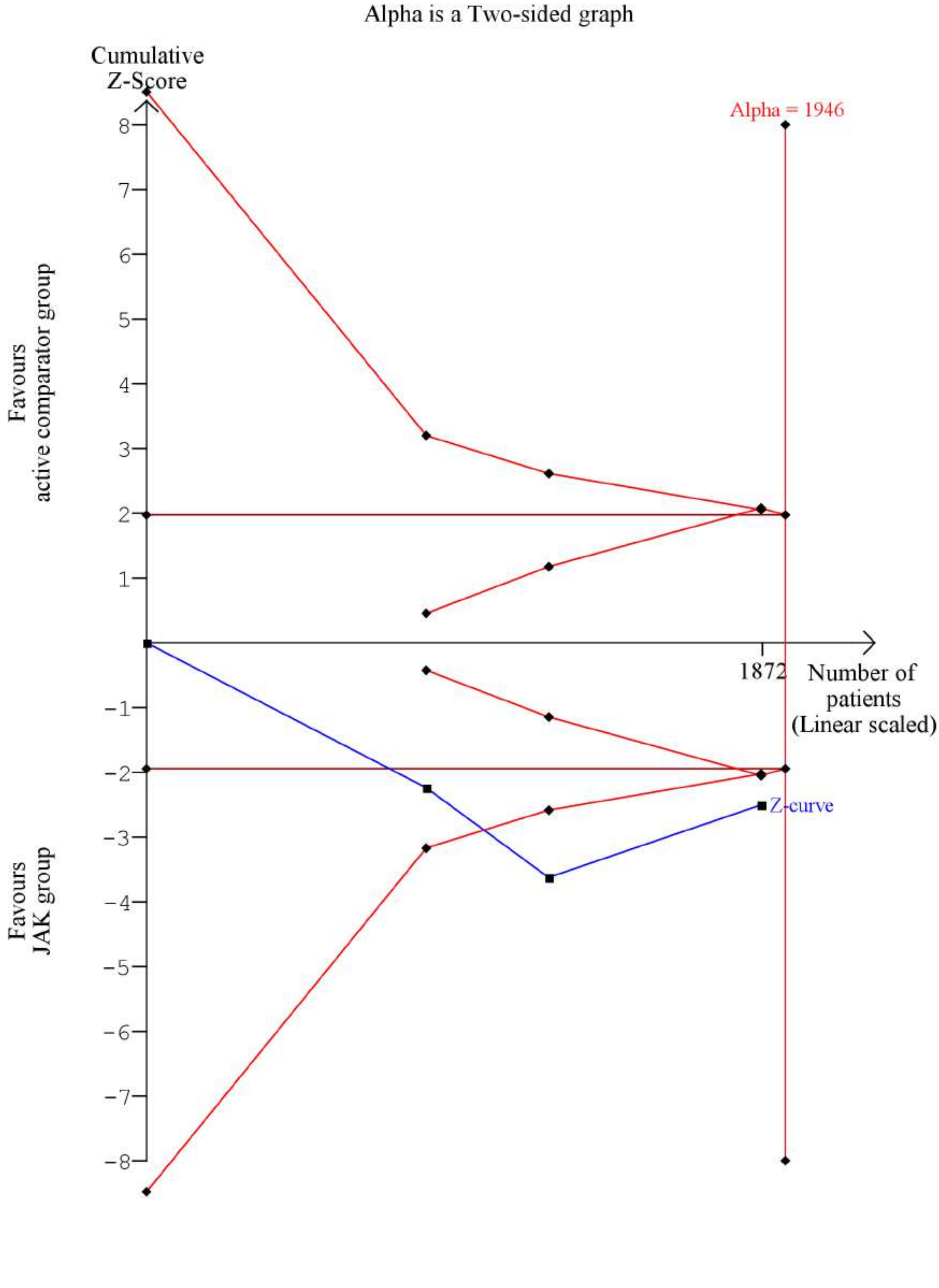

S147. TSA of CDAI remission (JAK-inhibitors vs MTX)

The blue line (Z-curve) crosses the conventional boundary (brown straight line) and trial sequential boundary (red lines sloping inwards) but does not reach the line of required sample size, therefore showing the statistically significant superiority of JAK inhibitors in this outcome but the addition of further studies might influence these results.

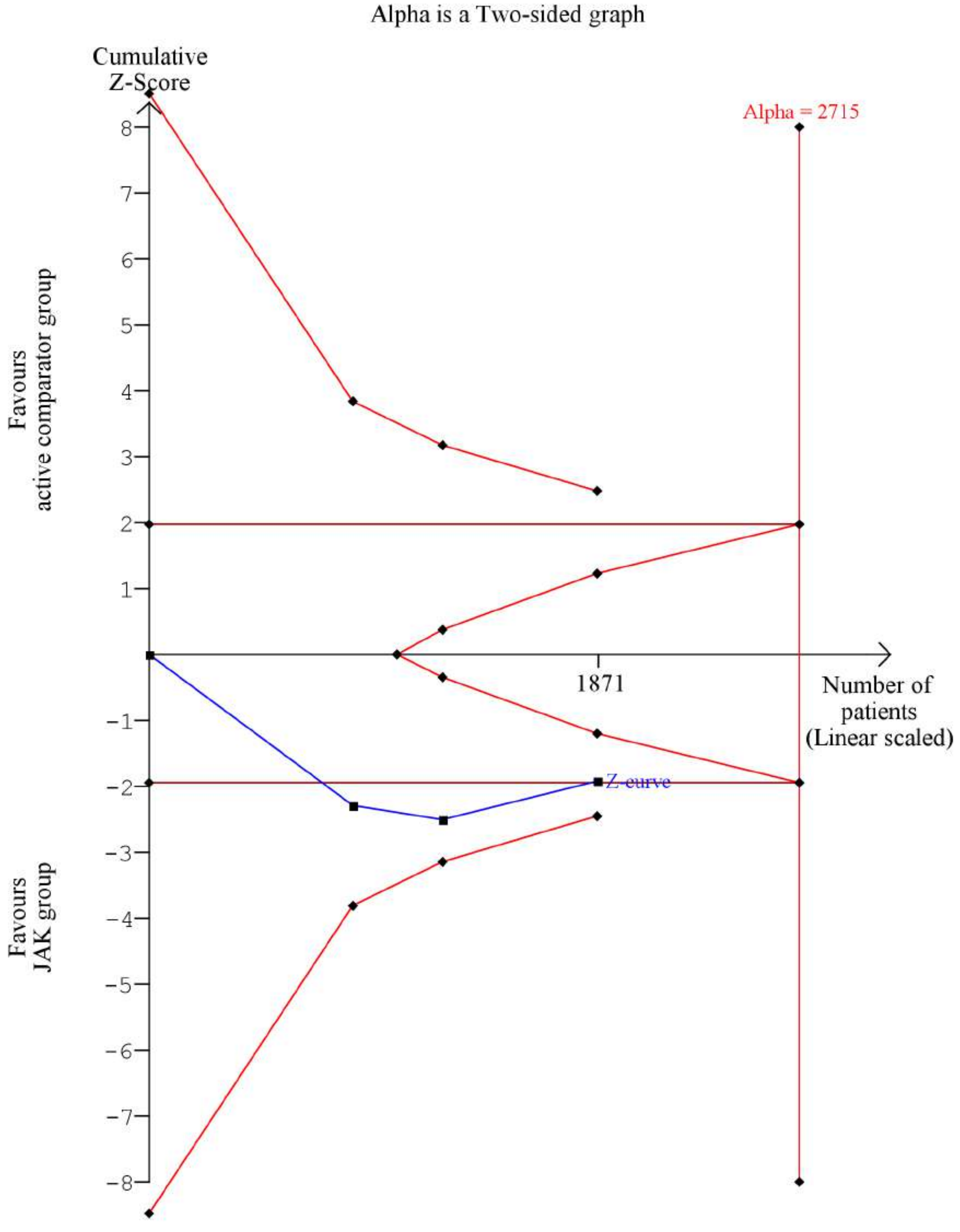

S148. TSA of SDAI remission (JAK-inhibitors vs MTX)

The blue line (Z-curve) first crosses the conventional boundary (brown straight line) but returns and does not reach the line of required sample size, therefore showing no statistically significant difference between groups in this outcome but the addition of further studies might influence these results.

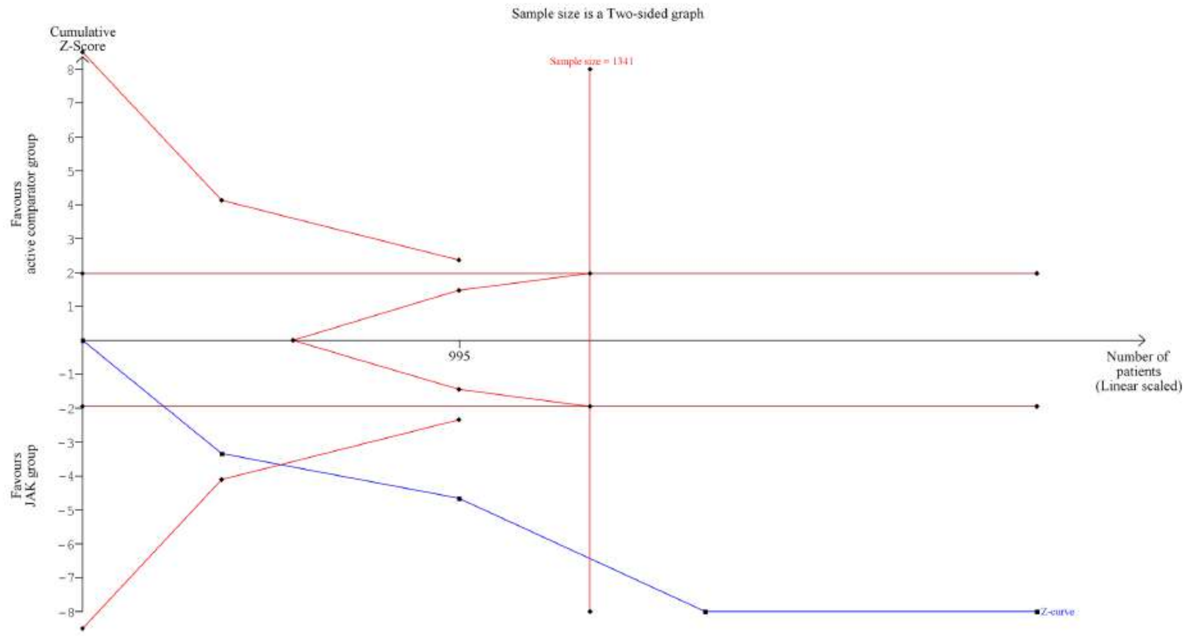

S149. TSA of DAS28-CRP remission (JAK-inhibitors vs MTX)

The blue line (Z-curve) crosses the conventional boundary (brown straight line) and trial sequential boundary (red lines sloping inwards), and reaches the line of required sample size, therefore showing the statistically significant superiority of JAK inhibitors in this outcome and the addition of further studies would not influence this results.

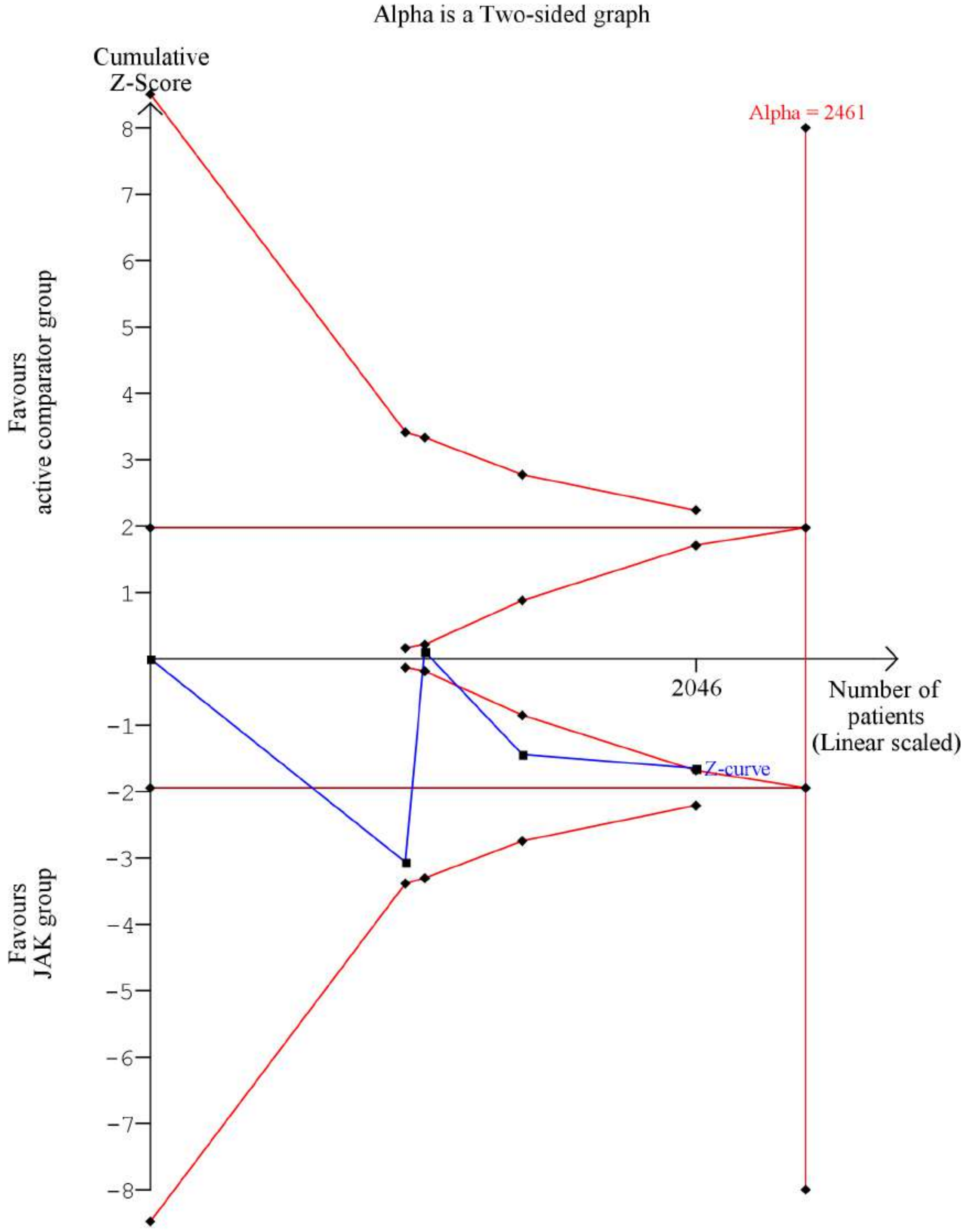

S150. TSA of DAS28-ESR remission (JAK-inhibitors vs MTX)

The blue line (Z-curve) first crosses the conventional boundary (brown straight line) but returns and does not reach the line of required sample size, therefore showing no statistically significant difference between groups in this outcome but the addition of further studies might influence these results.

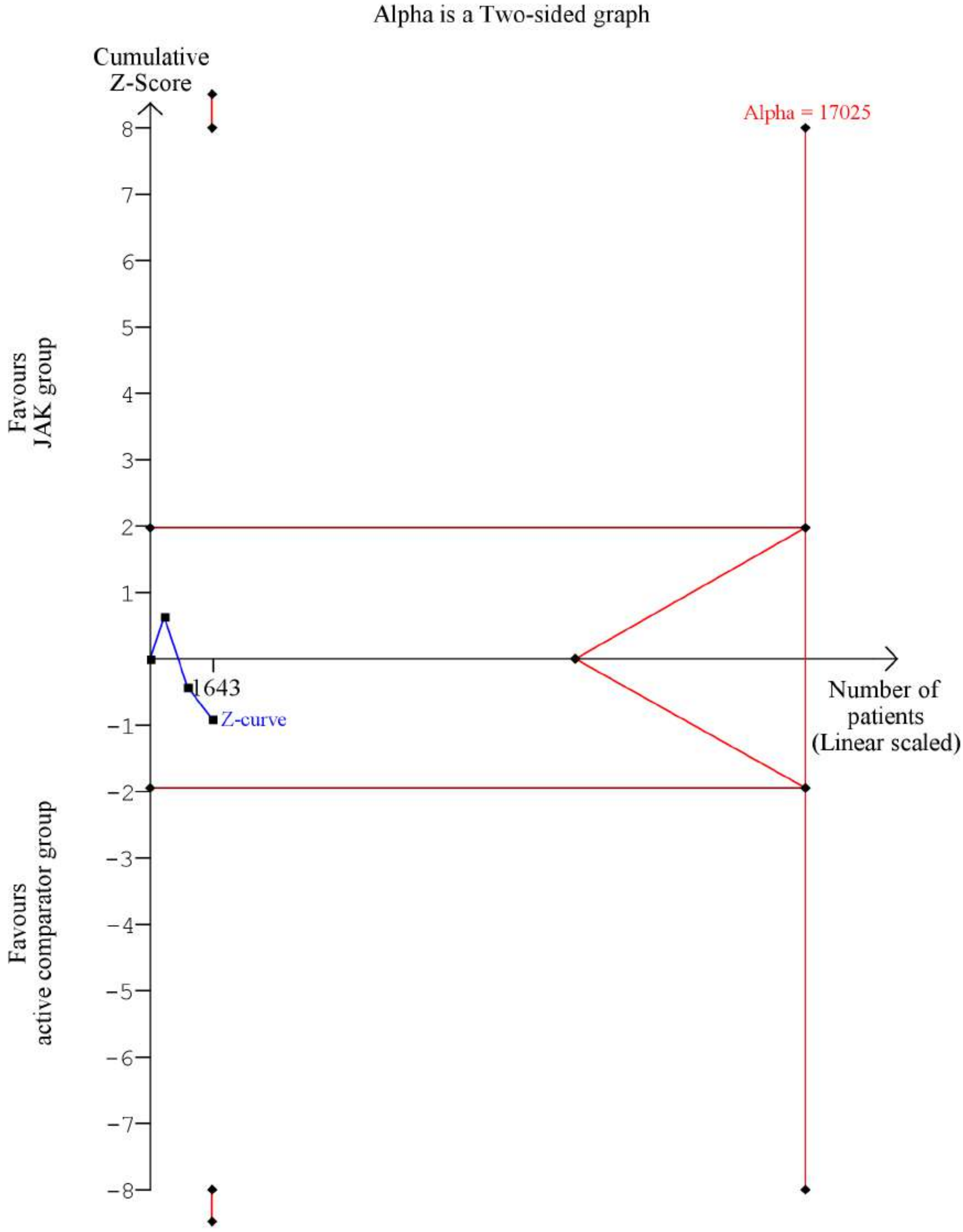

S151. TSA of serious side effects (JAK-inhibitors vs MTX)

The blue line (Z-curve) does not cross the conventional boundary (brown straight line) and does not reach the line of required sample size, therefore showing no statistically significant difference between groups in this outcome and the addition of further studies might influence these results

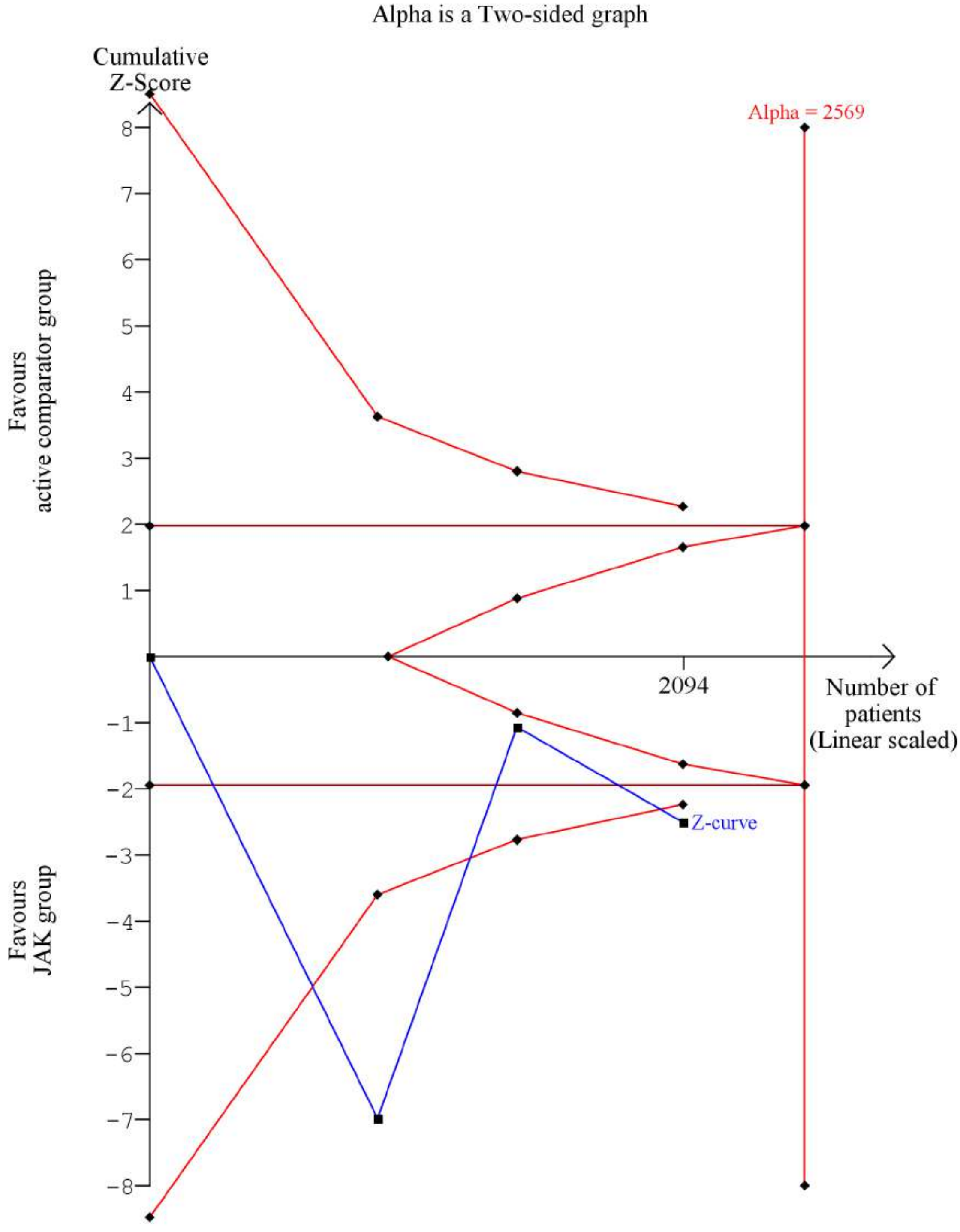

S152. TSA of SF-36 PCS (JAK-inhibitors vs MTX)

The blue line (Z-curve) crosses the conventional boundary (brown straight line) and trial sequential boundary (red lines sloping inwards) but does not reach the line of required sample size, therefore showing the statistically significant superiority of JAK inhibitors in this outcome but the addition of further studies might influence these results.

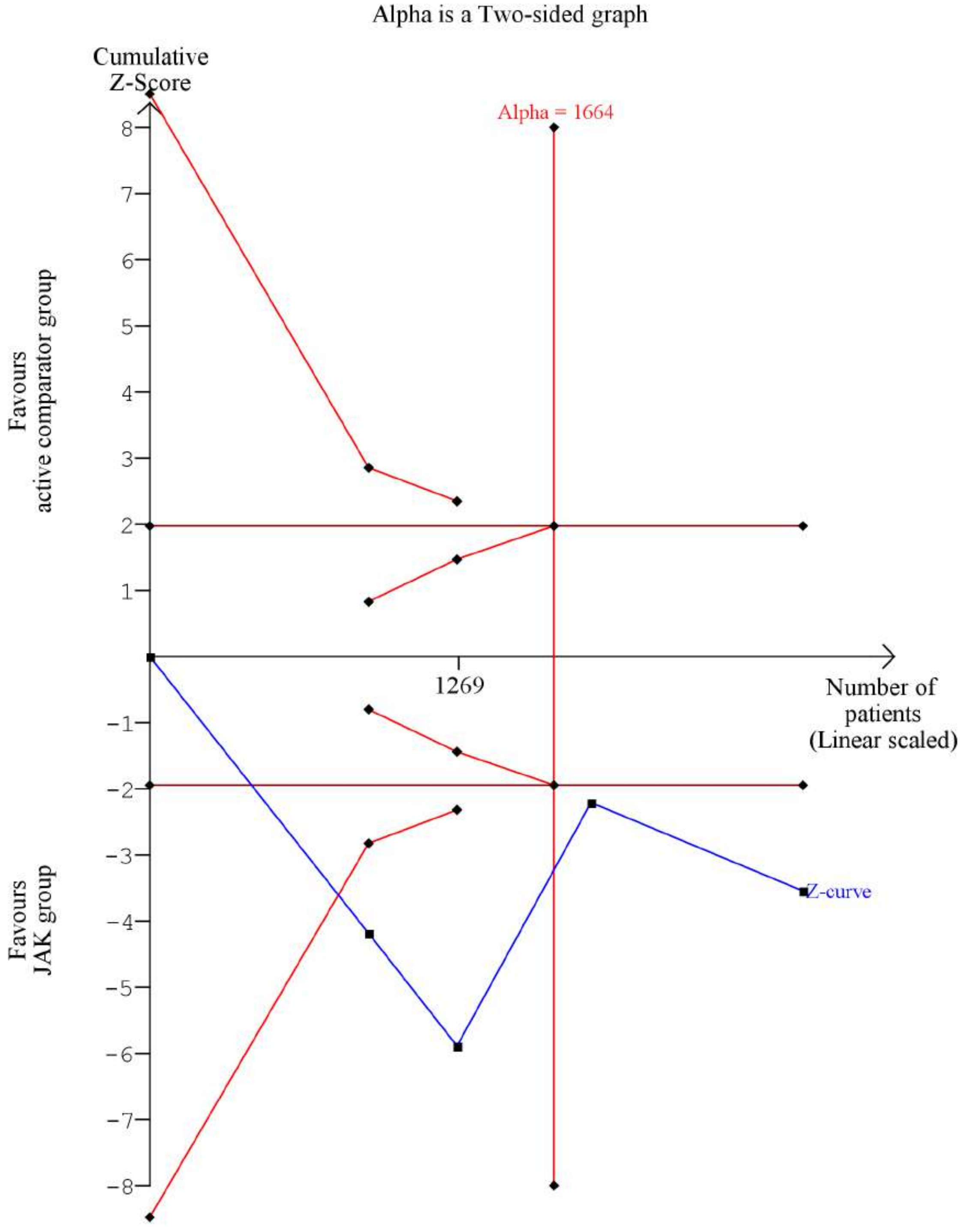

S153. TSA of FACIT-F (JAK-inhibitors vs MTX)

The blue line (Z-curve) crosses the conventional boundary (brown straight line) and trial sequential boundary (red lines sloping inwards), and reaches the line of required sample size, therefore showing the statistically significant superiority of JAK inhibitors in this outcome and the addition of further studies would not influence these results.

(3) JAK inhibitors compared to bDMARDs

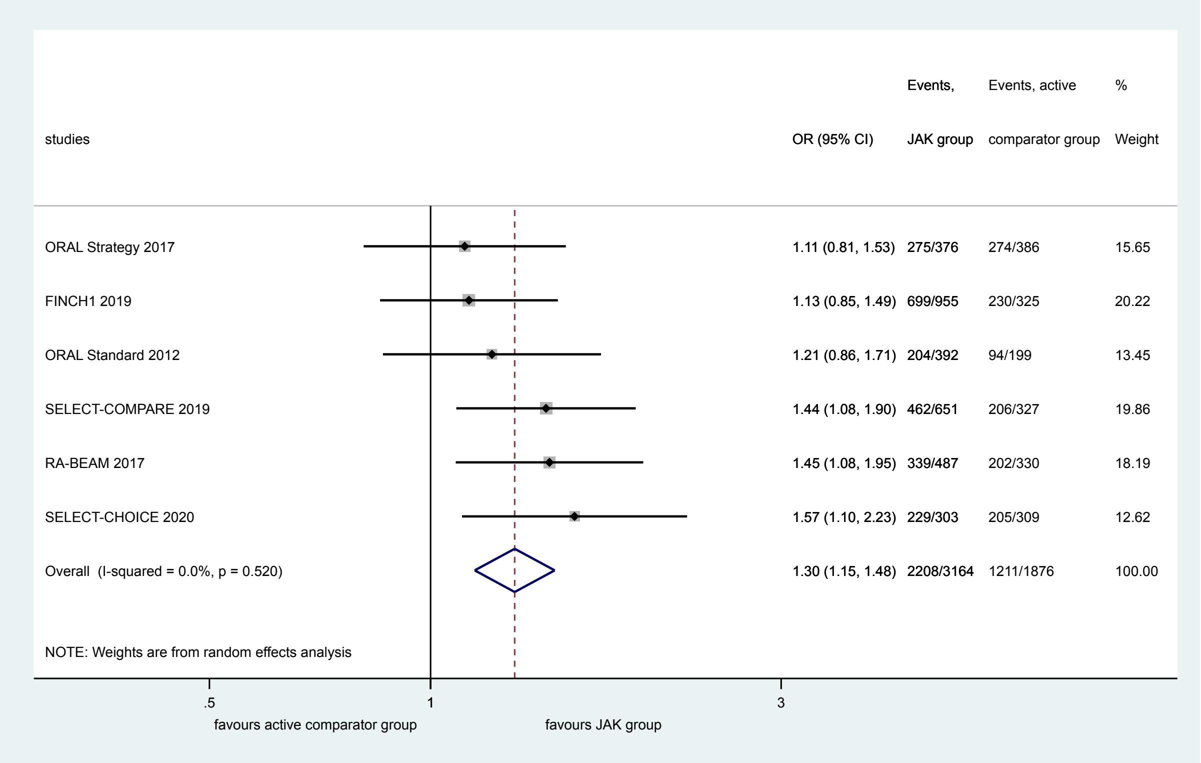

S154. Forest plot of studies comparing the number of patients reaching at least 20% in ACR criteria between patients treated with JAK inhibitors and bDMARDs within 6 months (ACR20 response)

Black diamonds exhibit the individual effect of each study, whereas outer lines show the confidence intervals (CI) and the size of grey squares are proportional to the weight of each study. The blue diamond demonstrates the overall effect, the edges of which represent the CIs.

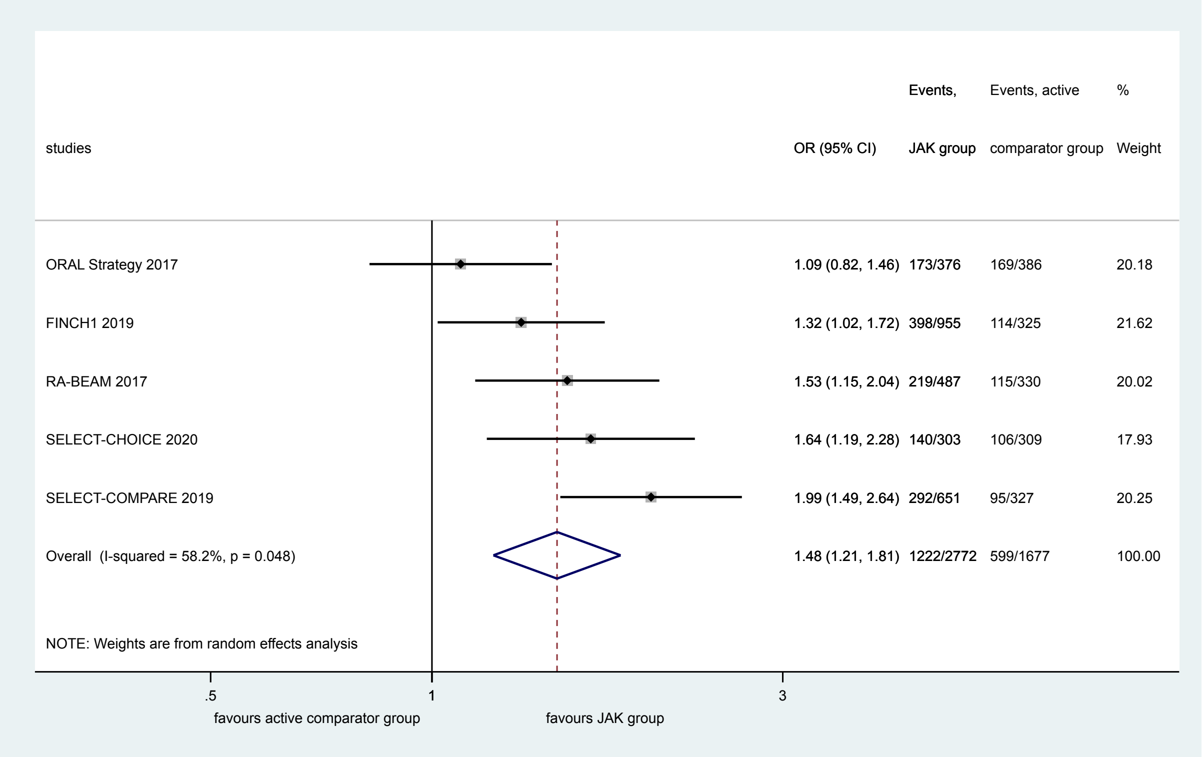

S155. Forest plot of studies comparing the number of patients reaching at least 50% in ACR criteria between patients treated with JAK inhibitors and bDMARDs within 6 months (ACR50 response)

Black diamonds exhibit the individual effect of each study, whereas outer lines show the confidence intervals (CI) and the size of grey squares are proportional to the weight of each study. The blue diamond demonstrates the overall effect, the edges of which represent the CIs.

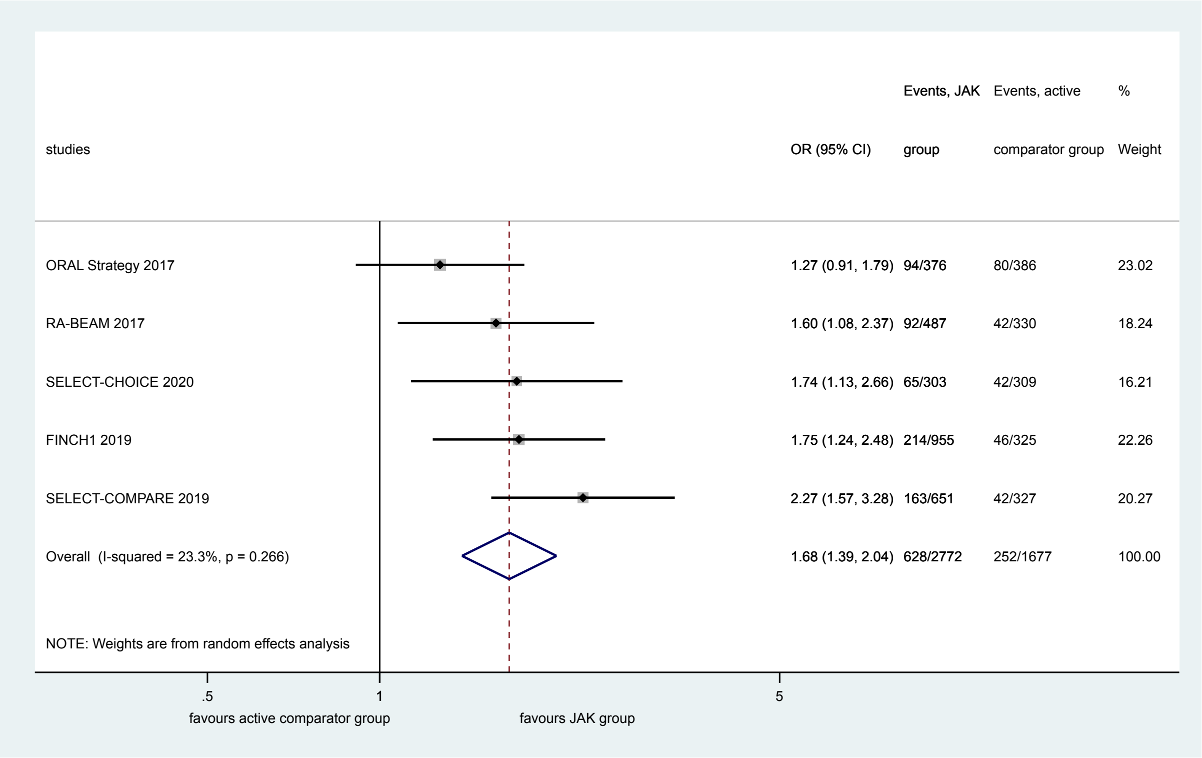

S156. Forest plot of studies comparing the number of patients reaching at least 70% in ACR criteria between patients treated with JAK inhibitors and bDMARDs within 6 months (ACR70 response)

Black diamonds exhibit the individual effect of each study, whereas outer lines show the confidence intervals (CI) and the size of grey squares are proportional to the weight of each study. The blue diamond demonstrates the overall effect, the edges of which represent the CIs.

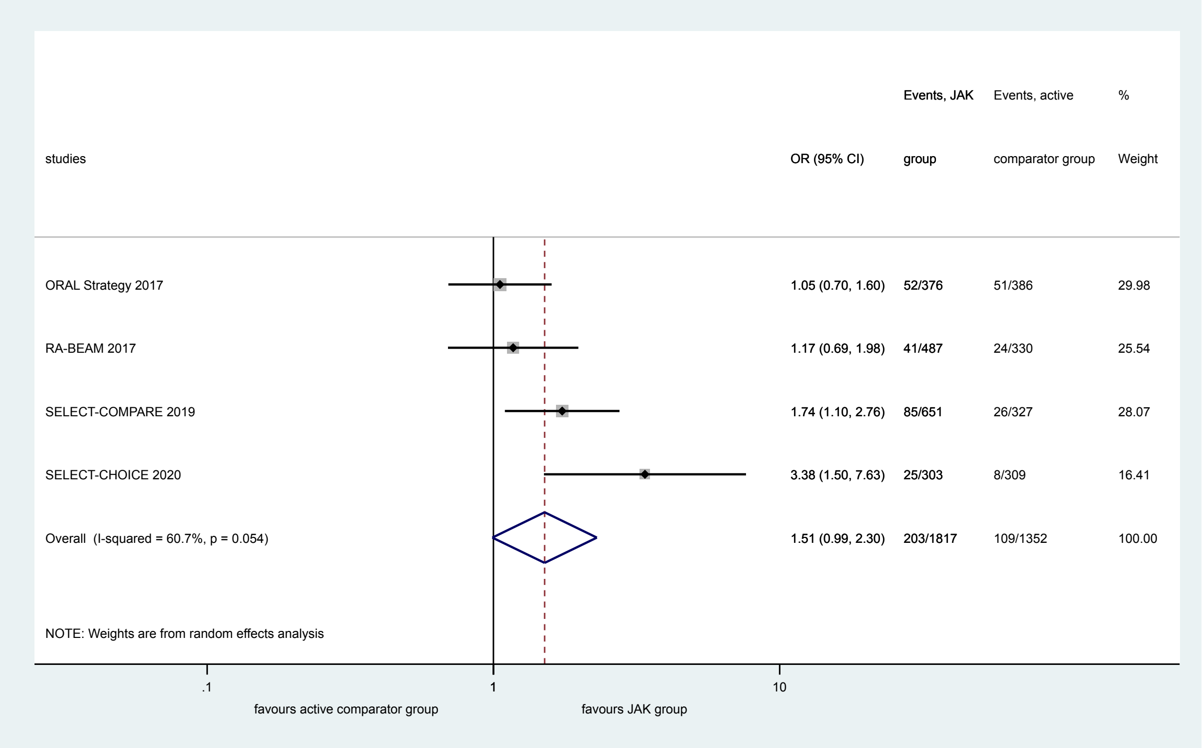

S157. Forest plot of studies comparing the number of patients reaching remission defined by CDAI (≤ 2.8) between patients treated with JAK inhibitors and bDMARDs within 6 months (CDAI remission)

Black diamonds exhibit the individual effect of each study, whereas outer lines show the confidence intervals (CI) and the size of grey squares are proportional to the weight of each study. The blue diamond demonstrates the overall effect, the edges of which represent the CIs.

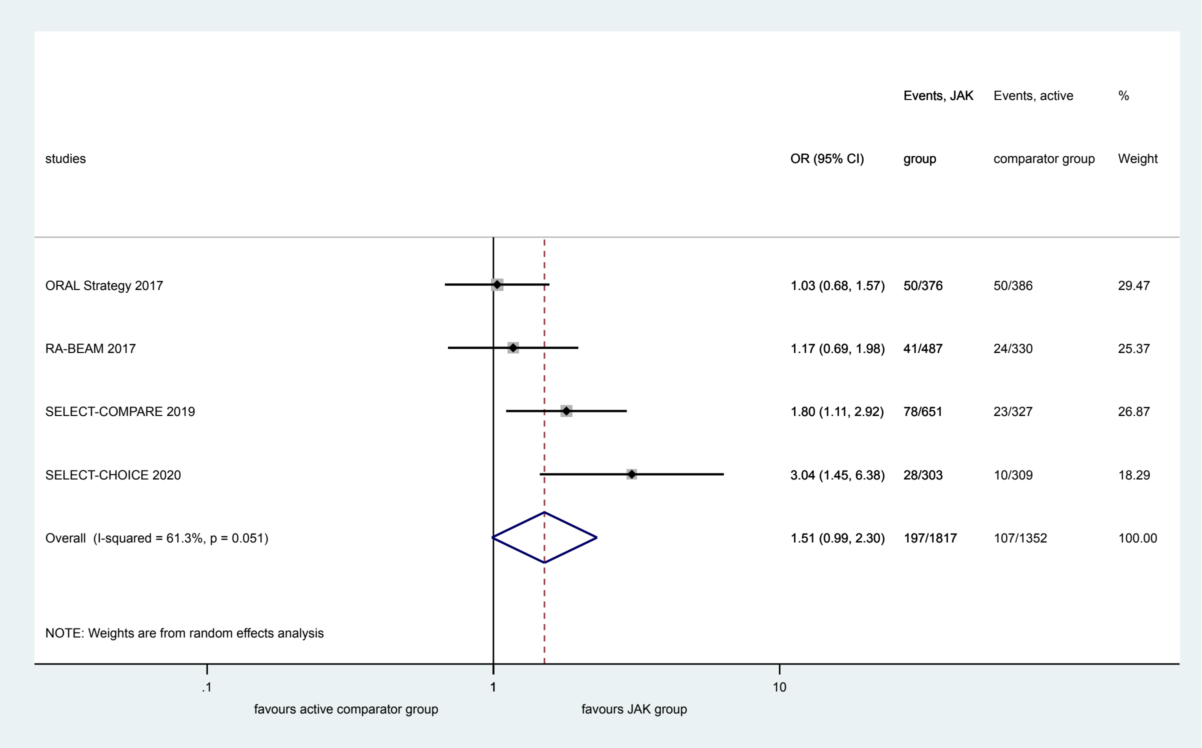

S158. Forest plot of studies comparing the number of patients reaching remission defined by SDAI (≤ 3.3) between patients treated with JAK inhibitors and bDMARDs within 6 months (SDAI remission)

Black diamonds exhibit the individual effect of each study, whereas outer lines show the confidence intervals (CI), and the size of grey squares are proportional to the weight of each study. The blue diamond demonstrates the overall effect, the edges of which represent the CIs.

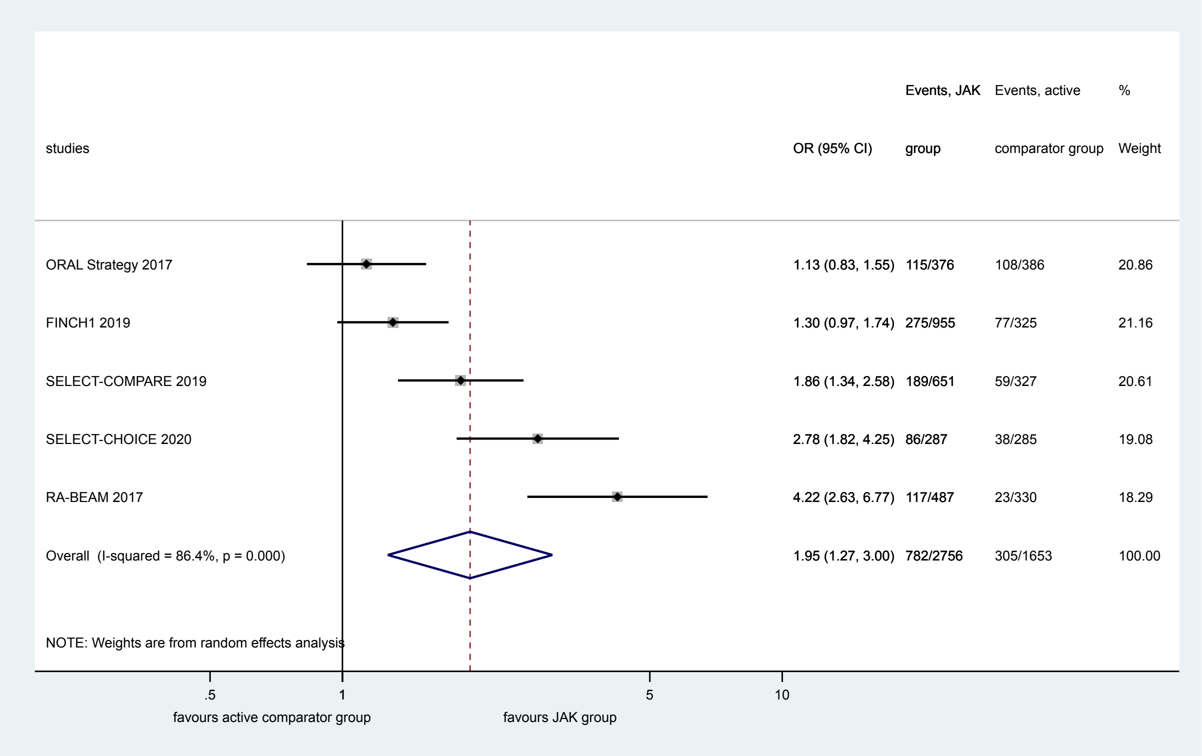

S159. Forest plot of studies comparing the number of patients reaching remission defined by DAS28-CRP (<2.6) between patients treated with JAK inhibitors and tbDMRADs within 6 months (DAS28-CRP remission)

Black diamonds exhibit the individual effect of each study, whereas outer lines show the confidence intervals (CI), and the size of grey squares are proportional to the weight of each study. The blue diamond demonstrates the overall effect, the edges of which represent the CIs.

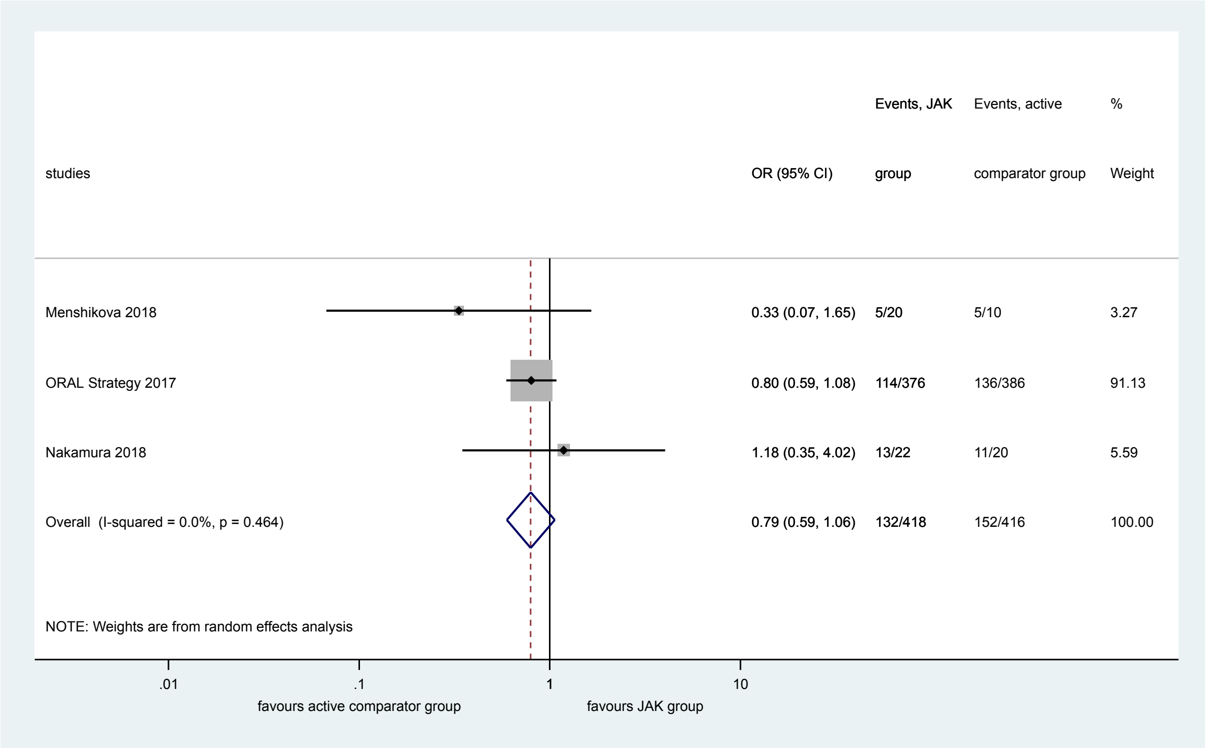

S160. Forest plot of studies comparing the number of patients reaching remission defined by DAS28-CRP (<2.6) between patients treated with JAK inhibitors and tbDMRADs over 6 months (DAS28-CRP remission)

Black diamonds exhibit the individual effect of each study, whereas outer lines show the confidence intervals (CI), and the size of grey squares are proportional to the weight of each study. The blue diamond demonstrates the overall effect, the edges of which represent the CIs.

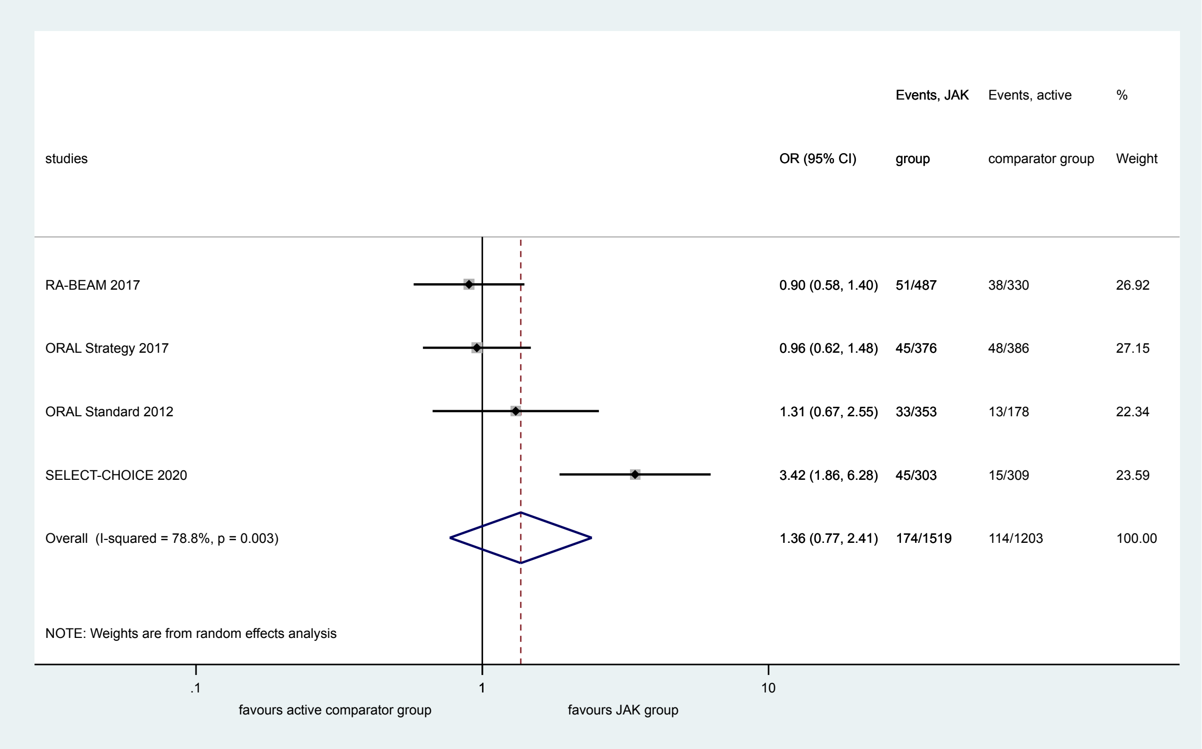

S161. Forest plot of studies comparing the number of patients reaching remission defined by DAS28-ESR (<2.6) between patients treated with JAK inhibitors and tbDMRADs within 6 months (DAS28-ESR remission)

Black diamonds exhibit the individual effect of each study, whereas outer lines show the confidence intervals (CI), and the size of grey squares are proportional to the weight of each study. The blue diamond demonstrates the overall effect, the edges of which represent the CIs.

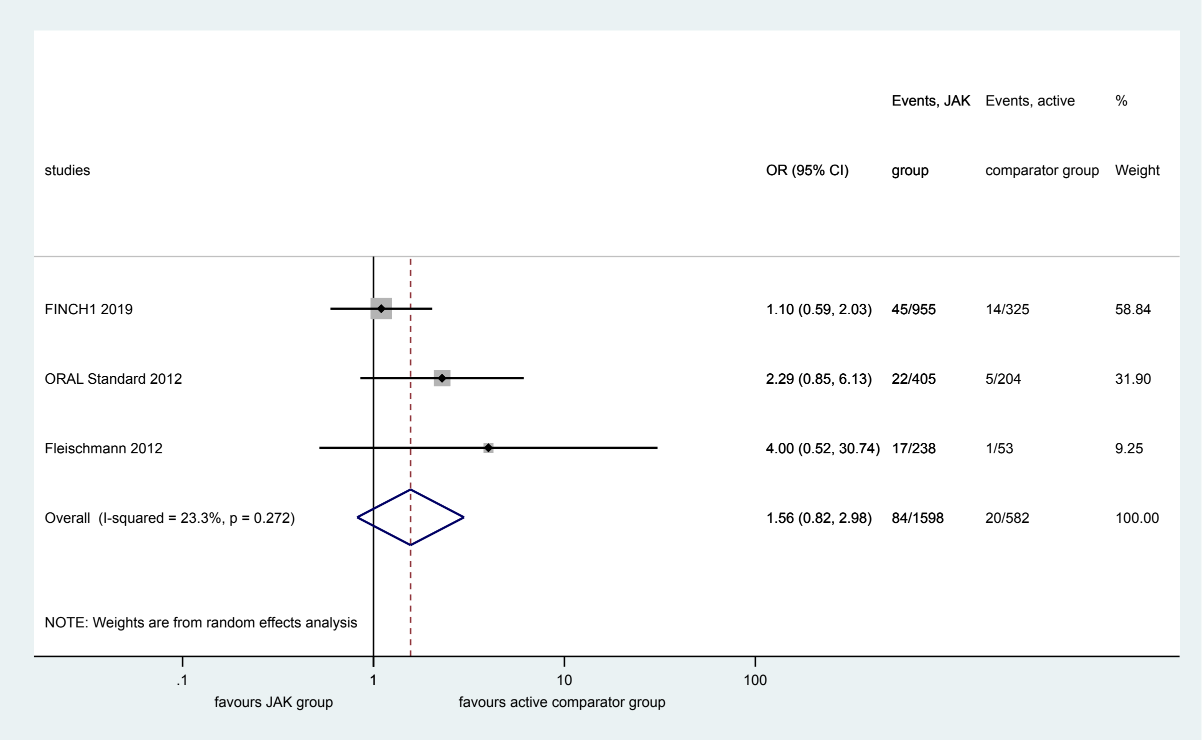

S162. Forest plot of studies comparing the number of patients experiencing serious side effects during the study between patients treated with JAK inhibitors and bDMARDs within 6 months (Serious side effects)

Black diamonds exhibit the individual effect of each study, whereas outer lines show the confidence intervals (CI), and the size of grey squares are proportional to the weight of each study. The blue diamond demonstrates the overall effect, the edges of which represent the CIs.

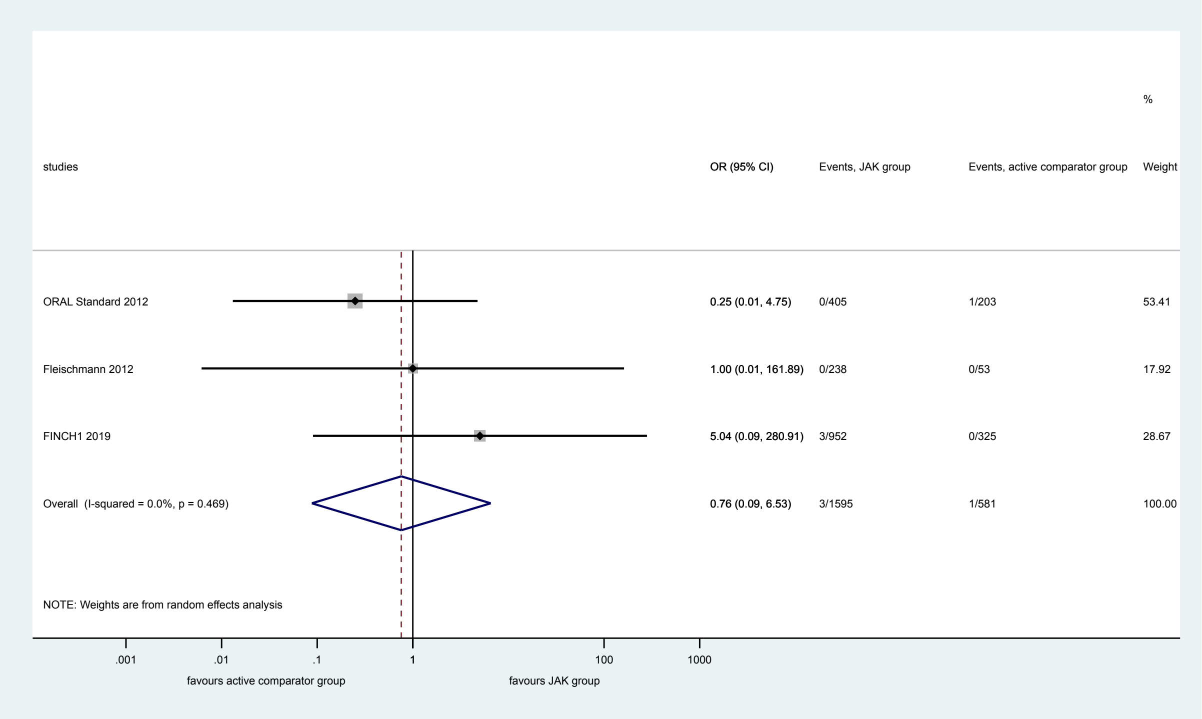

S163. Forest plot of studies comparing the number of patients who dies during study period between patients treated with JAK inhibitors and bDMARDs (Deaths)

Black diamonds exhibit the individual effect of each study, whereas outer lines show the confidence intervals (CI), and the size of grey squares are proportional to the weight of each study. The blue diamond demonstrates the overall effect, the edges of which represent the CIs.

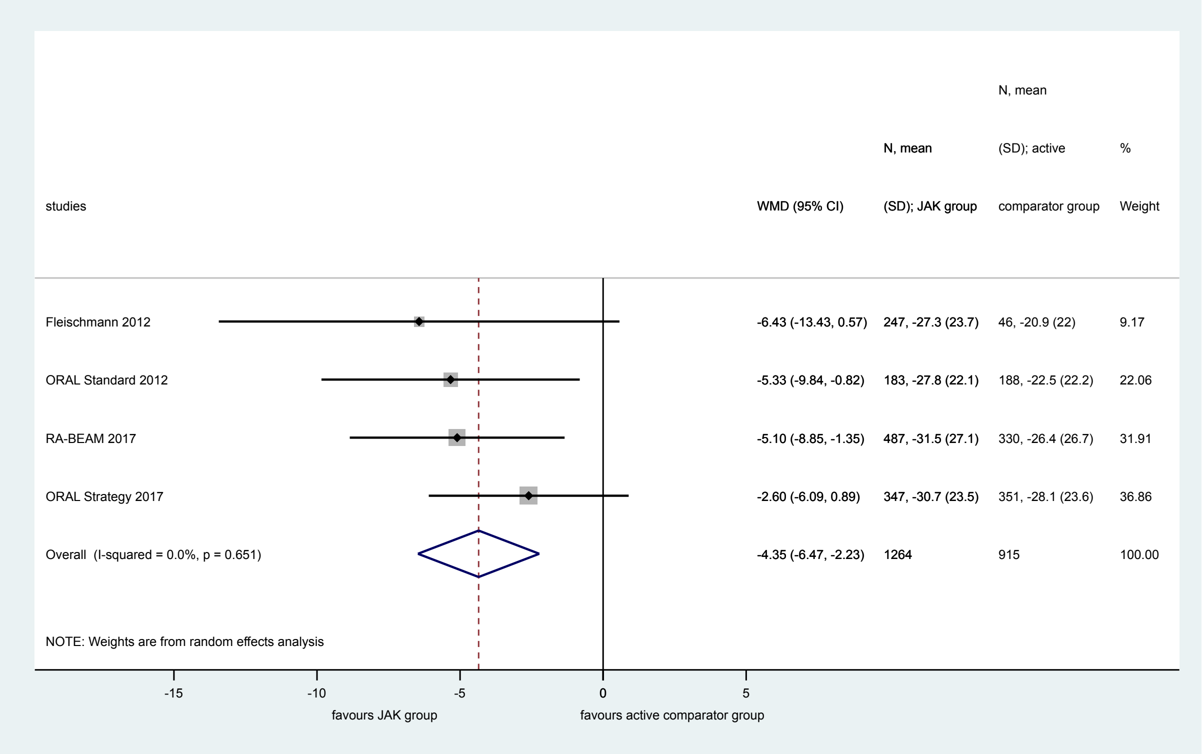

S164. Forest plot of studies comparing the LSM change from baseline in pain measured on VAS ranging from 0-100 mm between patients treated with JAK inhibitors and bDMARDs within 6 months (Pain VAS)

Black diamonds exhibit the individual effect of each study, whereas outer lines show the confidence intervals (CI), and the size of grey squares are proportional to the weight of each study. The blue diamond demonstrates the overall effect, the edges of which represent the CIs.

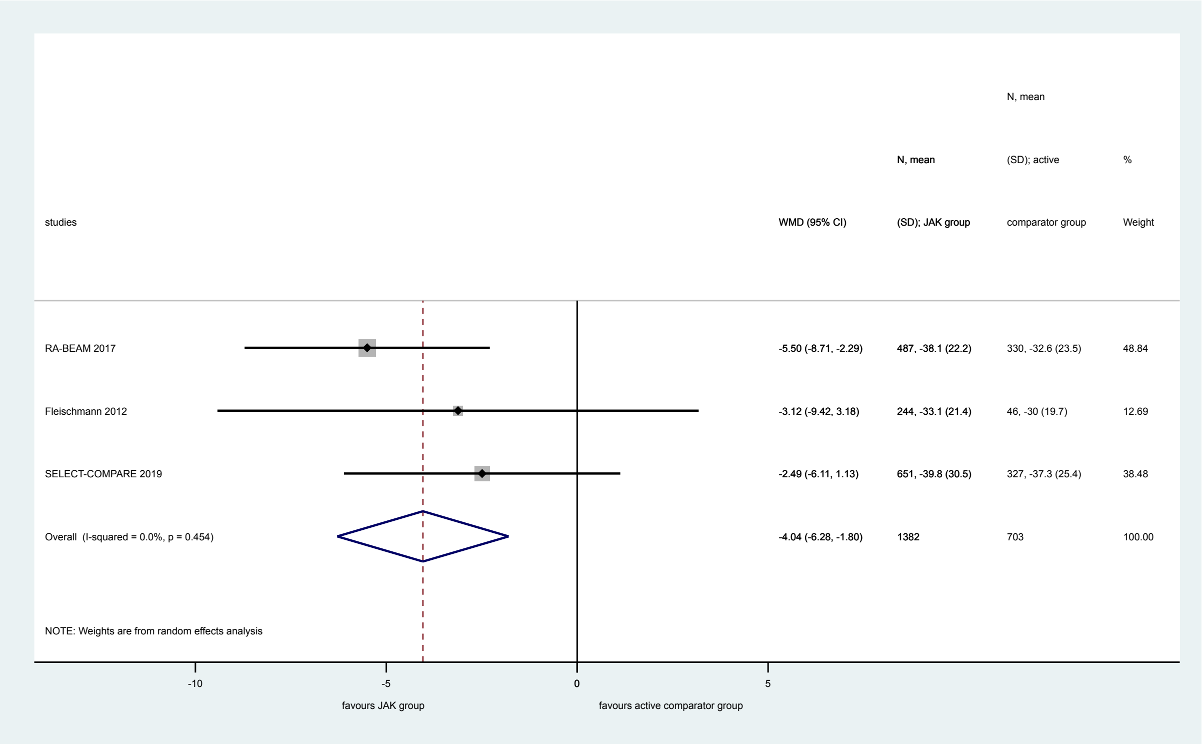

S165. Forest plot of studies comparing the LSM change from baseline in PGA measured on VAS ranging from 0-100 mm between patients treated with JAK inhibitors and bDMARDs within 6 months (PGA VAS)

Black diamonds exhibit the individual effect of each study, whereas outer lines show the confidence intervals (CI), and the size of grey squares are proportional to the weight of each study. The blue diamond demonstrates the overall effect, the edges of which represent the CIs.

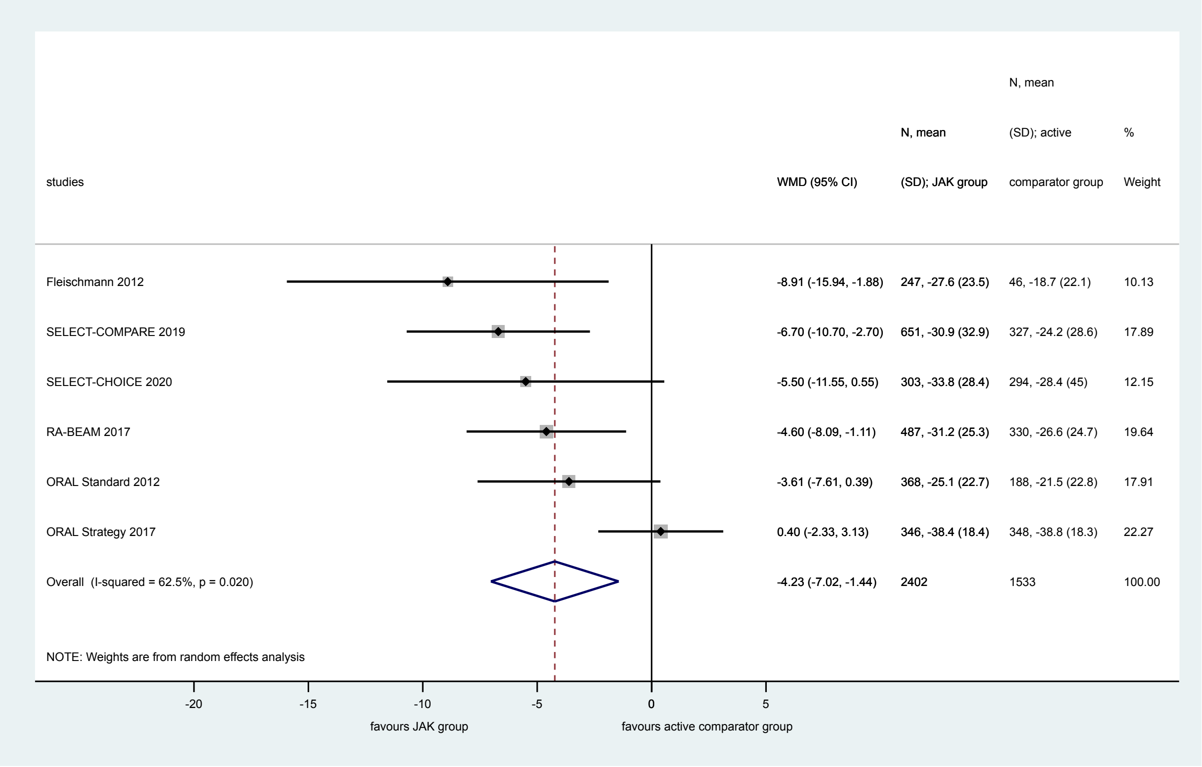

S166. Forest plot of studies comparing the LSM change from baseline in PtGA measured on VAS ranging from 0-100 mm between patients treated with JAK inhibitors and bDMARDs within 6 months (PtGA VAS)

Black diamonds exhibit the individual effect of each study, whereas outer lines show the confidence intervals (CI), and the size of grey squares are proportional to the weight of each study. The blue diamond demonstrates the overall effect, the edges of which represent the CIs.

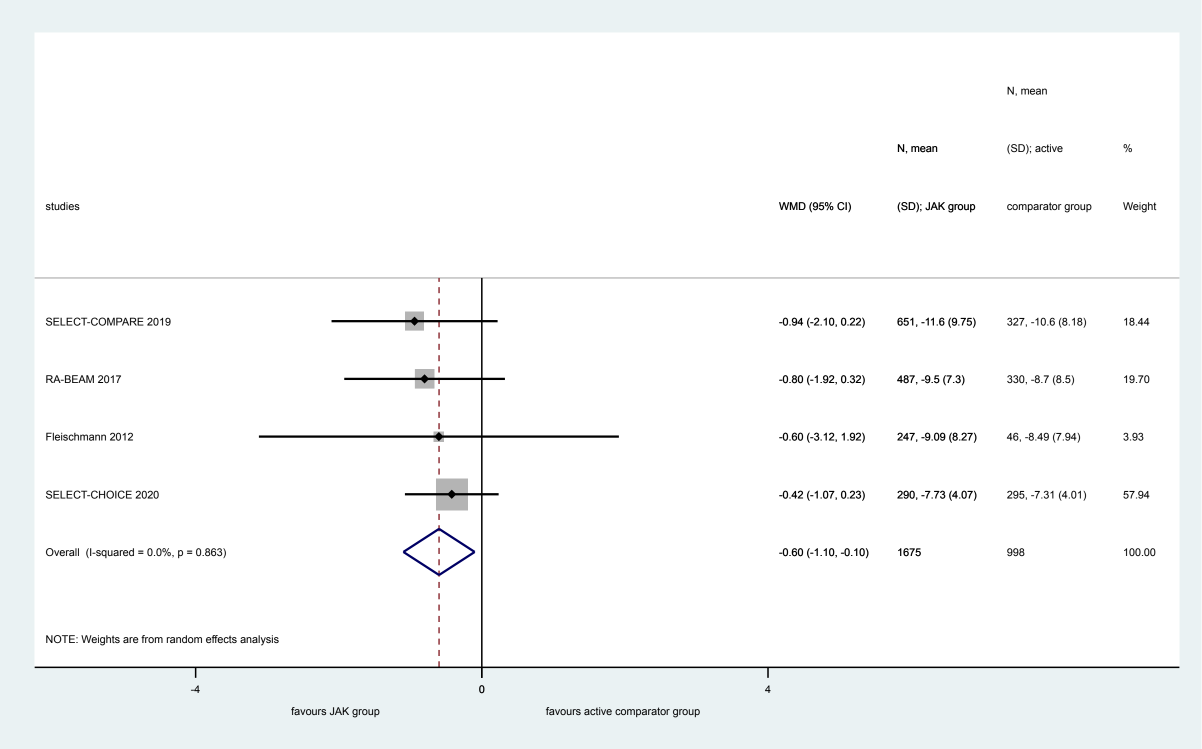

S167. Forest plot of studies comparing the LSM change from baseline in the number of swollen joint counts between patients treated with JAK inhibitors and bDMARDs within 6 months (Swollen Joint Count)

Black diamonds exhibit the individual effect of each study, whereas outer lines show the confidence intervals (CI), and the size of grey squares are proportional to the weight of each study. The blue diamond demonstrates the overall effect, the edges of which represent the CIs.

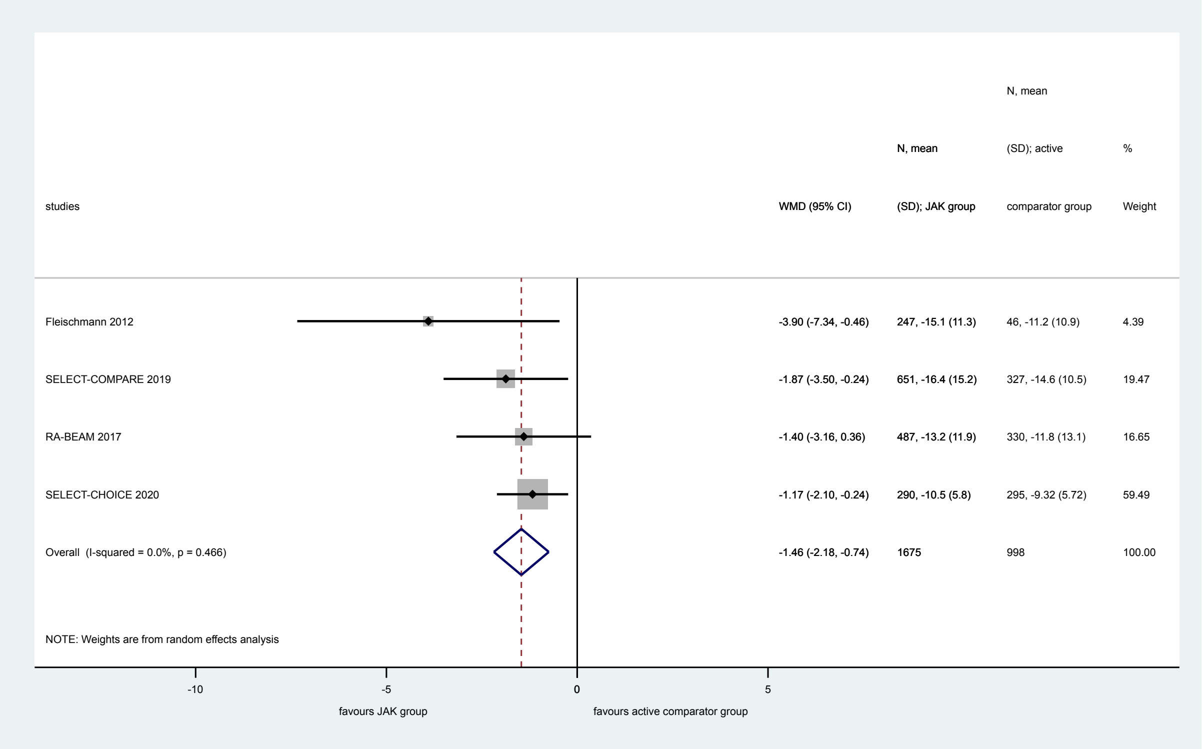

S168. Forest plot of studies comparing the LSM change from baseline in the number of tender joint counts between patients treated with JAK inhibitors and bDMARDs within 6 months (Tender Joint Count)

Black diamonds exhibit the individual effect of each study, whereas outer lines show the confidence intervals (CI), and the size of grey squares are proportional to the weight of each study. The blue diamond demonstrates the overall effect, the edges of which represent the CIs.

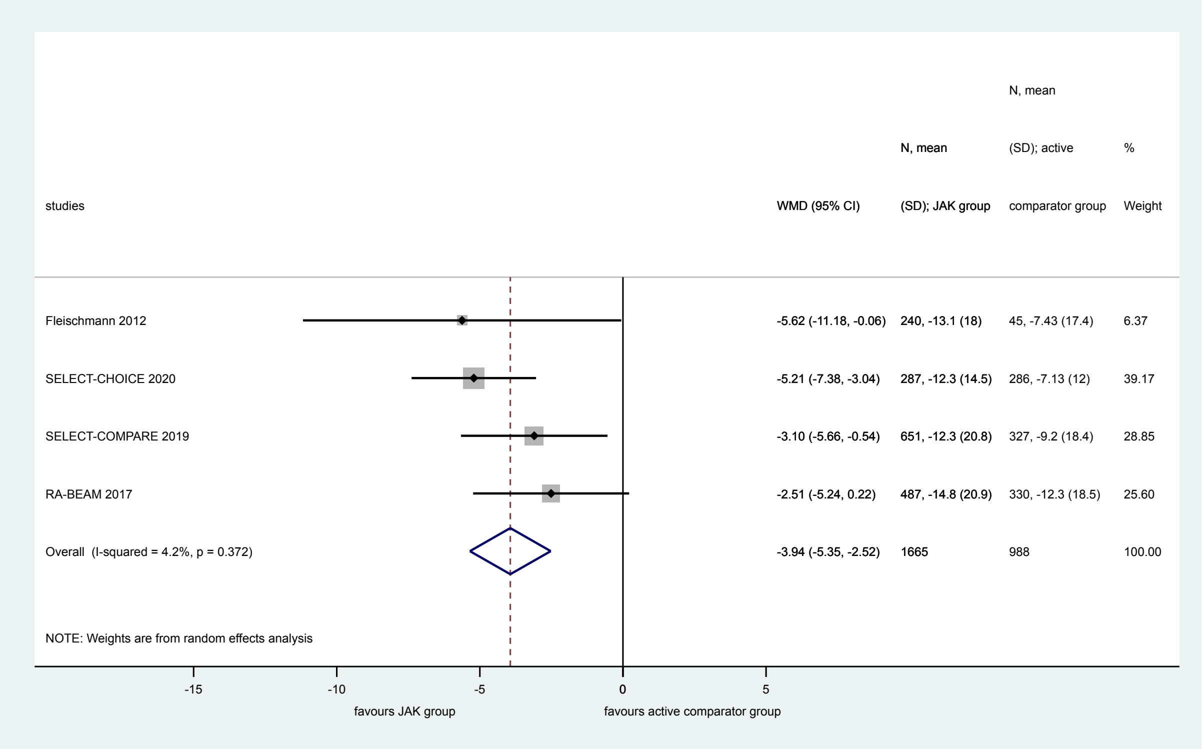

S169. Forest plot of studies comparing the LSM change from baseline in CRP value (mg/l) between patients treated with JAK inhibitors and bDMARDs within 6 months (CRP)

Black diamonds exhibit the individual effect of each study, whereas outer lines show the confidence intervals (CI), and the size of grey squares are proportional to the weight of each study. The blue diamond demonstrates the overall effect, the edges of which represent the CIs.

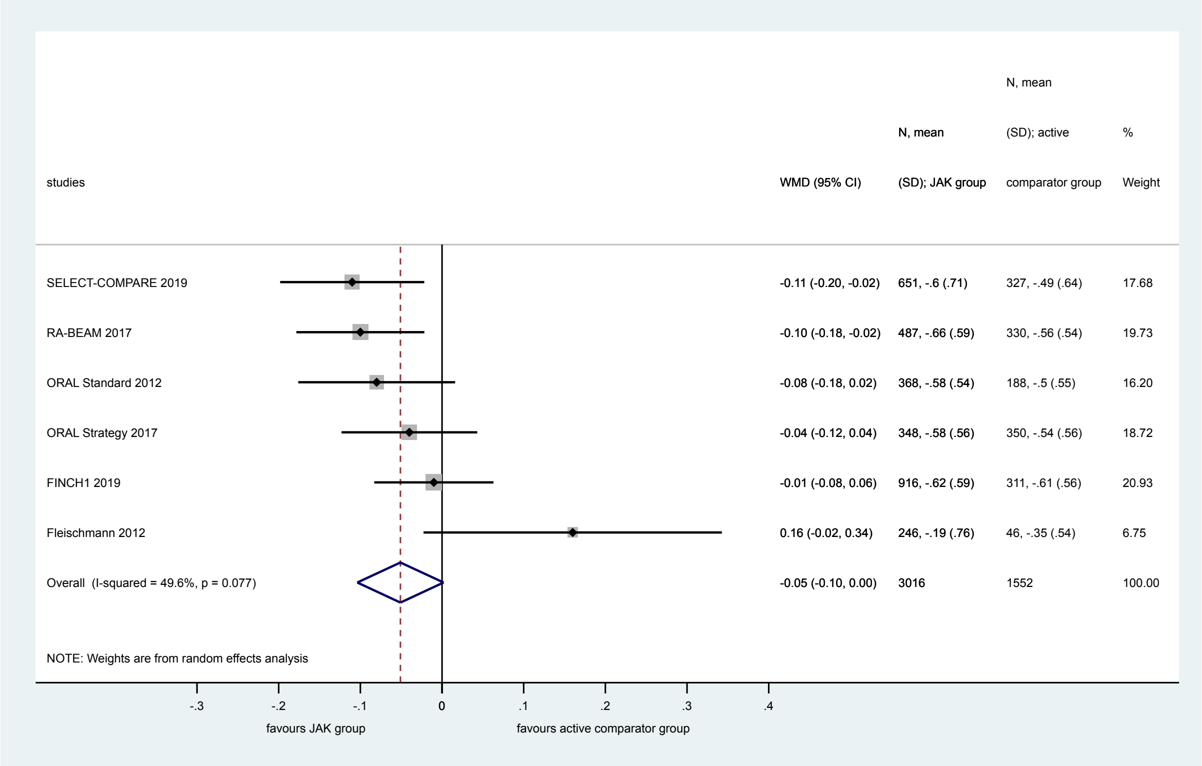

S170. Forest plot of studies comparing the LSM change from baseline in the score on HAQ-DI between patients treated with JAK inhibitors and bDMARDs within 6 months (HAQ-DI difference)

Black diamonds exhibit the individual effect of each study, whereas outer lines show the confidence intervals (CI), and the size of grey squares are proportional to the weight of each study. The blue diamond demonstrates the overall effect, the edges of which represent the CIs.

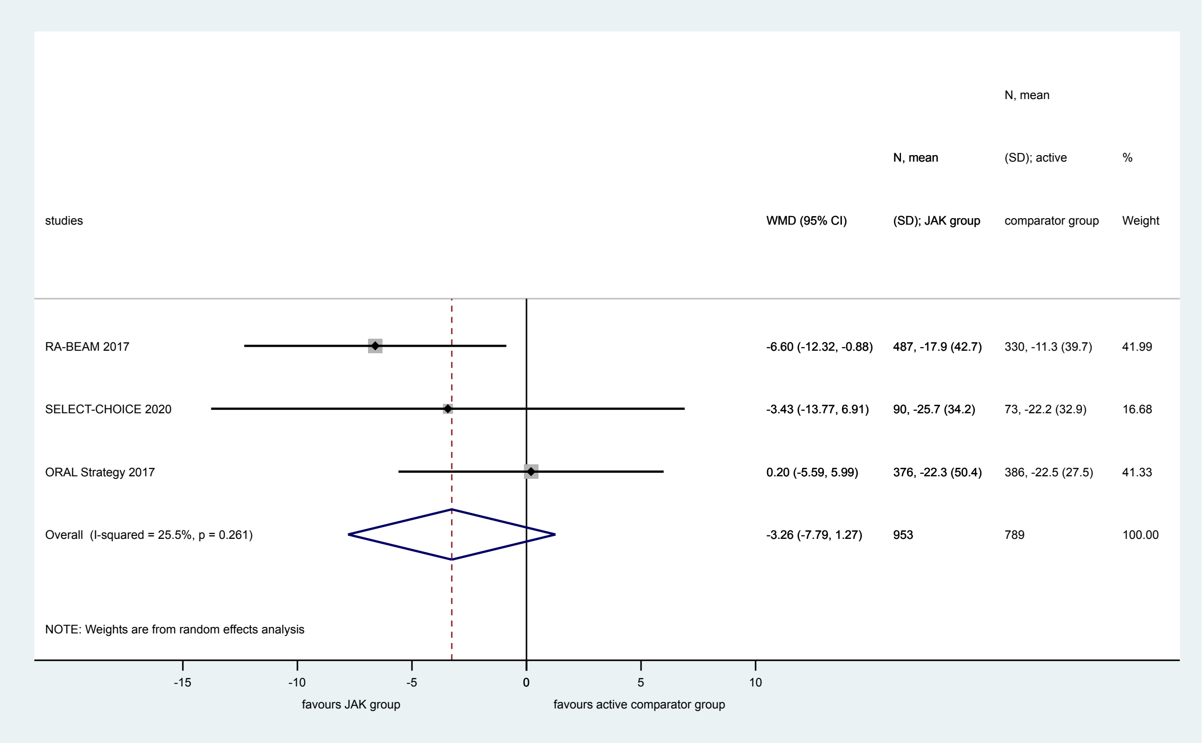

S171. Forest plot of studies comparing the LSM change from baseline in the score on WPAI assessing overall work impairment (productivity loss) between patients treated with JAK inhibitors and bDMARDs within 6 months (WPAI OWI)

Black diamonds exhibit the individual effect of each study, whereas outer lines show the confidence intervals (CI), and the size of grey squares are proportional to the weight of each study. The blue diamond demonstrates the overall effect, the edges of which represent the CIs.

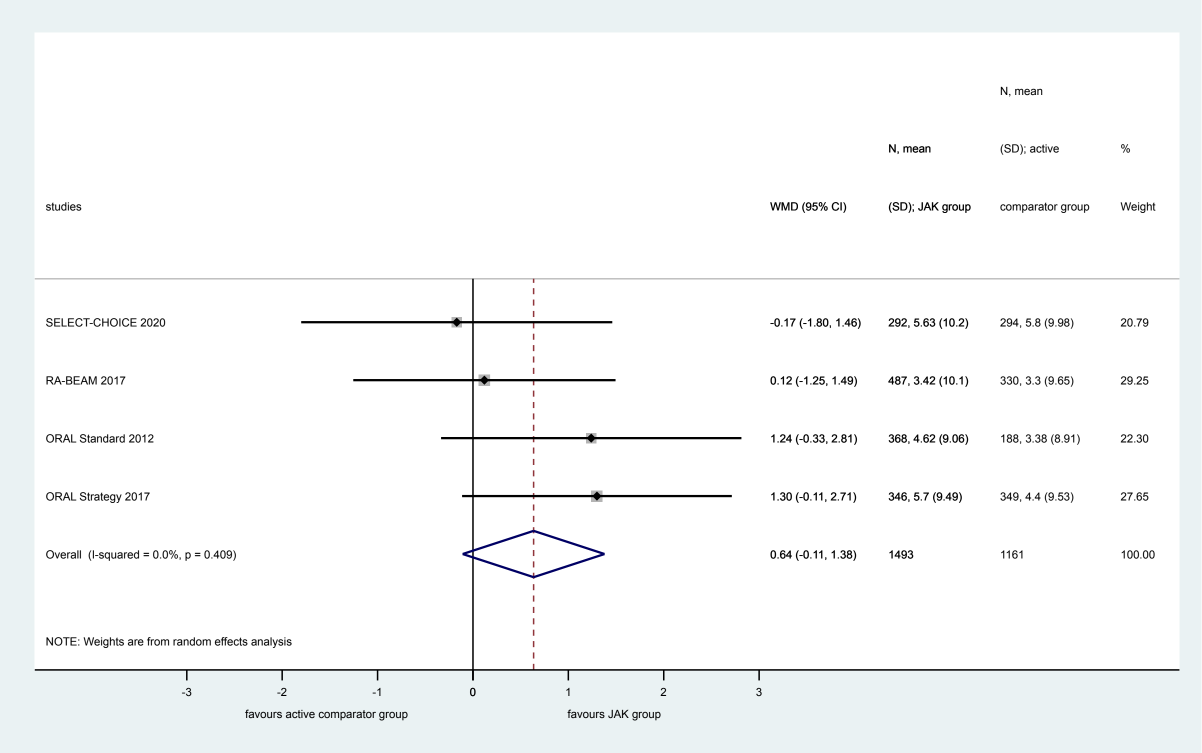

S172. Forest plot of studies comparing the LSM change from baseline in the score on SF-36 assessing the mental component score between patients treated with JAK inhibitors and bDMARDs within 6 months (SF-36 MCS)

Black diamonds exhibit the individual effect of each study, whereas outer lines show the confidence intervals (CI), and the size of grey squares are proportional to the weight of each study. The blue diamond demonstrates the overall effect, the edges of which represent the CIs.

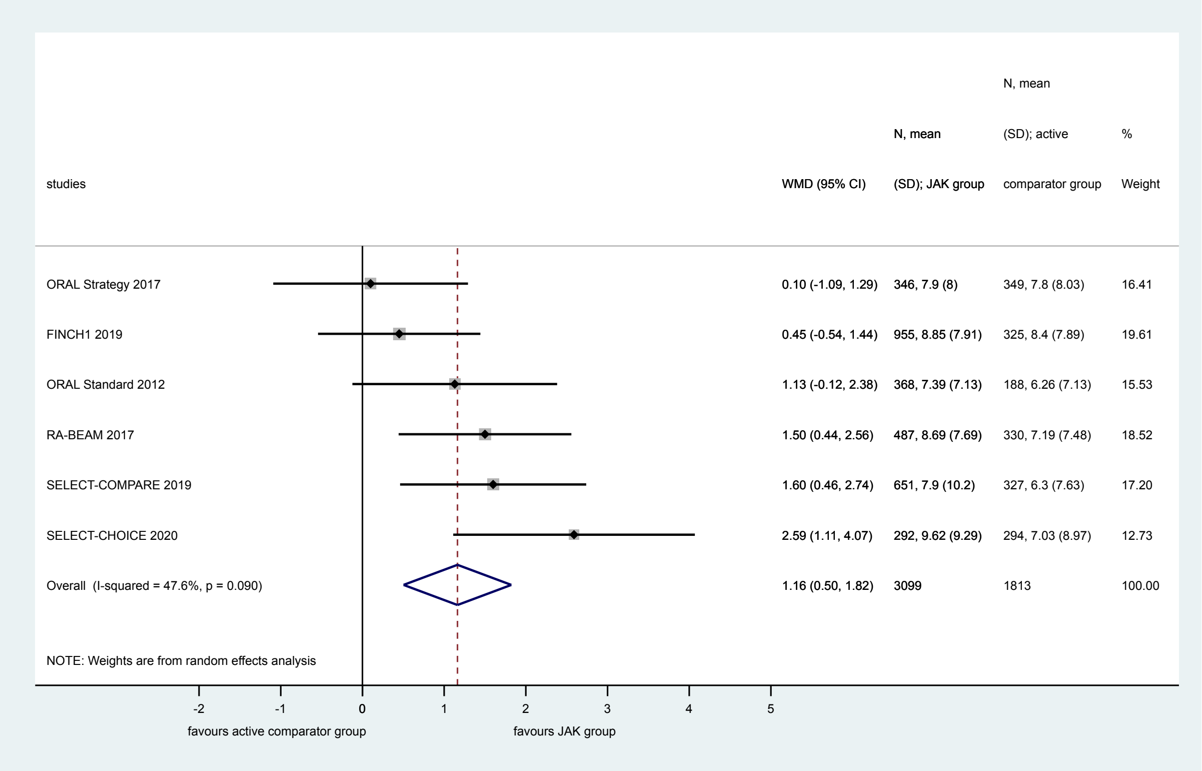

S173. Forest plot of studies comparing the LSM change from baseline in the score on SF-36 assessing the physical component score between patients treated with JAK inhibitors and bDMARDs within 6 months (SF-36 PCS)

Black diamonds exhibit the individual effect of each study, whereas outer lines show the confidence intervals (CI), and the size of grey squares are proportional to the weight of each study. The blue diamond demonstrates the overall effect, the edges of which represent the CIs.

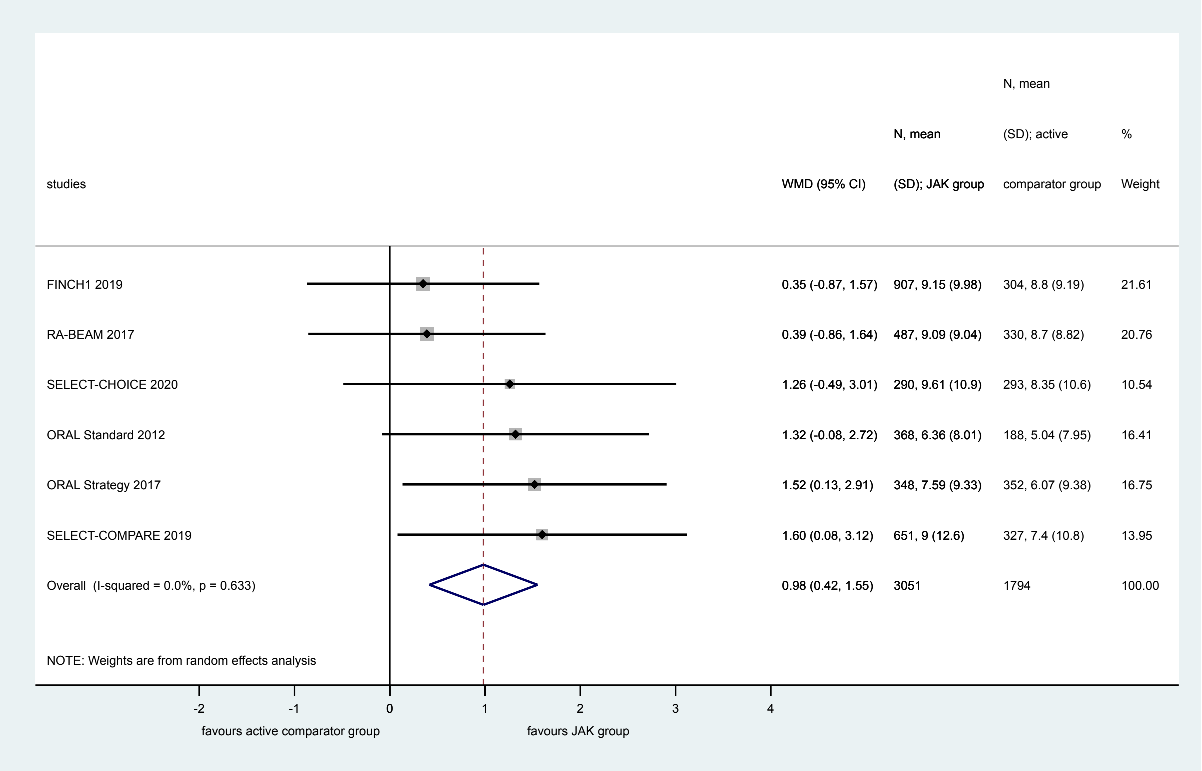

S174. Forest plot of studies comparing the LSM change from baseline in the score on FACIT-F between patients treated with JAK inhibitors and bDMARDs within 6 months (FACIT-F)

Black diamonds exhibit the individual effect of each study, whereas outer lines show the confidence intervals (CI), and the size of grey squares are proportional to the weight of each study. The blue diamond demonstrates the overall effect, the edges of which represent the CIs.

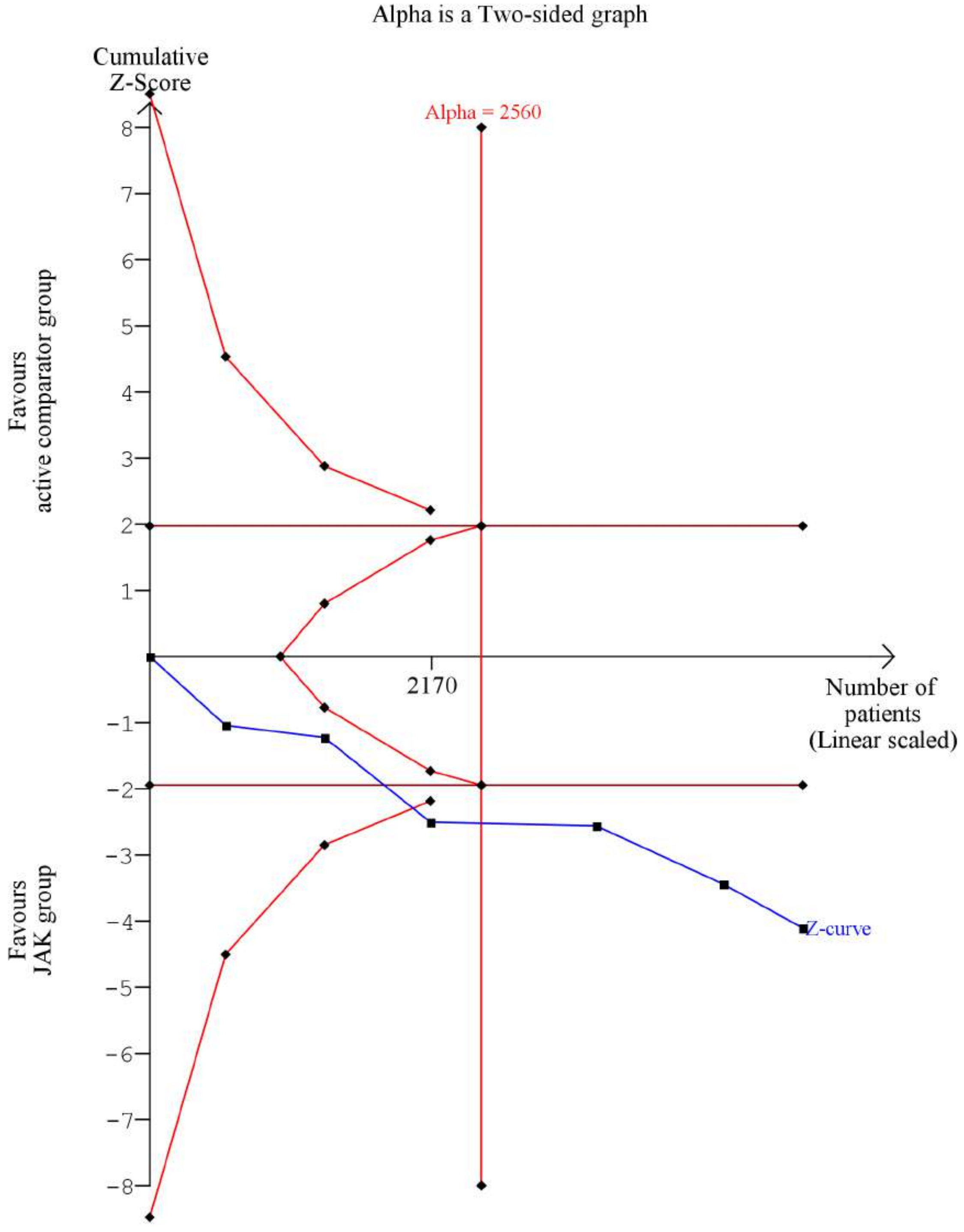

S175. TSA of ACR20 response (JAK-inhibitors vs bDMARDs)

The blue line (Z-curve) crosses the conventional boundary (brown straight line) and trial sequential boundary (red lines sloping inwards), and reaches the line of required sample size, therefore showing the statistically significant superiority of JAK inhibitors in this outcome and the addition of further studies would not influence this results.

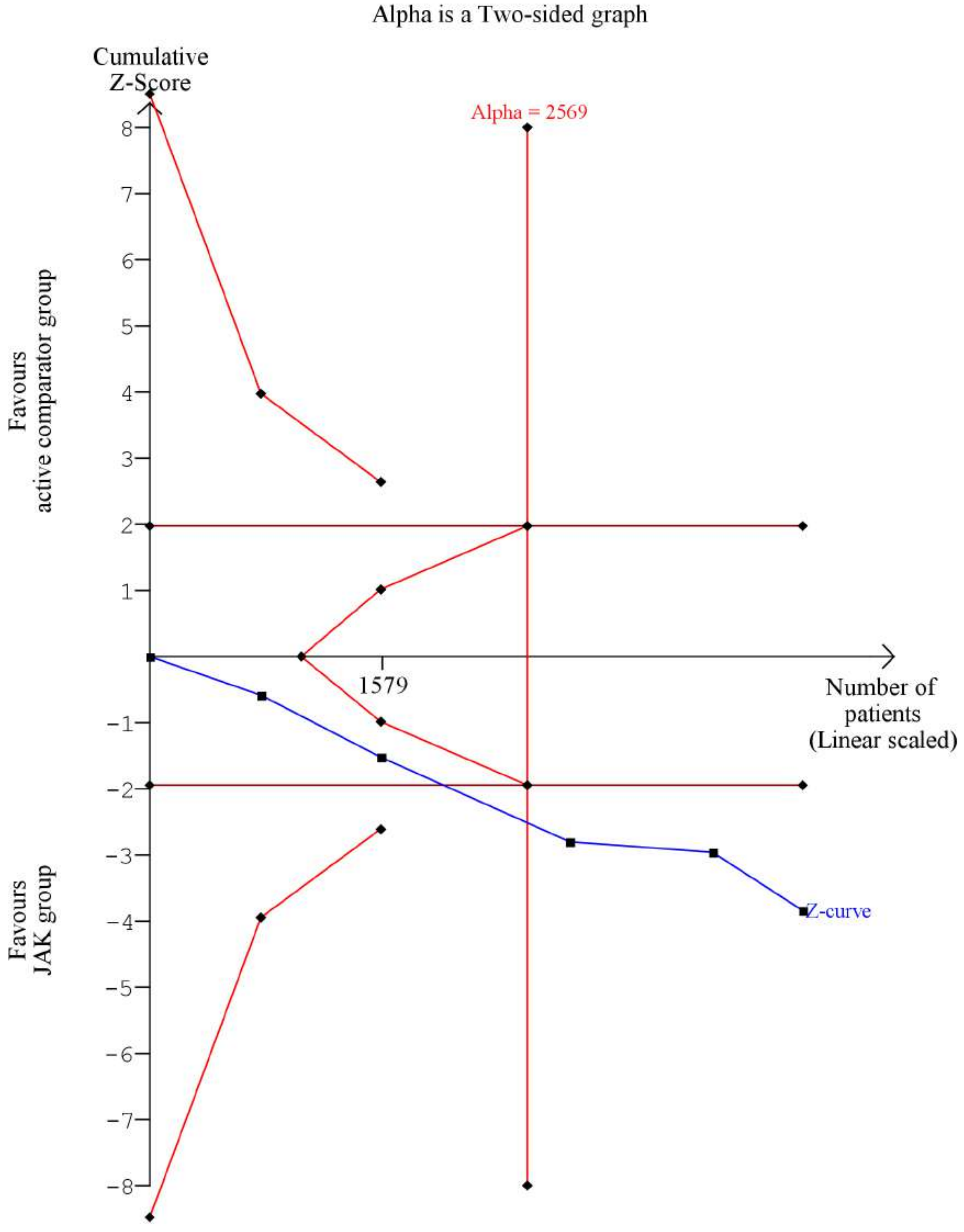

S176. TSA of ACR50 response (JAK-inhibitors vs bDMARDs)

The blue line (Z-curve) crosses the conventional boundary (brown straight line), and reaches the line of required sample size, therefore showing the statistically significant superiority of JAK inhibitors in this outcome and the addition of further studies would not influence this results.

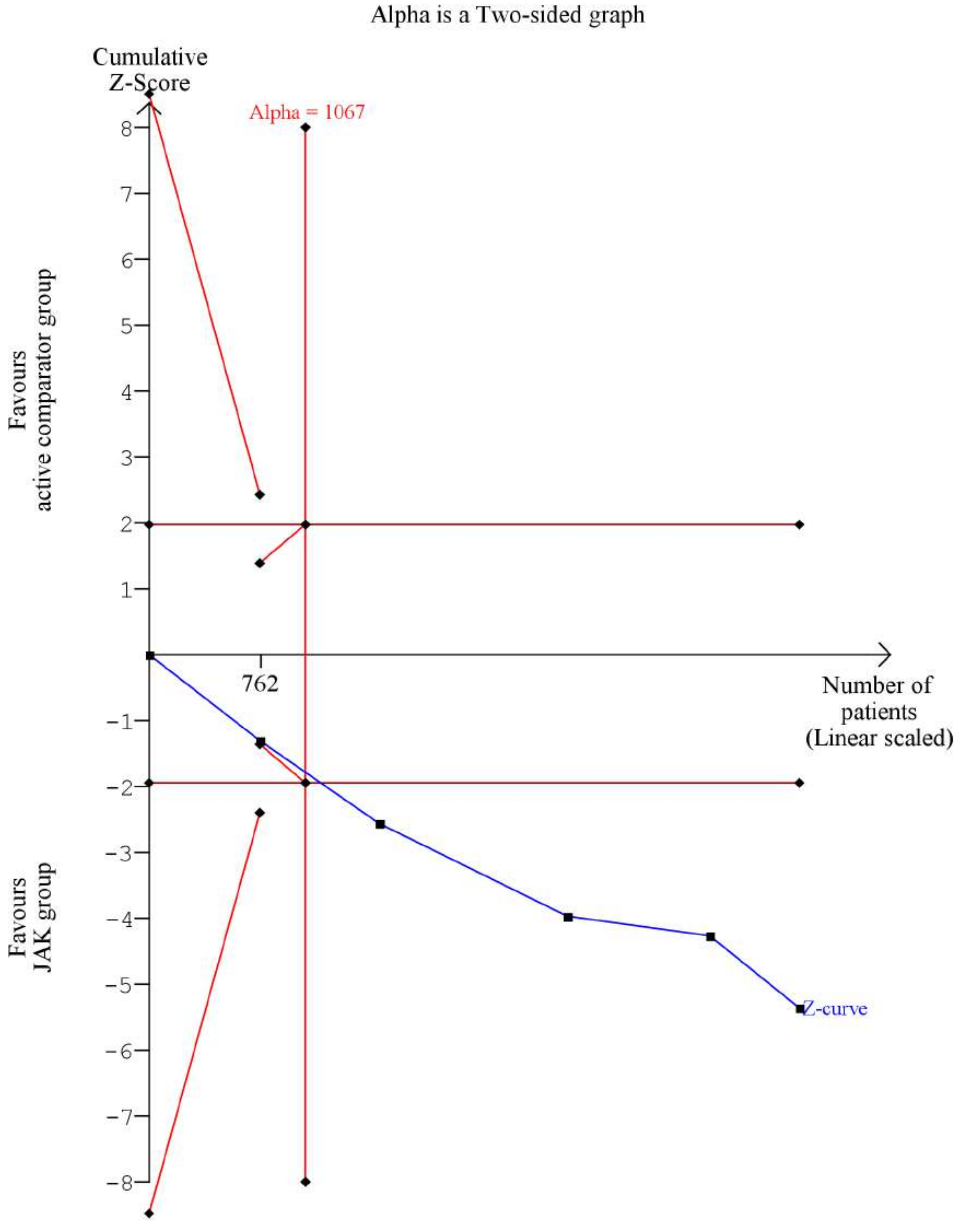

S177. TSA of ACR70 response (JAK-inhibitors vs bDMARDs)

The blue line (Z-curve) crosses the conventional boundary (brown straight line), and reaches the line of required sample size, therefore showing the statistically significant superiority of JAK inhibitors in this outcome and the addition of further studies would not influence this result

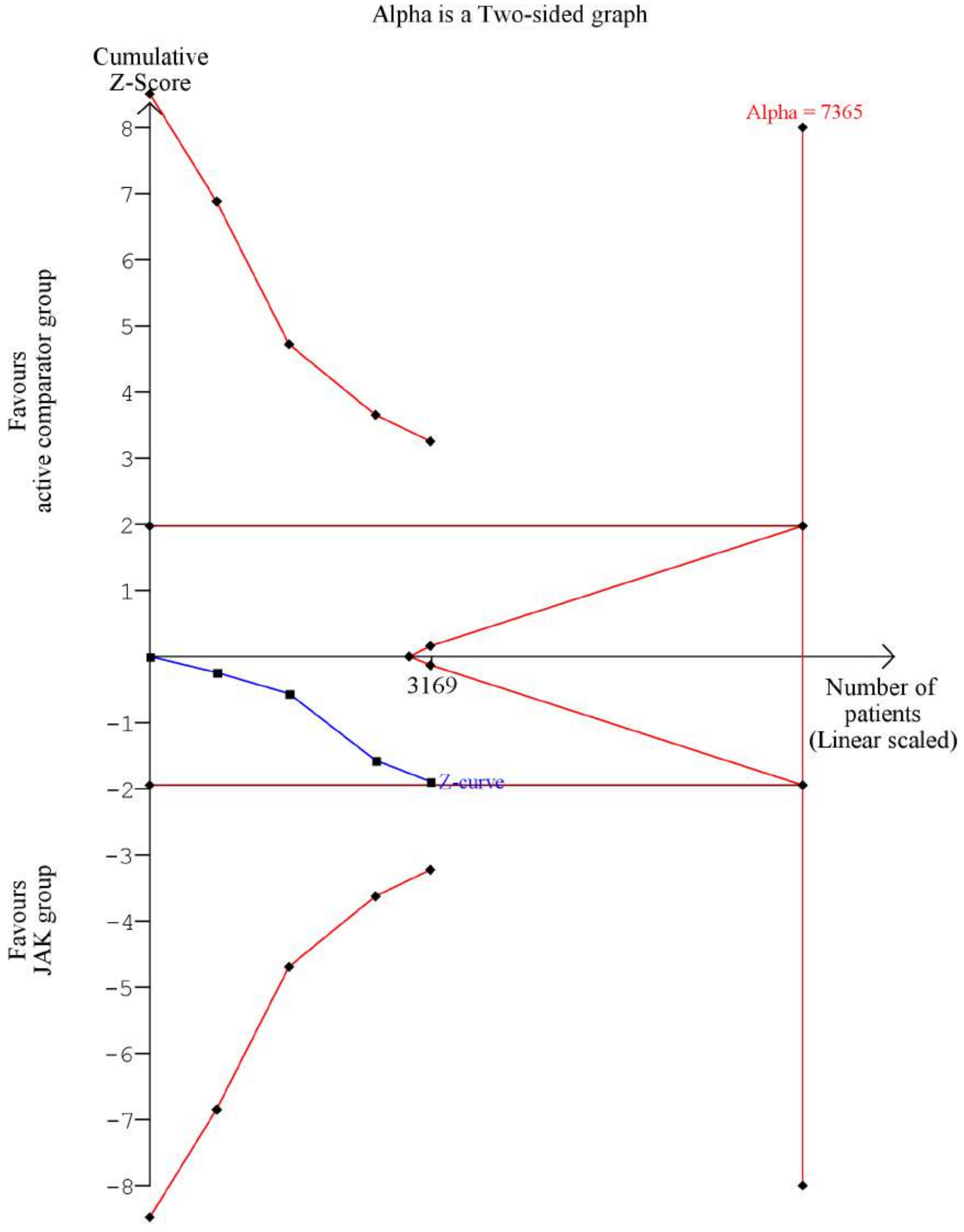

S178. TSA of CDAI remission (JAK-inhibitors vs bDMARDs)

The blue line (Z-curve) does not cross the conventional boundary (brown straight line) and does not reach the line of required sample size, therefore showing no statistically significant difference between groups in this outcome and the addition of further studies might influence these results.

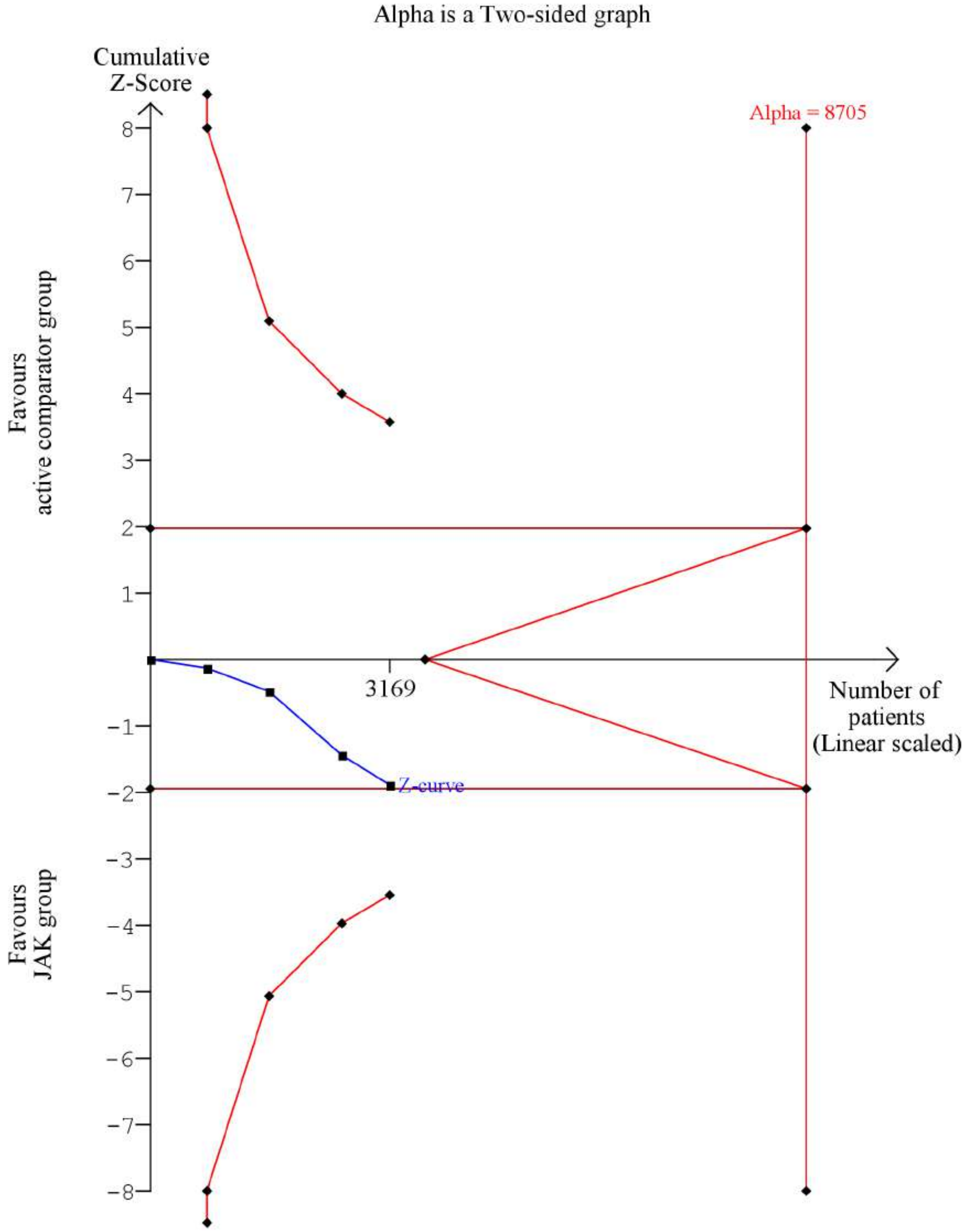

S179. TSA of SDAI remission (JAK-inhibitors vs bDMARDs)

The blue line (Z-curve) does not cross the conventional boundary (brown straight line) and does not reach the line of required sample size, therefore showing no statistically significant difference between groups in this outcome and the addition of further studies might influence these results.

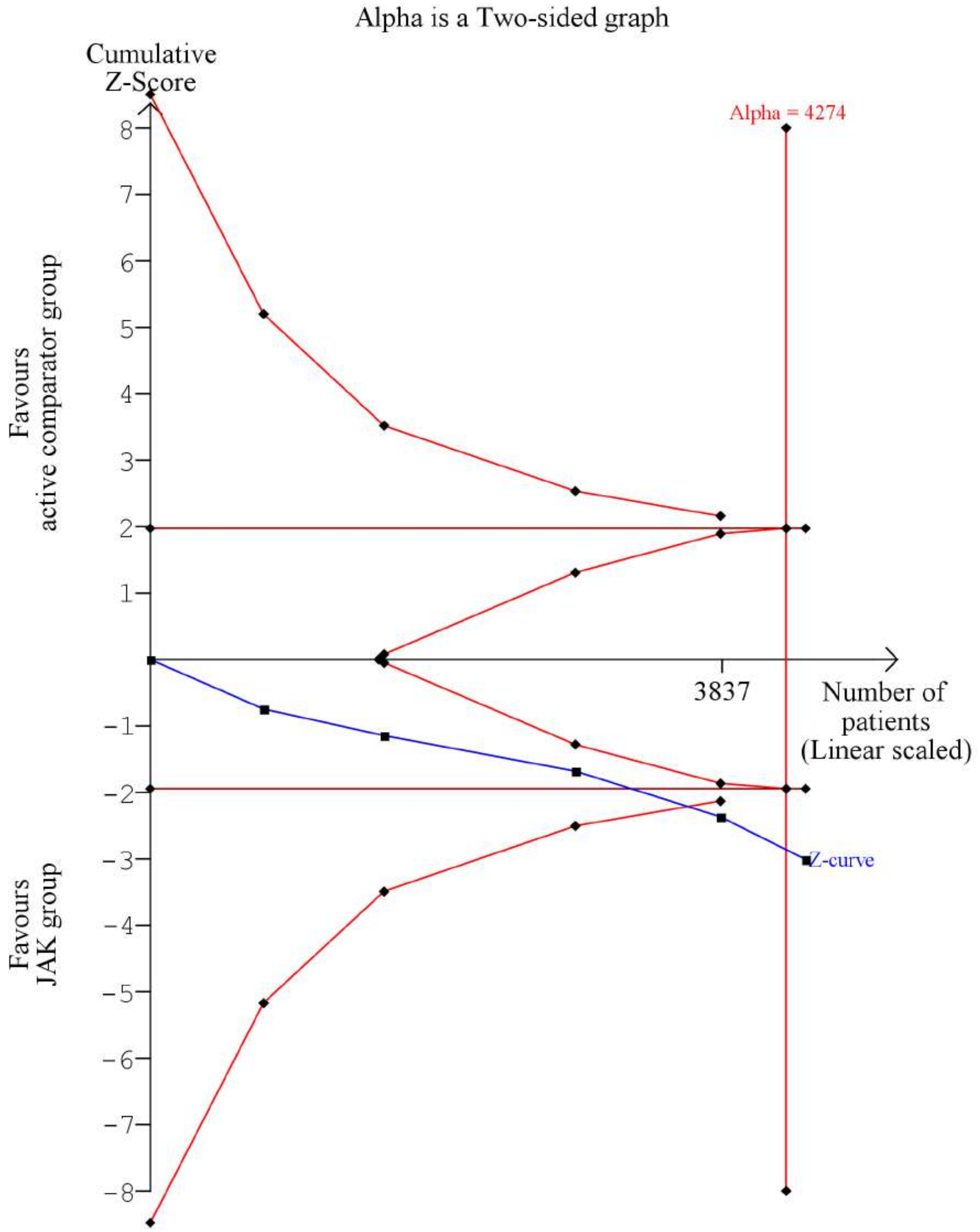

S180. TSA of DAS28-CRP remission within 6 months (JAK-inhibitors vs bDMARDs)

The blue line (Z-curve) crosses the conventional boundary (brown straight line) and trial sequential boundary (red lines sloping inwards), and reaches the line of required sample size, therefore showing the statistically significant superiority of JAK inhibitors in this outcome and the addition of further studies would not influence these results.

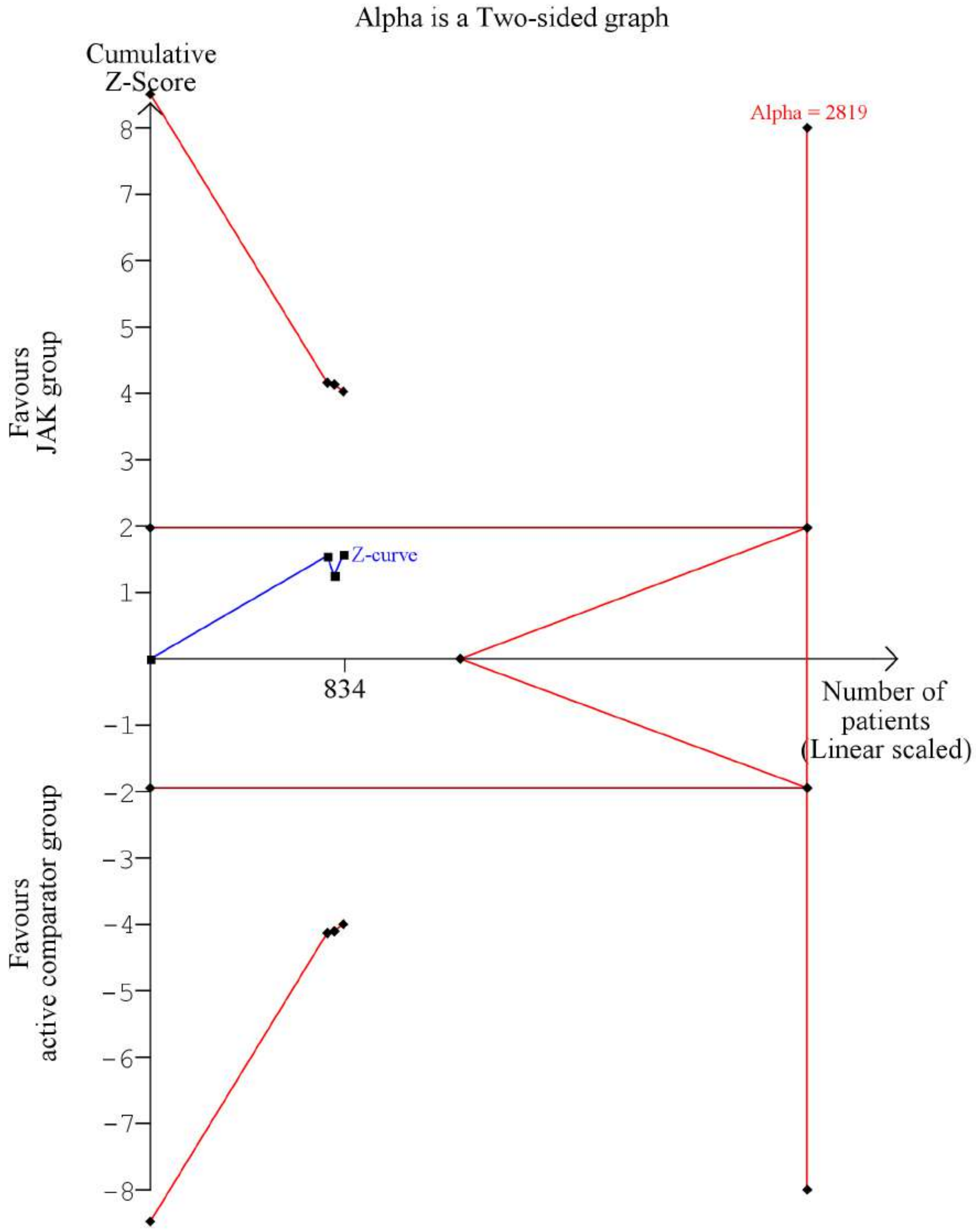

S181. TSA of DAS28-CRP remission over 6 months (JAK-inhibitors vs bDMARDs)

The blue line (Z-curve) does not cross the conventional boundary (brown straight line) and does not reach the line of required sample size, therefore showing no statistically significant difference between groups in this outcome and the addition of further studies might influence these results.

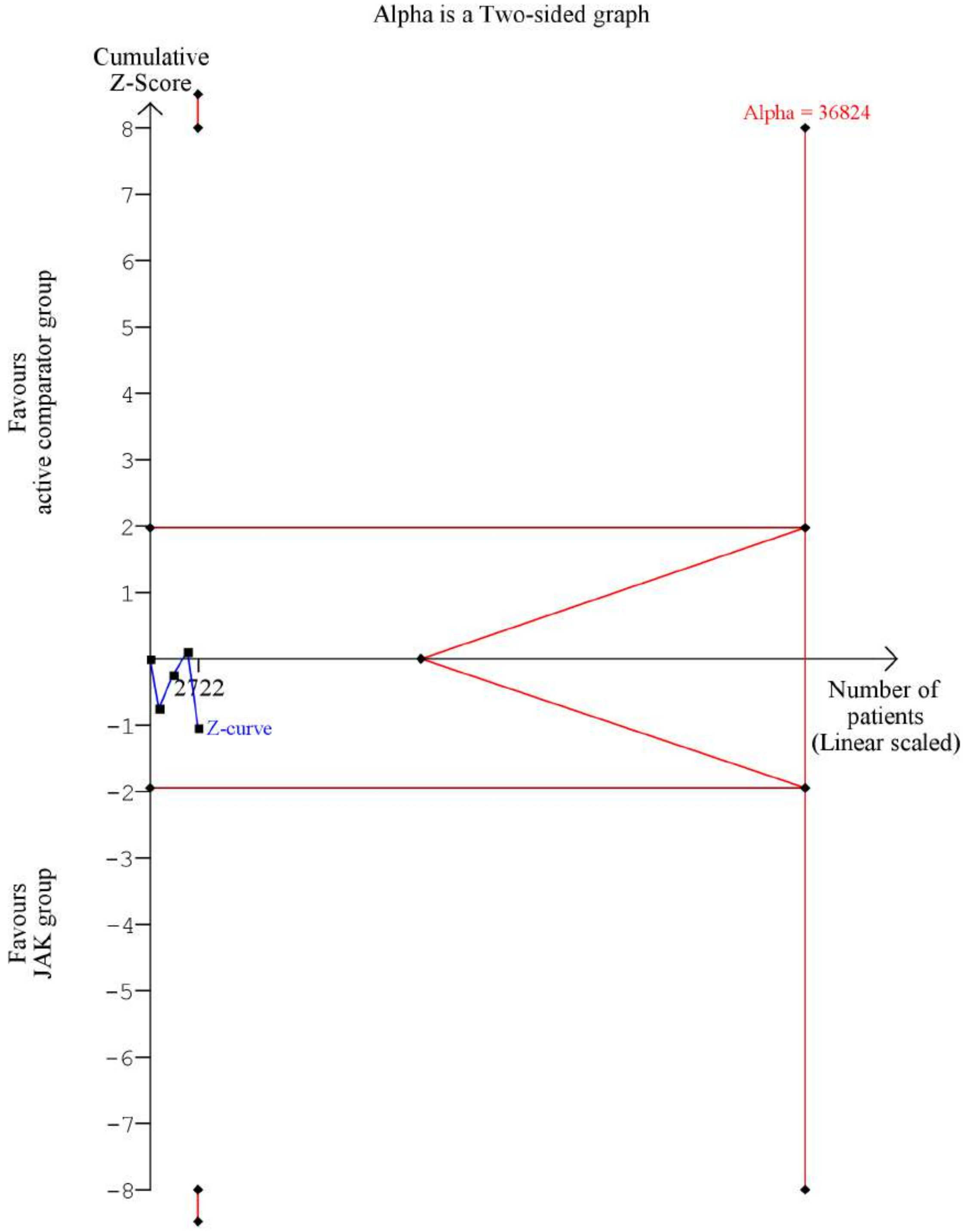

S182. TSA of DAS28-ESR remission (JAK-inhibitors vs bDMARDs)

The blue line (Z-curve) does not cross the conventional boundary (brown straight line) and does not reach the line of required sample size, therefore showing no statistically significant difference between groups in this outcome and the addition of further studies might influence these results.

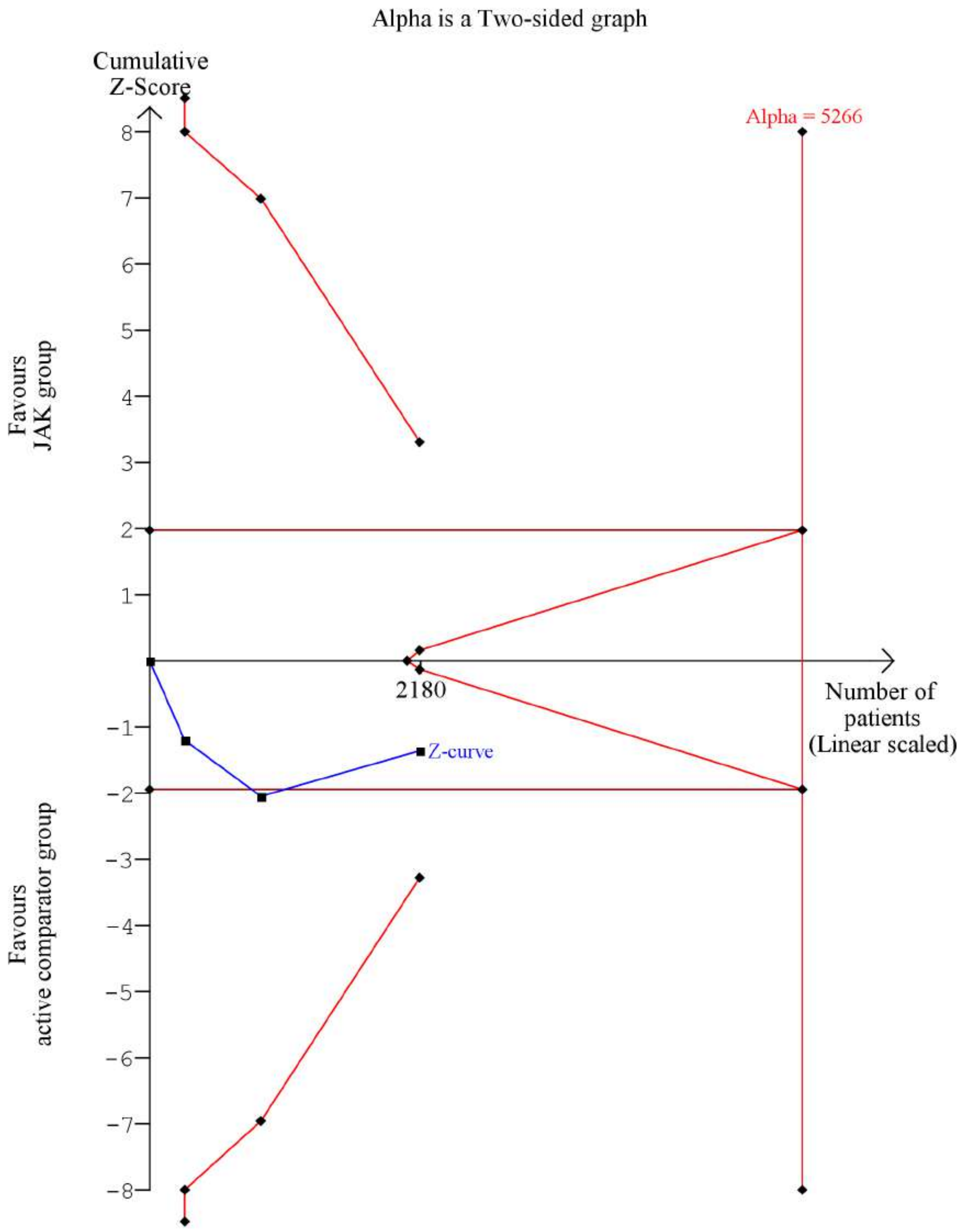

S183. TSA of serious side effects (JAK-inhibitors vs bDMARDs)

The blue line (Z-curve) does not cross the conventional boundary (brown straight line) and does not reach the line of required sample size, therefore showing no statistically significant difference between groups in this outcome and the addition of further studies might influence these results.

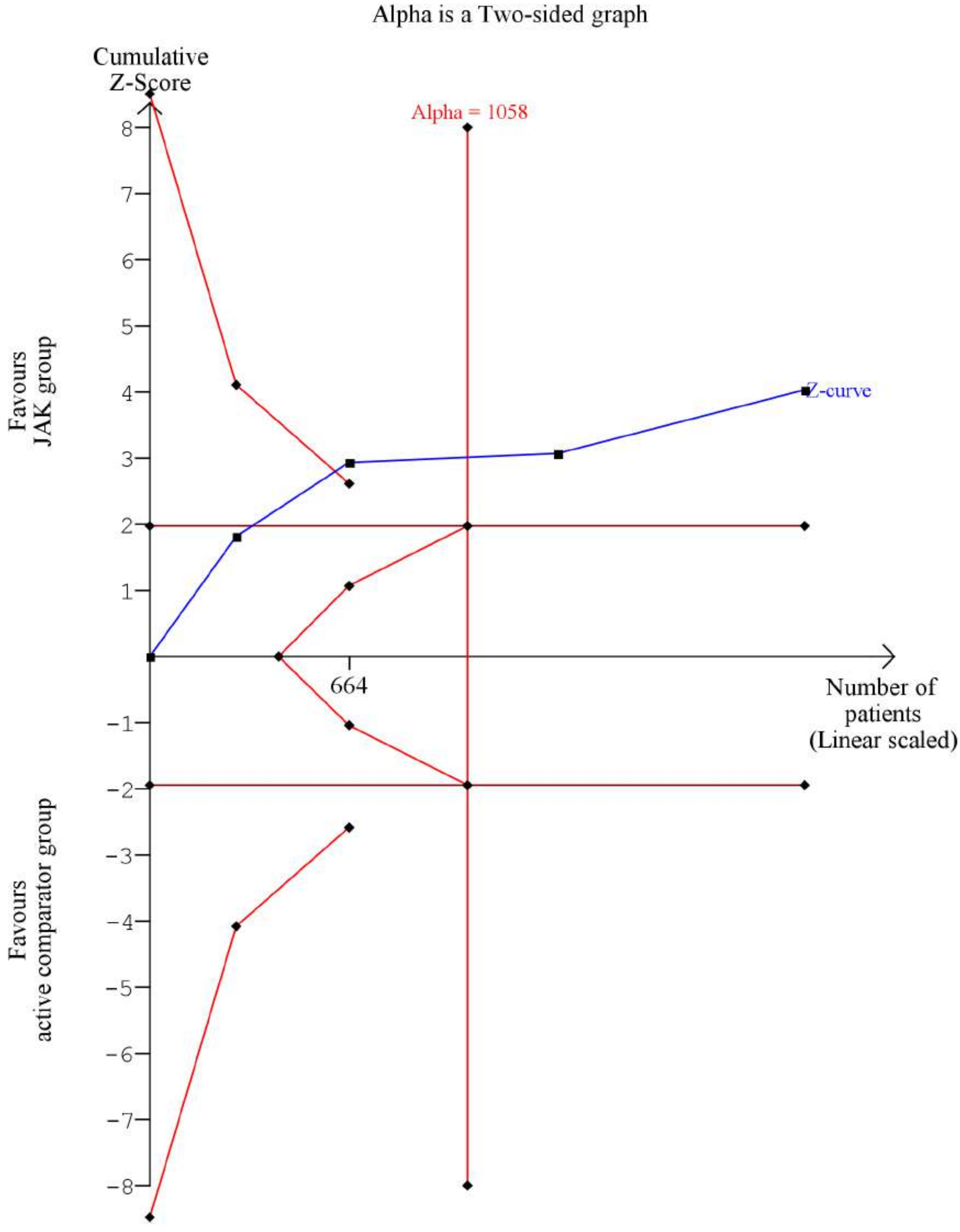

S184. TSA of Pain VAS (JAK-inhibitors vs bDMARDs)

The blue line (Z-curve) crosses the conventional boundary (brown straight line) and trial sequential boundary (red lines sloping inwards), and reaches the line of required sample size, therefore showing the statistically significant superiority of JAK inhibitors in this outcome and the addition of further studies would not influence these results.

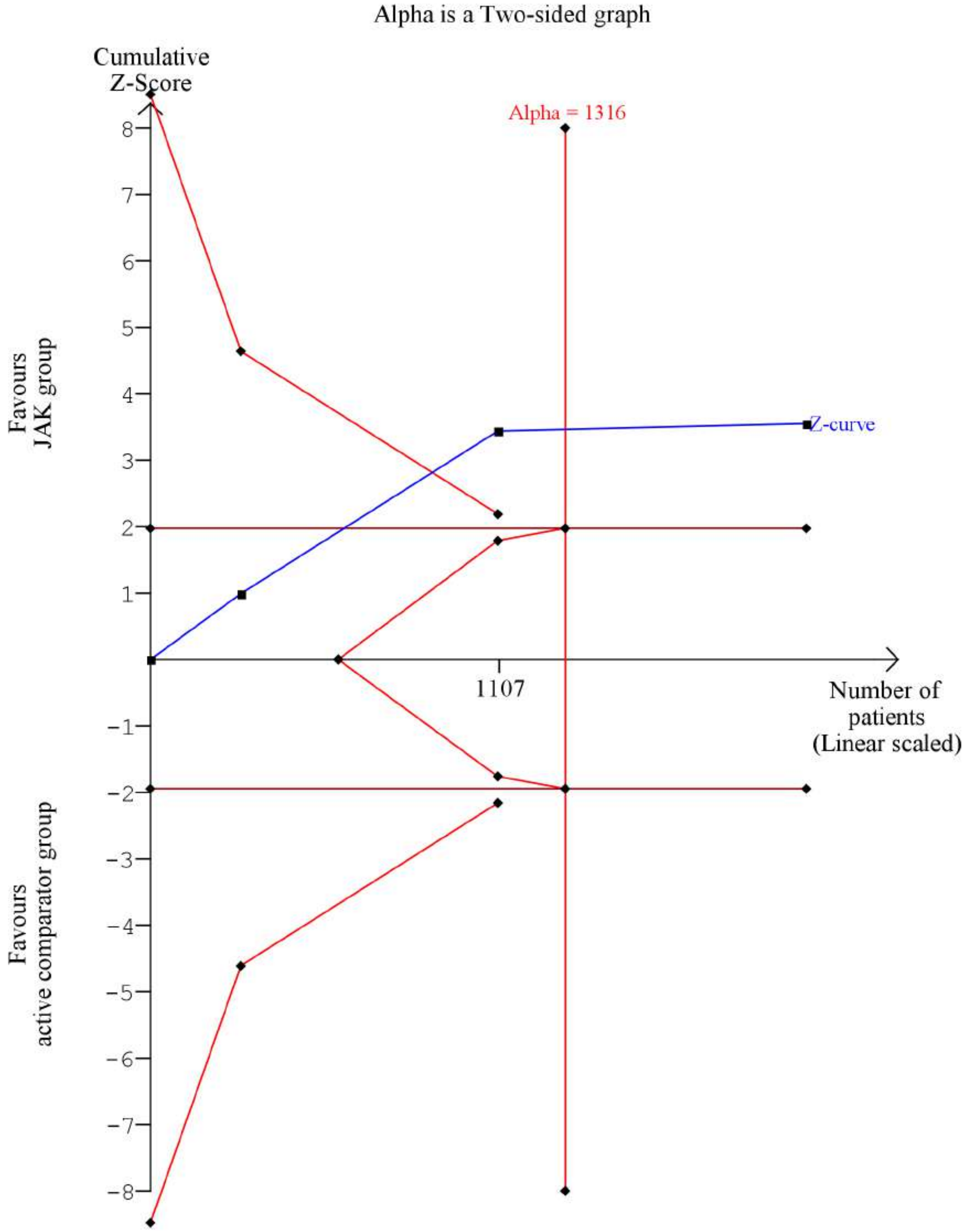

S185. TSA of PGA VAS (JAK-inhibitors vs bDMARDs)

The blue line (Z-curve) crosses the conventional boundary (brown straight line) and trial sequential boundary (red lines sloping inwards), and reaches the line of required sample size, therefore showing the statistically significant superiority of JAK inhibitors in this outcome and the addition of further studies would not influence these results.

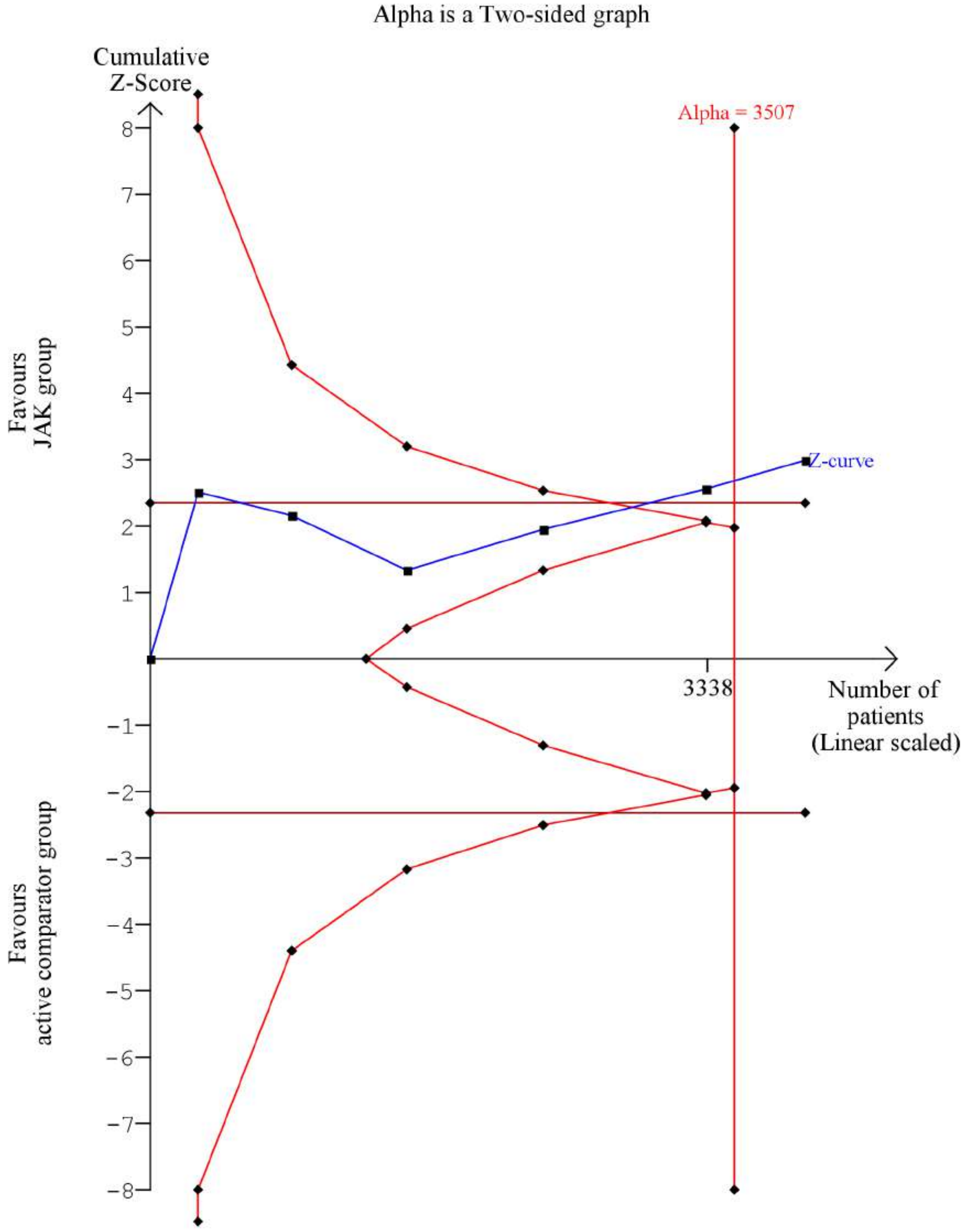

S186. TSA of PtGA VAS (JAK-inhibitors vs bDMARDs)

The blue line (Z-curve) crosses the conventional boundary (brown straight line) and trial sequential boundary (red lines sloping inwards), and reaches the line of required sample size, therefore showing the statistically significant superiority of JAK inhibitors in this outcome and the addition of further studies would not influence these results.

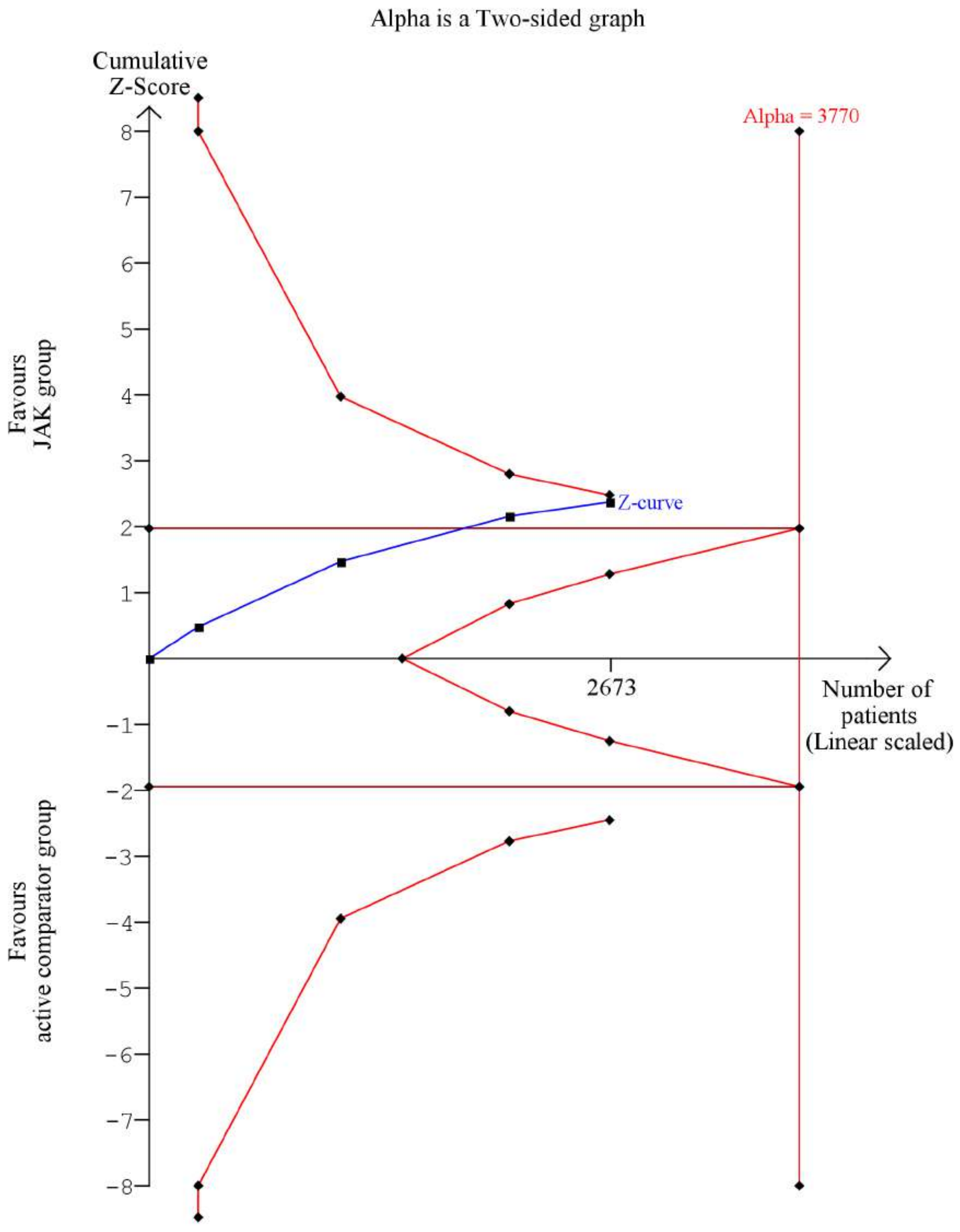

S187. TSA of Swollen Joint Count (JAK-inhibitors vs bDMARDs)

The blue line (Z-curve) crosses the conventional boundary (brown straight line) but does not reach the line of required sample size, therefore showing the statistically significant superiority of JAK inhibitors in this outcome but the addition of further studies might influence these results.

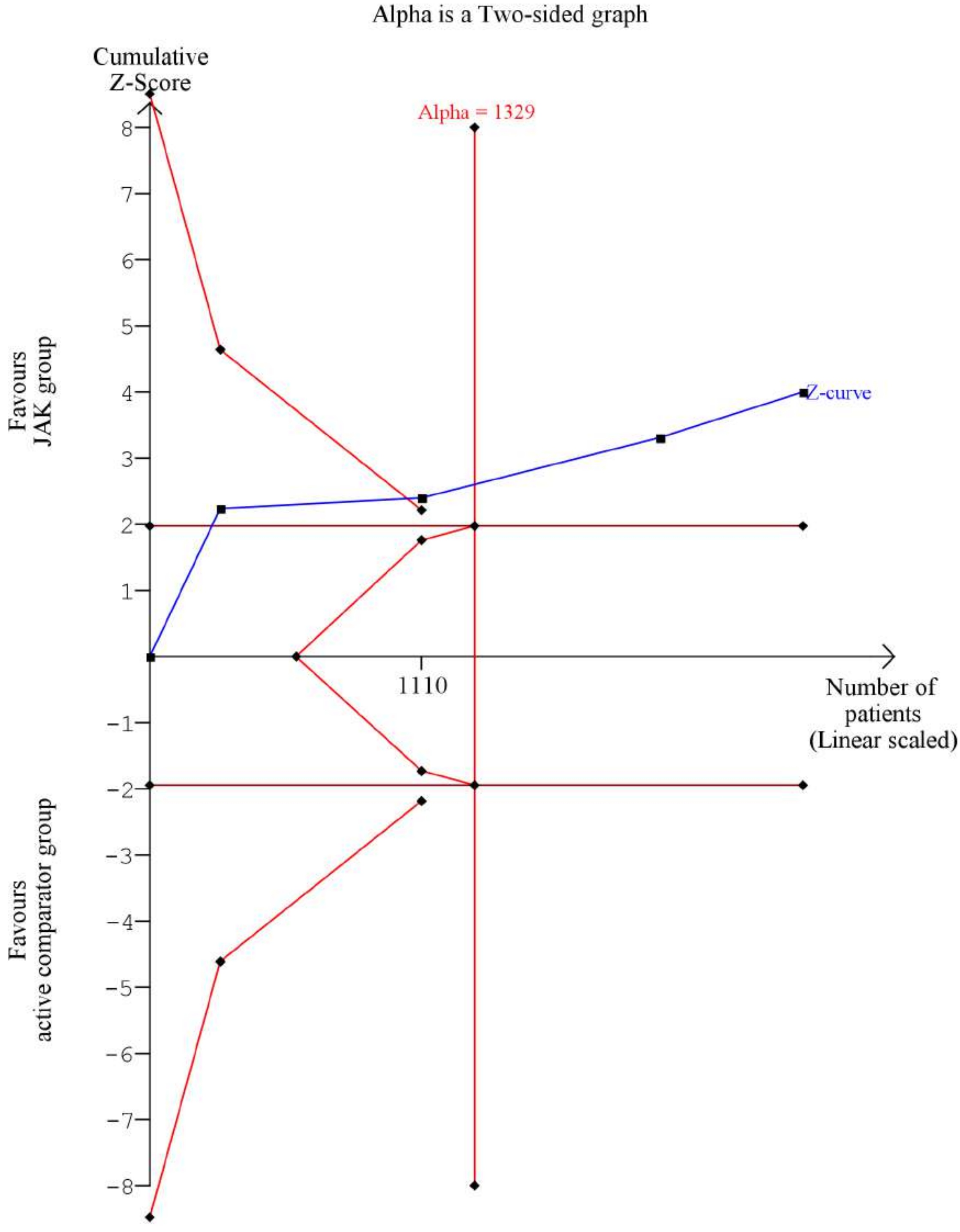

S188. TSA of Tender Joint Count (JAK-inhibitors vs bDMARDs)

The blue line (Z-curve) crosses the conventional boundary (brown straight line) and trial sequential boundary (red lines sloping inwards), and reaches the line of required sample size, therefore showing the statistically significant superiority of JAK inhibitors in this outcome and the addition of further studies would not influence these results.

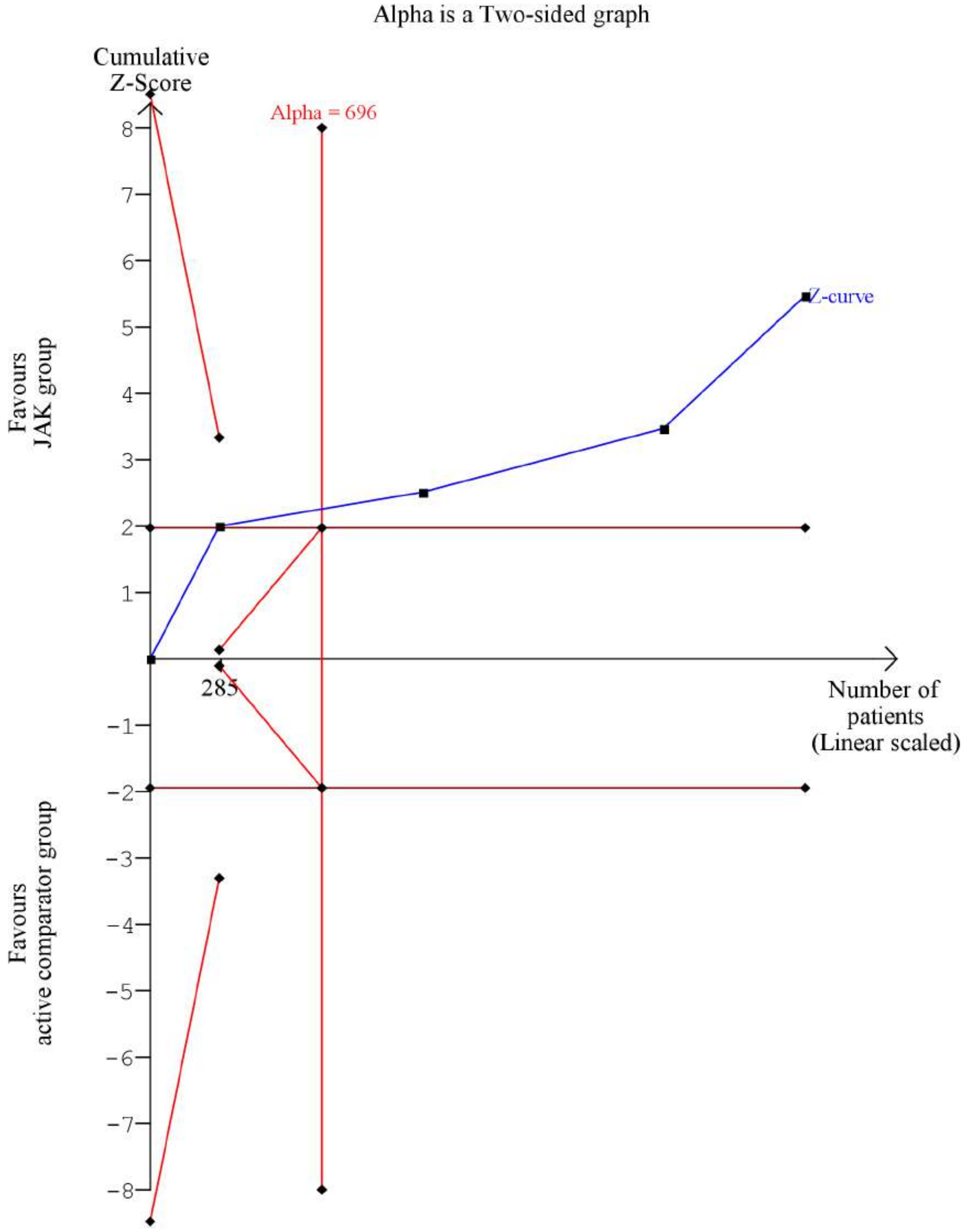

S189. TSA of CRP (mg/l) (JAK-inhibitors vs bDMARDs)

The blue line (Z-curve) crosses the conventional boundary (brown straight line), and reaches the line of required sample size, therefore showing the statistically significant superiority of JAK inhibitors in this outcome and the addition of further studies would not influence this result.

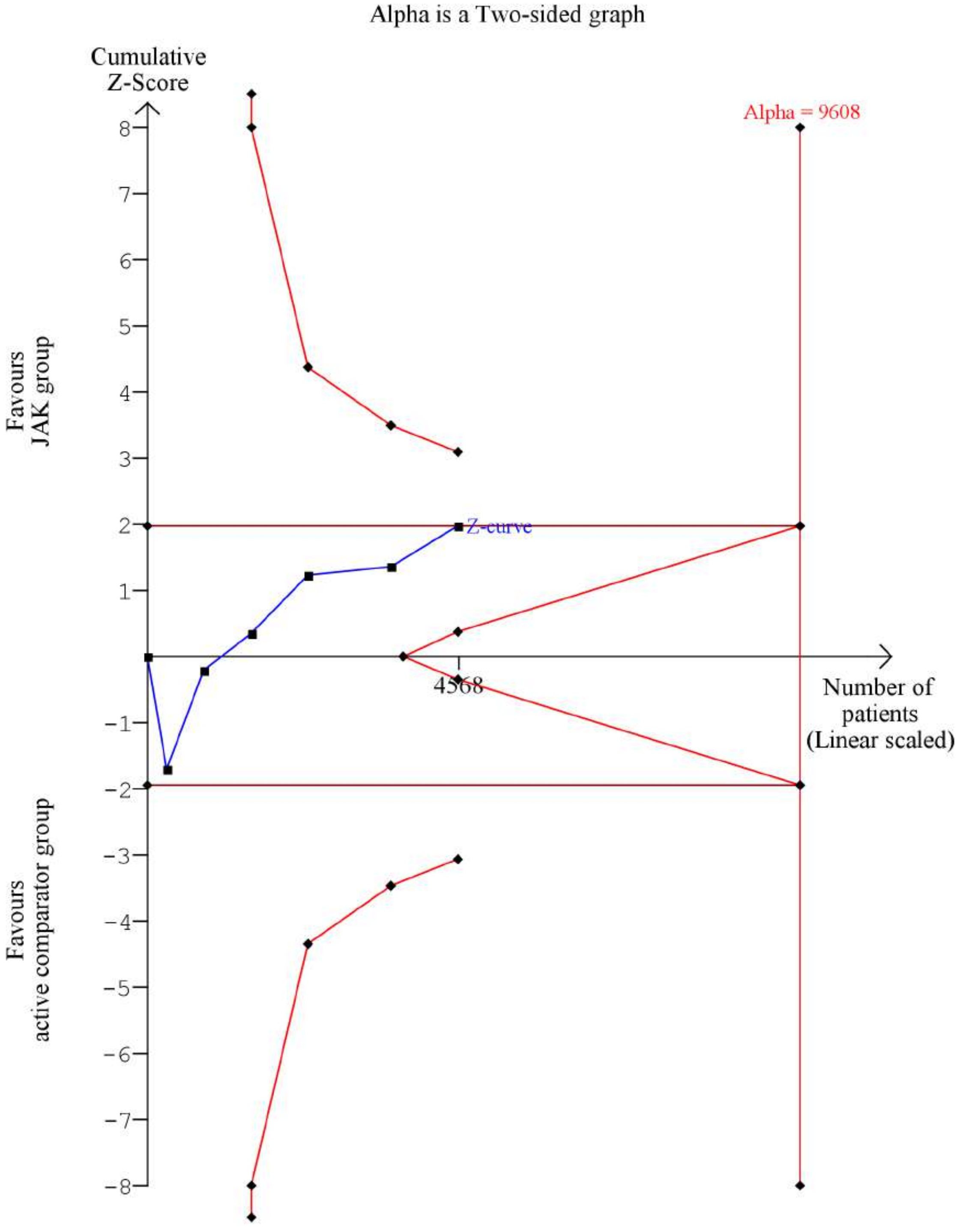

S190. TSA of HAQ-DI difference (JAK-inhibitors vs bDMARDs)

The blue line (Z-curve) does not cross the conventional boundary (brown straight line) and does not reach the line of required sample size, therefore showing no statistically significant difference between groups in this outcome and the addition of further studies might influence these results.

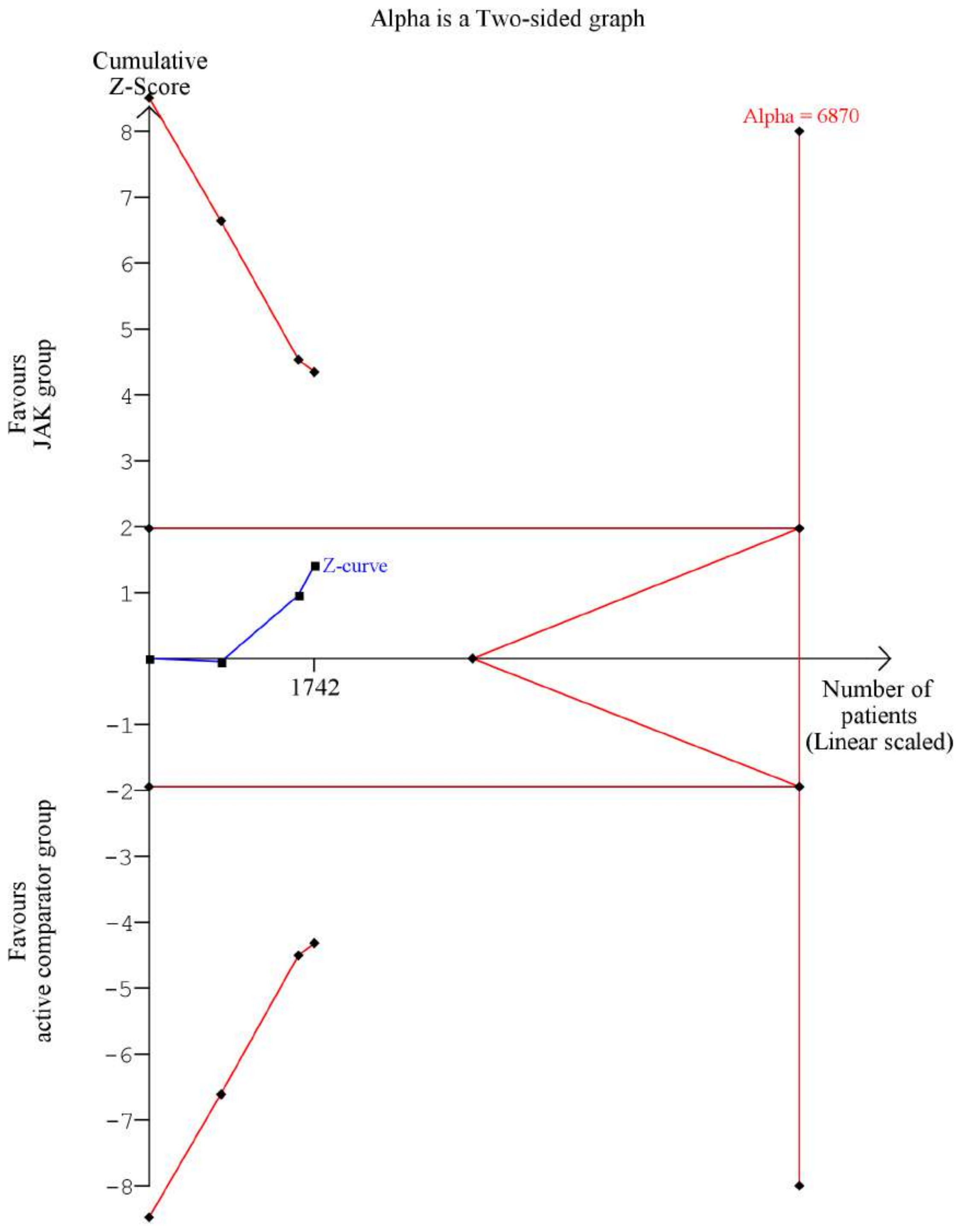

S191. TSA of WPAI OWI (JAK-inhibitors vs bDMARDs)

The blue line (Z-curve) does not cross the conventional boundary (brown straight line) and does not reach the line of required sample size, therefore showing no statistically significant difference between groups in this outcome and the addition of further studies might influence these results.

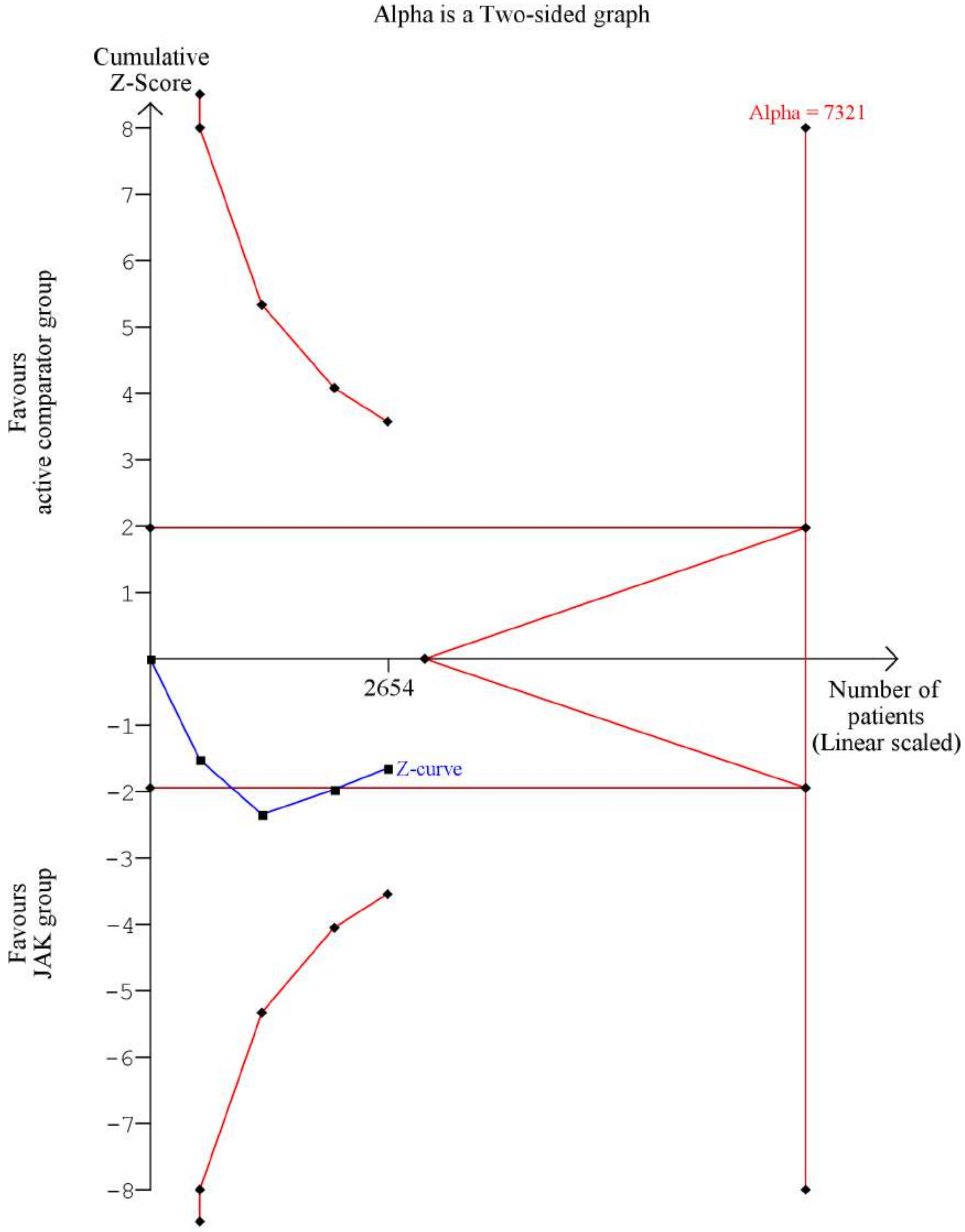

S192. TSA of SF-36 MCS (JAK-inhibitors vs bDMARDs)

The blue line (Z-curve) does not cross the conventional boundary (brown straight line) and does not reach the line of required sample size, therefore showing no statistically significant difference between groups in this outcome and the addition of further studies might influence these results.

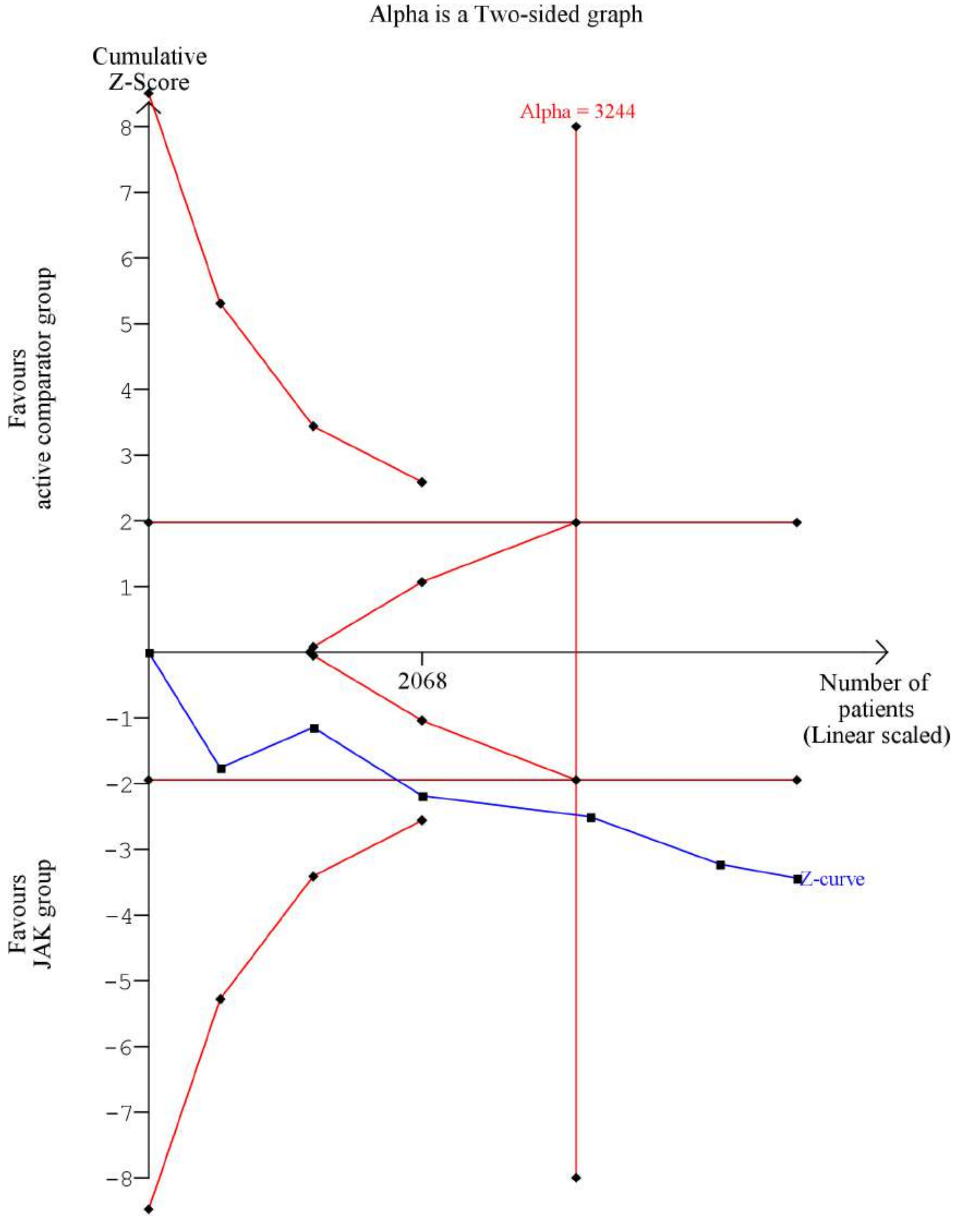

S193. TSA of SF-36 PCS (JAK-inhibitors vs bDMARDs)

The blue line (Z-curve) crosses the conventional boundary (brown straight line) and trial sequential boundary (red lines sloping inwards), and reaches the line of required sample size, therefore showing the statistically significant superiority of JAK inhibitors in this outcome and the addition of further studies would not influence these results.

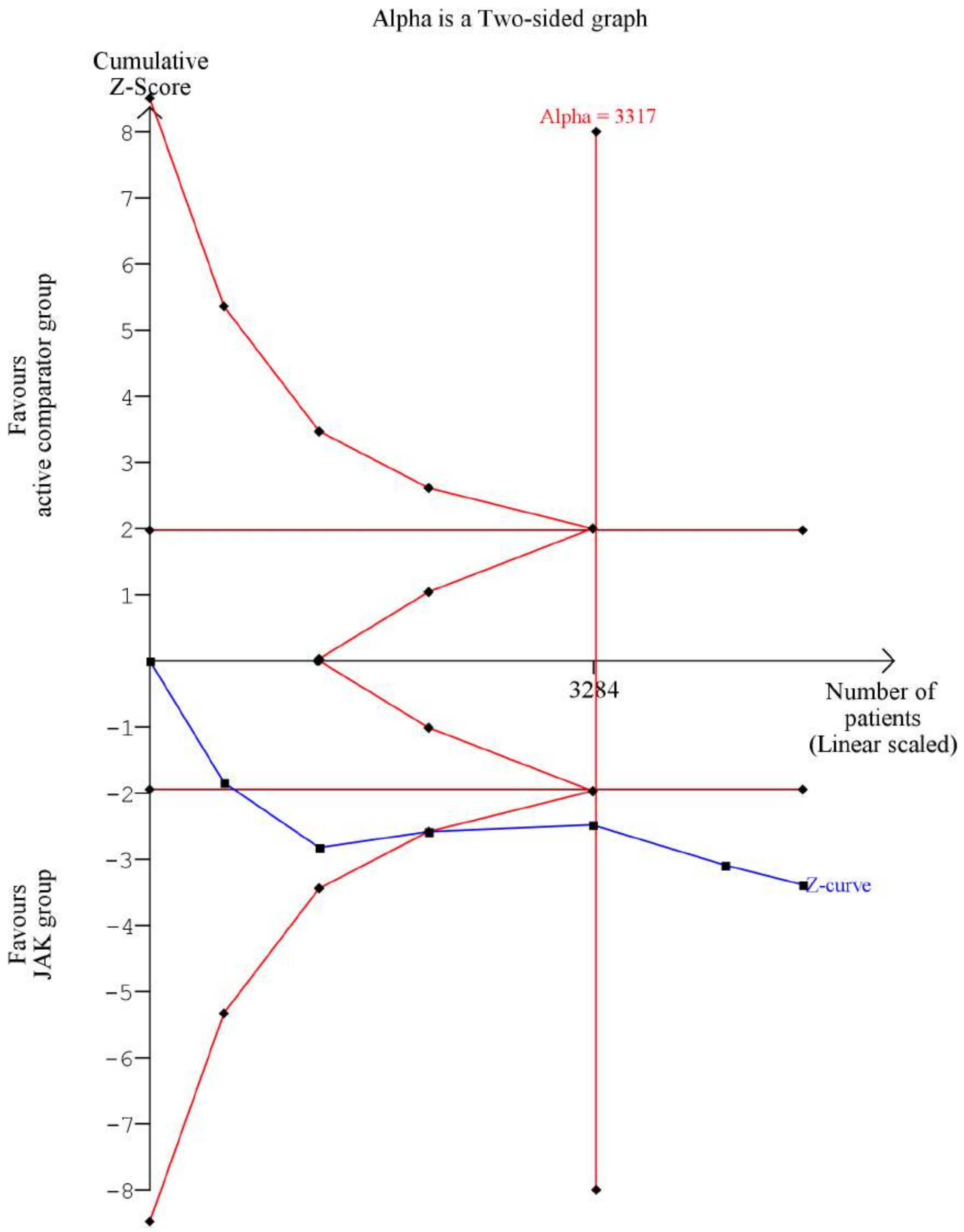

S194. TSA of FACIT-F (JAK-inhibitors vs bDMARDs)

The blue line (Z-curve) crosses the conventional boundary (brown straight line) and trial sequential boundary (red lines sloping inwards), and reaches the line of required sample size, therefore showing the statistically significant superiority of JAK inhibitors in this outcome and the addition of further studies would not influence these results.

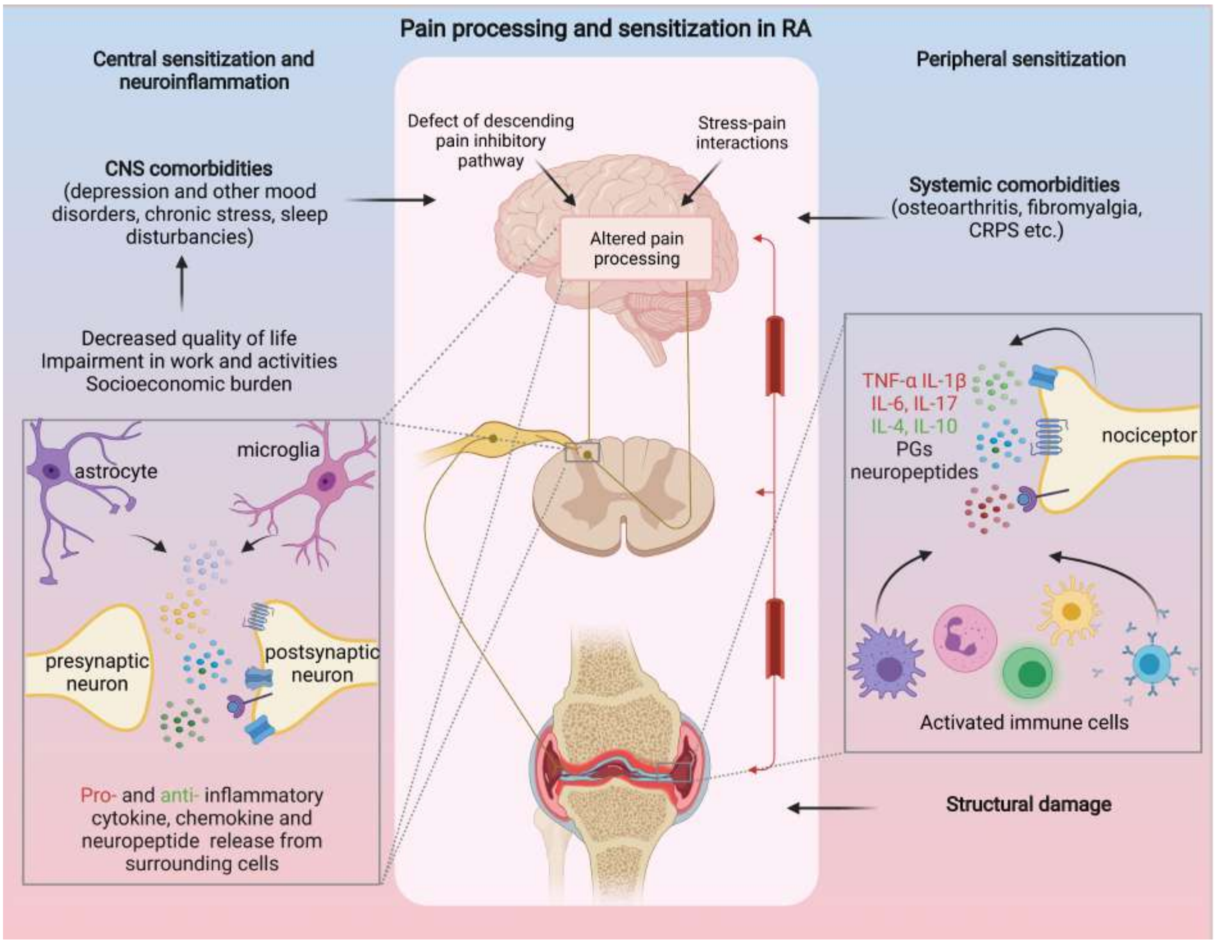

S195. Hypothesized inflammatory and non-inflammatory factors contributing to induce and maintain pain in RA.

Immune cells e.g. macrophages, neutrophil granulocytes, dendritic -, T- and B cells recruited in the joints produce several inflammatory cytokines (TNFa, IL-1b, IL-6, IL-17, IL-4, IL-10) that can activate their receptors on nociceptors leading to peripheral sensitization. Non-neuronal structures such as astrocytes and glial cells also participate in regulating inflammatory processes by producing cytokines, chemokines and neuropeptides involved in the pathogenesis of the disease. Neuroinflammatory processes also take place in the dorsal root ganglia and the central nervous system resulting in central sensitization. Locally activated immune cells and soluble molecules can also travel through the bloodstream contributing to the complex systemic effects. Together with the defect of the descending pain inhibitory routes, these changes lead to the alteration of pain processing. Chronic inflammation can result in irreversible structural damage and the presence of comorbidities can also play an important role contributing to pain. Mental disorders and the stress related to coping with the disease can affect pain modulation via the complex interactions between the regulation of stress and pain, whereas systemic diseases can also influence pain symptoms.

RA, rheumatoid arthritis; TNF-a, tumor necrosis factor-alpha; IL-1b, Interleukin-1 beta, IL-6, Interleukin-6; IL-17, Interleukin-17; IL-4, Interleukin-4, IL-10, Interleukin-10; PG, prostaglandin; CNS, central nervous system; CRPS, complex regional pain syndrome

Figure S195 was prepared with the help of BioRender (www.biorender.com)
